# Concurrent Linear Deracemization of Secondary Benzylic Alcohols via Simultaneous Photocatalysis and Whole-cell Biocatalysis

**DOI:** 10.1021/acscatal.5c04974

**Published:** 2025-08-18

**Authors:** W. Y. Wylan Wong, Stephen Wallace, Craig P. Johnston

**Affiliations:** † EaStCHEM, School of Chemistry, University of St Andrews, St Andrews, Fife KY16 9ST, United Kingdom; ‡ Institute of Quantitative Biology, Biochemistry and Biotechnology, School of Biological Sciences, 3124 University of Edinburgh, Edinburgh EH9 3FF, United Kingdom

**Keywords:** deracemization, photocatalysis, biocatalysis, photobiocatalysis, whole-cell

## Abstract

Photobiocatalysis enables remarkable synthetic transformations by combining the exquisite stereoselectivity of enzymes with the mild generation of high-energy intermediates by photocatalysis, but practical applications remain limited due to enzyme photodamage. The deracemization of secondary alcohols is a key model reaction for photobiocatalytic protocols due to the importance of the enantioenriched products. However, current strategies rely on the temporal separation of catalytic cycles to circumvent incompatibilities, precluding photobiocatalytic transformations that require the *in situ* generation of reactive intermediates. We report a single-step concurrent linear deracemization protocol by combining a water-soluble photocatalyst (sodium anthraquinone-2-sulfonate) with a promiscuous alcohol dehydrogenase (*Geotrichum candidum* acetophenone reductase) encapsulated in lyophilized microbial whole cells. Insights into enzyme selectivity and system dynamics from molecular docking and kinetic modeling guided the optimization of the multicomponent system. Our approach represents a modular and generalizable strategy for developing photobiocatalytic cascades operating under mutually compatible conditions, wherein spatial separation mitigates photodamage and enables simultaneous dual catalytic turnover.

## Graphical abstract

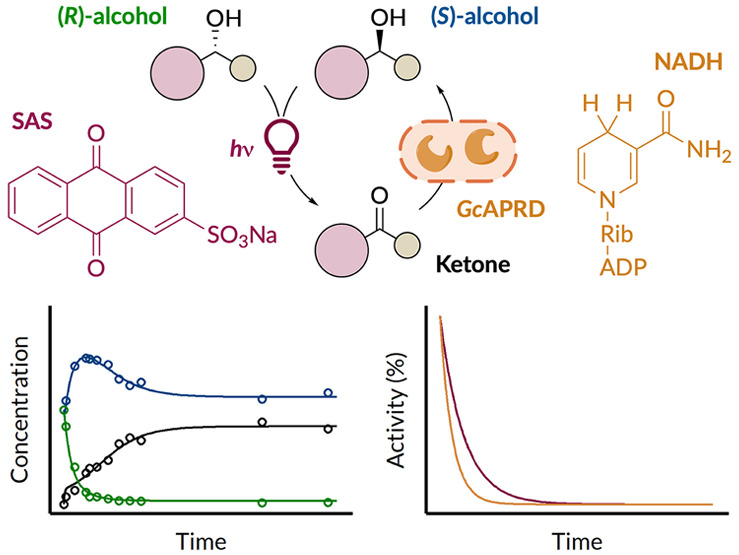

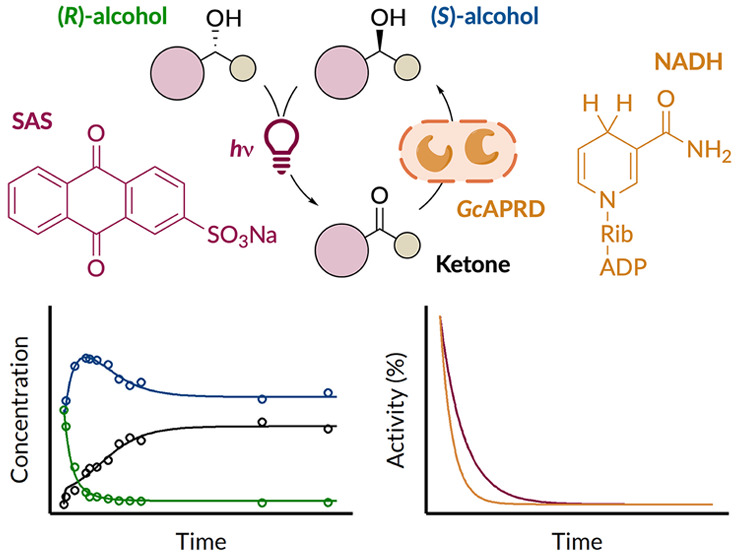

## Introduction

Photobiocatalysis is a field of growing interest as it offers a safer and more sustainable alternative to synthetic methods involving rare transition metals, toxic materials, high temperature or pressure, and multistep syntheses requiring purification or other manipulations between steps.^1,2^ Dual catalytic systems, such as the integration of photo- and biocatalytic cycles, enables reactivity that cannot be achieved by individual catalytic systems. Photocatalysis enables the facile generation of highly reactive intermediates with light as a “traceless reagent”, while biocatalysis confers excellent regio- and stereoselectivity under mild conditions. However, biocatalysts are prone to inactivation by irradiation, since photochemically generated reactive oxygen species (ROS) can degrade a wide range of side chain functional groups, limiting their combined synthetic utility.^3–9^ Advances in photocatalyst and protein engineering have mitigated the incompatibility by enabling milder light sources and improving enzyme photostability, while encapsulation, immobilization, flow reactors, and biphasic systems circumvent photodamage by spatially separating the catalytic cycles.^10–17^ While tailoring catalysts for specific classes of transformations demands significant experimental effort and iterative design, spatial separation represents a more modular and generalizable strategy that can greatly expand access to diverse photobiocatalytic transformations by enabling combinatorial catalyst pairing.^18,19^

Whole-cell biocatalysts (WCBs) naturally possess a cellular envelope, conferring a layer of controlled separation between cytoplasmic enzymes and the external environment. Within the envelope, reducing systems such as ROS-scavenging enzymes can mitigate the effects of photo-oxidative damage by neutralizing ROS and repairing oxidized residues.^4,20,21^ The shielding effect of residual cell wall materials extends to dead cells in harsh environments.^22^ The model organism, *Escherichia coli*, is an attractive chassis due to its simple culture conditions, genetic tractability, and low prevalence of endogenous photosensitizers as a Gram-negative species.^23,24^ The surprising resilience of *E. coli* cellular components to irradiation-induced oxidative damage was demonstrated by continual respiration for 1–2 h upon UV (ultraviolet) irradiation.^25^ Blue light irradiation has also been shown to induce a transiently nonproliferative yet still viable physiological state.^26^

Although the cellular envelope of WCBs presents a barrier for mass transport, it can also be leveraged as a scaffold for chemical modifications to enhance robustness, selective permeability, solvent and light tolerance, and even embed photocatalytic function.^27–29^ While chemical modifications can significantly enhance WCB performance, even unmodified WCBs exhibit sufficient biocatalytic activity under photochemical conditions in the presence of organic solvents. Additionally, lyophilization has been demonstrated as a reliable method to prepare bench-stable WCBs without damaging catalytic activity.^30^ This baseline resilience, coupled with scalability and simple preparation, positions unmodified WCBs as a practical and accessible alternative to purified enzymes in synthetic applications demanding simultaneous photo- and biocatalytic activity.

Park and coworkers developed *E. coli* WCBs expressing heme-dependent P450 monooxygenases, where Eosin Y binds and functions as a donor of photoexcited electrons to regenerate the oxidized heme group (Scheme 1A).^31^ While validating the application of WCBs under visible light irradiation, the role of the photocatalyst remained limited to replacing redox cofactors in this system. Recently, Zhong and coworkers developed a WCB based on *E. coli* expressing a benzophenone-containing dehalogenase, capable of catalyzing intracellular aryl hydrodehalogenation under violet light irradiation (λ = 380 nm) (Scheme 1B).^32,33^ Earlier examples of whole-cell photobiocatalysis, where photoexcited moieties directly participated in the target reaction, were often limited to artificial enzymes developed through extensive protein engineering,^34^ bioconjugation,^35–37^ and codon reassignment,^38^ as well as variants of the three naturally occurring enzymes containing photoactive prosthetic groups (DNA photolyase,^39^ chlorophyll f synthase,^40^ and fatty acid decarboxylase^41^). While the properties of the cellular envelope were potentially beneficial to the reactions, their precise role remained relatively underexplored.

**Scheme 1:**
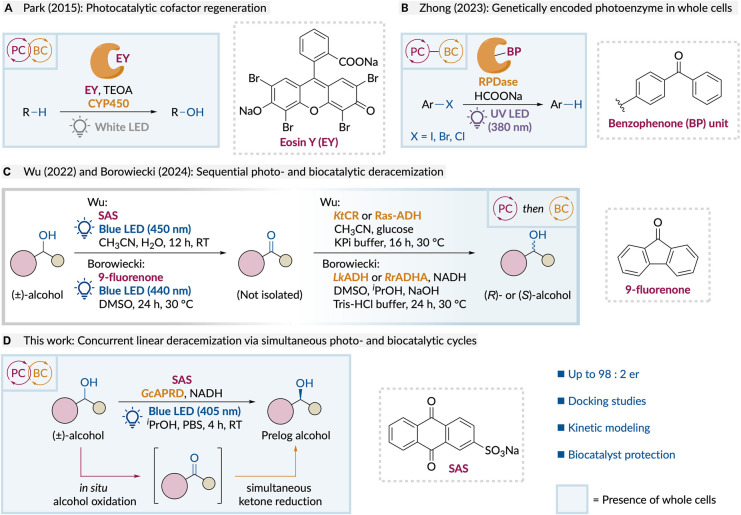
Current Approaches for Whole-Cell Photobiocatalysis

Combining separate photocatalytic and biocatalytic steps is a simpler and more modular approach to developing whole-cell photobiocatalytic reactions.^42^ Currently, enantioenriched secondary alcohols are popular targets in whole-cell photobiocatalytic protocols, owing to the extensive knowledge base of alcohol dehydrogenases (ADHs) and the prevalence of these products as chiral building blocks in the agrochemical, pharmaceutical, fine chemical, fragrance, and flavor industries.^43^ This dual catalytic strategy offers significant potential to address the challenges of deracemization, an attractive approach to obtaining enantioenriched secondary alcohols with high atom- and step-economy.^44^ Inspired by natural photosynthetic pathways, methodologies employing simultaneous photo- and biocatalytic turnover would enable the *in situ* generation of reactive species that undergo further transformation under biocatalytic control, unlocking otherwise challenging transformations due to the instability or difficulties in the handling of intermediates.

Despite the advantages of applying whole-cell photobiocatalysis for secondary alcohol deracemization, only three such procedures have been reported to date and none that employs concurrent dual catalysis (Scheme 1C). Wu and coworkers first demonstrated the photobiocatalytic deracemization of secondary alcohols (up to 99:1 er) by photo-oxidizing racemic alcohols using sodium anthraquinone-2-sulfonate (SAS), followed by the direct addition of *E. coli* WCBs expressing ADHs.^45^ This procedure could not be performed in a single step, which the authors attributed to the generation of ROS during the photocatalytic cycle inactivating the ADHs and was not rescued by the addition of catalase. Thus, this protocol required temporal separation of the two steps, along with drastic changes to the reaction media to accommodate the WCBs.

Borowiecki and coworkers expanded the substrate scope of this procedure and replaced acetonitrile with dimethyl sulfoxide (DMSO), to which ADHs generally exhibit better tolerance, enabling higher concentrations of organic substrates to be dissolved.^46^ While this system achieved excellent enantioselectivity (>99:1 er) for a range of substrates with varying electronic properties, it requires an O_2_ atmosphere, increasing operational complexity and safety risk, and necessitating separate steps due to solvent incompatibility. Specifically, 9-fluorenone-catalyzed photo-oxidation was water-sensitive while the bioreduction step was intolerant to high organic solvent content. Recently, Gotor-Fernández and coworkers reported a deracemization protocol for alcohols with an identical reaction design which further expands the scope to β-chlorohydrins, a challenging class of substrates to oxidize.^47^

On the other hand, the deracemization of alternative substrates using simultaneous photo- and biocatalytic cycles has been reported in the literature with some limitations. Wenger and coworkers demonstrated the formal asymmetric reduction of 1-pyrrolines to pyrrolidines using iridium- and ruthenium-based photocatalysts and *E. coli* WCBs expressing a monoamine oxidase.^48^ While the product differs from the starting substrate, this reaction proceeds through a network that utilizes the principles of cyclic deracemization. Later, Glueck, Winkler, Kroutil, and coworkers developed a protocol for the photobiocatalytic cyclic deracemization of sulfoxides using cell lysates containing methionine sulfoxide reductases alongside protochlorophyllide, a novel photocatalyst isolated from photosynthetic purple bacteria.^49^ Although an elegant framework, the protocol required a commercially unavailable photocatalyst produced by a complex biosynthetic pathway. This limitation was later addressed by the authors, who reported a new methodology using Eosin Y, a cheap and commercially available photocatalyst.^50^ However, the adapted protocol required temporally alternating photo- and biocatalytic cycles due to incompatibilities between Eosin Y and the externally supplied reductant. Overall, current methodologies suffer from poor compatibility between the photo- and biocatalytic steps, resulting in long reaction times (26–48 h) with additional manipulation between the separated steps. Furthermore, the lack of methodologies using both low cost and commercially available catalysts hampers their scale-up and economic feasibility.

In this work, we report a simultaneous photobiocatalytic system for the deracemization of secondary benzylic alcohols using recombinant *E. coli* expressing *Geotrichum candidum* acetophenone reductase (*Gc*APRD) as a photorobust WCB alongside SAS as a water-soluble photocatalyst (Scheme 1D). Two distinct, orthogonal catalytic cycles operating in opposite directions with external energy input are needed to offset the thermodynamically unfavorable decrease in entropy that accompanies the conversion of a racemic mixture into a single enantiomer, while also overcoming the kinetic constraint imposed by the principle of microscopic reversibility.^51^ The present protocol entails simultaneous alcohol oxidation and ketone reduction enabled by the photoprotective effect of whole-cell encapsulation, conferring enantioselectivity with the biocatalytic reduction step. Furthermore, to ensure the general applicability of this protocol, we rationalized the experimental results with molecular docking and kinetic modeling, leading to the derivation of a predictive framework to determine optimal reaction times for diverse substrates. This integrated approach can provide valuable insights for optimizing multicatalytic procedures containing multiple simultaneous fundamental reactions and variables.^52^

## Results and Discussion

### Photocatalytic Oxidation of Secondary Benzylic Alcohols

The investigation began with adapting an existing photocatalytic alcohol oxidation protocol to improve biocompatibility, focusing on tolerance toward biologically relevant buffers and media while employing nontoxic photocatalysts. We drew inspiration from studies on the photocatalytic oxidation of secondary benzylic alcohols using thioxanthone in DMSO and SAS in a water/toluene biphasic system.^53,54^ A range of photocatalysts was screened under modified conditions deemed biocompatible, using M9CA culture media with (±)-**1a** as a standard substrate for ease of analysis by quantitative ^19^F­{^1^H} NMR spectroscopy (Table 1). To our delight, four photocatalysts were able to catalyze the photo-oxidation reaction to give ketone **1b** in 30–75% yield in bacterial culture media (entries 2–5). Surprisingly, this contradicts earlier reports by Kokotos and coworkers, wherein DMSO, and other polar aprotic solvents to a lesser extent, were deemed indispensable in stabilizing the photoexcited singlet state of diaryl ketones, such as SAS and thioxanthone.^53,55^ Our results imply that monochromatic blue light-emitting diodes (LEDs), matching the absorbance peak of thioxanthone near 400 nm in water–ethanol mixtures, may be more efficient as a photon source to repopulate the excited state of the photocatalyst without the need for DMSO and less energy-efficient, high-powered compact fluorescent lamps (CFLs) in the protocol by Kokotos, although actinometric studies are required for verification.^56,57^ Furthermore, we verified the roles of ethanol as a cosolvent, O_2_ as an oxidant for SAS regeneration (Figures S2–S3), and light as an energy source, respectively (entries 11–13).

**Table 1:**
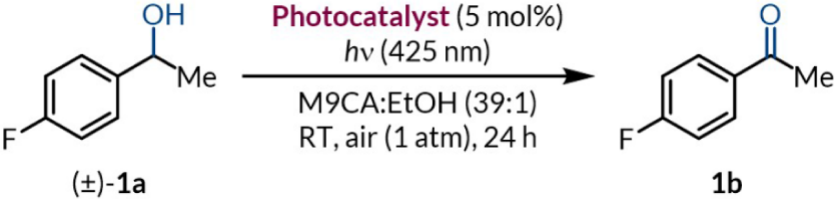
Photocatalyst Screening and Optimization^a^

Entry	Photocatalyst and variations	Yield (%)^b^
1	None	<1
2	SAS	75
3	Thioxanthone	51
4	Riboflavin	31
5	Phenazine	30
6	Phenazine ethosulfate	3
7	Rose Bengal	1
8	Neutral Red	<1
9	Safranin O	<1
10	Methylene Blue	<1
11	SAS, no EtOH	63
12	SAS, under N_2_	11
13	SAS, dark reaction	<1
14^c^	SAS, *h*ν (405 nm), PBS:^*i*^PrOH (17:3), 6 h	74

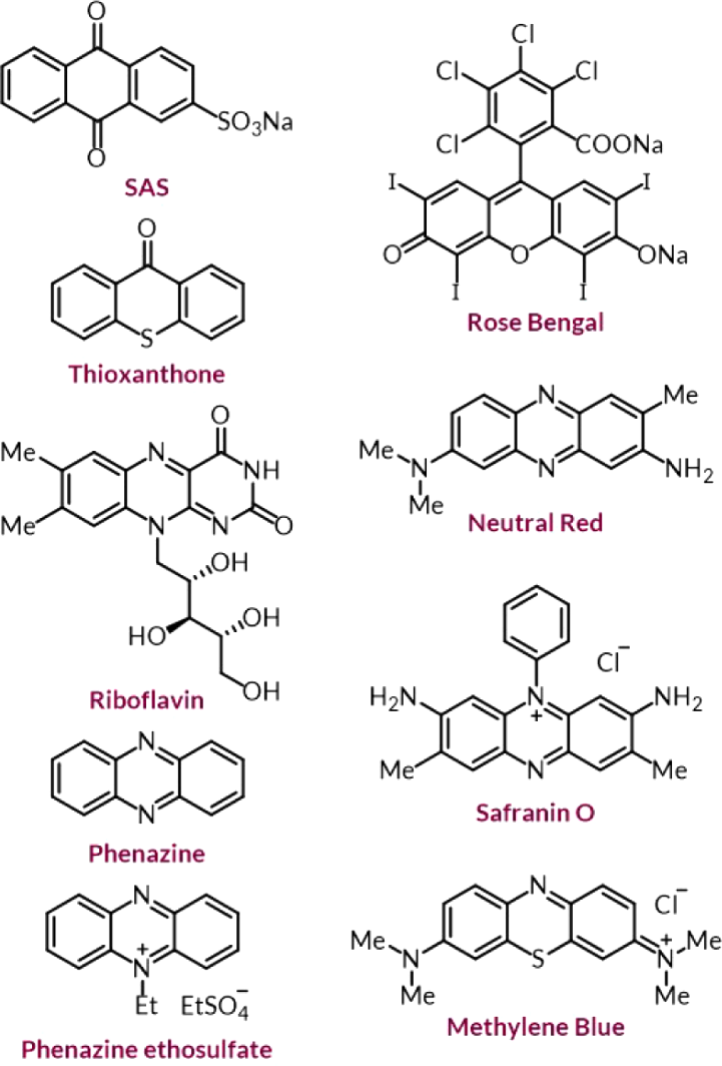

a Reaction conditions: (±)-**1a** (200 μmol), photocatalyst (10 μmol), M9CA media with 2% w/v glucose (600 μL total), ethanol (EtOH, 15 μL), blue LED (425 nm), room temperature, ambient atmosphere, 24 h.

b Yields were determined by ^19^F­{^1^H} NMR spectroscopy using 2,2,2-trifluoroethanol (TFE, 0.33 equiv) as an internal standard.

c Reaction conditions: (±)-**1a** (10 μmol), photocatalyst (0.5 μmol), ^*i*^PrOH (75 μL), PBS (pH 7.4, 425 μL), blue light (405 nm), room temperature, ambient atmosphere, 6 h.

Unfortunately, the yield was limited by the heterogeneous nature of the reaction, as the low cosolvent concentration was insufficient to fully dissolve (±)-**1a** (333 mM). Considering the toxicity of additional solvents toward *E. coli*, the overall concentration of solutes was lowered to ensure biocompatibility and solubility. Shorter wavelength light was preferred for improved spectral overlap with SAS absorption (Figure S4), thereby accelerating the reaction and minimizing light exposure time, potentially enhancing biocompatibility. M9CA media was replaced by phosphate-buffered saline (PBS) to further reduce media complexity. Without increasing photocatalyst loading, the optimized conditions afforded ketone **1b** with a similar yield (74%) after only 6 h (entry 14). Notably, ketone formation plateaued after 6 h (Figure S5), which suggests the upper yield limit to be approximately 75% (entries 2 and 14). Subquantitative yields were attributed to the degradation and slow regeneration of SAS under the reaction conditions, as shown by ultraviolet–visible (UV–vis) spectroscopy and mechanistic studies (Figures S2–S4). We observed the formation of an unidentified fluorinated side product that did not correlate with the decrease in SAS-catalyzed **1b** formation rate but required oxygen, implying its formation to be SAS-independent (Figure S5). We deduced that higher SAS loading would accelerate the desired reaction while compensating for photocatalyst degradation.

### Biocatalytic Reduction of Prochiral Benzylic Ketones

Having established a biocompatible photo-oxidation protocol, we proceeded to develop a whole-cell bioreduction process to complete the concurrent linear deracemization strategy. We commenced by screening WCBs for their ability to reduce ketone **1b** as a model substrate in the absence of auxiliary enzymes for cofactor recycling, with or without expressing heterologous ADHs reported in the literature (Table 2).^58–67^ The WCBs were based on lyophilized cells of *E. coli* and *Saccharomyces cerevisiae*, both of which endogenously produce a variety of ketone-reducing enzymes with broad substrate specificities and mostly Prelog stereopreference.^68,69^ With ketone **1b** as a substrate, Prelog stereopreference corresponds to the production of alcohol (*S*)-**1a** and vice versa.

**Table 2:**
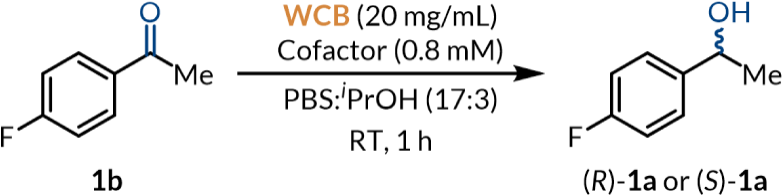
Biocatalyst Screening, Optimization, and Control Experiments^a^

Entry	WCB host/heterologous enzyme^b^	Cofactor	Yield (%)^c^	er [(*S*):(*R*)]^d^
1	None/None	NADH	0	-
2	*E. coli*/None	NADH	3	>99:1
3	*E. coli*/*Rr*ADHA	NADH	81	>99:1
4	*E. coli*/*Gc*APRD	NADH	93	>99:1
5	*E. coli*/*Eb*SDR8	NADH	35	2:98
6	*E. coli*/*Lx*CAR-S154Y	NADH	0	-
7	*E. coli*/*Lk*ADH	NADPH	0	-
8	*E. coli*/*Kt*CR	NADPH	0	-
9	*S. cerevisiae*/None	NADH	0	-
10	*E. coli*/*Gc*APRD	None	92	>99:1
11^e^	*E. coli*/*Gc*APRD	NADH	6	>99:1
12^f^	*E. coli*/*Gc*APRD	NADH	85	>99:1

a Reaction conditions: **1b** (10 μmol), lyophilized WCB (10 mg), cofactor (0.8 mM from stock), PBS (500 μL total), ^*i*^PrOH (75 μL), room temperature, ambient atmosphere, 1 h.

b *E. coli* refers to the strain BL21­(DE3).

c Yields were determined by ^19^F­{^1^H} NMR spectroscopy using TFE (0.33 equiv) as an internal standard.

d Enantiomeric ratios (er) were determined by high performance liquid chromatography (HPLC) on the appropriate chiral stationary phase. Absolute configuration was assigned based on the reported stereopreference of the corresponding biocatalysts.

e EtOH instead of ^*i*^PrOH.

f SAS (6 mM) dissolved in the reaction mixture, ambient light.

First, reduced nicotinamide adenine dinucleotide (NADH) could not reduce ketone **1b** without biocatalysts under standard conditions (entry 1). Only trace amounts of product (*S*)-**1a** were obtained from *E. coli* harboring an empty plasmid vector (pET-22b­(+)), presumably due to native enzymes being inefficient at reducing ketone **1b** or being expressed at low levels (entry 2). Encouragingly, the Prelog-specific enzymes *Rr*ADHA and *Gc*APRD afforded enantiopure product (*S*)-**1a** in very good to excellent yields in 1 h (entries 3–4). On the other hand, the anti-Prelog-specific *Eb*SDR8 afforded alcohol (*R*)-**1a** in poor yield (entry 5), potentially due to low catalytic efficiency with substrate **1b**, but with excellent enantioselectivity.^70^ Product **1a** was not observed using any other WCBs we screened (entries 6–9).^45,46,66^ Since soluble protein expression was verified by SDS-PAGE except for *Kt*CR (Figure S1), we attributed the results primarily to the catalytic activity of the enzymes.

Despite using WCBs, substrate-coupled cofactor recycling was necessary to support catalytic activity due to the lack of externally supplied energy sources (e.g., glucose). ^*i*^PrOH was shown to be an effective cosubstrate for NADH regeneration by *Rr*ADHA, *Gc*APRD, and *Eb*SDR8 without auxiliary enzymes in accordance with the literature (entries 2–4).^59,60,64^ *Lx*CAR-S154Y and *Lk*ADH exhibited ^*i*^PrOH-dependent NAD­(P)H regeneration activity in previous studies but did not support the reduction of ketone **1b** in the present system.^46^ *Kt*CR also failed to catalyze the reduction without auxiliary enzymes, in line with the absence of literature reports. We further characterized ^*i*^PrOH-dependent cofactor regeneration by *Gc*APRD due to its excellent yield and enantioselectivity corresponding very closely to observations by Matsuda and coworkers using WCBs containing *Gc*APRD (entries 10–11).^71 *i*^PrOH was indeed required for NADH regeneration in addition to its role as a cosolvent, hence it could not be replaced by ethanol. We were pleased to find that endogenous NADH in the WCB was sufficient for *Gc*APRD to reduce ketone **1b** in excellent yield, further indicating the effectiveness of the cofactor regeneration system. Regardless, supplying high concentrations of NADH (0.8 mM), at approximately 10-fold the intracellular concentration of glucose-fed *E. coli* in the exponential growth phase (83 μM), is a potential strategy to enhance the bioreduction rate when WCBs are applied in one-pot deracemizations in order to outcompete the photo-oxidation process.^72^ Remarkably, the presence of SAS (6 mM) under ambient light did not notably impact the yield of the reduction (entry 12), demonstrating the excellent biocompatibility of the photocatalyst in the ground state.

### Optimization of the Analytical Scale Whole-Cell Photobiocatalytic Deracemization

With the complementary photo-oxidation and bioreduction processes in hand, we subjected alcohol (±)-**1a** to a concurrent linear deracemization protocol under modified bioreduction conditions with added SAS and blue light irradiation (Table 3). Surprisingly, despite previous unsuccessful attempts to achieve simultaneous photocatalysis and whole-cell biocatalysis, our procedure furnished alcohol (*S*)-**1a** from its racemate in good yield with excellent enantioselectivity at significantly shorter reaction times compared to previous methods (entry 1).^45,46^ Accounting for changes in catalyst activity over the course of the 4 h reaction, the presence of ketone **1b** indicates a higher total turnover number (TTN) of the photocatalytic cycle than the biocatalytic cycle, rendering the alcohol/ketone ratio an important indicator of relative rates between the photo-oxidation and bioreduction processes. In addition, the formation of the unidentified side product (*vide supra*) was prevented by incorporating WCBs regardless of ADH expression, as cell bodies can scatter light and reduce effective photon input, suppressing the undesired side reaction.

**Table 3:**
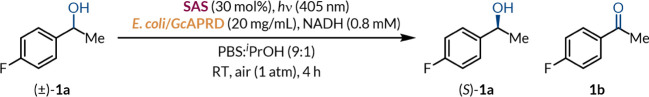
Optimization for Concurrent Linear Deracemization^a^

Entry	Variation from standard conditions	**1a** (%)	**1b** (%)	**1a:1b**	er ((*S*):(*R*))^b^
1	None	56	17	77:23	96:4
2	No SAS	96	4	96:4	50:50
3	Dark reaction	84	11	88:12	56:44
4	EtOH instead of ^*i*^PrOH	1	87	1:99	-
5	*E. coli*/Empty vector	0	>99	0:100	-
6	No NADH	68	31	69:31	94:6
7	NADH (1.5 mM)	65	22	75:25	96:4
8	^*i*^PrOH (5%)	48	29	62:38	98:2
9	^*i*^PrOH (15%)	75	21	78:22	95:5
10	*E. coli*/*Gc*APRD (30 mg/mL)	58	16	78:22	97:3
11	*E. coli*/*Gc*APRD live cell suspension	76	24	76:24	94:6
12	SAS (5 mol %)	84	8	91:9	75:25
13	SAS (20 mol %)	82	18	82:18	82:18
14	SAS (35 mol %)	38	62	38:62	97:3
15	*h*ν (365 nm)	83	17	83:17	88:12
16	*h*ν (380 nm)	75	17	82:18	94:6
17	*h*ν (425 nm)	59	27	69:31	93:7
18^c^	Adding superoxide dismutase (1 mg/mL)	54	17	76:24	96:4
19^d^	Adding catalase (8 mg/mL)	57	15	79:21	96:4

a Reaction conditions: (±)-**1a** (10 μmol), SAS (6 mM from stock), lyophilized *E. coli*/GcAPRD (10 mg), NADH (0.8 mM from stock), PBS (500 μL total), ^*i*^PrOH (50 μL), blue LED (405 nm), room temperature, ambient atmosphere, 4 h. Yields of (*S*)-**1a** and **1b** were determined by ^19^F­{^1^H} NMR spectroscopy using TFE (0.33 equiv) as an internal standard.

b Enantiomeric ratios (er) were determined by high performance liquid chromatography (HPLC) on the appropriate chiral stationary phase. Absolute configuration was assigned based on the reported stereopreference of the corresponding biocatalysts.

c Lyophilizate from bovine erythrocytes (Merck).

d Lyophilizate from bovine liver (Merck).

The contribution of each catalytic cycle was confirmed by removing essential components. As expected, without SAS or irradiation, there was little to no oxidation, resulting in minimal formation of ketone **1b** and no enantioenrichment (entries 2–3). Meanwhile, without *Gc*APRD or ^*i*^PrOH for NADH recycling, photo-oxidation proceeded to completion, outcompeting the very limited bioreduction cycle (entries 4–5). In line with results from the standalone bioreduction process, endogenous NADH was able to catalyze the reduction, though with a reduced alcohol/ketone ratio. Since alcohol (*S*)-**1a** produced by *Gc*APRD remained susceptible to photo-oxidation in the concurrent linear deracemization reaction, the bioreduction process required a higher TTN to outcompete photo-oxidation. Therefore, additional NADH supply was necessary to increase the alcohol/ketone ratio, but further improvement in alcohol/ketone ratio above 0.8 mM NADH was accompanied by decreased enantioenrichment (entries 6–7). Increasing ^*i*^PrOH concentration up to 15% (v/v) showed a similar trend of increasing alcohol/ketone ratio at the expense of reduced enantioselectivity (entries 8–9), corroborating previous studies on isolated *Gc*APRD by Matsuda and coworkers.^73^ Despite the excellent catalytic activity and stability of *Gc*APRD with ^*i*^PrOH concentrations up to 30% (v/v), the cosubstrate competes with ketone **1b** for the substrate binding site, providing no benefit to the bioreduction rate beyond the enzyme saturation concentration.^74^ On the other hand, significantly increasing WCB loading had the simultaneous effect of scattering light and decreasing photo-oxidation turnover frequency (TOF), but it was superfluous as it only led to marginal improvements in the alcohol/ketone ratio and enantiomeric ratio (entry 10). The effect of lyophilization on the reaction compared to freshly prepared cell suspensions was also minimal (entry 11).

An alternative strategy to finetune the relative rates of each catalytic cycle was modifying the TOF of the photo-oxidation process. This can be achieved by varying SAS loading or changing the irradiation wavelength. As expected of the reaction design, there is a clear trade-off between further enantioenrichment and maintaining a high alcohol/ketone ratio with increasing SAS loading, where higher oxidation rates drove overall deracemization at the expense of outcompeting bioreduction (entries 12–14). The optimized SAS loading (30 mol %) represented the most effective balance (Figure S8), contributing to accelerated photo-oxidation. On the other hand, irradiation at increasing wavelengths displayed an unexpected trade-off. Despite the SAS absorbance peak at 335 nm under these solvent conditions (Figure S4A), longer wavelength light seemingly promoted photo-oxidation, thereby accelerating the enantioenrichment at the expense of alcohol/ketone ratio (entries 15–17). This might either be ascribed to higher photon fluxes provided by the LEDs at longer wavelengths (Table S4) or higher TTN for the photocatalyst as a result of slower photocatalyst degradation. Altogether, 405 nm was identified to be the optimal compromise as the longest wavelength without a significant decline in the alcohol/ketone ratio. Longer wavelength visible light was desirable to suppress side reactions, chiefly the photodecomposition of the photo- and biocatalysts.^75^

The exogenous addition of lyophilized superoxide dismutase (SOD) or catalase did not lead to improvements in proportional alcohol yields (entries 18–19). This either suggests endogenous SOD and catalase activity in the WCB to be in significant excess compared to the exogenous enzymes, or that superoxide anions and hydrogen peroxide do not represent the key extracellular ROS involved in biocatalyst degradation, though intracellular pathways in this system remain unclear. Notably, within the reaction time scale, we observed the degradation of SAS (Figure S4) and inferred the loss of catalytic activity by *E. coli*/*Gc*APRD as the level of ketone **1b** and both enantiomers of alcohol **1a** plateaued (Figure 1). Biocatalyst degradation under irradiation in the presence of photocatalysts and organic solvents is a widely reported phenomenon.^76–78^ Meanwhile, the photocatalyst degradation we observed concurred with the rapid α-hydroxylation of SAS upon irradiation at 365 nm in oxygenated aqueous solution reported by Phillips and coworkers, which drastically decreased its triplet excited state energy based on density functional theory calculations and effectively disabled its photocatalytic properties.^79,80^ Dimerization of long-lived excited state ketyl radicals is also a well-known degradation pathway of carbonyl-containing photocatalysts.^81^

**Figure 1:**
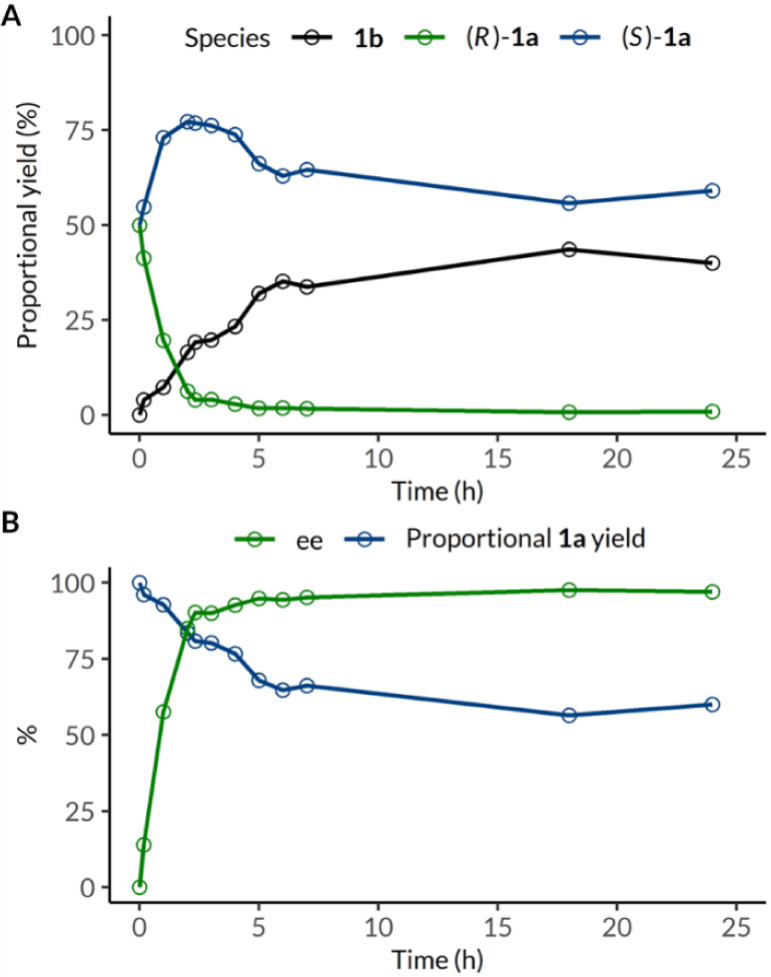
Kinetic profile of the concurrent linear deracemization of alcohol (±)-**1a** under standard conditions with respect to the proportional yield of each species (**A**) or that of total alcohol **1a** and its enantiomeric excess (**B**).

Inspection of the reaction design revealed the interplay between timing and catalyst loading when accounting for degradation. Reaction trajectories showed the gradual accumulation of ketone **1b**, indicating a higher TOF for photo-oxidation than bioreduction during 0–6 h (Figure 1). Furthermore, the net consumption of alcohol (*S*)-**1a** after 2 h indicates a faster decline in the catalytic activity of *E. coli*/*Gc*APRD than of SAS. To maximize the alcohol/ketone ratio and enantioenrichment, the reaction must proceed for as long as possible before the turnover of the biocatalytic cycle becomes significantly slower than that of the photocatalytic cycle. After extensive optimization (Figures S8–S10), 30 mol % and 4 h were selected as the SAS loading and reaction time, respectively.

### Biocatalyst Protection by Whole-Cell Encapsulation

The protective effect of whole-cell encapsulation was investigated by comparing the decrease of biocatalytic activity between total cell lysates and whole-cell lyophilizates. To induce ROS generation, alcohol (±)-**26a** was added as a sacrificial substrate to turnover the photocatalytic cycle without the ketone product competing for *Gc*APRD binding. After 2 h irradiation, a more significant decline in catalytic activity was observed in total cell lysates (57%) than lyophilized WCBs (24%), demonstrating a moderate level of photodamage mitigation by encapsulation (Figure 2). While lower reduction yields for ketone **1b** were observed for unirradiated WCBs compared to lysates due to the mass transfer barrier imposed by the cellular envelope, the longevity of irradiated *Gc*APRD is crucial to the reaction. On the other hand, the low colony-forming unit count of lyophilized WCB suggests culturability is unimportant to its catalytic activity (Table S6). Although lyophilized cells are not replicating, they are metabolically active upon rehydration and may support the regeneration of damaged cellular components.^82^ Further studies are also required to elucidate the degree and types of photodamage suffered by *Gc*APRD in different biocatalyst preparations.

**Figure 2:**
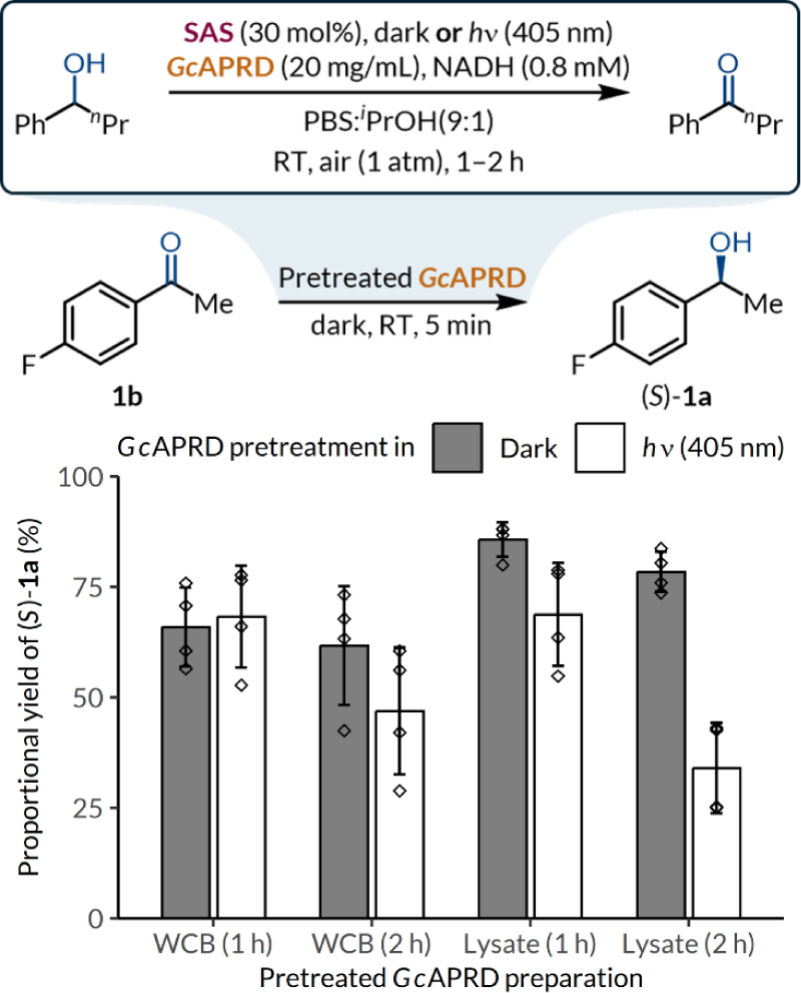
Effect of encapsulation on biocatalyst degradation. Diamonds (◊) denote individual data points and error bars indicate standard deviation (*n* = 4).

### Scope and Limitations

To assess the general applicability of this facile deracemization protocol, we explored the substrate scope by varying the sterics and electronics of the aromatic ring and the aliphatic group (Scheme 2). First, hydrogen and small halogen substituents on the *para-* and *meta-*positions were well-tolerated ((*S*)-**1a**–**2a,** (*S*)-**4a**–**5a**, and (*S*)-**7a**–**8a**), while *ortho*-substitution resulted in significantly less enantioenrichment and a decreasing alcohol/ketone ratio with increasing steric bulk ((*S*)-**3a,** (*S*)-**6a**, and (*S*)-**9a**). Since SAS-catalyzed photo-oxidation undergoes a proton-coupled electron transfer (PCET) or hydrogen atom transfer (HAT) step from the substrate benzylic position to the SAS radical cation (SAS^•+^), steric hindrance by *ortho*-substituents could disfavor photo-oxidation compared to the corresponding *para*-substituents (Table 4, entries 1–2).^83^ While ADH-catalyzed reductions are generally facilitated by electron-withdrawing substituents, the bioreduction rate similarly suffered when bulkier *ortho*-substituents block NAD­(P)­H-dependent hydride addition.^84^

**Scheme 2:**
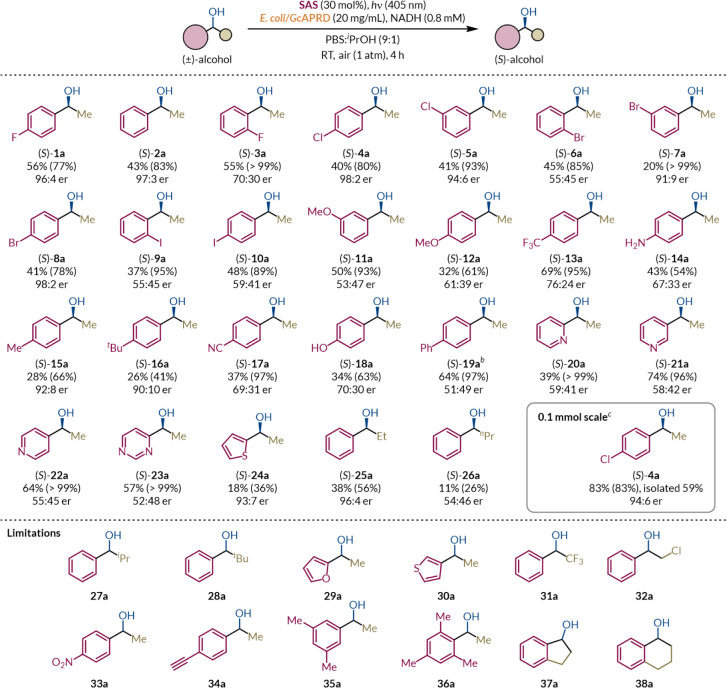
Substrate Scope for the Concurrent Linear Deracemization of Secondary Benzylic Alcohols[Fn sch2-fn1]

a
Reaction conditions: (±)-alcohol (10 μmol), SAS (6 mM from stock), lyophilized *E. coli*/*Gc*APRD (10 mg), NADH (0.8 mM from stock), PBS (500 μL total), ^*i*^PrOH (50 μL), blue LED (405 nm), room temperature, ambient atmosphere, 4 h. Yields of (*S*)-**1a**, (*S*)-**3a**, and (*S*)-**13a** determined by ^19^F­{^1^H} NMR spectroscopy using TFE (0.33 equiv) as an internal standard. Yields of the remaining products determined by ^1^H NMR spectroscopy using *p*-xylene (0.25 equiv) as an internal standard. Yields as proportions of total extracted product are quoted in parentheses. Er determined by HPLC on appropriate chiral stationary phases. ^*b i*^PrOH (75 μL). ^*c*^Reaction conditions: (±)-**4a** (0.1 mmol), SAS (6 mM from stock), lyophilized *E. coli*/*Gc*APRD (100 mg), NADH (0.8 mM from stock), PBS (5 mL total), ^*i*^PrOH (500 μL), blue LED (405 nm), room temperature, ambient atmosphere, 4 h.

**Table 4:**
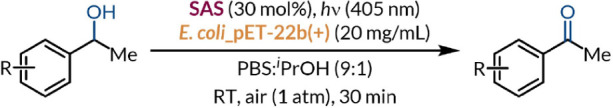
Effect of Substituent Position and Electronic Properties on the Photo-Oxidation^a^

Entry	Ketone product (R group)	Yield (%)^b^
1	**1b** (*p*-F)	38 (48)
2	**3b** (*o*-F)	8 (10)
3	**11b** (*m-*MeO)	8 (10)
4	**12b** (*p-*MeO)	19 (28)
5	**13b** (*p-*CF_3_)	12 (14)

a Reaction conditions: (±)-alcohol (10 μmol), SAS (30 mol %), PBS (500 μL total), ^*i*^PrOH (50 μL), blue LED (405 nm), room temperature, ambient atmosphere, 30 min.

b Yields of **11b** and **12b** were determined by ^1^H NMR spectroscopy using *p*-xylene (0.25 equiv) as an internal standard. Yields of the remaining products were determined by ^19^F­{^1^H} NMR spectroscopy using TFE (0.33 equiv) as an internal standard. Yields as proportions of the total extracted product are quoted in parentheses.

Substrates with electronically neutral and mildly electron-withdrawing substituents attained the highest degree of enantioenrichment, while higher proportional alcohol yields were obtained from electron-deficient substrates (Figure 3).^85,86^ Electron-donating substituents increase electron density at the benzylic position, thereby promoting photo-oxidation by stabilizing the benzylic radical intermediate via spin delocalization and favoring PCET or HAT toward the electron hole in SAS^•+^.^83,87^ In contrast, electron-withdrawing substituents render the benzylic position electron deficient, thermodynamically favoring nucleophilic hydride addition, hence promoting bioreduction.^88,89^ As a result, strongly electron-deficient substrates ((*S*)-**13a**, (*S*)-**17a**, and (*S*)-**20a**–**23a**) with low photo-oxidation rates generally resulted in high proportional alcohol yield with low enantioenrichment. Meanwhile, electron-rich substrates ((*S*)-**12a**, (*S*)-**14a**–**16a**, (*S*)-**18a**, and (*S*)-**24a**) with low bioreduction rates generally gave rise to reduced proportional alcohol yield but higher enantiomeric excess depending on their electron density. Substrates with low solubility in aqueous media ((*S*)-**10a** and (*S*)-**19a**) did not follow this trend, as they were less accessible to both catalytic cycles even with increased isopropanol concentration and remained largely unreacted (Table S5). Intriguingly, *meta*- and *para*-methoxy substituted substrates ((*S*)-**11a**–**12a**) were also outliers to the trend based on electronic effects at the benzylic position, suggesting important roles for steric and additional electrostatic interactions. When considering the photo-oxidation process, electron-deficient substrates clearly display lower oxidation rates (Table 4). Surprisingly, with only a mildly electron-withdrawing substituent, alcohol (±)-**11a** was oxidized at a lower rate than substrate (±)-**13a** with a strong electron-withdrawing group (entries 3 and 5). Moreover, electron-rich arene (±)-**12a** showed an unexpectedly lower oxidation rate compared to electronically neutral arene (±)-**1a** (entries 1 and 4). In addition to steric and electronic effects, the methoxy groups on anisoles (±)-**11a** and (±)-**12a** may, in competition with the benzylic C–H bond, undergo HAT with photoexcited SAS in the presence of Na_2_HPO_4_ as a base, which is a major ingredient of PBS.^90^

**Figure 3:**
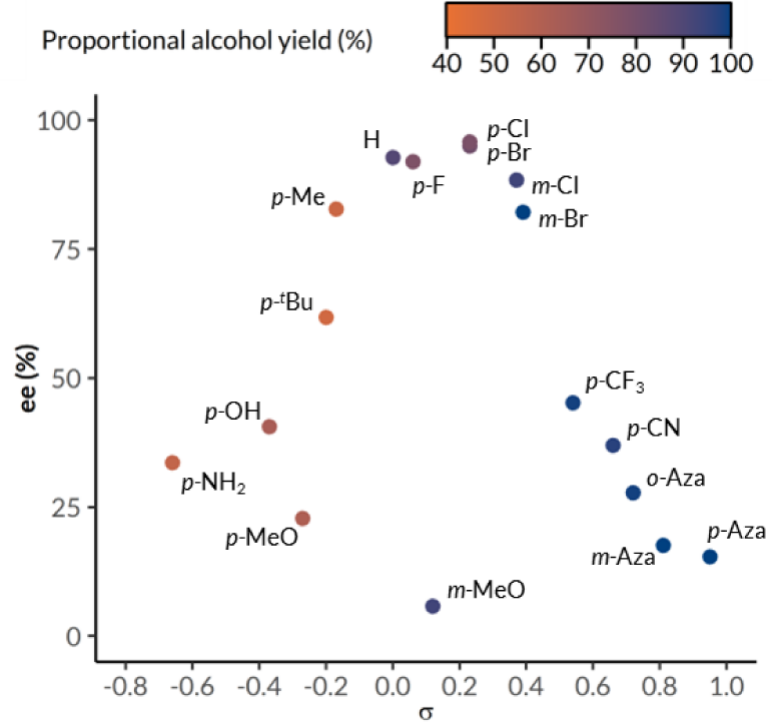
Effect of aryl substituents and heteroaromatic groups on enantiomeric excess (ee) and proportional alcohol yield. Aza denotes aza-substitution on the aromatic ring instead of phenyl substituents. Hammett constants (σ) were obtained from Hansch and Deady. Data for products (*S*)-**10a** and (*S*)-**19a** are omitted.

On the other hand, yields from the *Gc*APRD-catalyzed bioreduction did not fully adhere to the typical trends observed for nonenzymatic ketone reduction (Table 5).^89^ Specifically, the yields of alcohols were expected to increase for electron-deficient substrates, a trend followed by fluoro and methoxy substituents (entries 1–4). To our surprise, the electron-deficient ketone **13b** afforded a lower yield than the electronically neutral ketone **1b** (entry 5). This observation was attributed to the additional influence of binding kinetics, governed by steric effects and electrostatic interactions in the enzyme’s large and small binding pockets.

**Table 5:**
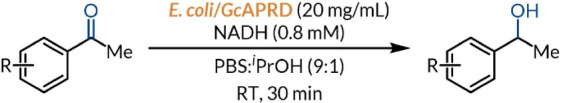
Effect of Substituent Position and Electronic Properties on the Bio-reduction^a^

Entry	Alcohol product (R group)	Yield (%)^b^
1	**1a** (*p*-F)	82 (91)
2	**3a** (*o*-F)	84 (>99)
3	**11a** (*m-*MeO)	61 (95)
4	**12a** (*p-*MeO)	51 (60)
5	**13a** (*p-*CF_3_)	72 (75)

a Reaction conditions: ketone (10 μmol), lyophilized WCB (10 mg), NADH (0.8 mM from stock), PBS (500 μL total), ^*i*^PrOH (50 μL), room temperature, ambient atmosphere, 30 min.

b Yields of **11a** and **12a** were determined by ^1^H NMR spectroscopy using *p*-xylene (0.25 equiv) as an internal standard. Yields of the remaining products were determined by ^19^F­{^1^H} NMR spectroscopy using TFE (0.33 equiv) as an internal standard. Yields as proportions of the total extracted product are quoted in parentheses.

Next, we investigated the tolerance of different alkyl groups in the small pocket. Increasing the length of the linear alkyl chain ((*S*)-**25a**–**26a**) led to substantially decreased proportional alcohol yield, while enantioinduction was effectively abolished upon increasing the chain to an *n*-propyl group. Unlike the thermodynamically challenging electron-rich substrates, the diminishing bioreduction rates for substrates with longer aliphatic chains are explained by binding kinetics. Consistent with previous studies, *Gc*APRD only tolerates aliphatic chains of up to two carbons due to a bulky aromatic residue, Trp288, restricting space in the small binding pocket despite favorable C­(sp^3^)–H···π interactions (Figure 4).^91,92^ While the Trp288Ala mutation enables the reduction of ketones with two bulky substituents, it also removes a major contributor to the enantioselectivity of *Gc*APRD, which suffers significantly even in the double mutants with an expanded large pocket reported by Matsuda.^92–94^ However, this remains an interesting direction to explore for increasing enzyme photostability by removing photolabile residues, such as Trp, from the active site.^5^

**Figure 4:**
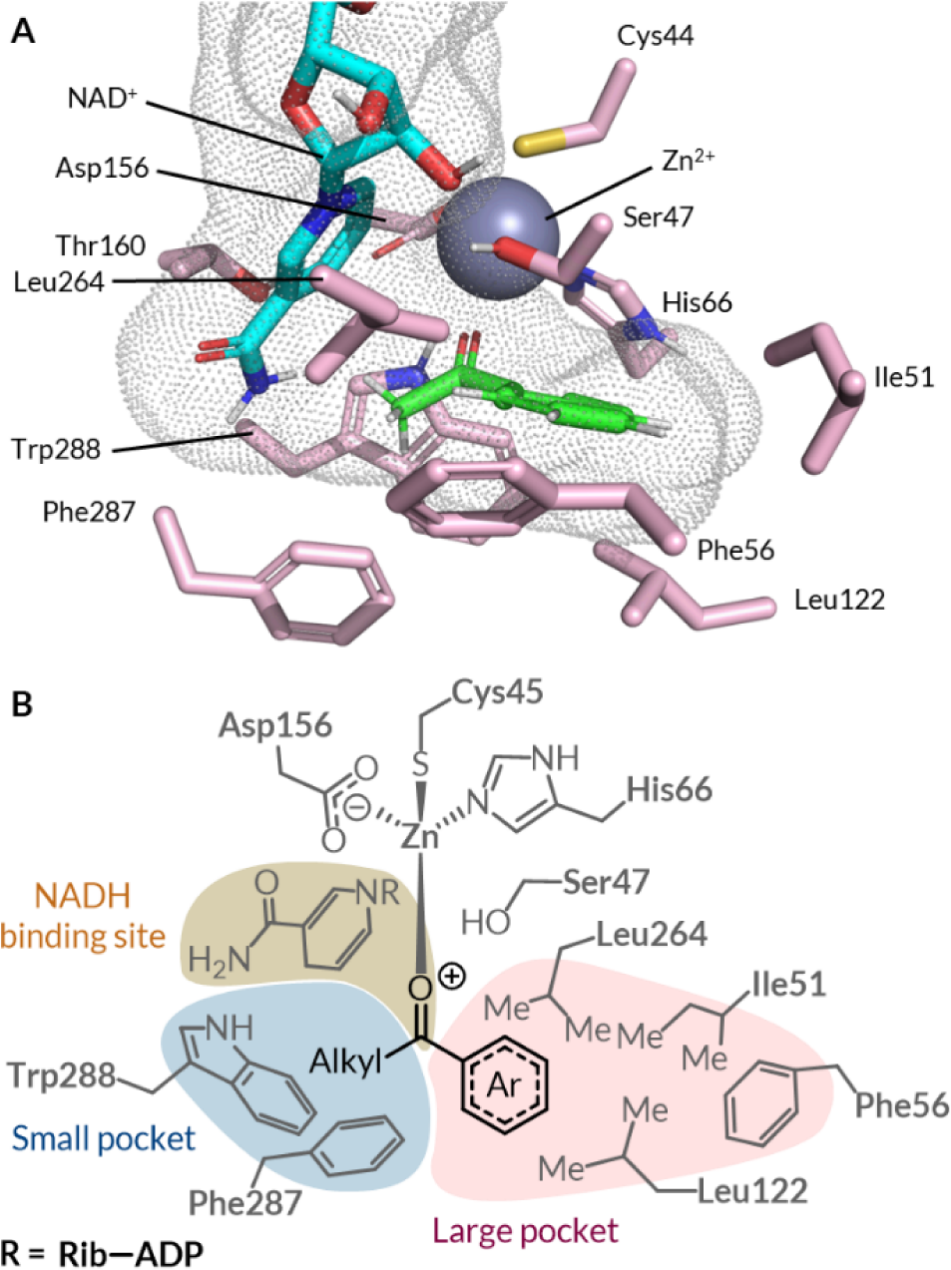
Simulated structure of the *Gc*APRD-**2b** complex by flexible docking (**A**) and the general structure of the *Gc*APRD-ketone complex (**B**). The binding site is shown as a dotted internal surface. Nonpolar hydrogens are omitted for clarity except for ketone **2b**. Rib, ribose; ADP, adenosine diphosphate.

Finally, we demonstrated the scalability of this batch photochemical procedure using alcohol (±)-**4a** on a 0.1 mmol scale by obtaining similar results to the analytical 10 μmol scale, with very good proportional alcohol yield, excellent enantioselectivity, and moderate isolated yield. Unfortunately, as a dual catalytic system, the substrate scope was inherently limited by both SAS and *Gc*APRD. Aryl groups strongly favored PCET or HAT at benzylic positions via stabilization of the resulting radical, driving the competing C–H oxidation for alcohols with additional benzylic C–H bonds ((±)-**15a**, (±)-**35a**–**38a**)). Likewise, branched alkyl ((±)-**27a**–**28a**), 2-furyl ((±)-**29a**), 3-thienyl ((±)-**30a**), benzylic trifluoromethyl ((±)-**31a**), α-chloro ((±)-**32a**), and alkynyl ((±)-**34a**) groups were not tolerated and led to substrate degradation, although further screening may reveal suitable photocatalysts for these substrates, such as in the case of (±)-**32a** as shown by Gotor-Fernández and co-workers.^47^ Substituting the methyl group with isopropyl or *tert*-butyl groups ((±)-**27a**–(±)-**28a**) resulted in proportional benzyl alcohol yields of 62% and 67%, respectively (Figure S11). This suggests competitive pathways for the ketone intermediates, which potentially undergo the Norrish type I reaction, i.e., α-scission between the benzylic carbon and the more stabilizing group, either via direct photoactivation or catalyzed by SAS.^95,96^ For branched alkyl phenyl ketones, the Norrish type I reaction could readily generate benzoyl (PhCHO^•^) and branched alkyl radicals with inductive stabilization, outcompeting their extremely kinetically hindered *Gc*APRD-catalyzed reduction.^71^ PhCHO^•^ could then be quenched by HAT and subsequently reduced by *Gc*APRD to form benzyl alcohol.^97^ Meanwhile, the 3-thienyl group can form a stabilized thienyl radical, promoting its cleavage from the benzylic carbon and leading to nearly complete substrate degradation. Additionally, the aryl *p*-nitro substituent ((±)-**33a**) was not tolerated and resulted in a mixture of products, consistent with the reduction of nitroarenes to nitrosoarenes by ^*i*^PrOH under blue light as observed by Yan and coworkers.^98^

### Molecular Docking Studies and Bioinformatic Analyses

To rationalize the experimental observations regarding bioreduction, we performed computational analyses to elucidate the structural factors governing *Gc*APRD-substrate interactions (see Supporting Information section 5.2 and Scheme S2 for computational details). Rigid molecular docking was initially performed to obtain a plausible binding pose for ketone **2b** in *Gc*APRD (PDB accession: 6ISV),^93^ selected for its consistency with the experimentally observed product, alcohol (*S*)–**2a**, and homologues with existing crystal structures of enzyme–substrate complexes (PDB accession: 3WNQ and 3WLF) (Figure S6A).^99–101^ Referencing the simulated *Gc*APRD-**2b** complex, we identified eight residues within 4 Å distance of the substrate in addition to the three residues coordinating to Zn (Figure 4). Feasible binding poses for some substrates, including ketone **2b**, were identified by flexible docking with rotational freedom for seven of the pocket residues excluding Thr160 (Figures 4A and S6B). Poses were selected for realistic Zn-coordination distances within 2.5 Å and an orientation permitting hydride attack from NADH on the *Re*-face of the ketone.^102^ The large pocket was predicted to accommodate *ortho-*substituted arenes (**3b** and **6b**). Arenes with small *meta*- and *para-*substituents (**1b**, **4b**, **5b**, **7b, 11b**, **12b**, **14b**, **17b**, and **18b**) were also accepted, either in the large pocket with torsional movements of the pocket residues or extending to the binding site entrance, implying induced fit behavior.^103^ No feasible binding poses were predicted for some of the arenes with bulkier substituents (**8b**, **10b**, **16b**, and **19b**). Competitive, unproductive binding poses were predicted for several ketones with alternative lone pair donors (**4b**, **6b**, **7b**, **11b**, **12b**, **17b**, **18b**, **20b**–**23b**), while ketones with a less pronounced steric difference between the alkyl and aryl groups (**25b**–**26b**) were not predicted to occupy the small pocket, suggesting additional steric and electrostatic factors hindering bioreduction rates.

As *Gc*APRD belongs to the highly structurally conserved Zn-dependent medium-chain dehydrogenase/reductase (MDR) superfamily with an almost exclusive Prelog stereopreference, conservation is expected in the chemical properties of the key amino acid residues controlling substrate specificity and enantioselectivity across the superfamily.^104–106^ The degree of conservation of the binding pocket residues in the MDR superfamily was probed by a multiple sequence alignment of the 3,477 protein sequences in the NCBI Conserved Domain Database entry (CDD entry: cd08254) containing *Gc*APRD (Table S3).^107^ The Zn-coordinating, cofactor-binding, and catalytic residues were found to be highly conserved as expected. In contrast, the large pocket was formed from residues with limited conservation among members of the MDR superfamily that possess a similar substrate specificity. Biased mutation hotspots, such as the above average distribution of bulky hydrophobic residues in this case, have been shown to play a critical role in controlling substrate specificity and stereoselectivity.^99^

Notably, substrate binding poses observed in molecular docking studies suggest that *Gc*APRD may better tolerate *para*-substituents contributing to attractive π-interactions with Phe56 in the large pocket (Figure 4). These include C­(sp^2^)–H···π, C­(sp^3^)–H···π, O–H···π, N–H···π, and halogen C–Cl···π and C–Br···π interactions.^108–114^ Meanwhile, *n*→π* and C–F···π interactions, primarily based on electrostatic rather than van der Waals forces, are limited to electron-deficient π-systems.^115,116^ Hence, they are unlikely to stabilize the binding of ketones **11b**, **12b**, and **13b** to *Gc*APRD in the large pocket containing the electron-rich benzyl group of Phe56. Matsuda and coworkers recently demonstrated that the Phe56Ile mutation expands the large pocket to accommodate bulkier substrates without sacrificing enantioselectivity, verifying its role in substrate binding in the large pocket.^94^

To probe other mutations that can potentially expand the substrate scope, we targeted the residues with limited conservation for *in silico* site-directed mutagenesis and performed flexible docking on the mutants with challenging substrates (Scheme S2). Saturation mutagenesis at position 56 predicted the Phe56Ser, Phe56Thr, and Phe56Tyr mutants to stabilize the productive binding pose for substrates containing methoxy substituents through hydrogen bonding (Figure S7), although pyridyl and pyrimidyl groups remained strongly coordinated to Zn. No other improved single, double, or triple mutant was identified among the residues Ile51, Phe56, Leu122, and Leu264. Previous structural investigations of Zn metalloenzymes of the MDR family have also revealed the highly dynamic nature of the binding site and the presence of a catalytic water molecule in a pentacoordinate Zn intermediate.^117^ This highlights the need for polarizable force fields in the elucidation of *Gc*APRD-substrate interactions, as simple scoring functions, such as the one employed in this study, often neglect important factors such as nonelectrostatic interactions.^118^ Meanwhile, larger-scale behaviors like induced fit effects require molecular dynamics investigations as docking algorithms only provide limited flexibility, but this is beyond the scope of the current study.^119,120^

### Kinetic Modeling for the Optimization of Multicatalytic Systems

Time course studies revealed the time sensitivity of the reported reaction design as the nonselective oxidative catalytic cycle counterproductively depletes the desired product enantiomer. Hence, effective deracemization with simultaneous oxidative and reductive cycles can only occur if reduction kinetically outcompetes oxidation. Fine-tuning of the two catalytic cycles is further complicated when accounting for catalyst degradation and the redox potential of different substrates, all of which influence the TTN of each catalytic cycle.

Isolating the properties of the photo-oxidation and bioreduction processes within the concurrent linear deracemization system can be very informative for optimization, but obtaining isolated parameters via direct measurements is an arduous process.^52^ To facilitate the characterization and optimization of reaction parameters hidden in this multicomponent reaction, we developed a deterministic kinetic model. It describes the evolution of five species in the reaction, namely the (*R*)–alcohol, the (*S*)-alcohol, the ketone intermediate, the photocatalyst SAS, and the biocatalyst *Gc*APRD using ordinary differential equations (ODEs) (Figures 5A and S12). The photo-oxidation process was characterized by three parameters describing photocatalyst excitation, effective reactivity, and degradation. The photocatalyst excitation parameter was fixed as a constant since it is independent from substrate properties other than photon absorption in the same range as the photocatalyst. The bioreduction process was characterized by three more parameters, describing biocatalyst degradation in addition to the two classical Michaelis–Menten parameters (Michaelis constant and maximum initial velocity).^121^ Realistic estimates for parameter values were obtained by experimentally measuring the individual components of the deracemization of (±)-**1a** under standard conditions (Figures S4 and S13).

**Figure 5:**
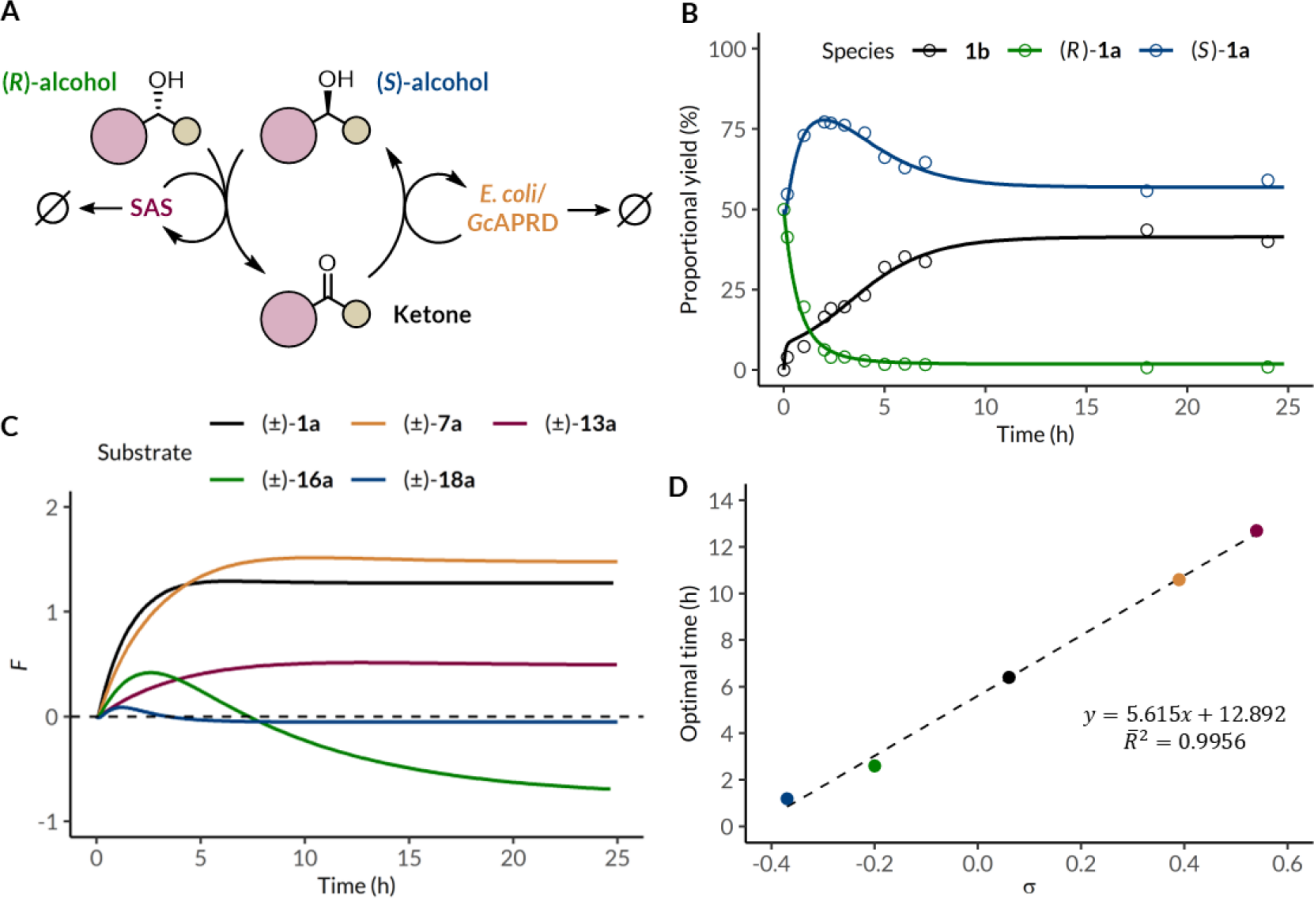
Kinetic modeling of the deracemization reaction. Schematic of the model, where slashed circles (Ø) denote degradation products (**A**). Model-fitted trajectories of substrate alcohol enantiomers (**1a**) and the ketone intermediate (**1b**) are superimposed on experimentally measured proportional yields (**B**). RMSE: 0.28 ((*R*)-**1a**), 0.29 ((*S*)-**1a**), 0.40 (**1b**). Objective function fitness scores (*F*) for the deracemization of electronically diverse alcohol substrates over time (**C**). Optimal reaction time for electronically diverse alcohol substrates based on maximum *F* score against Hammett constants (σ) obtained from Hansch (**D**). *p* < 0.01. R̅^2^; adjusted *R*-squared.

Parameter values for each catalytic cycle were obtained by fitting the model to experimentally measured trajectories of the alcohol substrate enantiomers and the ketone intermediate (Figures 5B and S14). The catalyst degradation parameters were used to simulate their degradation trajectories. With a much larger first-order rate constant than that of the photocatalyst, the WCB was determined to lose proportional activity faster regardless of substrates (Table S9). The timing of biocatalyst activity loss corresponded to the decline in (*S*)-**1a** yield after 2 h, while the timing of photocatalyst activity loss corresponded to the yield of **1b** plateauing of shortly after 6 h. This is expected due to the mutual perturbation between two catalytic cycles, although the photocatalyst actively contributes to biocatalyst degradation via ROS generation while the biocatalyst only decreases photocatalyst activity by scattering photon input.^17,78^

To guide further optimization using the model, we developed a simple objective function accounting for the overall effectiveness of deracemization at any time point throughout the reaction, where the fitness score (*F*) is increasingly sensitive to changes in er approaching enantiopurity, and linearly correlated to proportional alcohol yield, reflecting the emphasis on enantioenrichment (equation S12). *F* is defined as zero for the initial state of the system containing a racemic alcohol and no ketone. Simulating the effect of adjusting each parameter individually revealed various strategies to optimize this protocol (Figure S15). Increasing photocatalyst excitation rate or effective reactivity improves the rate and effectiveness of deracemization of a given substrate, whereas the equivalent changes in the biocatalyst lead to similar but much less pronounced effects on *F*, indicating the photocatalytic cycle to be rate-limiting with respect to the overall reaction. As expected, reducing the degradation rates of each catalyst leads to increases in maximum *F* over the course of the reaction. Intriguingly, improving photocatalyst stability results in *F* suffering at time scales beyond the substrate-specific optimal reaction time, while improving biocatalyst stability provides a less time-sensitive approach to optimization.

To ensure the general applicability of this protocol, it is also important to consider the effects of the electronic properties of the substrates on the model parameters. We obtained model parameters and calculated *F* scores for five electronically diverse substrates ((±)-**1a**, (±)-**7a**, (±)-**13a**, (±)-**16a**, and (±)-**18a**) (Figure 5C and Table S9). The optimal reaction time for each substrate based on our objective function has a strong, linear correlation to the corresponding Hammett constants (Figure 5D). This enables the prediction of optimal reaction times for any substrate with a known Hammett constant and potentially other relevant constants describing electronic properties, as long as it has a strong correlation to the observed reactivity under standard conditions. Meanwhile, the maximum *F* scores for each substrate across the reaction time scale corroborate the observed trend that this protocol is the most effective for electronically neutral and mildly electron-deficient substrates, regardless of reaction time, due to the inherently opposing thermodynamic preferences of the photo-oxidation and bioreduction processes (Figure S16).

## Conclusion

By combining the unique strengths of photocatalysis and biocatalysis while mitigating their incompatibilities by spatial compartmentalization using whole cells, we developed a one-step protocol for the concurrent linear deracemization of secondary benzylic alcohols with simultaneous photo- and biocatalytic cycles. The reported protocol presents a versatile strategy that can likely be applied to many combinations of photo- and biocatalysts as lyophilized whole cells, including (*R*)-specific ADHs that provide access to the opposite enantiomer synthesized in this work given the identification of a suitable biocatalyst. However, the scope of this deracemization protocol is inherently limited since the alcohol substrates and the corresponding ketone intermediates must undergo catalytic cycles with opposing preferences for electronic properties. While a minor contributor for most of the substrates investigated in this study, the outliers to the observed trends could be explained by steric effects and interactions in the enzyme binding pocket as revealed by molecular docking. Additionally, we identified several bulky, nonpolar residues in the large pocket with limited conservation that may control substrate specificity, which are potential targets for protein engineering to accommodate a broader substrate scope.

Furthermore, the introduction of whole cells complicates optimization due to the addition of variables in terms of biocatalyst activity and stability, making the mutual perturbations between the catalytic cycles inextricable.^52^ We addressed this challenge with kinetic modeling, enabling the direct extraction of parameters which describe the isolated properties of each catalyst within the multicomponent reaction. Additionally, we observed a quantitative relationship between substrate electronic properties and deracemization effectiveness over time. This information gave rise to a predictive model for reaction time optimization using only a small data set of five substrates. In conclusion, we demonstrated the power of whole-cell photobiocatalysis combined with a simple, computationally inexpensive modeling framework for dynamic reaction networks. Future work will focus on the outlined optimization strategies with a focus on improving photocatalyst activity and biocatalyst stability.

## Materials and Methods

All information pertaining to the materials and methods used in this study is reported in the Supporting Information.

## Supplementary Material



## Data Availability

The research data underpinning this publication can be accessed at 10.17630/b6ccc7a3-9657-4cc3-80fb-23c1f764d95c.

## Notes

Additional experimental details, materials, methods, NMR spectra for all compounds, HPLC traces for chiral compounds, and details of the theoretical studies (PDF)

## Abbreviations

ROS: reactive oxygen species
WCB: whole-cell biocatalyst
UV: ultraviolet
ADH: alcohol dehydrogenase
er: enantiomeric ratio
SAS: sodium anthraquinone-2-sulfonate
DMSO: dimethyl sulfoxide
LED: light-emitting diode
CFL: compact fluorescent lamp
PBS: phosphate-buffered saline
UV–vis spectroscopy: ultraviolet–visible spectroscopy
EtOH: ethanol
HPLC: high performance liquid chromatography
NAD­(P)­H: reduced nicotinamide adenine dinucleotide (phosphate)
TTN: total turnover number
TOF: turnover frequency
SOD: superoxide dismutase
SDS-PAGE: sodium dodecyl sulfate polyacrylamide gel electrophoresis
PCET: proton-coupled electron transfer
HAT: hydrogen atom transfer
ee: enantiomeric excess
Rib: ribose
ADP: adenosine diphosphate.
