# Supplementary material for: Concurrent Linear Deracemization of Secondary Benzylic Alcohols via Simultaneous Photocatalysis and Whole-cell Biocatalysis
Source: ACS Catal. 2025 Aug 18;15(17):15195–210. doi: 10.1021/acscatal.5c04974 (PMC12418310; doi:10.1021/acscatal.5c04974)
Supplement: Supplementary file 1 [file cs5c04974_si_001.pdf]

Concurrent Linear Deracemization of Secondary Benzylic Alcohols via Simultaneous Photocatalysis and Whole-cell Biocatalysis

Wang Yui Wylan Wong,[1] Stephen Wallace,[2*] Craig P. Johnston[1*]

[1] EaStCHEM, School of Chemistry, University of St Andrews, St Andrews, Fife, KY16 9ST, United Kingdom

[2] Institute of Quantitative Biology, Biochemistry and Biotechnology, School of Biological Sciences, University of Edinburgh, Edinburgh, EH9 3FF, United Kingdom

*Correspondence to:

Stephen Wallace: stephen.wallace@ed.ac.uk

Craig P. Johnston: cpj3@st-andrews.ac.uk

Supporting Information

Supporting Information

Concurrent Linear Deracemization of Secondary Benzylic Alcohols via Simultaneous Photocatalysis and Whole-cell Biocatalysis

Table of Contents

1 General information ... 3

1.1 Preparation of solvents and sterile solutions ... 3

1.2 Molecular cloning ... 3

1.3 Experimental Details ... 4

1.4 Purification of Products ... 4

1.5 Analysis of Products ... 4

2 General procedures for chemical transformations ... 6

2.1 General procedure A ... 6

2.2 General procedure B ... 6

2.3 General procedure C ... 7

2.4 General procedure D ... 7

2.5 General procedure E ... 8

3 Preparation of recombinant biocatalysts ... 9

3.1 Custom ribosome binding site design ... 9

3.2 Construction of recombinant expression plasmids ... 9

3.3 Plasmid transformation into chemically competent *E. coli* cells ... 14

3.4 Preparation of whole-cell biocatalysts (WCBs) ... 14

3.5 Verification of protein expression ... 14

4 Photocatalytic oxidation of secondary benzylic alcohols ... 16

4.1 Mechanistic investigation ... 16

4.2 Side product formation ... 19

5 Biocatalytic reduction of secondary benzylic ketones ... 20

5.1 Enzyme degradation measurements ... 20

5.2 Molecular docking ... 21

5.3 Bioinformatic analysis ... 29

6 Concurrent linear deracemization ... 30

6.1 Reaction optimization ... 30

6.2 Deracemization with whole-cell suspension ... 35

6.3 Viability assay for WCBs ... 36

7 Kinetic model ... 37

7.1 Derivation of a kinetic model for concurrent linear deracemization ... 37

7.2 Experimental measurements of parameters in a standard system ... 41

7.3 Model fitting to measured reaction trajectories ... 43

7.4 Model-guided reaction optimization ... 47

8 Characterization of reference compounds and deracemization products ... 49

9 References ... 76

10 NMR Spectral data ... 81

11 Chiral HPLC traces ... 130

1 General information

All reagents and solvents were obtained from commercial suppliers and were used without further purification unless otherwise stated. The desired products were purchased to serve as NMR and HPLC reference compounds for product formation. For products that were not commercially available, the reference compounds were synthesized as described in **general procedure A**. For all photochemical procedures, EvoluChem LEDs (HepatoChem) were used in EvoluChem PhotoRedOx Boxes (HepatoChem) at RT following manufacturer instructions.

1.1 Preparation of solvents and sterile solutions

Solvents used for purification purposes (hexane, pentane, EtOAc, CH_2Cl_2) were used as received from suppliers without further purification. Hexane is defined as *n*-hexane, pentane is defined as *i*-pentane, and petrol is defined as petroleum ether 40 – 60 °C. Sterile solutions were obtained either by autoclaving at 120 °C for 15 min or passing through sterile cellulose acetate filters (0.20 µm). Culture media were prepared according to the literature, namely LB[1], TB[2], M9, and M9CA.[3] M9 refers to the M9 minimal salts with glucose (2.0% w/v) in deionized H_2O (dH_2O). M9CA refers to casamino acids (0.2% w/v) added to M9.

1.2 Molecular cloning

All synthetic genes were codon-optimized for *E. coli* BL21(DE3) and synthesized by Integrated DNA Technologies. Oligonucleotide primers were synthesized by Integrated DNA Technologies or Thermo Fisher Scientific. All PCRs were performed using Q5® High-Fidelity 2X Master Mix (NEB) following manufacturer recommendations for 30 cycles with the annealing temperature descending by 0.5 °C per cycle, starting from 72 °C. Products from PCR were purified from agarose gel with a Peqlab peqGOLD Gel Extraction Kit (VWR Chemicals). All restriction enzymes were purchased from Thermo Fisher Scientific and used following manufacturer recommendations. Plasmid DNA was purified with a Peqlab peqGOLD Plasmid Miniprep Kit I (VWR Chemicals) from *E. coli* DH5α. All plasmids were constructed in *E. coli* DH5α using NEBuilder® HiFi DNA Assembly Master Mix (NEB) or GeneArt™ Gibson Assembly HiFi Master Mix (Thermo Fisher Scientific) and confirmed by Sanger sequencing (Eurofins Genomics).

1.3 Experimental Details

All reactions were performed in aqueous solvent under an ambient atmosphere unless stated otherwise. Room temperature (RT) refers to 20 – 30 °C. Reactions that require heating were performed using DrySyn blocks (Asynt) and a contact thermocouple. 'Concentrated *in vacuo*' refers to the use of an IKA RV8 with an IKA HB digital heating bath and Vacuubrand MD1C vacuum controller. Rotary evaporator condensers were fitted to a recirculating chiller (Julabo F500) filled with either a water/ethylene glycol mix (1:1) or Julabo Thermal G cooling fluid set to 0 °C. 'Dried *in vacuo*' refers to the use of a Schlenk line and high vacuum pump (Edwards RV8).

1.4 Purification of Products

Analytical thin layer chromatography (TLC) was performed using pre-coated aluminium backed plates (Merck TLC Silica 60 F_{254}). Visualization was achieved under UV light (λ = 254 nm) and/or with staining with aqueous $KMnO_4$ solution, ethanolic vanillin solution, or ethanolic phosphomolybdic acid (PMA) solution followed by gentle heating. Flash chromatography was performed using compressed air (hand bellows or airline) in glass columns containing porosity 2/3 sintered disks over silica gel (60 Å pore size, 40 – 63 µm particle size), using the solvent system stated.

1.5 Analysis of Products

Compound names have been generated using ChemDraw® Professional (PerkinElmer) software. The numbering system for compounds does not correspond to the IUPAC names but was chosen to allow for the simplistic and consistent assignment of spectra.

Optical Rotations $[\alpha]_D^{20}$ were measured on a Perkin Elmer Precisely/Model-341 polarimeter operating at the sodium D line with a 100 mm path cell at 20 °C. Concentration (*c*) is reported in g/100 mL. Temperatures are reported in °C.

High Performance Liquid Chromatography (HPLC) was performed on an Agilent 1260 Infinity II LC system consisting of a 1260 Infinity II Quaternary Pump, 1260 Infinity II Vial Sampler, 1260 Infinity II Multicolumn Thermostat, and a 1260 Infinity II Diode Array Detector WR. Separation was achieved using either Daicel CHIRALPAK® AD-H, AS-H, IA, IB, IC, or

IG columns or Daicel CHIRALCEL® OJ-H or OD-H columns using the method stated. All columns (4.6 mm ø x 250 mm, 5 µm particle size) were used with a corresponding guard column (4 mm ø x 10 mm, 5 µm particle size). HPLC traces of enantiomerically enriched compounds were compared with authentic racemic samples. Wavelengths (λ) are reported in nm, retention times (t_R) are reported in minutes, temperatures are reported in °C, and solvent flow rates are reported in mL/min.

Nuclear Magnetic Resonance (NMR) spectra (^{1}H, ^{13}C{^{1}H}, and ^{19}F{^{1}H}) were acquired on either a Bruker AV300 with a BBFO probe (^{1}H 300 MHz; ^{13}C{^{1}H} 75 MHz; ^{19}F{^{1}H} 282 MHz), a Bruker AV400 with a BBFO probe (^{1}H 400 MHz; ^{13}C{^{1}H} 101 MHz; ^{19}F{^{1}H} 377 MHz), a Bruker AVII 400 with a BBFO probe (^{1}H 400 MHz; ^{13}C{^{1}H} 101 MHz; ^{19}F{^{1}H} 376 MHz), a Bruker AVIII-HD 500 with a SmartProbe BBFO+ probe (^{1}H 500 MHz, ^{13}C{^{1}H} 126 MHz, ^{19}F{^{1}H} 470 MHz), or a Bruker AVIII 500 with a CryoProbe Prodigy BBO probe (^{1}H 500 MHz, ^{13}C{^{1}H} 126 MHz, ^{19}F 471 MHz). All NMR spectra were recorded at 25 °C unless otherwise stated with the stated deuterated solvent used as a lock for spectra and internal reference ($CDCl_3$: ^{1}H, 7.26 ppm; ^{13}C, 77.2 ppm; DMSO-d_6: 1H 2.50 ppm; ^{13}C 39.5 ppm. For ^{19}F{^{1}H} NMR, spectra were externally referenced to $CFCl_3$ (0.00 ppm). All chemical shifts are quoted in parts per million (ppm) relative to the residual solvent peak. All coupling constants, *J*, are quoted in Hz. Multiplicities are indicated as s (singlet), d (doublet), t (triplet), q (quartet), m (multiplet), and multiples thereof. The abbreviation Ar denotes aryl and app. denotes apparent. NMR peak assignments were confirmed using 2D ^{1}H correlated spectroscopy (COSY), 2D ^{1}H–^{13}C heteronuclear multiple-bond correlation spectroscopy (HMBC), and 2D ^{1}H–^{13}C heteronuclear single quantum coherence (HSQC) where necessary.

UV-visible spectra and optical density were measured with FLUOstar Omega Microplate Reader (BMG Labtech), Infinite M200 PRO plate reader (Tecan), or Implen NanoPhotometer NP80-Touch UV/Vis Spectrophotometer (Geneflow). Nucleic acid and protein concentration were measured with Implen NanoPhotometer NP80-Touch UV/Vis Spectrophotometer (Geneflow).

2 General procedures for chemical transformations

2.1 General procedure A

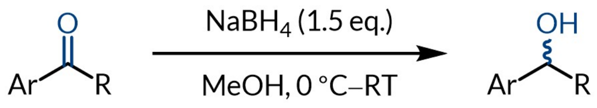

$NaBH_4$ (1.5 eq.), MeOH (1.0 M), and benzylic ketone (1.0 eq.) were added into a round-bottom flask at 0 °C. The mixture was stirred at RT until completion as determined by TLC or up to 24 h, followed by the addition of dH_2O (10.0 mL). The reaction mixture was extracted with EtOAc (3 × 10 mL) unless otherwise stated. The organic phase was separated, washed with brine (1 mL), dried over Na_2SO_4, filtered, and concentrated *in vacuo*.

2.2 General procedure B

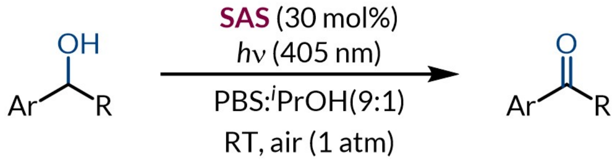

SAS (30 mol% from 8 mM stock in PBS) and benzylic alcohol (1 eq.) were added into 1X PBS (pH 7.4, 500 µL, 20 mM) containing 10% (v/v) ^{i}PrOH. The mixture was stirred and irradiated by Evoluchem blue LED (405 nm, HepatoChem) in EvoluChem PhotoRedOx Boxes (HepatoChem) at RT for a specified time for each experiment.

For yield determination by $^{19}F\{^1H\}$ NMR spectroscopy, the reaction mixture was homogenized by the direct addition of DMSO-d_6 with TFE (0.33 eq.) as internal standard, vigorous shaking with a vortexer (3 min, 3,000 RPM), followed by centrifugation (3 min, 15,000 × *g*) to obtain clarified supernatant.

For yield determination by ^1H NMR spectroscopy, the reaction mixture was transferred to a 2 mL microcentrifuge tube, brine (500 µL) was added, and then the mixture was extracted with $CHCl_3$ (3 × 1 mL) by vigorous shaking with a vortexer (3 min, 3,000 RPM). The organic phase was separated by centrifugation (3 min, 15,000 × *g*), dried over Na_2SO_4, filtered, and concentrated *in vacuo*. *p*-Xylene (0.25 eq.) was used as an internal standard.

2.3 General procedure C

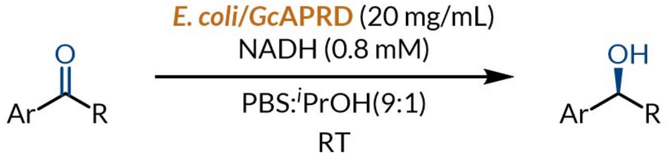

E. coli/*Gc*APRD (20 mg/mL), NADH (0.8 mM), and benzylic ketone (1.0 eq.) were added into 1X PBS (pH 7.4, 500 µL, 20 mM) containing 10% (v/v) ^{i}PrOH in a 4 mL glass vial. The mixture was stirred at 750 RPM at RT for a specified time for each experiment.

For yield determination by ^{19}F{^{1}H} NMR spectroscopy, the reaction mixture was transferred to a 1.5 mL microcentrifuge tube, mixed with 6 M HCl (10 µL) followed by saturated $NaHCO_3$ (75 µL) for neutralization, homogenized by the direct addition of DMSO-d_6 with TFE (0.33 eq.) as internal standard, vigorous shaking with a vortexer (3 min, 3,000 RPM), followed by centrifugation (3 min, 15,000 × *g*) to obtain clarified supernatant.

For yield determination by ^{1}H NMR spectroscopy, the reaction mixture was transferred to a 2 mL microcentrifuge tube, mixed with 6 M HCl (10 µL) followed by saturated $NaHCO_3$ (75 µL) for neutralization, brine (500 µL) was added, and then the mixture was extracted with $CHCl_3$ (3 × 1 mL) by vigorous shaking with a vortexer (3 min, 3,000 RPM). The organic phase was separated by centrifugation (3 min, 15,000 × *g*), dried over Na_2SO_4, filtered, and concentrated *in vacuo*. *p*-Xylene (0.25 eq.) was used as an internal standard.

2.4 General procedure D

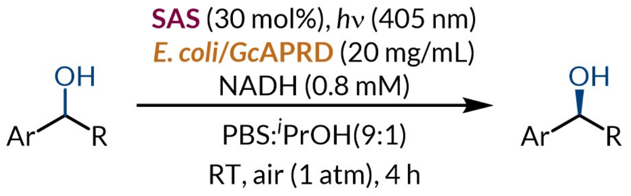

SAS (30 mol% from 8 mM stock in PBS), *E. coli*/*Gc*APRD (20 mg/mL, estimated equivalent to OD_{600} of 29.5), NADH (0.8 mM), and racemic benzylic alcohol (1 eq.) were added into 1X PBS (pH 7.4, 500 µL, 20 mM) containing 10% (v/v) ^{i}PrOH in a 4 mL glass vial. The mixture was stirred and irradiated by Evoluchem blue LED (405 nm, HepatoChem) in EvoluChem PhotoRedOx Boxes (HepatoChem) at RT for 4 h, then 6 M HCl (10 µL) was added to precipitate biological materials, followed by saturated $NaHCO_3$ (75 µL) for neutralization.

For yield determination by $^{19}F\{^1H\}$ NMR spectroscopy, the reaction mixture was homogenized by the direct addition of DMSO-d_6 with TFE (0.33 eq.) as internal standard, vigorous shaking with a vortexer (3 min, 3,000 RPM), followed by centrifugation (3 min, 15,000 × *g*) to obtain clarified supernatant.

For yield determination by ^1H NMR spectroscopy, the reaction mixture was transferred to a 2 mL microcentrifuge tube, brine (500 µL) was added, and then the mixture was extracted with $CHCl_3$ (3 × 1 mL) by vigorous shaking with a vortexer (3 min, 3,000 RPM). The organic phase was separated by centrifugation (3 min, 15,000 × *g*), dried over Na_2SO_4, filtered, and concentrated *in vacuo*. *p*-Xylene (0.25 eq.) was used as an internal standard.

2.5 General procedure E

For enantiomeric ratio determination by HPLC analysis of samples dissolved in DMSO-d_6, the samples were extracted with Et_2O (750 µL) by vigorous shaking with a vortexer (3 min, 3,000 RPM). The organic phase was separated by centrifugation (1 min, 22,000 × *g*), then passed through a silica plug and eluted with Et_2O (10 mL). If required, the silica plug was further eluted with acetone (4 mL).

For enantiomeric ratio determination by HPLC analysis of samples dissolved in $CDCl_3$, the samples were passed through a silica plug and eluted with Et_2O (10 mL). If required, the silica plug was further eluted with acetone (4 mL).

HPLC analysis was performed with appropriate chiral stationary phases and settings.

3 Preparation of recombinant biocatalysts

3.1 Custom ribosome binding site design

A custom ribosome binding site (RBS), RBS1, was designed using De Novo DNA using free energy model version 2.1 with a predicted strength of 9628.70 (a. u.).[4]

3.2 Construction of recombinant expression plasmids

Table S1. List of plasmids used in this study.

Plasmid name	Description	Reference
pET-22b(+)	pET-22b(+) vector (pBR322/Rop oriV, AmpR, *lacI*, T7 IPTG induction, pelB signal)	Novagen
pADH-*Rr*ADHA	pET-22b(+) vector, RBS1, CDS of *Rhodococcus ruber* alcohol dehydrogenase A (*Rr*ADHA) with C-terminal 6×His tag fusion, pelB signal deletion	This work
pADH-*Gc*APRD	pET-22b(+) vector, RBS1, CDS of *Geotrichum candidum* acetophenone reductase (*Gc*APRD) with C-terminal 6×His tag fusion, pelB signal deletion	This work
pADH-*Lk*ADH	pET-22b(+) vector, RBS1, CDS of *Lactobacillus kefir* alcohol dehydrogenase (*Lk*ADH) with C-terminal 6×His tag fusion, pelB signal deletion	This work
pADH-*Kt*CR	pET-22b(+) vector, RBS1, CDS of *Kluyveromyces thermotolerans* carbonyl reductase (*Kt*CR) with C-terminal 6×His tag fusion, pelB signal deletion	This work
pADH-*Eb*SDR8	pET-22b(+) vector, RBS1, CDS of *Empedobacter brevis* short chain dehydrogenase/reductase (*Eb*SDR8) with C-terminal 6×His tag fusion, pelB signal deletion	This work
pADH-*Lx*CAR-S154Y	pET-22b(+) vector, RBS1, CDS of *Leifsonia xyli* carbonyl reductase (*Lx*CAR, S154Y mutant) with C-terminal 6×His tag fusion, pelB signal deletion	This work

Table S2. Oligonucleotide primers used for plasmid construction.

Plasmid	Template	Sense	Sequence
pADH-*Rr*ADHA	*Rr*ADHA CDS	F	GTTTAACTTTAAGAAGGAGATATACAATGAAAGCTGTTCAATACACGGAGATC
		R	CGGAGCTCGAATTCGCGGATCCTGGAACAACAACCCCACGGC
pADH-*Gc*APRD	*Gc*APRD CDS	F	GTTTAACTTTAAGAAGGAGATATACAATGGGCAAGGTCCCTGAGAC
		R	CGGAGCTCGAATTCGCGGATCCGATATCATCAAAGTCTGTCAGCACAAGG
pADH-*Lk*ADH	*Lk*ADH CDS	F	GTTTAACTTTAAGAAGGAGATATACAATGACGGACCGTCTCAAGG
		R	CGGAGCTCGAATTCGGGGATCCTTGCGCGGTATAACCGCC
pADH-*Kt*CR	*Kt*CR CDS	F	AATAATTTTGTTTAACTTTAAGAAGGAGATATACAATGCCCAAAACAATAGCTACTGGAC
		R	GAGTGCGGCCGCAAGCTTGATACTGCTGTAGCCACCGT
pADH- *Eb*SDR8	*Eb*SDR8 CDS	F	AAATAATTTTGTTTAACTTTAAGAAGGAGATATACAATGTCAATTCTAAAGGATAAAGTAGCTATAGTTACGG
		R	GAGTGCGGCCGCAAGCTTGACAGCGGTGTAGCCACC
pADH-*Lx*CAR-S154Y	*Lx*CAR-S154Y CDS	F	ATAATTTTGTTTAACTTTAAGAAGGAGATATACAATGGCACAATATGATGTAGCTGGAAG
		R	GAGTGCGGCCGCAAGCTTTTGCGCGGTGTAGCCAC

All plasmids listed in Table S1 were constructed by HiFi assembly with PCR-amplified, codon-optimized synthetic genes (*vide infra*) using primers listed in Table S2. The nucleotide sequences (5′ to 3′) of the resulting operons are listed and annotated as follows: promoter, operator, **RBS**, CDS, *terminator*.

pADH-*Rr*ADHA

TAATACGACTCACTATAGGGGAATTGTGAGCGGATAACAATTCCCCTCTAGAAATAAT**TTTGTTTAAC**
TTTAAGAAGGAGATATACAATGAAAGCTGTTCAATACACGGAGATCGGGTCAGAACCTGTGGTGGTTG
ATATTCCTACTCCTACACCTGGTCCGGGTGAGATTCTCTTAAAGGTTACAGCCGCAGGGTTATGTCAT
AGTGACATTTTCGTAATGGACATGCCGGCTGCTCAGTACGCGTACGGTCTCCCGTTGACCTTGGGCCA
TGAGGGCGTGGGTACCGTTGCCGAACTTGGTGAGGGTGTAACGGGTTTCGGGGTTGGTGATGCCGTAG
CCGTGTATGGCCCGTGGGGTTGCGGTGCGTGCCATGCCTGTGCTCGGGGTCGGGAGAATTACTGTACG
CGTGCTGCTGATTTAGGTATCACACCTCCTGGGCTCGGCAGTCCGGGTTCTATGGCTGAATATATGAT
CGTAGACTCAGCGCGTCACCTTGTACCGATCGGGGACCTGGACCCAGTGGCAGCAGCCCCATTAACGG
ACGCTGGTTTAACGCCGTACCATGCAATCTCGCGTGTACTCCCATTGTTGGGCCCTGGCTCAACCGCC
GTAGTGATTGGCGTAGGCGGTCTTGGGCATGTTGGGATTCAAATTTTGCGGGCCGTATCGGCTGCCCG
GGTGATCGCAGTTGATTTGGACGATGACCGGTTAGCATTGGCGCGCGAGGTAGGCGCGGATGCCGCAG
TCAAATCTGGCGCTGGGGCAGCTGACGCCATTCGTGAACTGACGGGGGGGCAGGGCGCAACAGCGGTC
TTCGACTTTGTAGGGGCGCAGTCCACAATTGACACCGCCCAACAGGTCGTAGCAGTCGATGGCCATAT
CAGCGTAGTTGGCATTCACGCCGGTGCACACGCAAAGGTGGGGTTTTTCATGATCCCTTTTGGCGCAT
CAGTGGTAACTCCTTATTGGGGTACCCGGTCCGAGCTGATGGAAGTAGTCGCGTTAGCACGCGCAGGT
CGCCTGGACATCCACACTGAAACGTTCACATTAGATGAAGGCCCTGCAGCCTACCGCCGTCTTCGGGA
AGGTTCGATTCGGGGCCGTGGGGTTGTTGTTCCAGGATCCGCGAATTCGAGCTCCGTCGACAAGCTTG
CGGCCGCACTCGAGCACCACCACCACCACCACTGAGATCCGGCTGCTAACAAAGCCCGAAAGGAAGCT
GAGTTGGCTGCTGCCACCGCTGAGCAATAA*CTAGCATAACCCCTTGGGGCCTCTAAACGGGTCTTGAG*
GGGTTTTTTG

pADH-*Gc*APRD

TAATACGACTCACTATAGGGGAATTGTGAGCGGATAACAATTCCCCTCTAGAAATAAT**TTTGTTTAAC**
TTTAAGAAGGAGATATACAATGGGCAAGGTCCCTGAGACTCATAAGGGTTATGTCTTTACTTCGGGTT
CGTCCCGGTTGACACTCAAGGATGTGCCGACATACAAACCGGGGCCAGGGGAGGTCCTGTTAAAATTA
GAGGCATCAGGCGTGTGCCACTCCGATTTGCATATTCTGCAAGGCTCCTTCCCAATTCCATCCAACTC
AGTGCTTGGGCACGAGATTACAGGCACCGTGGTGGCGTACGGGTTAGGTGTGGACCCAAAGACGTACC
CTGAGGGGCAACTTTATGCAGCCCACGGGCCGAACCCTTGCGGTTCCTGTCGTGAATGCCGCAGCGGG
AAAGATAACCTGTGCCATGCTGAAAACCGTACCAACTACGGGCTGGGTTACCCAGGGGGCTATCAGCA
ATATACGCTGGCCAAGGTGCATAATCTCATCAAGGTTCCAGATGGCGTCGGCGCTGCCATTGCGGCAG
TGACGACAGACGCTGTTCTCACGCCTTACCATGCCTTCAAGAAGGCTGACATTAACGGTTTAAGCAAA
ATTCTCATTATCGGGCTCGGTGGCCTCGGGATCAATGCAGTTCAGATTGCTAAAGCCATGGGCGCGCA
CGTCACGGCGTATGACCTGAAGGAGTCGAGCCGCCAACTTGCTCGTCAATTCGGCGCAGACGTTGTAC
TCGAAAGTCTCACCCTCGACGATGCGTCAAAAGAGTATGACTTTGTGGCAGACATCGTTAGTATCCAG
TCCACGTTTGATTTAGCCCTCAAGCAGGTCAAGTCTAATGGGTTGGTAATTCCACTGGGCTTGGGCAG
CCCAAAACTTACTTTCGACCAAAATGACCTCCTGGTCCGCGAAATTCGTATCTTGGGCAGCTTTTGGG
GGACATCGTTAGACCAAGCAGAAGTATTTGACTTGGTAAAATCTGGGGCGTTTAAGCCACAGGTAGAA
ACGGGCAAGTTCAAAGATCTTAATGAAATCCTCGAAAAGTTGGAAAAGGGGCAGATTAAGAGCCGCCT
TGTGCTGACAGACTTTGATGATATCGGATCCGCGAATTCGAGCTCCGTCGACAAGCTTGCGGCCGCAC
TCGAGCACCACCACCACCACCACTGAGATCCGGCTGCTAACAAAGCCCGAAAGGAAGCTGAGTTGGCT
GCTGCCACCGCTGAGCAATAA*CTAGCATAACCCCTTGGGGCCTCTAAACGGGTCTTGAGGGGTTTTTT*
G

pADH-*Lk*ADH

TAATACGACTCACTATAGGGGAATTGTGAGCGGATAACAATTCCCCTCTAGAAATAAT**TTTGTTTAAC**
TTTAAGAAGGAGATATACAATGACGGACCGTCTCAAGGGGAAGGTAGCCATTGTAACTGGGGGTACCT
TAGGTATCGGCCTCGCTATCGCCGACAAGTTTGTTGAAGAGGGCGCTAAGGTCGTTATCACAGGTCGC
CACGCTGATGTCGGTGAGAAGGCAGCGAAGAGTATCGGTGGGACGGACGTTATTCGTTTCGTGCAACA
TGATGCTAGTGATGAAGCTGGTTGGACCAAATTGTTCGATACGACCGAGGAGGCGTTCGGGCCAGTGA
CCACTGTGGTGAATAATGCCGGCATCGCGGTTTCTAAATCAGTAGAAGATACAACTACAGAAGAGTGG
CGCAAATTGTTGTCCGTGAATCTGGACGGGGTTTTCTTCGGCACCCGGTTAGGTATCCAGCGCATGAA
GAATAAAGGTCTGGGGGCGAGCATCATCAACATGAGCTCCATCGAGGGTTTTGTTGGCGACCCTACAT
TGGGCGCCTATAATGCCTCGAAAGGCGCTGTACGGATTATGTCGAAATCCGCTGCACTTGATTGTGCG
CTTAAAGACTACGATGTACGGGTGAACACAGTTCATCCAGGTTACATTAAGACTCCATTAGTCGATGA
TCTTGAGGGCGCTGAGGAAATGATGAGTCAGCGGACGAAGACCCCAATGGGTCATATCGGTGAGCCTA
ACGACATTGCGTGGATCTGCGTATACTTAGCTAGTGACGAGTCAAAGTTTGCGACGGGCGCTGAGTTC
GTGGTAGACGGCGGTTATACCGCGCAAGGATCCCCGAATTCGAGCTCCGTCGACAAGCTTGCGGCCGC
ACTCGAGCACCACCACCACCACCACTGAGATCCGGCTGCTAACAAAGCCCGAAAGGAAGCTGAGTTGG
CTGCTGCCACCGCTGAGCAATAA*CTAGCATAACCCCTTGGGGCCTCTAAACGGGTCTTGAGGGGTTTT*
TTG

pADH-*Kt*CR

TAATACGACTCACTATAGGGGAATTGTGAGCGGATAACAATTCCCCTCTAGAAATAAT**TTTGTTTAAC**
TTTAAGAAGGAGATATACAATGCCCAAAACAATAGCTACTGGACTAGAAATCCCGCAGCCGCGTCCGC
AATTGCCATCGCACGTGATGGATATGTTCAGCCTGCGTGGTAAAGTGGCGTGTATTTCCGGTGCGTCG
AGCGGTATCGGTGGCGCGGTGGCGGTGGCGTACGCACAGGCGGGCGCGGACATTGCTGTGTGGTATAA
TAGCCACGACGGCCTGATTCAGACTGCCCGTGAGTTGGCCGAGAAGTATGGTGTTCGTGCAAAGGCGT
ACAAGTGCGCGGTCAACGACGAAGAGCGCGTCCAGGCGACCATTCAACAGGTTCTGGCCGACTTCGGT
GGCAGAATTGATGTCTTTGTTGCTAATGCAGGCGTGGCTTGGGAAAAAGGTGCGTTGGTTGAAGCGCA
AGAACAGGGCACGGCGTCTCGTGAGTGGGATCGTGTTCTGCAGACCGACTTCCAGGGTGTTTATTACT
GCAGCAAATTCATCGGCGCCGTCTTTAAAAAACAAGGGTGCGGTAGCCTGGTGATCACCGCTAGCATG
AGCGGTCATGTAGTTAACGTGCCGCAACTGCAAACGTGTTATAACGCGGCAAAGGCAGGCGTCATCCA
CATGGCGCGCTCCCTCGCGGTTGAGTGGGCAGGTTTTGCTCGCGTGAACACCGTTAGCCCGGGTTACA
TTAGCACCCCGATCTCTGAATTCGCGCTGGATGATGTTAAGCAGAAATGGCTGATGCTGACCCCGCTG
GGTCGTGAGGGCTTACCGGAAGAGTTGGTTGGAGCCTACCTGTATCTGGGCTCCGATGCTTCGACCTT
TACTACCGGTACGGACATCGTGGTGGACGGTGGCTACAGCAGTATCAAGCTTGCGGCCGCACTCGAGC
ACCACCACCACCACCACTGAGATCCGGCTGCTAACAAAGCCCGAAAGGAAGCTGAGTTGGCTGCTGCC
ACCGCTGAGCAATAA*CTAGCATAACCCCTTGGGGCCTCTAAACGGGTCTTGAGGGGTTTTTTG*

pADH-*Eb*SDR8

TAATACGACTCACTATAGGGGAATTGTGAGCGGATAACAATTCCCCTCTAGAAATAAT**TTTGTTTAAC**
TTTAAGAAGGAGATATACAATGTCAATTCTAAAGGATAAAGTAGCTATAGTTACGGGTGCGAGCAGCG
GTATCGGCAAGGCGGTGGCGGAGCTGTATGCAAAGGAAGGTGCGAAAGTTGTCGTGTCAGATATCGAC
GAAGAACGTGGTAAAGAGGTGGTGGAGCAGATTAAAAAGAATGGTGGTGAAGCGATTTTCTTTAAAGC
TGATACCAGCTCCCCGGAAGAGAACGAAGCGCTGGTTAAAAAGGCCGTGGAGGTTTATGGCAAACTCG
ACATCGCGTGTAACAACGCGGGTATTGGCGGGCCAGCGGAGTTGACCGAAGATTACCCGCTGGACGGT
TGGAAAAAGGTCATCGACATCAACTTCAATGGCGTTTTTTACGGCTGCAAGTATCAACTGCAGGCAAT
GGAAAAAAACGGCGGCGGCTCGATCGTGAATATGGCATCTATCCACGGCACCGTTGCCGCTCCGATGA
GCTCCGCGTACACCTCCGCTAAGCATGGCGTGGTGGGTCTGACCAAGAACATTGGCGCCGAGTATGGT
AGCAAAAACATCCGCTGCAATGCAGTTGGTCCGGGTTACATTATGACTCCGTTGCTGTCTAATAACCT
GAGCGCGGACTACCTGGAATTACTCGTAACGAAGCACCCGATTGGTCGTCTGGGTCAACCGGAGGAGG
TGGCGGAGTTGGTTCTGTTCCTGAGTAGCGATAAAGCCAGCTTTATGACCGGCGGTTATTACTTGGTC
GATGGTGGCTACACCGCTGTCAAGCTTGCGGCCGCACTCGAGCACCACCACCACCACCACTGAGATCC
GGCTGCTAACAAAGCCCGAAAGGAAGCTGAGTTGGCTGCTGCCACCGCTGAGCAATAA*CTAGCATAAC*
CCCTTGGGGCCTCTAAACGGGTCTTGAGGGGTTTTTTG

pADH-*Lx*CAR-S154Y

TAATACGACTCACTATAGGGGAATTGTGAGCGGATAACAATTCCCCTCTAGAAATAAT**TTTGTTTAAC**
TTTAAGAAGGAGATATACAATGGCACAATATGATGTAGCTGGAAGGTCAGCAATCGTTACGGGCGGGG
GCAGCGGTATCGGCCGTGCGATCGCCTTAACGCTGGCCGCAAGCGGCGCAGCGGTCCTGGTGACCGAC
CTGAACGAGGAGAATGCGAATGCGGTGGTAGCCGAAATTAGCGCAGCTGGTGGCACCGCGCGTGCTTT
GGCTGGTGACGTCACCGATCCGGCATTCGCGGAAGCGTCTGTTGCTGCAGCGAACGAGTTGGCTCCGC
TGCGTATTGCTGTGAACAACGCGGGTATTGGTGGTGCCGCGGCTCCAGTTGGCGACTACCCGCTGGAC
AGCTGGCGTAAAGTTATTGAAGTTAACCTGAATGCGGTGTTCTATGGCATGCAAGCACAGCTGGACGC
CATCGGTGCGAACGGTGGCGGCGCGATTGTGAACATGGCATCCATCCTGGGTTCGGTGGGTTTTGCCA
ATTATAGCGCGTATGTGACTGCGAAACACGCCTTGCTGGGCCTGACGCAGAACGCGGCGCTTGAGTAT
GCGGGCAAGAACGTGCGCGTGGTCGCTGTCGGCCCGGGTTTCATCAGAACCCCGTTGGTTGCGTCAAA
TATGGATGCAGATACCTTGGCGTTCTTAGAGGGTAAGCACGCGCTGGGTCGCCTGGGTGAACCGGAAG
AGGTTGCCTCCCTGGTTGCGTTTCTGGCGTCCGATGCGGCGTCTTTTATCACCGGTAGCTACCATCTC
GTTGATGGTGGCTACACCGCGCAAAAGCTTGCGGCCGCACTCGAGCACCACCACCACCACCACTGAGA
TCCGGCTGCTAACAAAGCCCGAAAGGAAGCTGAGTTGGCTGCTGCCACCGCTGAGCAATAA*CTAGCAT*
AACCCCTTGGGGCCTCTAAACGGGTCTTGAGGGGTTTTTTG

3.3 Plasmid transformation into chemically competent *E. coli* cells

Chemically competent *E. coli* cells were prepared and transformed as described by Hsu.[5] Briefly, *E. coli* was grown to OD_{600} = 0.6 in 10 mL LB medium, then incubated on ice for 15 min. Cells were centrifuged (14,800 × *g*, 7 min, 4 °C) and gently resuspended in 4 mL ice-cold $CaCl_2$ (100 mM). After incubation on ice for 20 min, cells were centrifuged (14,800 × *g*, 7 min, 4 °C) and gently resuspended in 1 mL ice-cold $CaCl_2$ (100 mM) containing 15% glycerol (v/v), then immediately aliquoted and frozen in liquid N_2. Competent cells were stored at –80 °C.

To transform *E. coli* with exogenous DNA, competent cells were thawed on ice for 10 min before being incubated with 1–10 ng plasmid on ice for further 20 min. Next, cells were transformed via heat shock at 42 °C for 45 seconds, then incubated on ice for 2 min, before adding 2 times volume of LB medium and incubating at 37 °C for 30 min. From the resulting culture, 200 µL was plated on LB agar containing the appropriate selection marker and incubated at 37 °C overnight. For cells with ampicillin resistance, the LB agar contained 100 µg/mL ampicillin.

3.4 Preparation of whole-cell biocatalysts (WCBs)

An overnight pre-culture of *Escherichia coli* BL21(DE3) harboring a pADH expression plasmid was incubated in LB (1 L) containing ampicillin (100 µg/mL) and incubated at 37 °C until reaching OD_{600} of 0.6. Protein expression was induced by 0.4–1.0 mM IPTG for 20 h at 20 °C. Cells were harvested by centrifugation (4,500 × *g*, 15 min) and washed with PBS (3 × 50 mL). The pellet was frozen with liquid N_2 for lyophilization to obtain WCBs. For *Gc*APRD, the expression plasmid was pADH-*Gc*APRD and 1.0 mM IPTG was used for induction.

3.5 Verification of protein expression

WCB pellets or lyophilizates were resuspended in PBS (500 µL), and OD_{600} was measured. The pellets were collected by centrifugation then resuspended in BugBuster® Protein Extraction Reagent (Merck) according to manufacturer instructions, normalizing the volume by OD_{600} values. The suspensions were incubated for 2 h, total protein and soluble (supernatant) fractions were collected before and after centrifugation (22,000 × *g*, 5 min), respectively. His-tagged *Gc*APRD was purified by immobilized metal affinity chromatography using HisPur™

Ni-NTA Resin (Thermo Fisher Scientific) and eluted with imidazole (10–500 mM) in Tris HCl (50 mM, pH 8.0, 300 mM NaCl).

For SDS-PAGE, 4–20% Mini-PROTEAN TGX Stain-Free protein gels (Bio-Rad) containing a prestained Precision Plus protein standard ladder (Bio-Rad) were used to analyze samples. Gels were run in 1X Tris/Glycine/SDS (TGS) buffer (Bio-Rad) at 60 V for 5 min followed by 200 V for 30 min.

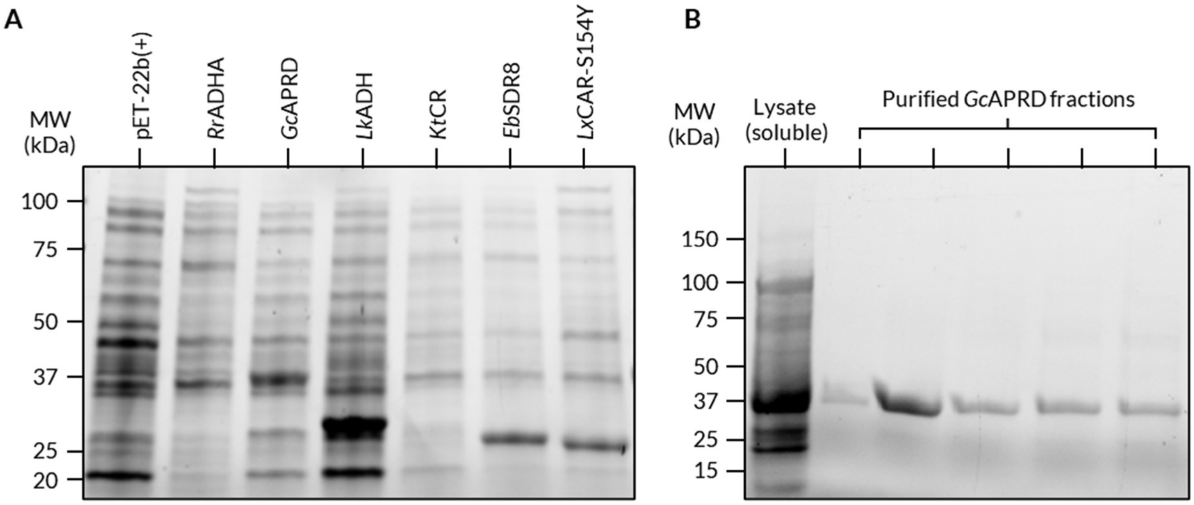

Figure S1. SDS-PAGE verification of protein expression. Lyophilized *E. coli* WCBs were lysed with BugBuster® Protein Extraction Reagent (Merck), the soluble fractions were denatured and reduced with NuPAGE LDS sample buffer (Thermo Fisher Scientific) and DTT at 95°C for 10 min, then separated on a 4–20% Mini-PROTEAN TGX Stain-Free protein gel (Bio-Rad) in 1X TGS buffer. Soluble fractions of lysates obtained from different WCBs normalized to total protein (**A**) and the purification of *Gc*APRD from the corresponding WCB (**B**) are shown. Theoretical molecular weight (MW) of 6×His-tagged enzymes including linker calculated by the ExPASy Server Compute pI/Mw tool:[6] *Rr*ADHA, 38 kDa; *Gc*APRD, 39 kDa; *Lk*ADH, 29 kDa; *Kt*CR, 32 kDa; *Eb*SDR8, 28 kDa; *Lx*CAR-S154Y, 27 kDa.

4 Photocatalytic oxidation of secondary benzylic alcohols

4.1 Mechanistic investigation

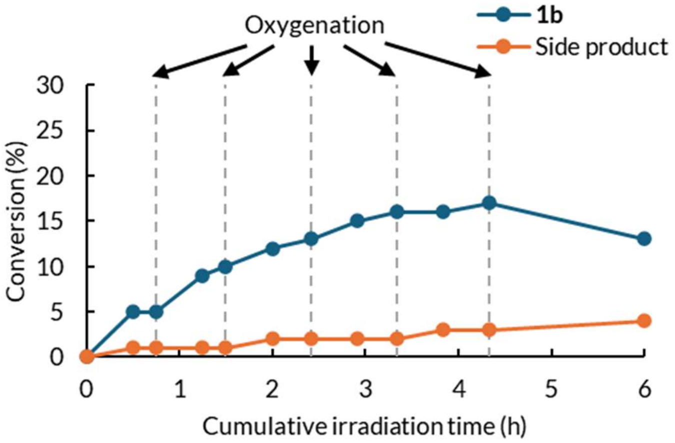

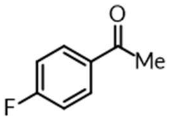

1b

Figure S2. Photocatalytic cycle interruption studies. SAS (5 mol%), (±)-**1a** (56 µmol), D_2O (70 µL), and ^{i}PrOH (70 µL) were added into 1X PBS (pH 7.4, 560 µL, 20 mM) in an NMR tube sealed with a J Young valve (GPE). The mixture was sparged with argon at 0 °C for 10 min before being irradiated by blue LED (405 nm) at RT for the specified time. At each sampling time point, conversion was determined by $^{19}F\{^{1}H\}$ NMR spectroscopy. At each time point marked by a dotted line, the mixture was cooled to 0 °C, oxygenated by bubbling with air for 5 min, then sparged with argon for 10 min before continuing irradiation.

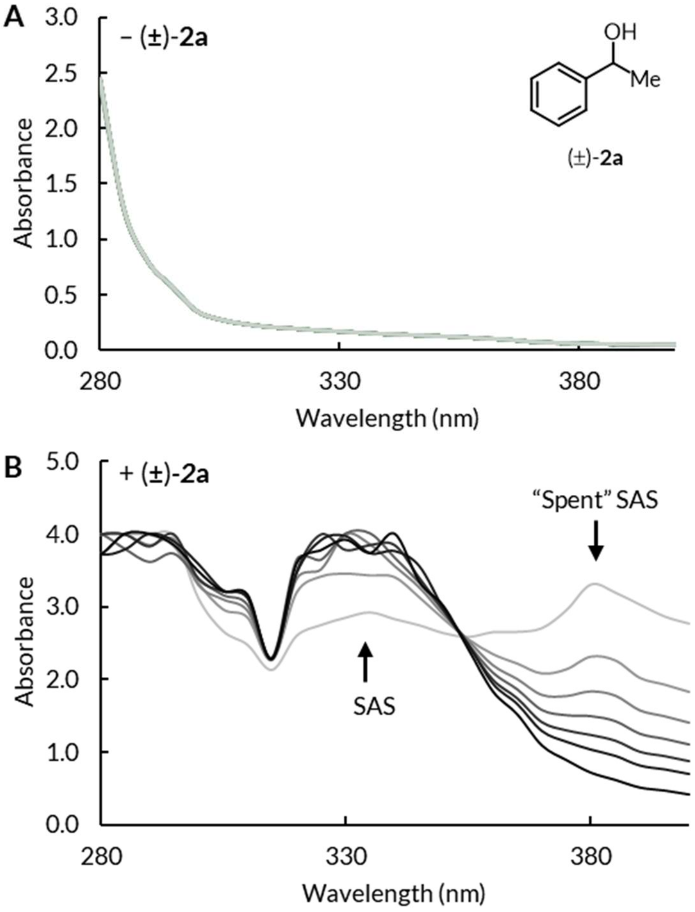

Figure S3. Aerobic photocatalyst regeneration. SAS (5 mol%) and (±)-**2a** (56 µmol, only for B) were added into 1X PBS (pH 7.4, 560 µL, 20 mM) containing 10% (v/v) ^{i}PrOH in a 4 mL glass vial. The mixture was sparged with argon at 0 °C for 10 min before being irradiated by blue LED (405 nm) at RT for 15 min before being sampled (150 µL) and exposed to air. UV-vis spectra were measured over 10.0 min at 1.0 min intervals (**A**) or 25.6 min at 0.8 min intervals (**B**) in a 96-well plate with an Infinite M200 PRO plate reader (Tecan). Increasing time of exposure to air shown by darker absorbance curves. Absorbance peaks labeled with corresponding compounds

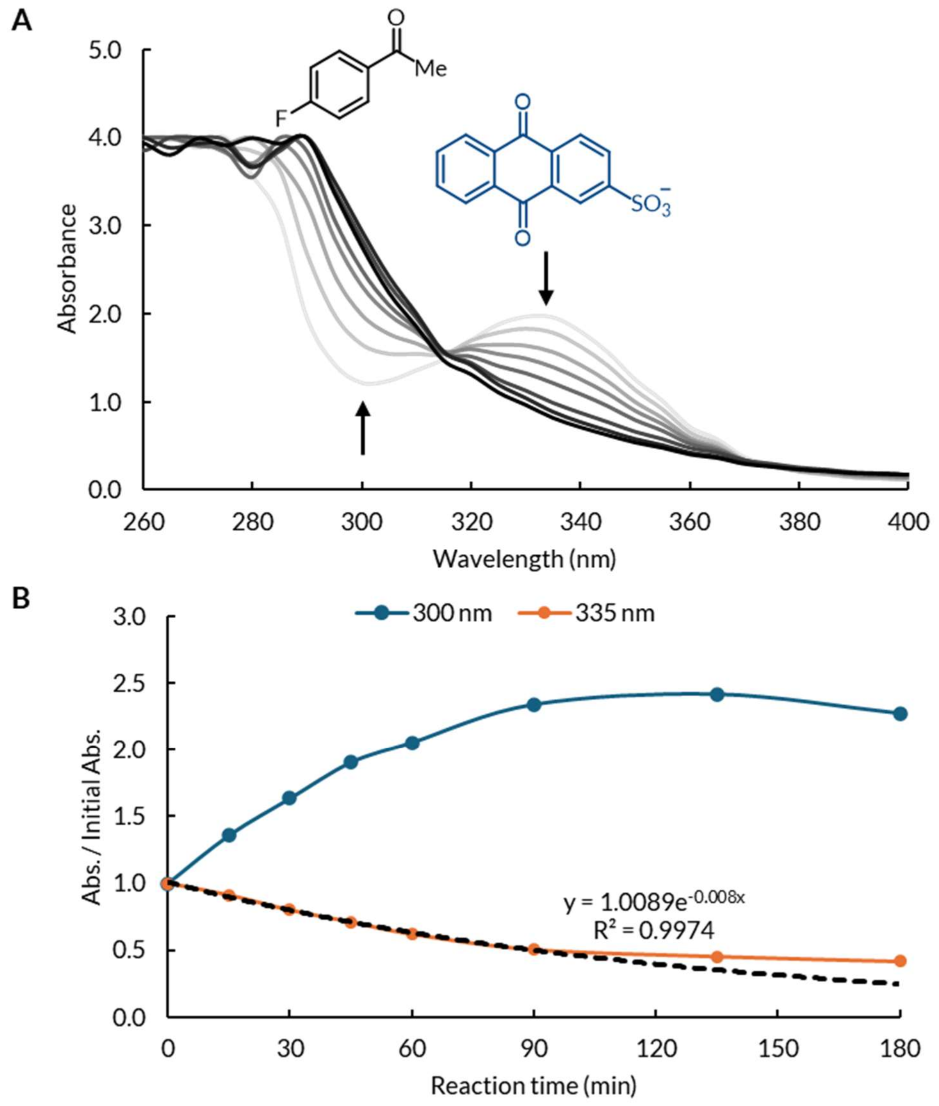

Figure S4. Kinetic profile of photocatalyst degradation. Following **general procedure B**, SAS (5 mol%) and (±)-**1a** (10 µmol) were added into 1X PBS (pH 7.4, 500 µL, 20 mM) containing 10% (v/v) ^{i}PrOH in a 4 mL glass vial. The mixture was stirred and irradiated by blue LED (405 nm) at RT for the specified time, followed by 1 h stirring in the dark. Each mixture was sampled (150 µL) and UV-vis spectra were measured in a 96-well plate with an Infinite M200 PRO plate reader (Tecan). (**A**) Increasing reaction time shown by darker absorbance curves. Absorbance peaks labeled with corresponding compounds. (**B**) Absorbance peak intensities for **1b** (300 nm) and SAS (335 nm). Least squares regression was performed on data points from the first 90 minutes to fit a first order decay curve, yielding the exponent's coefficient as an estimated rate constant of $8 \cdot 10^{-3}\ min^{-1}$.

4.2 Side product formation

A side product was observed and speculated to be *p*-fluorotoluene due to the reported formation of toluene as a photolysis product of acetophenone.[7] However, this was disproven by spiking an authentic sample of *p*-fluorotoluene into the endpoint mixture and the side product remained unidentified. Similarly, fluorobenzene, the pinacol coupling product, and overoxidation products (4-fluorobenzoic acid, 4-fluoro-2-hydroxy-acetophenone and 4-fluorophenylglyoxylic acid) were ruled out by comparing to an authentic sample or literature data.[8]

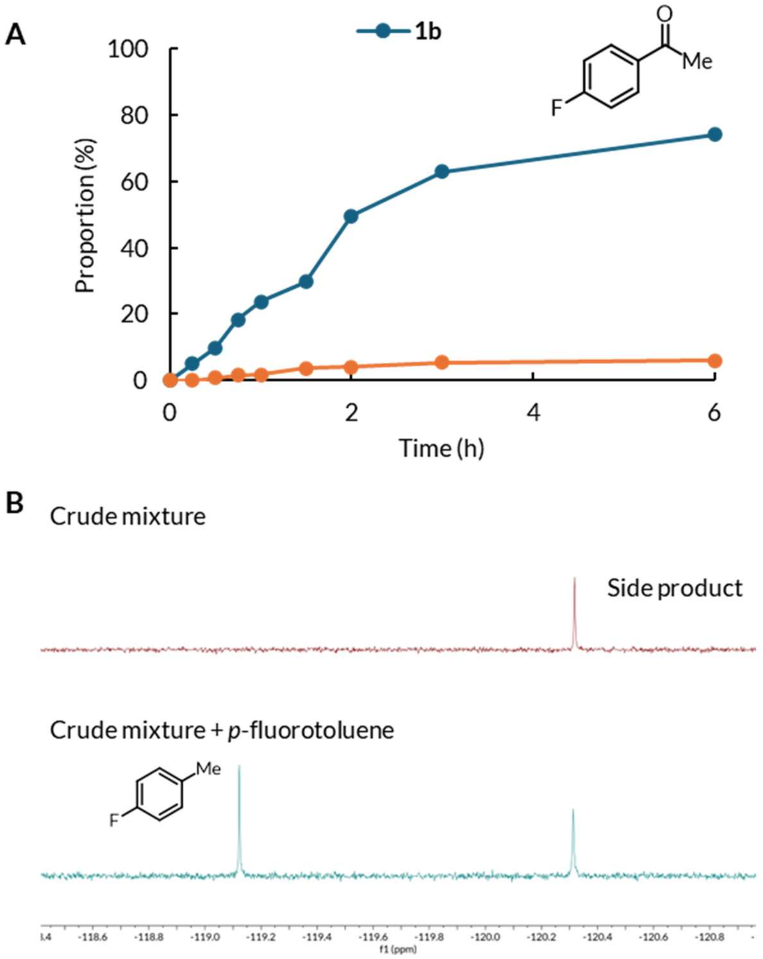

Figure S5. Kinetic profile of photo-oxidation and side product formation. Following **general procedure B**, SAS (5 mol%) and (±)-**1a** (10 µmol) were added into 1X PBS (pH 7.4, 500 µL, 20 mM) containing 15% (v/v) ^{i}PrOH in a 4 mL glass vial. The mixture was stirred and irradiated by blue LED (405 nm) at RT for the specified time. (**A**) Yields were determined by $^{19}F\{^1H\}$ NMR spectroscopy. (**B**) Partial $^{19}F\{^1H\}$ NMR spectra of the 6 h reaction mixture spiked with an authentic sample of *p*-fluorotoluene.

5 Biocatalytic reduction of secondary benzylic ketones

5.1 Enzyme degradation measurements

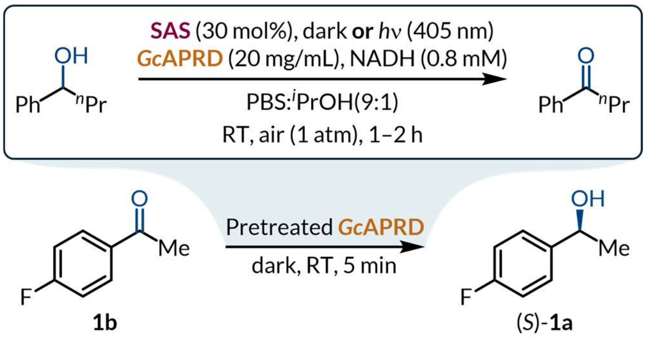

Scheme S1. Measurement of *Gc*APRD activity after treatment under reaction conditions.

For each reaction replicate, total cell lysate was prepared by resuspending 10 mg of *E. coli*/*Gc*APRD lyophilizate in 1X PBS (pH 7.4, 410 µL) containing SAS (3 µmol) in a centrifuge tube, followed by sonication on ice (Fisherbrand Model 120 Sonic Dismembrator, 108 W, 20 kHz, 10 s pulses followed by 30 s pauses for 18 cycles). The number of sonication cycles is scaled to the amount of *E. coli*/*Gc*APRD lyophilizate.

For measurements of enzyme degradation in total lysates, following **general procedure D**, NADH (0.8 mM), ^{i}PrOH (50 µL), and benzylic alcohol (±)-**26a** (40 µmol) were added into the lysate in a 4 mL glass vial. For measurements in WCB lyophilizates, following **general procedure D**, SAS (30 mol%), *E. coli*/*Gc*APRD (20 mg/mL), NADH (0.8 mM), and (±)-**26a** (40 µmol) were added into 1X PBS (pH 7.4, 500 µL, 20 mM) containing 10% (v/v) ^{i}PrOH in a 4 mL glass vial. For all biocatalyst preparations, the mixture was stirred and irradiated by blue LED (405 nm) at RT for 1–2 h. Then, following **general procedure C** for bio-reduction, ketone **1b** (10 µmol) was added to the whole-cell suspension and the mixture was stirred at 750 RPM at RT for 5 min. At the end point, 6 M HCl (10 µL) was added to precipitate biological materials, followed by saturated $NaHCO_3$ (75 µL) for neutralization. The reaction yields for alcohol (*S*)-**1a** were determined by $^{19}F\{^{1}H\}$ NMR spectroscopy.

5.2 Molecular docking

The computational workflow for molecular docking is summarized in Scheme S2 and explained in detail below.

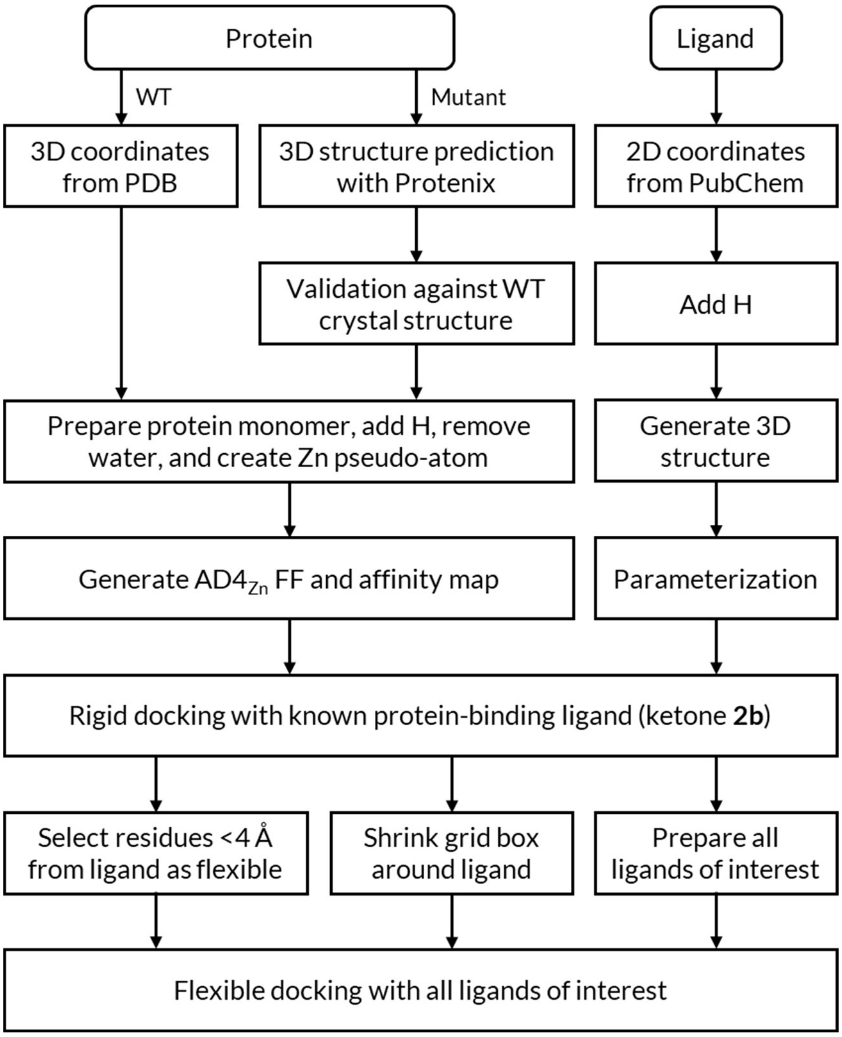

Scheme S2. General workflow for molecular docking and *in silico* site-directed mutagenesis.

Ligands were prepared by adding hydrogen atoms at pH 7.4 and generating three dimensional structures using Open Babel,[9] then parametrized with Meeko (https://github.com/forlilab/meeko). The receptor was obtained as the monomeric chain B of a crystal structure of *Gc*APRD (PDB accession: 6ISV).[10] It was prepared by removing all water molecules and adding hydrogen atoms using the ADFRsuite,[11] followed by separating flexible residues (for flexible docking only) and creating a pseudo-atom for Zn with a tetrahedral coordination sphere using the AutoDock Suite.[12]

To identify a suitable grid center, an AutoDock4$_{Zn}$ forcefield and resulting affinity maps were generated for the rigid receptor with a cubic grid box of 11390.6 Å^3 (22.5 Å × 22.5 Å × 22.5 Å) centered on the Zn atom. Rigid docking was performed on ketone **2b** with Autodock Vina 1.2.5 using the AutoDock4.2 (AD4) scoring function with an exhaustiveness of 64.[13]

After obtaining a crude binding pose for ketone **2b** via rigid docking, the flexible docking of all substrates was performed with Autodock Vina 1.2.5 using the AD4 scoring function with a cubic grid box of 3375.0 Å^3 (15 Å × 15 Å × 15 Å) centered on the 1′ aryl carbon atom of **2b** and an exhaustiveness of 64. The flexible residues Ser47, Ile51, Phe56, Leu122, Leu264, Phe287, and Trp288 were selected based on their proximity within 4 Å to ketone **2b** in the simulated *Gc*APRD-**2b** complex obtained from rigid docking and limited conservation among homologs (Table S3).

For site-directed mutagenesis simulations, wild-type (WT) and mutant structures were predicted using AlphaFold3 and Protenix Server (multiple sequence alignment mode) and validated by alignment against the crystal structure on PyMOL.[14] No large-scale structural modifications were predicted. The predicted WT structure with the lowest RMSD against the crystal structure was generated by Protenix (0.52 Å), while the AlphaFold3 counterpart had a slightly higher RMSD (0.60 Å). The Protenix-predicted structure with the lowest RMSD against the WT crystal structure was chosen for each mutant to use in flexible docking with a cubic grid box of 1423.8 Å^3 (11.25 Å × 11.25 Å × 11.25 Å) and the same flexible residue positions.

A

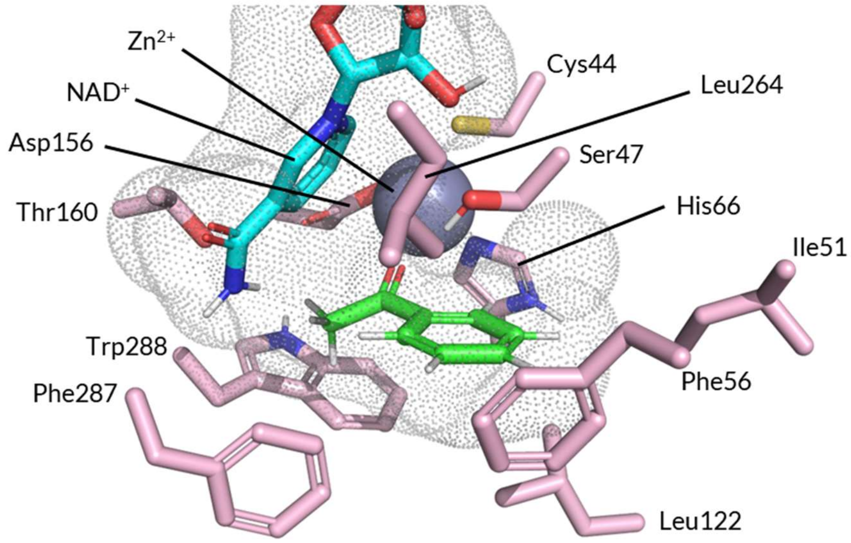

B

Ligand	Productive pose	Unproductive pose
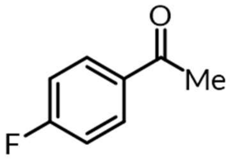 **1b**	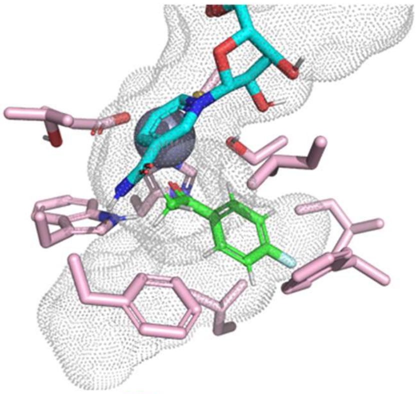	Not predicted
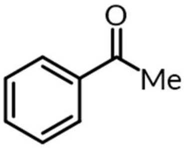 **2b**	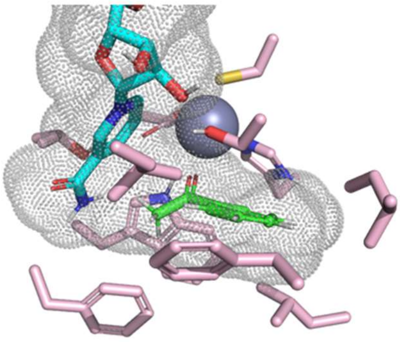	Not predicted
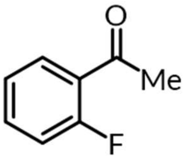 **3b**	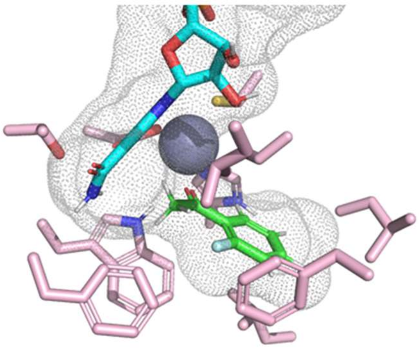	Not predicted

(continued)

**Ligand**	**Productive pose**	**Unproductive pose**
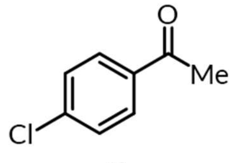 **4b**	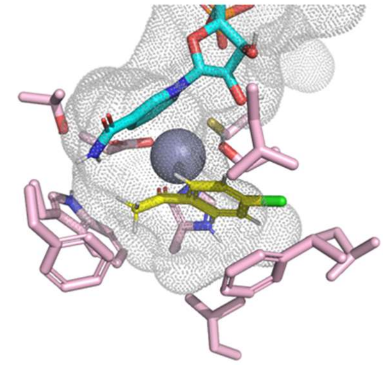	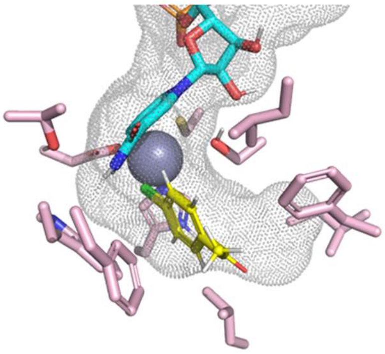
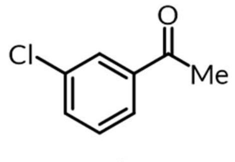 **5b**	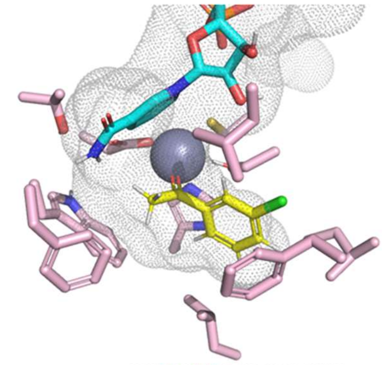	Not predicted
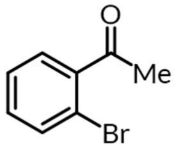 **6b**	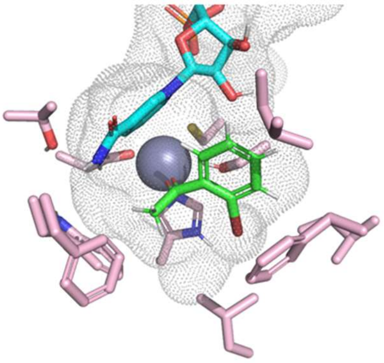	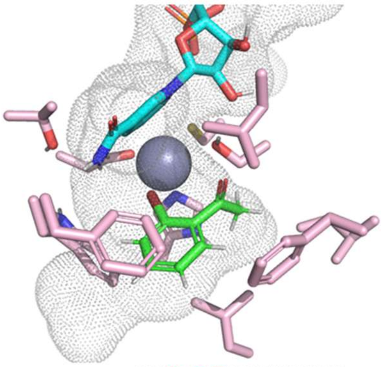
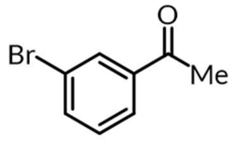 **7b**	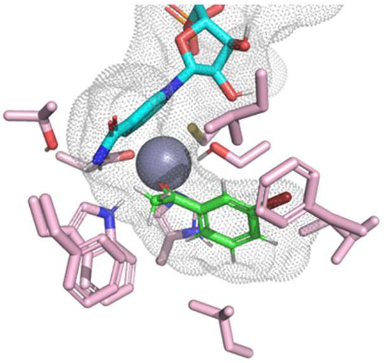	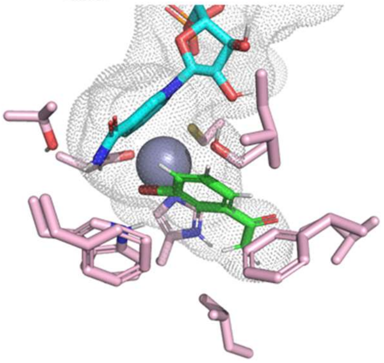

(continued)

Ligand	Productive pose	Unproductive pose
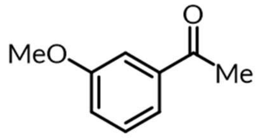 **11b**	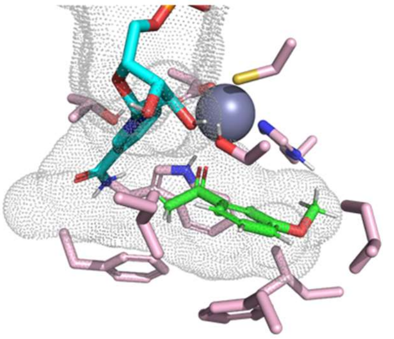	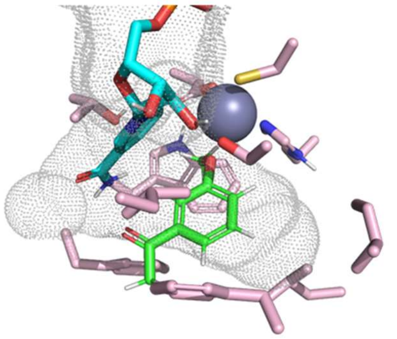
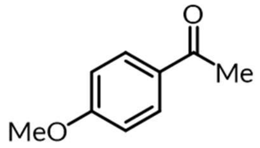 **12b**	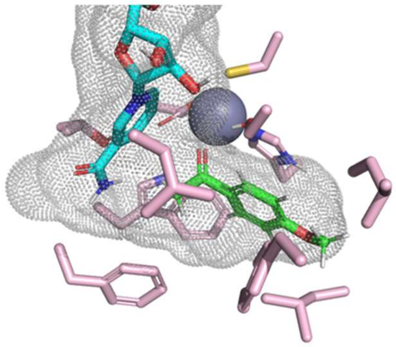	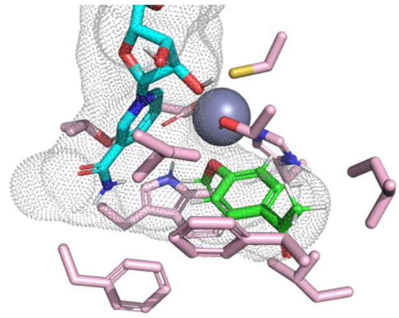
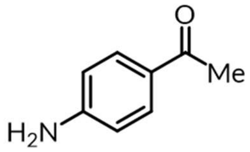 **14b**	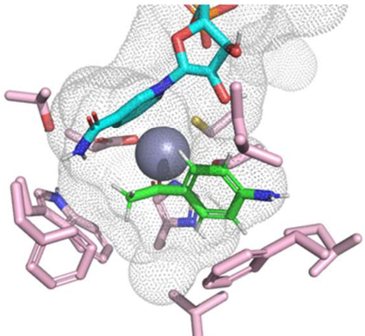	Not predicted
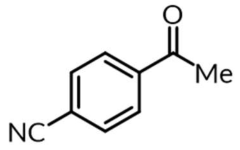 **17b**	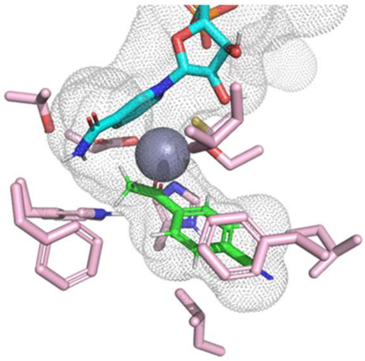	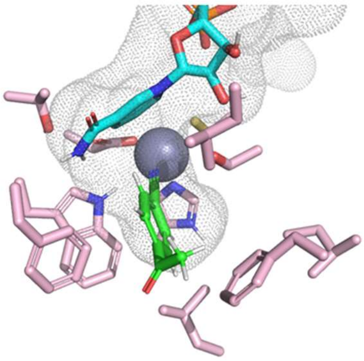

(continued)

Ligand	Productive pose	Unproductive pose

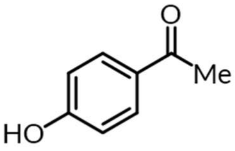

18b

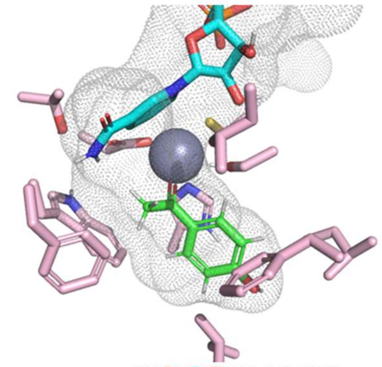

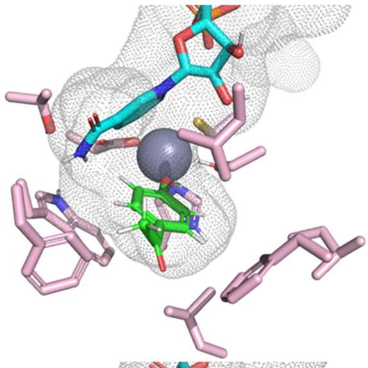

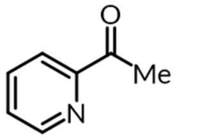

20b

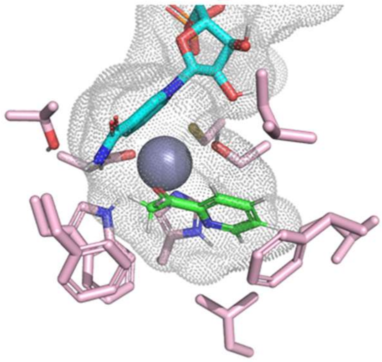

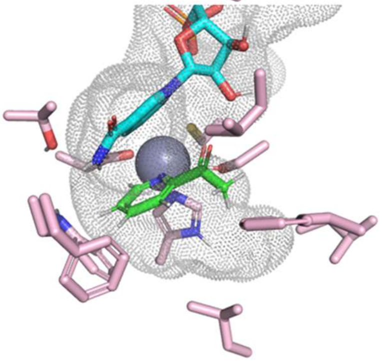

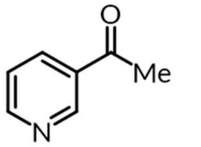

21b

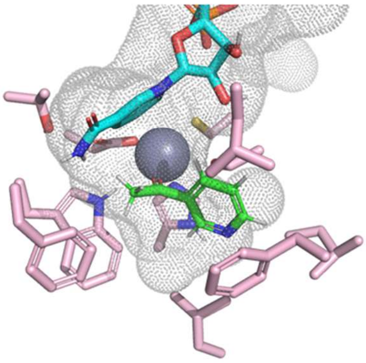

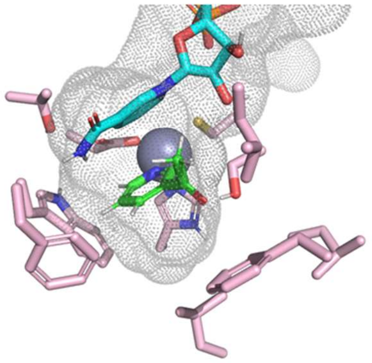

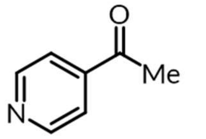

22b

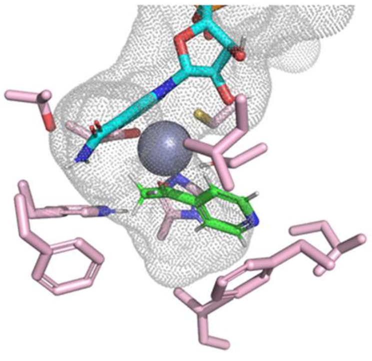

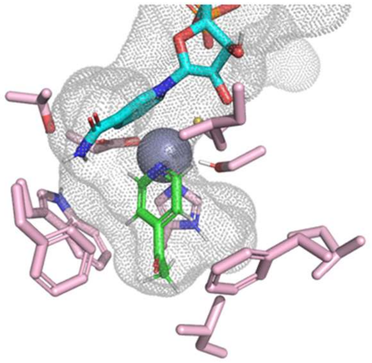

(continued)

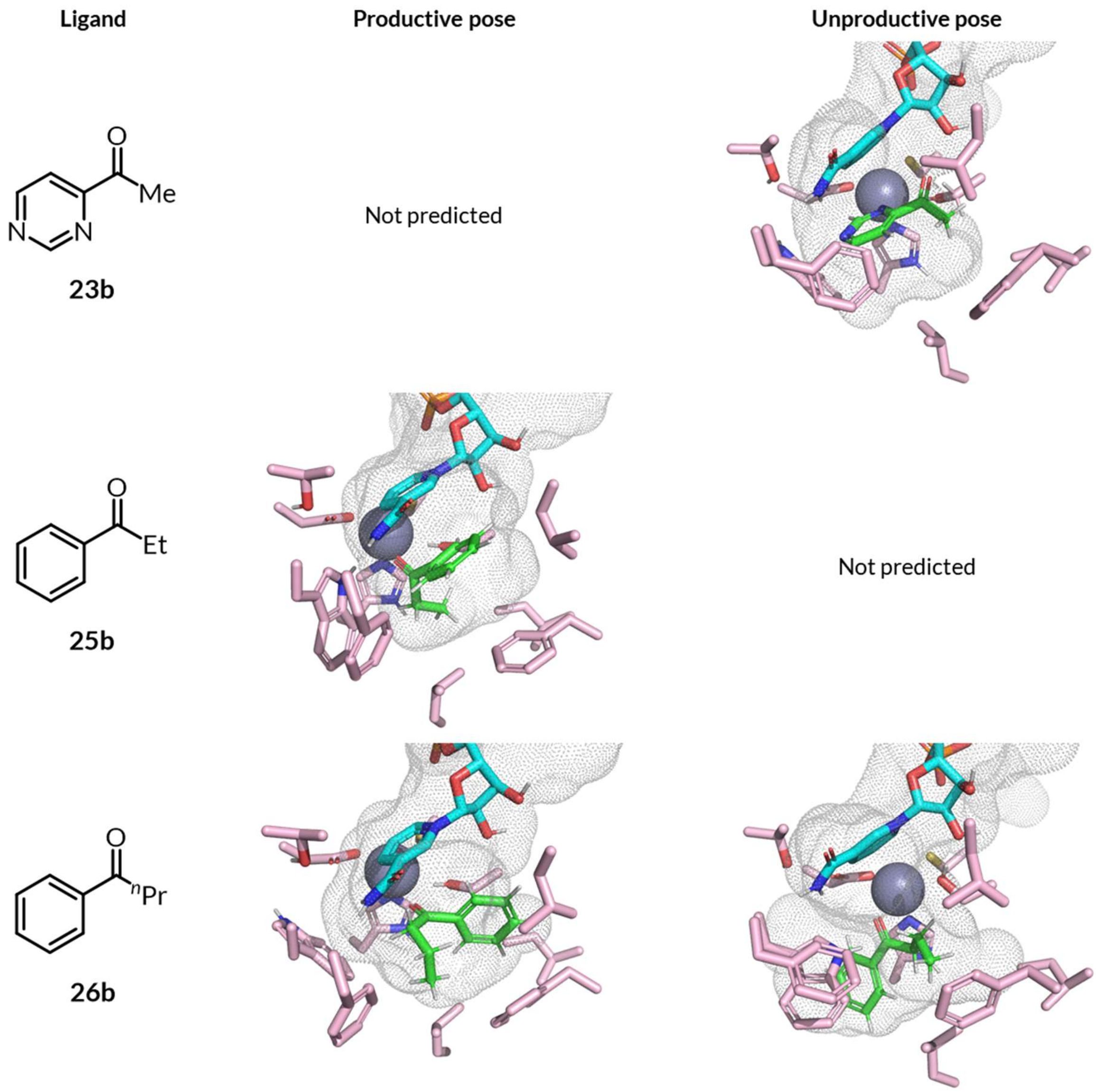

Figure S6. Molecular docking studies for substrate scope. Predicted *Gc*APRD-ketone binding pose for ketone **2b** by rigid docking (**A**) and poses for various ketones by flexible docking (**B**). Binding cavities are shown as dotted internal surfaces. Non-polar hydrogens are omitted for clarity except for the ketone ligands.

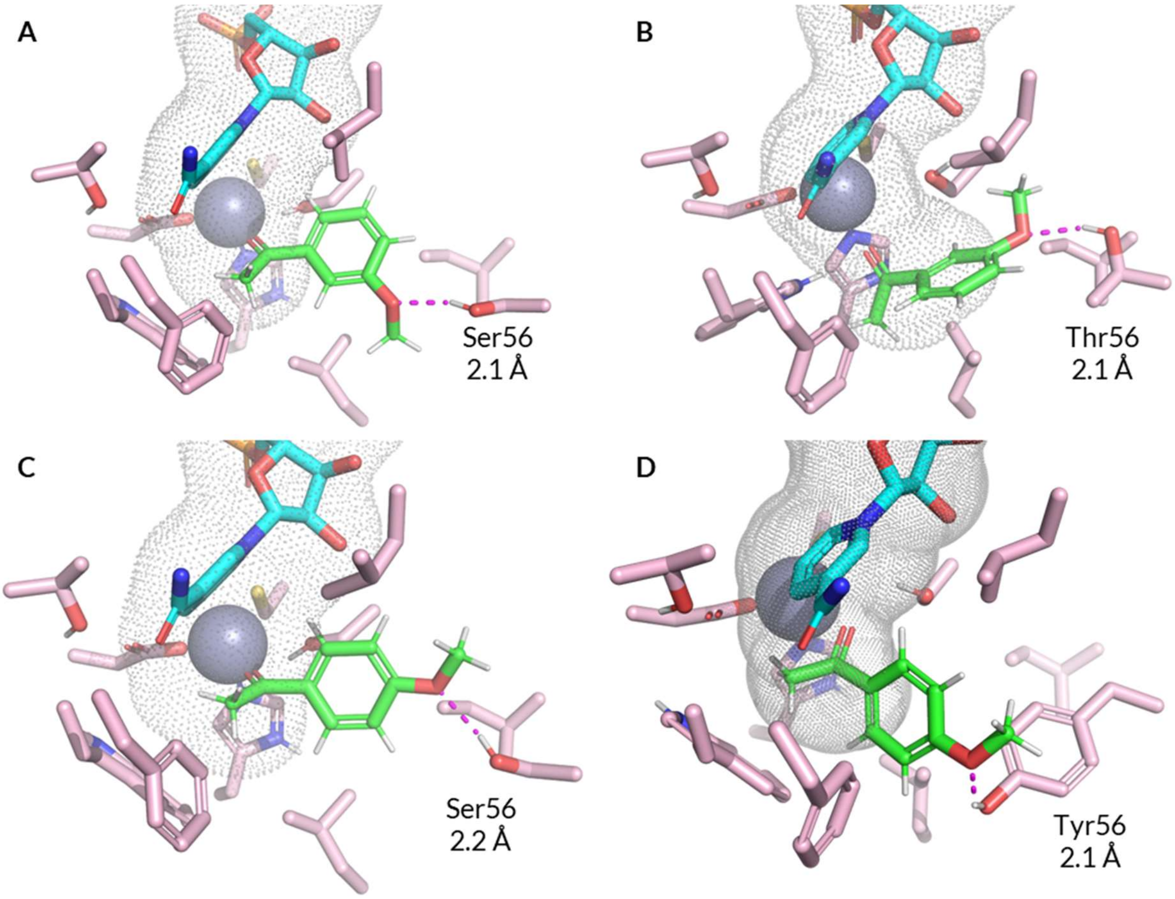

Figure S7. Molecular docking studies for simulated mutants. Predicted *Gc*APRD-ketone binding poses for ketones **11b** (**A–B**) and **12b** (**C–D**) by flexible docking in mutants Phe56Ser, Phe56Thr, and Phe56Tyr. Binding cavities are shown as dotted internal surfaces. Predicted hydrogen bonds are shown as dashed magenta lines with amino acid substitutions and interaction distances labeled. Non-polar hydrogens are omitted for clarity except for the ketone ligands.

5.3 Bioinformatic analysis

A multiple sequence alignment was performed on all 3,477 amino acid sequences of the NCBI CDD entry cd08254 using Clustal Omega (EMBL-EBI web server) with the default parameters.[15] Prevalences and occupancies were calculated using Jalview.[16]

Table S3. Prevalences of each amino acid residue in specified positions (referenced to *Gc*APRD) and the occupancies of each position in the multiple sequence alignment of the NCBI CDD entry cd08254. The values for residues present in *Gc*APRD are underlined.

	Prevalence at position (%)										
**Residue**	45	47	51	56	66	122	156	160	264	287	288
Arg	0.0	0.1	0.2	0.3	0.0	0.4	0.0	0.0	0.0	2.3	0.0
His	0.0	0.0	0.1	0.3	99.4	1.1	0.0	0.0	0.0	0.2	0.0
Lys	0.1	0.0	0.0	0.8	0.0	2.6	0.0	0.0	0.0	0.9	0.0
Asp	0.0	0.0	0.0	3.4	0.0	0.0	94.6	0.0	0.0	0.1	4.4
Glu	0.0	0.0	0.2	1.0	0.0	0.0	0.1	0.0	0.0	0.0	0.1
Ser	0.1	65.9	0.1	5.3	0.0	1.4	0.0	2.6	4.6	3.3	3.3
Thr	0.0	32.2	1.1	1.8	0.0	1.1	0.0	96.1	4.0	3.1	0.1
Asn	0.0	0.0	0.0	1.0	0.0	23.5	0.1	0.1	0.0	4.1	1.0
Gln	0.0	0.0	0.8	1.0	0.0	0.1	0.0	0.0	0.1	0.8	0.0
Cys	97.8	0.0	0.1	0.1	0.0	0.4	5.0	0.7	0.1	0.4	0.4
Gly	0.2	0.1	0.0	10.6	0.0	0.4	0.1	0.2	0.1	0.3	82.8
Pro	0.0	0.1	0.0	0.7	0.0	0.0	0.1	0.0	0.1	0.0	0.0
Ala	0.0	0.0	3.0	1.0	0.0	0.7	0.0	0.2	0.7	1.7	2.5
Val	0.0	0.1	5.7	24.0	0.0	16.4	0.0	0.1	2.3	2.0	0.1
Ile	0.1	0.0	13.3	8.2	0.0	16.2	0.0	0.0	12.0	3.3	0.1
Leu	0.0	0.0	39.1	20.1	0.0	12.9	0.0	0.0	36.3	15.2	0.1
Met	0.0	0.0	0.9	1.6	0.0	1.8	0.0	0.0	13.0	1.5	0.0
Phe	0.1	0.0	11.9	8.3	0.0	15.9	0.0	0.0	16.7	8.5	0.0
Tyr	0.1	0.0	22.2	0.4	0.0	5.1	0.0	0.0	9.4	31.4	1.6
Trp	0.0	0.0	0.1	0.1	0.0	0.1	0.0	0.0	0.0	20.0	2.7
**Occupancy (%)**	98.5	98.5	98.6	89.6	99.5	99.9	100.0	100.0	99.4	99.1	99.1

6 Concurrent linear deracemization

6.1 Reaction optimization

Table S4. Average relative irradiance of EvoluChem LEDs (HepatoChem) measured in a EvoluChem PhotoRedOx Box (HepatoChem), as reported by HepatoChem.[17] Photon flux is calculated from relative irradiance (I) and wavelength (λ) as follows:

$$Photon\ flux[\mu mol\ cm^{-2}\ s^{-1}] = I[mW\ cm^{-2}] \times \lambda[nm] \times 8.36 \times 10^{-6}$$

Model	Emission λ (nm)	Relative irradiance (mW cm^{-2})	Photon flux (μmol cm^{-2} s^{-1})
365PF non-dimmable	365	9	0.0274
380PF non-dimmable	380	8	0.0254
405PF non-dimmable	405	28	0.0947
425PF non-dimmable	425	33	0.1171

Table S5. Following **general procedure D**, SAS (30 mol%), *E. coli*/*Gc*APRD (20 mg/mL), NADH (0.8 mM), and (±)-alcohol (10 μmol) were added into 1X PBS (pH 7.4, 500 μL, 20 mM) containing 30% (v/v) ^{i}PrOH in a 4 mL glass vial. The mixture was stirred and irradiated by blue LED (405 nm) at RT for 4 h. The reaction yields were determined by ^{1}H NMR spectroscopy. Enantiomeric ratios (er) were determined following **general procedure E**.

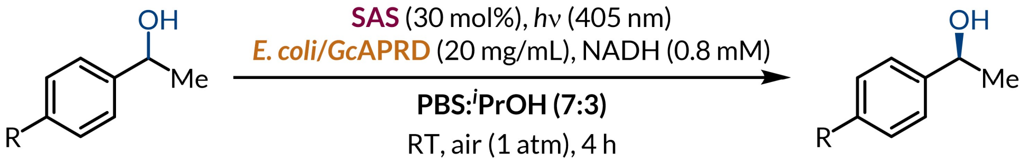

Product (R group)	Yield (%)	er
(*S*)-**10a** (*p*-I)	57 (93)	50:50
(*S*)-**19a** (*p*-Ph)	65 (70)	50:50

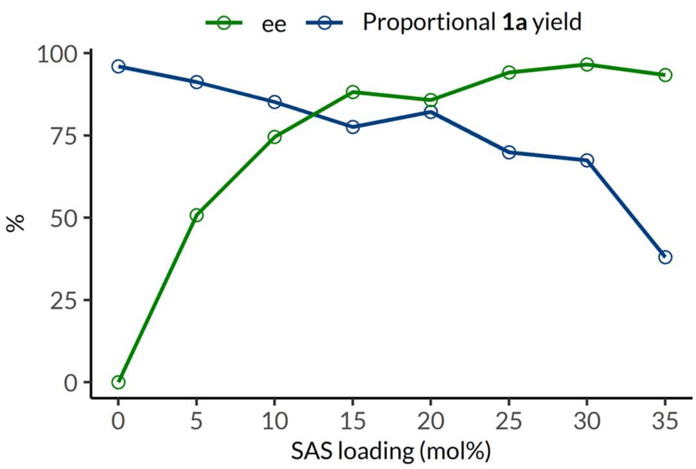

Figure S8. Effect of photocatalyst loading. Following **general procedure D**, SAS (5–30 mol%), *E. coli*/*Gc*APRD (20 mg/mL), NADH (0.8 mM), and (±)-**1a** (1.4 µL, 10 µmol) were added into 1X PBS (pH 7.4, 500 µL, 20 mM) containing 10% (v/v) ^{i}PrOH in a 4 mL glass vial. The mixture was stirred and irradiated by blue LED (405 nm) at RT for 4 h. Reaction yields were determined by $^{19}F\{^{1}H\}$ NMR spectroscopy. Enantiomeric excess (ee) was determined following **general procedure E**.

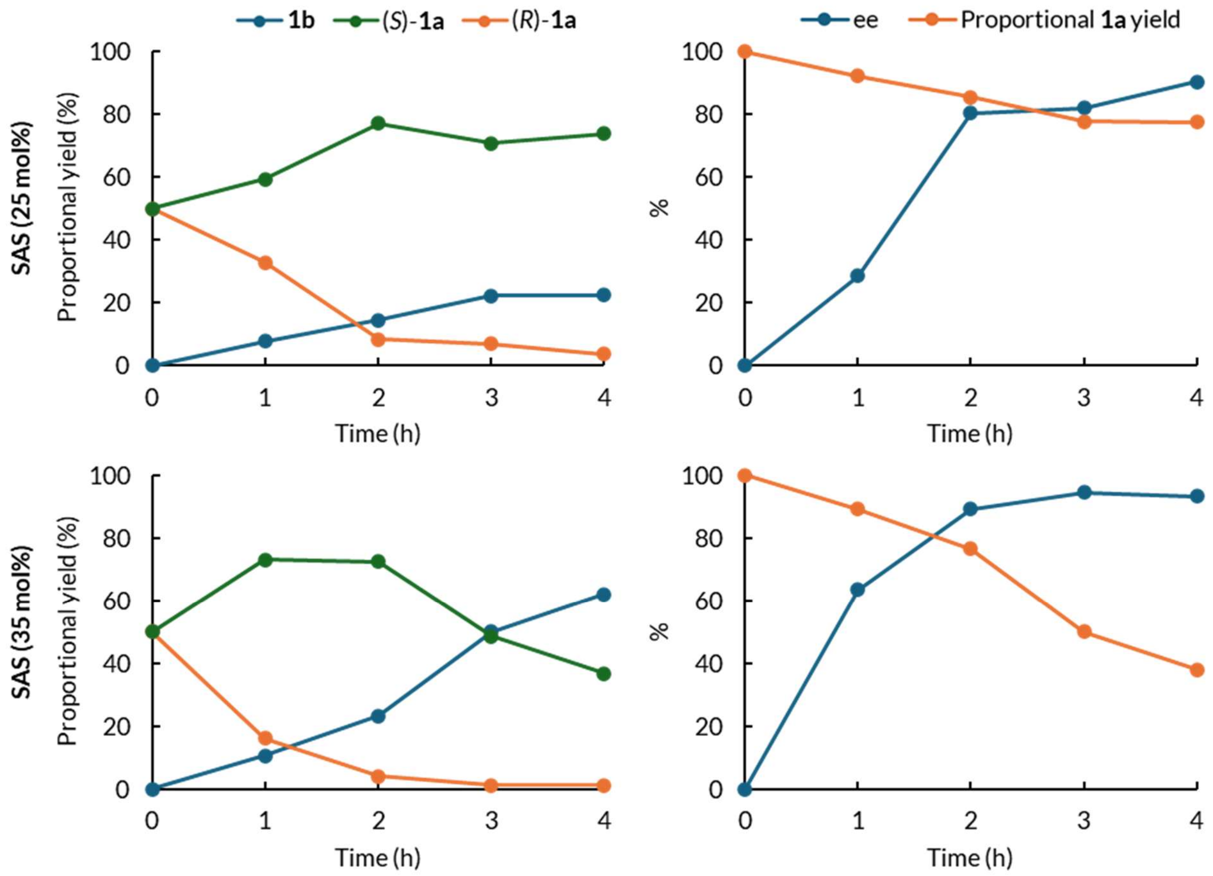

Figure S9. **Kinetic profiles at different photocatalyst loadings.** Following **general procedure D**, SAS (25 or 35 mol%), *E. coli*/*Gc*APRD (20 mg/mL), NADH (0.8 mM), and (±)-**1a** (1.4 μL, 10 μmol) were added into 1X PBS (pH 7.4, 500 μL, 20 mM) containing 10% (v/v) ^{i}PrOH in a 4 mL glass vial. The mixture was stirred and irradiated by blue LED (405 nm) at RT for the specified time. Reaction yields were determined by $^{19}F\{^{1}H\}$ NMR spectroscopy. Enantiomeric excess (ee) was determined following **general procedure E**.

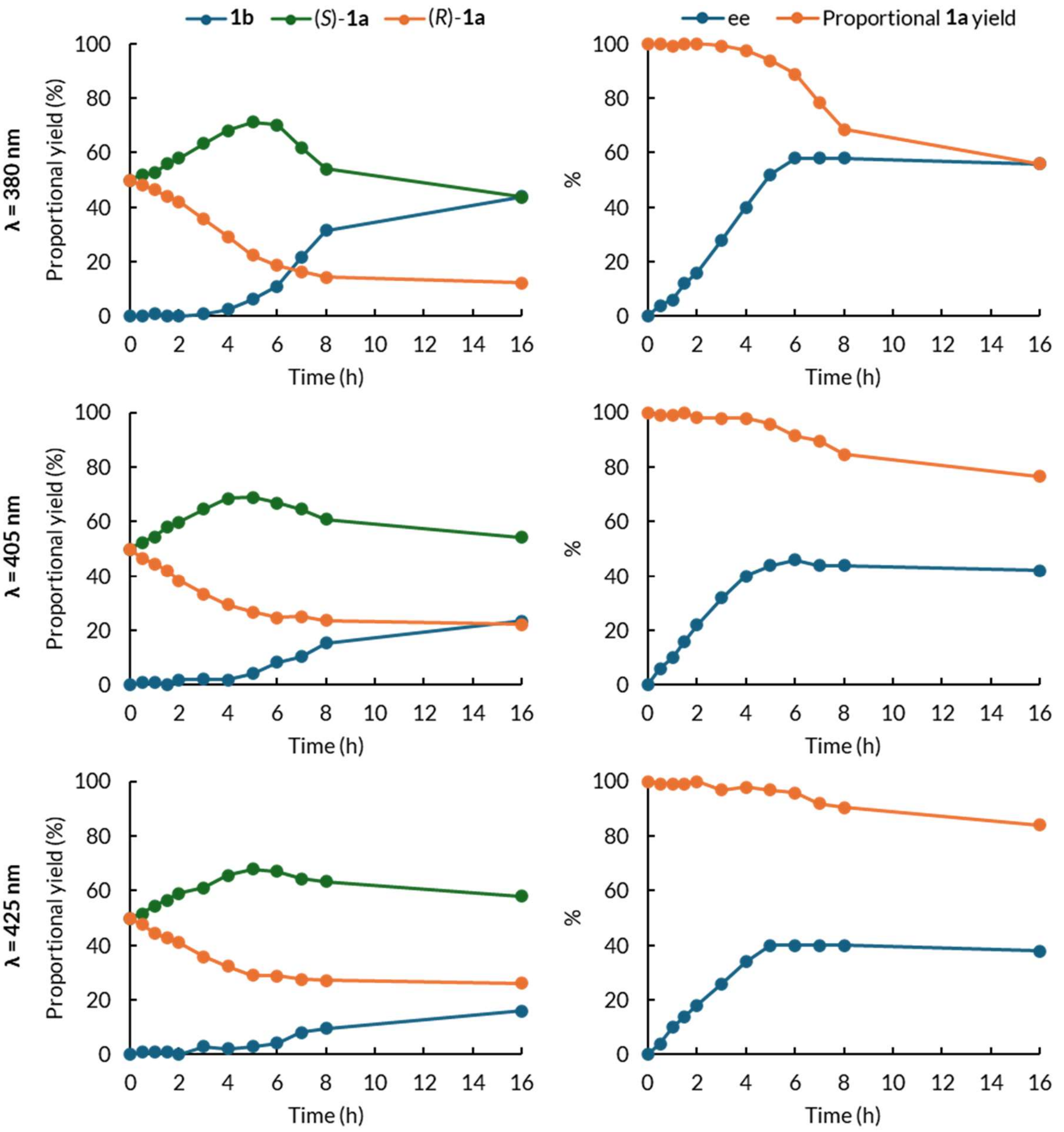

Figure S10. Kinetic profiles at different irradiation wavelengths. Following **general procedure D**, SAS (5 mol%), *E. coli*/*Gc*APRD (20 mg/mL), NADH (0.8 mM), and (±)-**1a** (1.4 µL, 10 µmol) were added into 1X PBS (pH 7.4, 500 µL, 20 mM) containing 15% (v/v) ^{i}PrOH in a 4 mL glass vial. The mixture was stirred and irradiated by UV or blue LED (380, 405, or 425 nm) at RT for the specified time. Reaction yields was determined by $^{19}F\{^{1}H\}$ NMR spectroscopy. Enantiomeric excess (ee) was determined following **general procedure E**.

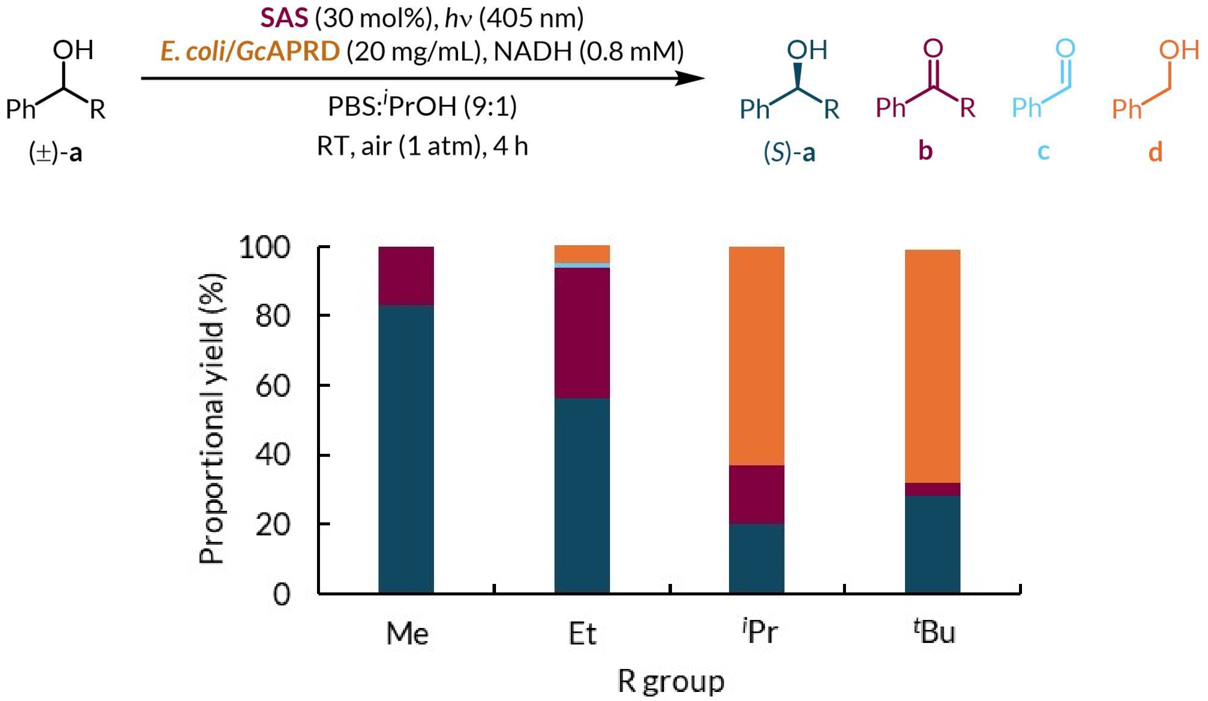

Figure S11. Effect of increasing alkane branching. Following **general procedure D**, SAS (30 mol%), *E. coli*/*Gc*APRD (20 mg/mL), NADH (0.8 mM), and (±)-alcohols (10 µmol) were added into 1X PBS (pH 7.4, 500 µL, 20 mM) containing 10% (v/v) ^{i}PrOH in a 4 mL glass vial. The mixture was stirred and irradiated by blue LED (405 nm) at RT for 4 h. Yields were determined by ^{1}H NMR spectroscopy.

6.2 Deracemization with whole-cell suspension

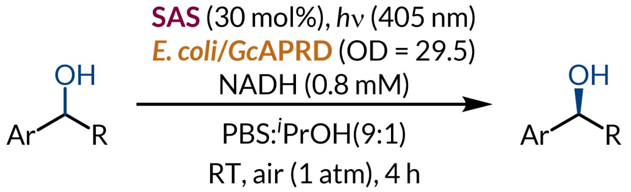

To prepare a whole-cell suspension, an overnight pre-culture of *E. coli* BL21(DE3) harboring a pADH-*Gc*APRD plasmid was incubated in LB (60 mL) containing ampicillin (100 µg/mL) and incubated at 37 °C until reaching OD_{600} of 0.6. Protein expression was induced by 1.0 mM IPTG for 20 h at 20 °C. Cells were harvested by centrifugation (4,500 × *g*, 15 min), washed with PBS (3 × 50 mL), and resuspended in 1X PBS (pH 7.4, 410 µL) containing SAS (3 µmol), NADH (0.8 mM), and ^{i}PrOH (10% v/v) in a 4 mL glass vial to reach OD_{600} of 29.5.

Following **general procedure D**, racemic benzylic alcohol (10 µmol) was added to the whole-cell suspension and the mixture was stirred and irradiated by Evoluchem blue LED (405 nm, HepatoChem) in EvoluChem PhotoRedOx Boxes (HepatoChem) at RT for 4 h. Then, 6 M HCl (10 µL) was added at the end point to precipitate biological materials, followed by saturated $NaHCO_3$ (75 µL) for neutralization.

For yield determination by $^{19}F\{^1H\}$ NMR spectroscopy, the reaction mixture was homogenized by the direct addition of DMSO-d_6 with TFE (0.33 eq.) as internal standard, vigorous shaking with a vortexer (3 min, 3,000 RPM), followed by centrifugation (3 min, 15,000 × *g*) to obtain a clarified supernatant for product detection.

6.3 Viability assay for WCBs

Following **general procedure D**, SAS (30 mol%), *E. coli*/*Gc*APRD lyophilizate (20 mg/mL), NADH (0.8 mM), and (±)-**1a** (11.1 µmol) were added into 1X PBS (pH 7.4, 555 µL, 20 mM) containing 10% (v/v) ^{i}PrOH in a 4 mL glass vial and stirred at RT for 10 min. Separately, 5 µL and 50 µL aliquots of the reaction mixture were diluted with 1X PBS to a total volume of 200 µL and plated on LB agar containing ampicillin (100 µg/mL). The remaining reaction mixture was stirred and irradiated by blue LED (405 nm) at RT for 4 h. Then, 5 µL and 50 µL aliquots of the mixture were diluted and plated as above. All plates were incubated at 37 °C overnight and colonies were counted for colony-forming unit (CFU) calculations.

Table S6. CFU assay pre- and post-reaction. No CFUs were observed on LB agar plates post-reaction.

Sample	CFU/mL
Pre-reaction	7.92×10^3
Post-reaction	$< 2.00 \times 10^2$

7 Kinetic model

7.1 Derivation of a kinetic model for concurrent linear deracemization

The reaction is modeled as five fundamental reactions (Figure S12) involving five tracked species: (R)-alcohol (R), (S)-alcohol (S), ketone intermediate (I), total photocatalyst (PC_T), and total active biocatalyst (BC_T). PC, PC^*, and PCH_2 denote the ground, triplet excited, and hydroquinone (reduced) states of the photocatalyst, the sum of which is equal to PC_T assuming a negligible singlet excited state population due to efficient intersystem crossing. O_2 is treated as being excess. All model parameters are described in Table S7.

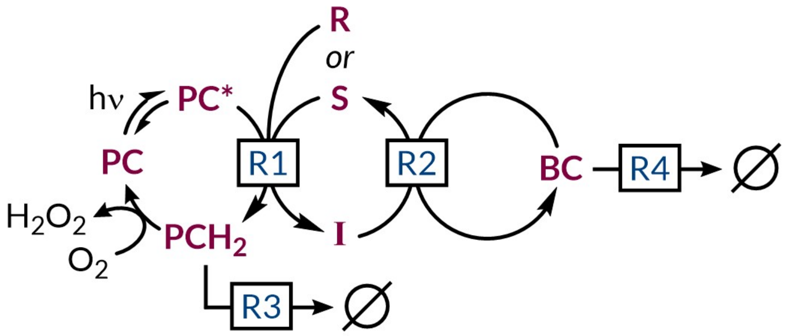

Figure S12. Detailed structure of the kinetic model for concurrent linear deracemization. The tracked species are denoted in bold and purple, fundamental reactions are denoted in blue, and degradation products are denoted by slashed circles (∅).

To model the biocatalytic cycle, we investigated the properties of *Gc*APRD. Matsuda and colleagues reported the purified enzyme to follow Michaelis-Menten kinetics,[18] which we found to remain true for the *E. coli*/*Gc*APRD WCB (Figure S13A). The biocatalytic cycle was modeled using Michaelis-Menten kinetics (equation S2) while enzyme degradation was assumed to be first order in accordance with the open Michaelis-Menten model developed by Gabrielsson and Peletier (equation S3).[19] The rate equation for $R2$ can be expressed in terms of the standard parameters V_{max} and K_M, where V_{max} is the maximum initial rate with all of BC in enzyme-substrate form before any enzyme degradation had occurred, thus implicitly accounting for initial $[BC_T]$ ($[BC_T]_0$) as shown in equation S1, which is laborious to measure in whole-cell systems. The scaling factor $\frac{[BC_T]}{[BC_T]_0}$ describes the effect of BC degradation on V_{max}.

$$V_{max} = k_{R2}[BC_T]_0 \qquad \text{(S1)}$$

$$R2 = \frac{k_{R2}[BC_T]_0[I]}{K_M + [I]} \cdot \frac{[BC_T]}{[BC_T]_0} = \frac{V_{max}[I]}{K_M + [I]} \cdot \frac{[BC_T]}{[BC_T]_0} \qquad \text{(S2)}$$

$$R4 = k_{deg,BC}[BC_T] \qquad \text{(S3)}$$

On the other hand, the non-stereospecific photocatalytic oxidation of R and S is described according to the homogeneous photocatalysis model illustrated by Bloh.[20] The kinetics of photocatalyst excitation, relaxation, and regeneration by O_2 are modeled with a steady-state approximation due to their significantly faster timescales than that of changes in substrate concentrations. Therefore, the fractions of total photocatalyst (PC_T) in each state (PC, PC^*, PCH_2) are approximated as constants. First, total photocatalyst degradation is modeled as first order decay with equation S4 as equivalent to $R3$, since it is the only pathway for PC_T depletion. The rate constant $k_{deg,PC}$ implicitly accounts for the fraction of photocatalyst present in the reduced hydroquinone form (PCH_2).

$$R3 = k_{R3}[PCH_2] = k_{R3}\frac{[PCH_2]}{[PC_T]} \cdot [PC_T] = k_{deg,PC}[PC_T] \qquad \text{(S4)}$$

Then, to simplify the Bloh model, we derived physically meaningful composite parameters k_{ex} and K_{eff} in equations S5 and S6.

$$k_{ex,PC} = \phi \cdot \langle L_p^{\alpha} \rangle \qquad \text{(S5)}$$

$$K_{eff} = \frac{1}{k_{cat} \cdot \tau} = \frac{k_{relax}}{k_{cat}} \qquad \text{(S6)}$$

Substitution with equations S5 and S6 and rearrangement of the Bloh model yield the simplified two-parameter model in equations S7 and S8, analogous to the Michaelis-Menten model. Given that k_{ex} implicitly accounts for $[PC^*]$ and $\frac{[PC^*]}{[PC_T]}$ is approximately constant, the scaling factor $\frac{[PC_T]}{[PC_T]_0}$ describes the decreasing photocatalytic reaction rates due to PC degradation.

$$R1_R = \frac{\phi \cdot \langle L_p^{\alpha} \rangle \cdot \tau \cdot k_{R1}[R]}{1 + \tau \cdot k_{R1}[R]} \cdot \frac{[PC_T]}{[PC_T]_0} = \frac{k_{ex} \cdot [R]}{K_{eff} + [R]} \cdot \frac{[PC_T]}{[PC_T]_0} \qquad \text{(S7)}$$

$$R1_S = \frac{\phi \cdot \langle L_p^{\alpha} \rangle \cdot \tau \cdot k_{R1}[S]}{1 + \tau \cdot k_{R1}[S]} \cdot \frac{[PC_T]}{[PC_T]_0} = \frac{k_{ex} \cdot [S]}{K_{eff} + [S]} \cdot \frac{[PC_T]}{[PC_T]_0} \qquad \text{(S8)}$$

Note that the parameter k_{ex} is only dependent on the photon source set-up, light scattering, and absorbance, but independent from substrates that do not absorb light in the wavelength

used. This effectively renders k_{ex} an easily measurable constant for a specific set of conditions and equipment, but optionally a parameter to investigate the effect of changing conditions with the same substrate. Finally, the dynamic behavior of the 5 species of interest can be described as an ordinary differential equation (ODE) system in equation S9, expressed in terms of the above fundamental reactions.

$$\begin{pmatrix} R' \\ S' \\ I' \\ {PC_T}' \\ {BC_T}' \end{pmatrix} = \begin{pmatrix} -R1_R \\ -R1_S + R2 \\ R1_R + R1_S - R2 \\ -R3 \\ -R4 \end{pmatrix} \tag{S9}$$

Table S7. Parameters of the overall kinetic model.

Parameter[a]	Physical meaning	Units[b]
$k_{R1} - k_{R4}$	Rate constants of fundamental reactions $R1 - R4$	variable
$\boldsymbol{V_{max}}$	Maximum biocatalytic reaction rate	$mM\ min^{-1}$
$\boldsymbol{K_M}$	Michaelis constant, i.e. relative rates of enzyme-substrate complex decay to its formation, also substrate concentration at which $R2 = \frac{V_{max}}{2} \cdot \frac{[BC_T]}{[BC_T]_0}$	mM
$\boldsymbol{k_{deg,BC}}$	Rate constant of biocatalyst degradation	min^{-1}
$\boldsymbol{k_{deg,PC}}$	Rate constant of photocatalyst degradation	min^{-1}
ϕ	Photocatalyst quantum yield	unitless
$\langle L_p^{\alpha} \rangle$	Average volumetric rate of photon absorption	$mM\ min^{-1}$
k_{cat}	Second order rate constant of the reaction: $PC^* + (R \text{ or } S) \rightarrow PCH_2 + I$	$mM^{-1}\ min^{-1}$
τ	Photocatalyst half-life	min
k_{relax}	First order rate constant of photocatalyst relaxation, inverse of half-life τ	min^{-1}
$\boldsymbol{k_{ex}}$	Average rate of photocatalyst excitation	$mM\ min^{-1}$
$\boldsymbol{K_{eff}}$	Effective reactivity, i.e. relative rates of photocatalyst relaxation to $R1$ reaction, also substrate concentration at which $R1 = \frac{k_{ex}}{2} \cdot \frac{[PC_T]}{[PC_T]_0}$	mM

[a]Parameters fitted in the final model (equation S9) are in bold. [b]Consistency of units with parameters in bold is prioritized over convenient magnitudes. For instance, while τ is likely on a much shorter timescale than minutes, the magnitude of its units are unimportant as it is not explicitly considered in equation S9.

7.2 Experimental measurements of parameters in a standard system

To obtain realistic estimations for the relevant parameters outlined in Table S7, initial rate studies were performed individually on the biocatalytic and photocatalytic cycles using conditions representative of the concurrent linear deracemization protocol.

The rate constant $k_{deg,PC}$ for $R3$ was estimated to be $8 \cdot 10^{-3}\ min^{-1}$ in section 5 by fitting a first order exponential decay model to UV-Vis measurements by least squares regression (Figure S4B). The rate constant $k_{deg,BC}$ for $R4$ was assumed to be of the same order of magnitude.

The remaining parameters for $R1$ were assayed following **general procedure B**, where SAS (30 mol%), *E. coli*/empty vector (20 mg/mL), and (±)-**1a** (2.5–40.0 µmol) were added into 1X PBS (pH 7.4, 500 µL, 5–80 mM) containing 10% (v/v) ^{i}PrOH in a 4 mL glass vial. The mixture was stirred and irradiated by blue LED (405 nm) at RT for 10 min, then 6 M HCl (10 µL) was added to precipitate biological materials, followed by saturated $NaHCO_3$ (75 µL) for neutralization. Yields were determined by $^{19}F\{^1H\}$ NMR spectroscopy.

The remaining parameters for $R2$ were assayed following **general procedure C**, where *E. coli*/*Gc*APRD (20 mg/mL), NADH (0.8 mM), and **1b** (2.5–20.0 µmol) were added into 1X PBS (pH 7.4, 500 µL, 5–40 mM) containing 10% (v/v) ^{i}PrOH in a 4 mL glass vial. The mixture was stirred at RT for 1 min, then 6 M HCl (10 µL) was added to precipitate biological materials, followed by saturated $NaHCO_3$ (75 µL) for neutralization. Yields were determined by $^{19}F\{^1H\}$ NMR spectroscopy.

Initial rates v_0 for $R1$ and $R2$ at each substrate concentration were calculated with equation S10 and fitted with the Bloh and Michaelis-Menten models by least squares regression, i.e. equations S7 and S2, respectively, assuming no catalyst degradation, i.e. $[PC_T] = [PC_T]_0$ and $[BC_T] = [BC_T]_0$, in the short reaction timescales. The fits and resulting parameters are shown in Figure S13.

$$v_0 = \frac{[Substrate]\ (mM) \cdot Proportional\ yield\ (\%)}{Time\ (min)} \qquad (S10)$$

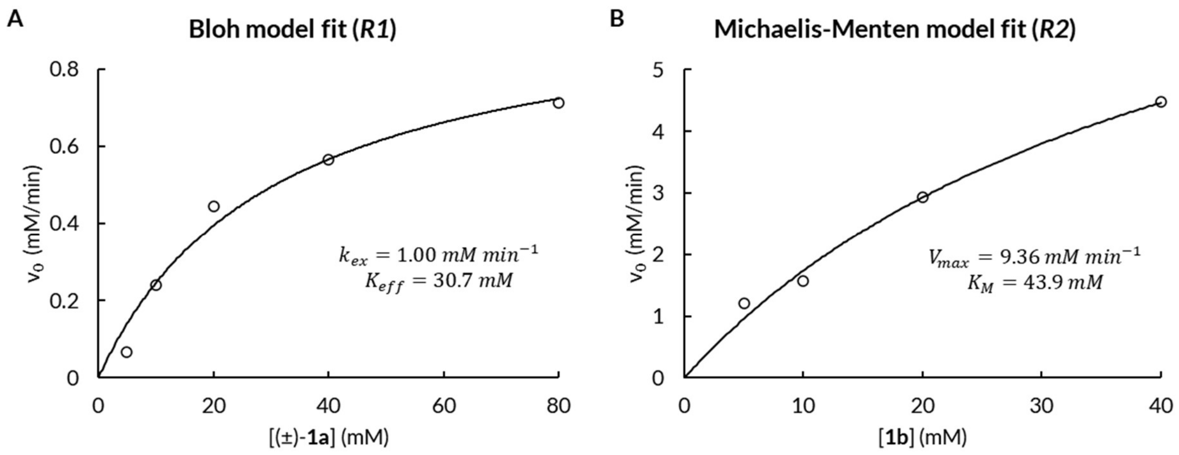

Figure S13. Parameter extraction for reactions $R1$ and $R2$. The Bloh (**A**) and Michaelis-Menten (**B**) models were fitted to initial rate data from each reaction by least squares regression to estimate parameters.

7.3 Model fitting to measured reaction trajectories

The ODE system described in equation S9 was fitted to experimentally determined trajectories of the species R, S, and I by least squares regression of the parameters V_{max}, K_M, $k_{deg,BC}$, k_{ex}, K_{eff}, and $k_{deg,PC}$ using the MATLAB 2024b *ode113* solver. Relative and absolute tolerances were set to 10^{-10} and 10^{-12}, respectively, to improve accuracy. Estimated parameters (Figures S3B and S12) were used for all substrates as initial guesses and parameter values were constrained to an order of magnitude above and below the estimated values, except k_{ex}, which was fixed to the value measured for (±)-**1a** but can be remeasured for substrates that absorb light in the irradiation wavelength or if conditions are modified. Calculated trajectories of the 5 tracked species and the rates of the 5 fundamental reactions were simulated over 25 h using the fitted parameters.

Initial concentrations of I and PC were known to be 0 mM and 6 mM. Initial concentrations of R and S were assumed to be 10 mM since a 20 mM solution of (±)-**1a** contains 50% of each enantiomer. $[BC_T]_0$ was estimated to be 0.0836 mM using constants obtained from the literature according to equation S11. The values of these constants are documented in Table S8. It should be noted that the precise value for $[BC_0]$ has a very minor influence on the fitted parameters but an estimate is required to simulate reaction trajectories.

$$[BC_T]_0\ (mM) = \frac{[BC_T]_0\ (mg\ mL^{-1}) \cdot P_{count} \cdot P_{comp}}{DW \cdot N_A} \cdot 10^6 \qquad (S11)$$

Table S8. Constants used in the estimation of $[BC_T]_0$.

Constant	Value	Ref.
Protein count per *E. coli* cell (P_{count})	$2.35 \cdot 10^6\ cell^{-1}$	21
Protein of interest composition out of total protein (P_{comp})	30%	22
E. coli dry weight per cell (DW)	$2.80 \cdot 10^{-10}\ mg\ cell^{-1}$	21
Avogadro constant (N_A)	$6.02214076 \cdot 10^{23}\ mol^{-1}$	-

Table S9. Parameter values derived from model fitting for the substrates (±)-**1a**, (±)-**7a**, (±)-**13a**, (±)-**16a**, and (±)-**18a**. The value of K_{eff} for all substrates was derived from fitting the model to the reaction trajectories with the substrate (±)-**1a**.

Substrate	Parameter	Value
(±)-**1a**	k_{ex}	$1.00\ mM\ min^{-1}$
	K_{eff}	$35.7\ mM$
	$k_{deg,PC}$	$0.0079\ min^{-1}$
	V_{max}	$9.38\ mM\ min^{-1}$
	K_M	$33.6\ mM$
	$k_{deg,BC}$	$0.0155\ min^{-1}$
(±)-**7a**	K_{eff}	$56.8\ mM$
	$k_{deg,PC}$	$0.0042\ min^{-1}$
	V_{max}	$9.37\ mM\ min^{-1}$
	K_M	$40.5\ mM$
	$k_{deg,BC}$	$0.0089\ min^{-1}$
(±)-**13a**	K_{eff}	$291.6\ mM$
	$k_{deg,PC}$	$0.0031\ min^{-1}$
	V_{max}	$9.40\ mM\ min^{-1}$
	K_M	$25.2\ mM$
	$k_{deg,BC}$	$0.0082\ min^{-1}$
(±)-**16a**	K_{eff}	$91.8\ mM$
	$k_{deg,PC}$	$0.0022\ min^{-1}$
	V_{max}	$9.36\ mM\ min^{-1}$
	K_M	$50.2\ mM$
	$k_{deg,BC}$	$0.0221\ min^{-1}$
(±)-**18a**	K_{eff}	$81.3\ mM$
	$k_{deg,PC}$	$0.0104\ min^{-1}$
	V_{max}	$9.28\ mM\ min^{-1}$
	K_M	$99.6\ mM$
	$k_{deg,BC}$	$0.0380\ min^{-1}$

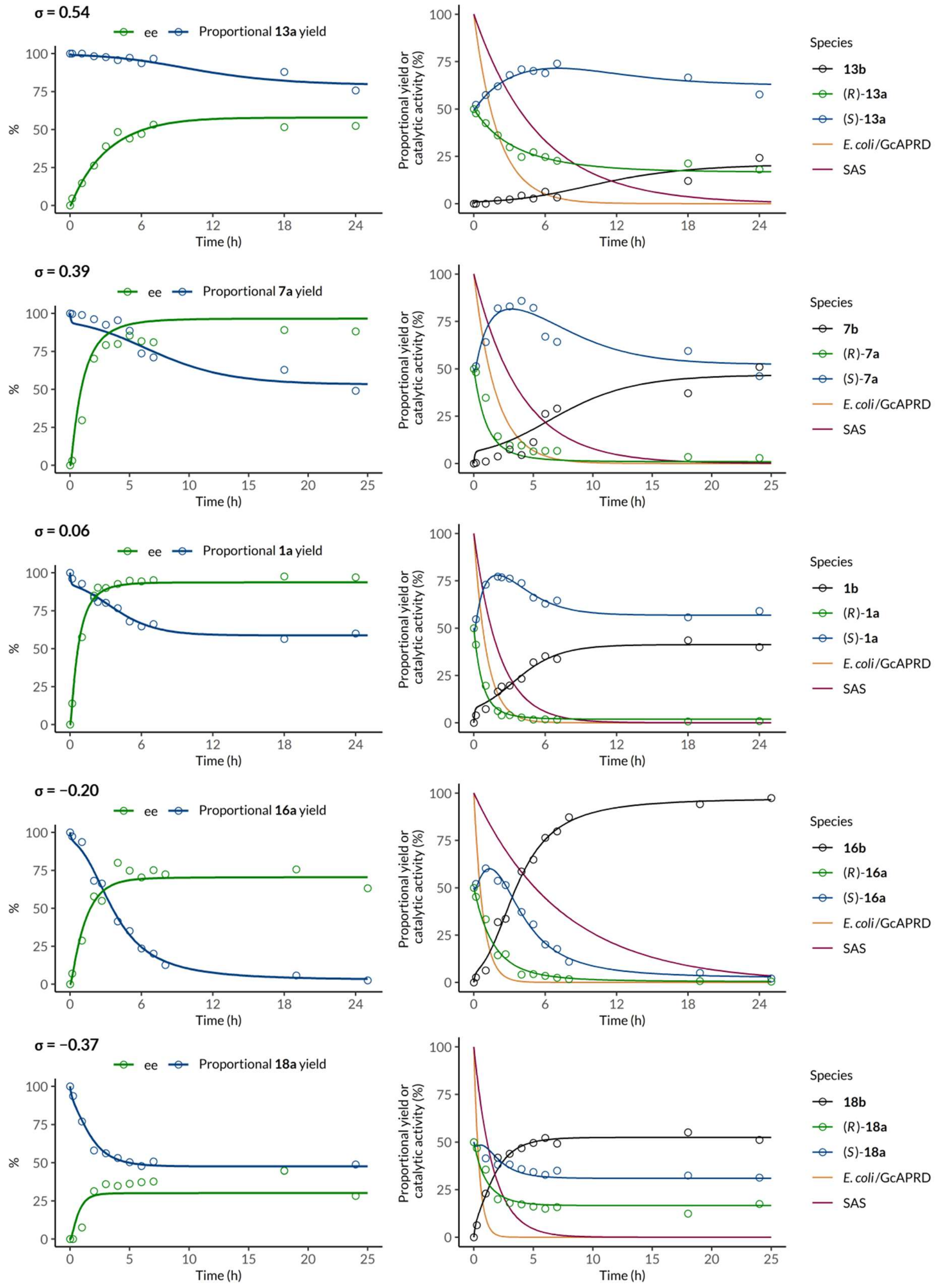

Figure S14. Model-fitted reaction trajectories in descending order of Hammett constants (σ). Hammett constants were obtained from Hansch.[23] Following **general procedure D**, SAS (30 mol%), *E. coli*/*Gc*APRD (20 mg/mL), NADH (0.8 mM), and (±)-alcohol (10 μmol) were added

into 1X PBS (pH 7.4, 500 µL, 20 mM) containing 10% (v/v) ^{i}PrOH in a 4 mL glass vial. The mixture was stirred and irradiated by blue LED (405 nm) at RT for specified durations. The yields for **1a**, **1b**, **13a**, and **13b** were determined by $^{19}F\{^{1}H\}$ NMR spectroscopy. The yields for **7a**, **7b**, **16a**, **16b**, **18a**, and **18b** were determined by ^{1}H NMR spectroscopy. Enantiomeric excess (ee) was determined following **general procedure E**. The proportional yields of each enantiomer of **1a**, **7a**, **13a**, **16a**, and **18a** were calculated from the corresponding yields and ee. Trajectories of *E. coli*/*Gc*APRD ($[BC_T]$) and SAS ($[PC_T]$) were predicted by the kinetic model.

7.4 Model-guided reaction optimization

A simple objective function was derived to aggregate the proportional alcohol yield and ee into a single term, defined as the fitness score (F) in equation S12, where Y denotes proportional alcohol yield (%) and $[S]$ and $[R]$ denote the respective concentrations of the (S)-alcohol and (R)-alcohol at any given time. F is increasingly more sensitive to changes in $\frac{[S]}{[R]}$ than in Y at higher enantiomeric ratios.

$$F = \log_{10}\left(\frac{Y}{100} \cdot \frac{[S]}{[R]}\right) \qquad \text{(S12)}$$

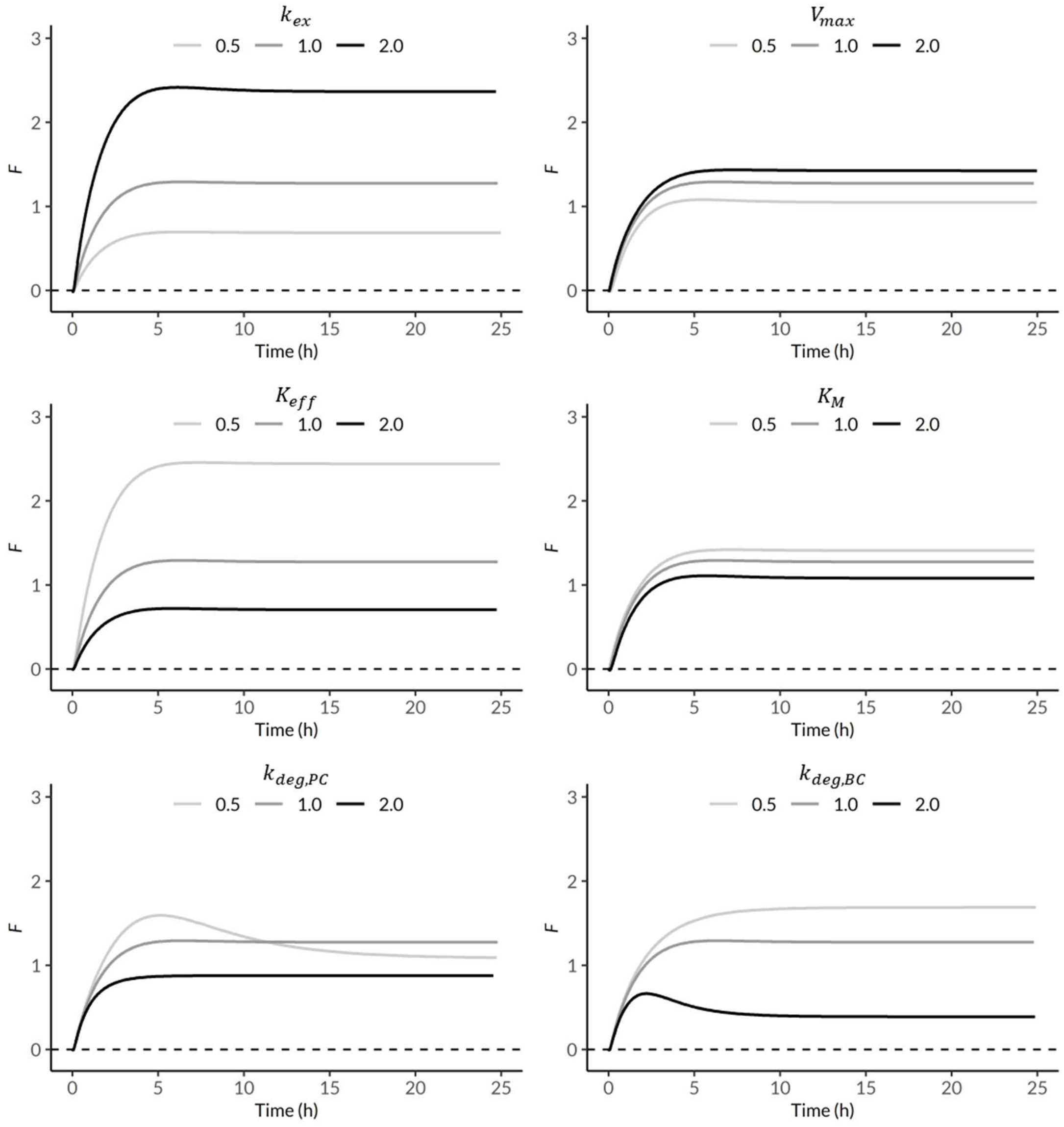

Figure S15. Simulations of varying parameter values. Parameter values were independently adjusted by the specified factors from the model-fitted values for substrate (±)-**1a**.

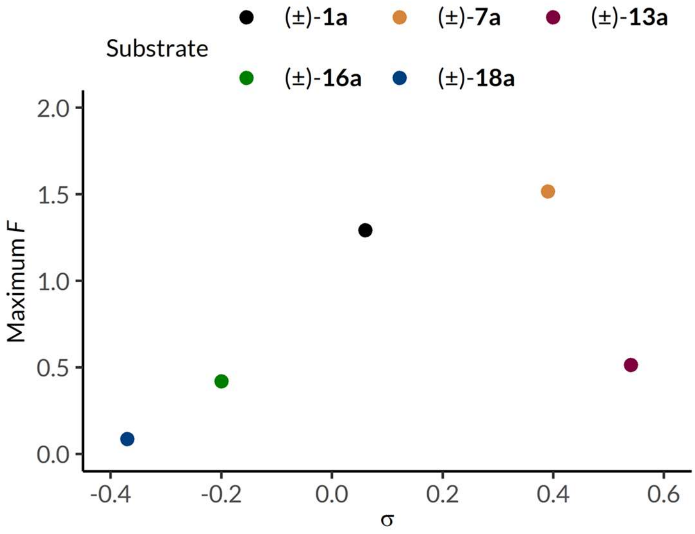

Figure S16. Relationship between maximum fitness score (*F*) over the course of the reaction and electronic properties. Hammett constants (σ) were obtained from Hansch.[23]

8 Characterization of reference compounds and deracemization products

(*S*)-1-(4-Fluorophenyl)ethan-1-ol (1a)

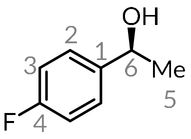

Following **general procedure D**, SAS (30 mol%), *E. coli*/*Gc*APRD (20 mg/mL), NADH (0.8 mM), and (±)-**1a** (1.4 µL, 10 µmol) were added into 1X PBS (pH 7.4, 500 µL, 20 mM) containing 10% (v/v) ^{i}PrOH in a 4 mL glass vial. The mixture was stirred and irradiated by blue LED (405 nm) at RT for 4 h. The reaction yield for **1a** was determined by $^{19}F\{^{1}H\}$ NMR spectroscopy (56%) with **1b** as a side product (17%). The enantiomeric ratio was determined following **general procedure E**. Characterization data is in accordance with literature.[24]

Chiral HPLC: Chiralpak AS-H (hexane : ^{i}PrOH, 93 : 7, flow rate = 1 mL min^{-1}, λ = 210 nm, 30 °C) t_R (*R*): 6.9 min, t_R (*S*): 7.6 min, 4 : 96 er

^{1}H NMR (400 MHz, $CDCl_3$) δ_H 7.39 – 7.30 (2H, m, **3-H**), 7.08 – 6.98 (2H, m, **2-H**), 4.90 (1H, q, *J* 6.4, **6-H**), 1.74 (1H, s, O-**H**), 1.48 (3H, d, *J* 6.4, **5-H**)

$^{13}C\{^{1}H\}$ NMR (126 MHz, $CDCl_3$) δ_C 162.3 (d, *J* 244.9, **C4**), 141.7 (d, *J* 3.2, **C6**), 127.2 (d, *J* 8.1, **C2**), 115.4 (d, *J* 21.4, **C3**), 70.0 (**C6**), 25.5 (**C5**)

$^{19}F\{^{1}H\}$ NMR (376 MHz, $CDCl_3$) δ_F –115.5 (ArF)

1-(4-Fluorophenyl)ethan-1-one (1b)

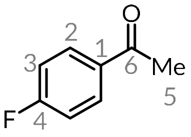

Characterization data is in accordance with literature.[25]

^{1}H NMR (500 MHz, $CDCl_3$) δ_H 7.98 (2H, dd, *J* 8.6, 5.5, **3-H**), 7.13 (2H, t, *J* 8.5, **2-H**), 2.59 (3H, s, **5-H**)

$^{13}C\{^{1}H\}$ NMR (126 MHz, $CDCl_3$) δ_C 196.6 (**C6**), 165.9 (d, *J* 254.6, **C4**), 133.7 (d, *J* 3.1, **C1**), 131.1 (d, *J* 9.3, **C2**), 115.8 (d, *J* 21.8, **C3**), 26.7 (**C5**)

$^{19}F\{^{1}H\}$ NMR (376 MHz, $CDCl_3$) δ_F –105.3 (ArF)

(*S*)-1-Phenylethan-1-ol (2a)

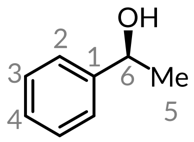

Following **general procedure D**, SAS (30 mol%), *E. coli*/*Gc*APRD (20 mg/mL), NADH (0.8 mM), and (±)-**2a** (1.2 µL, 10 µmol) were added into 1X PBS (pH 7.4, 500 µL, 20 mM) containing 10% (v/v) ^{i}PrOH in a 4 mL glass vial. The mixture was stirred and irradiated by blue LED (405 nm) at RT for 4 h. The reaction yield for **2a** was determined by ^{1}H NMR spectroscopy (43%) with **2b** as a side product (9%). The enantiomeric ratio was determined following **general procedure E**. Characterization data is in accordance with literature.[24]

Chiral HPLC: Chiralcel OD-H (hexane : ^{i}PrOH, 97 : 3, flow rate = 1 mL min^{-1}, λ = 210 nm, 30 °C) t_R (*R*): 13.6 min, t_R (*S*): 16.5 min, 3 : 97 er

^{1}H NMR (500 MHz, CDCl$_3$) δ_H 7.41 – 7.32 (4H, m, **2-H**, **3-H**), 7.31 – 7.25 (1H, m, **4-H**), 4.91 (1H, q, *J* 6.5, **6-H**), 1.76 (1H, s, O-**H**), 1.51 (3H, d, *J* 6.4, **5-H**)

^{13}C{^{1}H} NMR (126 MHz, CDCl$_3$) δ_C 146.0 (**C1**), 128.7 (**C3**), 127.6 (**C4**), 125.5 (**C2**), 70.6 (**C6**), 25.3 (**C5**)

Acetophenone (2b)

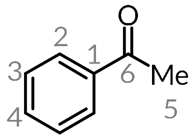

Characterization data is in accordance with literature.[25]

^{1}H NMR (400 MHz, CDCl$_3$) δ_H 8.00 – 7.93 (2H, m, **3-H**), 7.61 – 7.53 (1H, m, **4-H**), 7.52 – 7.42 (2H, m, **2-H**), 2.61 (3H, s, **5-H**)

^{13}C{^{1}H} NMR (126 MHz, CDCl$_3$) δ_C 198.3 (**C6**), 137.3 (**C1**), 133.3 (**C4**), 128.7 (**C2**), 128.5 (**C3**), 26.8 (**C5**)

(*S*)-1-(2-Fluorophenyl)ethan-1-ol (3a)

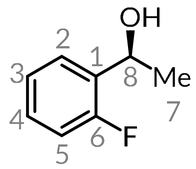

Following **general procedure D**, SAS (30 mol%), *E. coli*/*Gc*APRD (20 mg/mL), NADH (0.8 mM), and (±)-**3a** (1.3 µL, 10 µmol) were added into 1X PBS (pH 7.4, 500 µL, 20 mM) containing 10% (v/v) ^{i}PrOH in a 4 mL glass vial. The mixture was stirred and irradiated by blue LED (405 nm) at RT for 4 h. The reaction yield was determined by ^{19}F{^{1}H} NMR spectroscopy (55%). No side products were observed. The enantiomeric ratio was determined following **general procedure E**. Characterization data is in accordance with literature.[26]

Following **general procedure A**, $NaBH_4$ (170 mg, 4.5 mmol) and **3b** (368 uL, 3.0 mmol) were added into MeOH (3.0 mL) at 0 °C. The mixture was stirred at RT for 2 h, extracted with EtOAc (3 × 10 mL), and then dried *in vacuo* to afford (±)-**3a** as a pale yellow oil (285 mg, 68%).

Chiral HPLC: Chiralcel OD-H (hexane : ^{i}PrOH, 98 : 2, flow rate = 1 mL min^{-1}, λ = 210 nm, 30 °C) t_R (*R*): 10.1 min, t_R (*S*): 10.6 min, 30 : 70 er

^{1}H NMR (500 MHz, $CDCl_3$) δ_H 7.53 – 7.46 (1H, m, **2-H**), 7.25 (1H, d, *J* 10.2, **4-H**), 7.15 (1H, t, *J* 7.6, **3-H**), 7.02 (1H, dd, *J* 10.7, 8.2, **5-H**), 5.20 (1H, dt, *J* 10.7, 5.6, **8-H**), 1.88 (1H, d, *J* 4.3, **O-H**), 1.53 (3H, d, *J* 6.4, **7-H**)

^{13}C{^{1}H} NMR (126 MHz, $CDCl_3$) δ_C 159.8 (d, *J* 245.4, **C6**), 132.6 (d, *J* 13.2, **C1**), 128.8 (d, *J* 8.3, **C4**), 126.6 (d, *J* 4.6, **C2**), 124.3 (d, *J* 3.5, **C3**), 115.3 (d, *J* 21.8, **C5**), 64.6 (d, *J* 3.1, **C8**), 24.0 (**C7**)

^{19}F{^{1}H} NMR (470 MHz, $CDCl_3$) δ_F –120.1 (Ar**F**)

(*S*)-1-(4-Chlorophenyl)ethan-1-ol (4a)

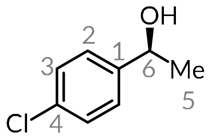

Following **general procedure D**, SAS (30 mol%), *E. coli*/*Gc*APRD (20 mg/mL), NADH (0.8 mM), and (±)-**4a** (1.3 µL, 10 µmol) were added into 1X PBS (pH 7.4, 500 µL, 20 mM) containing 10% (v/v) ^{i}PrOH in a 4 mL glass vial. The mixture was stirred and irradiated by blue LED (405 nm) at RT for 4 h. The reaction yield for **4a** was determined by ^{1}H NMR spectroscopy (40%) with **4b** as a side product (10%).

For the 0.1 mmol scale reaction, SAS (30 mol%), NADH (0.8 mM), and (±)-**4a** (13.4 µL, 0.1 mmol) were added into 1X PBS (pH 7.4, 5 mL, 20 mM) containing 10% (v/v) ^{i}PrOH in a 7 mL Schlenk tube connected to a CondenSyn waterless air condenser (Asynt) with a stopper to ensure a headspace volume of at least 3 times the reaction volume. The mixture was stirred and irradiated by blue LED (405 nm) at RT for 4 h, then mixed with ice-cold CH_3CN (10 mL) and MeOH (10 mL) in a 50 mL centrifuge tube and incubated at –20 °C for 30 min. Precipitated biological materials were removed by centrifugation (10 min, 4,800 × *g*) at 4 °C. The supernatant was concentrated *in vacuo*, extracted with CH_2Cl_2 (7 × 10 mL), washed with brine, and then purified by flash column chromatography (pentanes : Et_2O, 92 : 8 to 80 : 20). Fractions containing **4a** were dried *in vacuo* to afford (*S*)-**4a** as a pale yellow solid (9.2 mg, 59%).

Enantiomeric ratios were determined following **general procedure E**. Characterization data is in accordance with literature.[26]

Following **general procedure A**, $NaBH_4$ (170 mg, 4.5 mmol) and **4b** (389 uL, 3.0 mmol) were added into MeOH (3.0 mL) at 0 °C. The mixture was stirred at RT for 2 h, extracted with EtOAc (3 × 10 mL), and then dried *in vacuo* to afford (±)-**4a** as a pale yellow oil (367 mg, 78%).

$[\alpha]_D^{20}$ –13.9 (*c* 0.27, $CHCl_3$) {Lit.[27] $[\alpha]_D^{25}$ –17.2 (*c* 1.00, $CHCl_3$)}

Chiral HPLC: Chiralcel OJ-H (hexane : ^{i}PrOH, 93 : 7, flow rate = 1 mL min^{-1}, λ = 230 nm, 30 °C) t_R (*S*): 8.6 min, t_R (*R*): 9.2 min, 98 : 2 er

^{1}H NMR (400 MHz, $CDCl_3$) δ_H 7.31 (4H, m, **2-H, 3-H**), 4.94 – 4.84 (1H, m, **6-H**), 1.79 (1H, d, *J* 3.6, O-**H**), 1.48 (3H, app. d, *J* 6.5, **5-H**)

$^{13}C\{^{1}H\}$ NMR (126 MHz, $CDCl_3$) δ_C 144.4 (**C1**), 133.2 (**C4**), 128.8 (**C3**), 126.9 (**C2**), 69.9 (**C6**), 25.4 (**C5**)

1-(4-Chlorophenyl)ethan-1-one (4b)

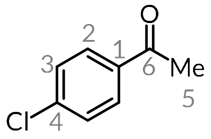

Characterization data is in accordance with literature.[28]

^{1}H NMR (500 MHz, $CDCl_3$) δ_H 7.93 – 7.86 (2H, m, **2-H**), 7.47 – 7.41 (2H, m, **3-H**), 2.59 (3H, s, **5-H**)

$^{13}C\{^{1}H\}$ NMR (126 MHz, $CDCl_3$) δ_C 197.0 (**C6**), 139.7 (**C4**), 135.6 (**C1**), 129.9 (**C2**), 129.0 (**C3**), 26.7 (**C5**)

1-(3-Chlorophenyl)ethan-1-ol (5a)

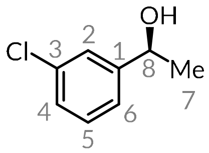

Following **general procedure D**, SAS (30 mol%), *E. coli*/*Gc*APRD (20 mg/mL), NADH (0.8 mM), and (±)-**5a** (1.3 µL, 10 µmol) were added into 1X PBS (pH 7.4, 500 µL, 20 mM) containing 10% (v/v) ^{i}PrOH in a 4 mL glass vial. The mixture was stirred and irradiated by blue LED (405 nm) at RT for 4 h. The reaction yield for **5a** was determined by ^{1}H NMR spectroscopy (41%) with **5b** as a side product (3%). The enantiomeric ratio was determined following **general procedure E**. Characterization data is in accordance with literature.[29]

Following **general procedure A**, $NaBH_4$ (57 mg, 1.5 mmol) and **5b** (128 uL, 1.0 mmol) were added into MeOH (1.0 mL) at 0 °C. The mixture was stirred at RT for 2 h, extracted with EtOAc (3 × 10 mL), purified by flash column chromatography (*n*-hexane : EtOAc, 96 : 4 to 90 : 10), and then dried *in vacuo* to afford (±)-**5a** as a white solid (100 mg, 64%).

Chiral HPLC: Chiralcel OD-H (hexane : ^{i}PrOH, 93 : 7, flow rate = 1 mL min^{-1}, λ = 230 nm, 30 °C) t_R (*S*): 7.6 min, t_R (*R*): 8.5 min, 94 : 6 er

^{1}H NMR (500 MHz, $CDCl_3$) δ_H 7.38 (1H, t, *J* 1.9, **2-H**), 7.31 – 7.21 (3H, m, **4-H**, **5-H, 6-H**), 4.88 (1H, q, *J* 6.1, **8-H**), 1.81 (1H, d, *J* 3.3, O-**H**), 1.49 (3H, d, *J* 6.5, **7-H**)

^{13}C{^{1}H} NMR (126 MHz, $CDCl_3$) δ_C 148.0 (**C1**), 134.5 (**C3**), 129.9 (**C5**), 127.7 (**C4**), 125.8 (**C2**), 123.7 (**C6**), 70.0 (**C8**), 25.4 (**C7**)

1-(3-Chlorophenyl)ethan-1-one (5b)

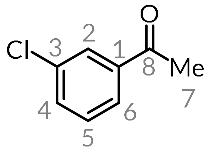

Characterization data is in accordance with literature.[30]

^{1}H NMR (500 MHz, $CDCl_3$) δ_H 7.92 (1H, t, *J* 1.9, **2-H**), 7.83 (1H, dt, *J* 7.8, 1.4, **6-H**), 7.53 (1H, ddd, *J* 8.0, 2.2, 1.1, **4-H**), 7.41 (1H, t, *J* 7.9, **5-H**), 2.59 (3H, s, **7-H**)

^{13}C{^{1}H} NMR (126 MHz, $CDCl_3$) δ_C 196.9 (**C8**), 138.7 (**C1**), 135.1 (**C3**), 133.2 (**C4**), 130.1 (**C5**), 128.5 (**C2**), 126.5 (**C6**), 26.8 (**C7**)

(*S*)-1-(2-Bromophenyl)ethan-1-ol (6a)

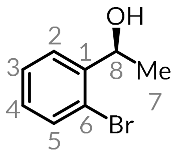

Following **general procedure D**, SAS (30 mol%), *E. coli*/*Gc*APRD (20 mg/mL), NADH (0.8 mM), and (±)-**6a** (1.4 µL, 10 µmol) were added into 1X PBS (pH 7.4, 500 µL, 20 mM) containing 10% (v/v) ^{i}PrOH in a 4 mL glass vial. The mixture was stirred and irradiated by blue LED (405 nm) at RT for 4 h. The reaction yield for **6a** was determined by ^{1}H NMR spectroscopy (45%) with an unidentified side product (8%). The enantiomeric ratio was determined following **general procedure E**. Characterization data is in accordance with literature.[26]

Following **general procedure A**, $NaBH_4$ (170 mg, 4.5 mmol) and 1-(2-bromophenyl)ethan-1-one (404 uL, 3.0 mmol) were added into MeOH (3.0 mL) at 0 °C. The mixture was stirred at RT for 2 h, extracted with EtOAc (3 × 10 mL), and then dried *in vacuo* to afford (±)-**6a** as a pale yellow oil (433 mg, 72%).

Chiral HPLC: Chiralcel OD-H (hexane : ^{i}PrOH, 98 : 2, flow rate = 1 mL min^{-1}, λ = 230 nm, 30 °C) t_R (*R*): 11.9 min, t_R (*S*): 13.0 min, 45 : 55 er

^{1}H NMR (500 MHz, $CDCl_3$) δ_H 7.60 (1H, dd, *J* 7.8, 1.7, **2-H**), 7.52 (1H, dd, *J* 8.0, 1.3, **5-H**), 7.35 (1H, td, *J* 7.6, 1.3, **3-H**), 7.13 (1H, td, *J* 7.7, 1.8, **4-H**), 5.25 (1H, qd, *J* 6.4, 3.5, **8-H**), 1.99 (1H, d, *J* 3.6, O-**H**), 1.49 (3H, d, *J* 6.4, **7-H**)

^{13}C{^{1}H} NMR (126 MHz, $CDCl_3$) δ_C 144.7 (**C1**), 132.8 (**C5**), 128.9 (**C4**), 128.0 (**C3**), 126.8 (**C2**), 121.9 (**C6**), 69.4 (**C8**), 23.7 (**C7**)

(*S*)-1-(3-Bromophenyl)ethan-1-ol (7a)

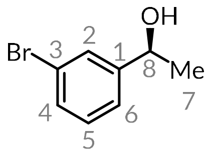

Following **general procedure D**, SAS (30 mol%), *E. coli*/*Gc*APRD (20 mg/mL), NADH (0.8 mM), and (±)-**7a** (1.4 µL, 10 µmol) were added into 1X PBS (pH 7.4, 500 µL, 20 mM) containing 10% (v/v) ^{i}PrOH in a 4 mL glass vial. The mixture was stirred and irradiated by blue LED (405 nm) at RT for 4 h. The reaction yield for **7a** was determined by ^{1}H NMR spectroscopy (20%). No side products were observed. The enantiomeric ratio was determined following **general procedure E**. Characterization data is in accordance with literature.[26]

Following **general procedure A**, $NaBH_4$ (170 mg, 4.5 mmol) and 1-(3-bromophenyl)ethan-1-one (398 uL, 3.0 mmol) were added into MeOH (3.0 mL) at 0 °C. The mixture was stirred at RT for 2 h, extracted with EtOAc (3 × 10 mL), and then dried *in vacuo* to afford (±)-**7a** as a brown oil (368 mg, 61%).

Chiral HPLC: Chiralcel OD-H (hexane : ^{i}PrOH, 93 : 7, flow rate = 1 mL min^{-1}, λ = 270 nm, 30 °C) t_R (*S*): 7.3 min, t_R (*R*): 7.9 min, 91 : 9 er

^{1}H NMR (500 MHz, $CDCl_3$) δ_H 7.54 (1H, t, *J* 1.8, **2-H**), 7.40 (1H, ddd, *J* 7.8, 2.0, 1.1, **4-H**), 7.32 – 7.26 (1H, m, **6-H**), 7.22 (1H, t, *J* 7.8, **5-H**), 4.87 (1H, qd, *J* 6.4, 2.9, **8-H**), 1.83 (1H, d, *J* 3.4, O-**H**), 1.49 (3H, d, *J* 6.4, **7-H**)

^{13}C{^{1}H} NMR (126 MHz, $CDCl_3$) δ_C 148.3 (**C1**), 130.6 (**C4**), 130.3 (**C5**), 128.7 (**C2**), 124.2 (**C6**), 122.8 (**C3**), 69.9 (**C8**), 25.4 (**C7**)

(*S*)-1-(4-Bromophenyl)ethan-1-ol (8a)

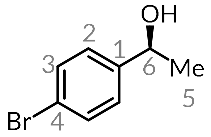

Following **general procedure D**, SAS (30 mol%), *E. coli*/*Gc*APRD (20 mg/mL), NADH (0.8 mM), and (±)-**8a** (1.4 µL, 10 µmol) were added into 1X PBS (pH 7.4, 500 µL, 20 mM) containing 10% (v/v) ^{i}PrOH in a 4 mL glass vial. The mixture was stirred and irradiated by blue LED (405 nm) at RT for 4 h. The reaction yield for **8a** was determined by ^{1}H NMR spectroscopy (41%) with **8b** as a side product (11%). The enantiomeric ratio was determined following **general procedure E**. Characterization data is in accordance with literature.[26]

Following **general procedure A**, $NaBH_4$ (170 mg, 4.5 mmol) and **8b** (597 mg, 3.0 mmol) were added into MeOH (3.0 mL) at 0 °C. The mixture was stirred at RT for 2 h, extracted with EtOAc (3 × 10 mL), and then dried *in vacuo* to afford (±)-**8a** as a white solid (441 mg, 73%).

Chiral HPLC: Chiralcel OD-H (hexane : ^{i}PrOH, 93 : 7, flow rate = 1 mL min^{-1}, λ = 254 nm, 30 °C) t_R (*S*): 7.3 min, t_R (*R*): 8.0 min, 98 : 2 er

^{1}H NMR (500 MHz, $CDCl_3$) δ_H 7.47 (2H, m, **3-H**), 7.25 (2H, m, **2-H**), 4.87 (1H, qd, *J* 6.4, 3.6, **6-H**), 1.77 (1H, d, *J* 3.7, O-**H**), 1.47 (3H, d, *J* 6.5, **5-H**)

^{13}C{^{1}H} NMR (126 MHz, $CDCl_3$) δ_C 144.9 (**C1**), 131.7 (**C3**), 127.3 (**C2**), 121.3 (**C4**), 70.0 (**C6**), 25.4 (**C5**)

1-(4-Bromophenyl)ethan-1-one (8b)

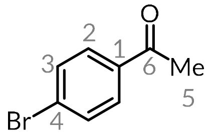

Characterization data is in accordance with literature.[25]

^{1}H NMR (500 MHz, $CDCl_3$) δ_H 7.85 – 7.79 (2H, m, **2-H**), 7.64 – 7.58 (2H, m, **3-H**), 2.59 (3H, s, **5-H**)

^{13}C{^{1}H} NMR (126 MHz, $CDCl_3$) δ_C 197.2 (**C6**), 136.0 (**C1**), 132.1 (**C3**), 130.0 (**C2**), 128.5 (**C4**), 26.7 (**C5**)

(*S*)-1-(2-Iodophenyl)ethan-1-ol (9a)

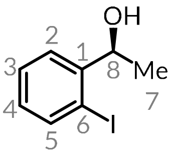

Following **general procedure D**, SAS (30 mol%), *E. coli*/*Gc*APRD (20 mg/mL), NADH (0.8 mM), and (±)-**9a** (1.4 µL, 10 µmol) were added into 1X PBS (pH 7.4, 500 µL, 20 mM) containing 10% (v/v) ^{i}PrOH in a 4 mL glass vial. The mixture was stirred and irradiated by blue LED (405 nm) at RT for 4 h. The reaction yield for **9a** was determined by ^{1}H NMR spectroscopy (37%) with **9b** as a side product (2%). The enantiomeric ratio was determined following **general procedure E**. Characterization data is in accordance with literature.[31]

Following **general procedure A**, $NaBH_4$ (57 mg, 1.5 mmol) and **9b** (142 uL, 1.0 mmol) were added into MeOH (1.0 mL) at 0 °C. The mixture was stirred at RT for 2 h, extracted with EtOAc (3 × 10 mL), and then dried *in vacuo* to afford (±)-**9a** as a pale yellow oil (161 mg, 65%).

Chiral HPLC: Chiralcel OD-H (hexane : ^{i}PrOH, 99 : 1, flow rate = 1 mL min^{-1}, λ = 254 nm, 30 °C) t_R (*R*): 24.3 min, t_R (*S*): 27.8 min, 45 : 55 er

^{1}H NMR (500 MHz, $CDCl_3$) δ_H 7.80 (1H, app. d, *J* 7.9, **5-H**), 7.57 (1H, dd, *J* 7.7, 1.8, **2-H**), 7.38 (1H, t, *J* 7.5, **3-H**), 7.26 (1H, s), 6.97 (1H, td, *J* 7.6, 1.8, **4-H**), 5.08 (1H, qd, *J* 6.3, 3.2, **8-H**), 1.94 (1H, d, *J* 3.3, O-**H**), 1.47 (3H, d, *J* 6.3, **7-H**)

^{13}C{^{1}H} NMR (126 MHz, $CDCl_3$) δ_C 147.6 (**C1**), 139.5 (**C5**), 129.3 (**C4**), 128.9 (**C3**), 126.5 (**C2**), 97.4 (**C6**), 73.9 (**C8**), 23.9 (**C7**)

1-(2-Iodophenyl)ethan-1-one (9b)

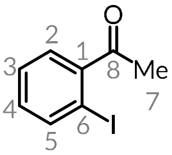

Characterization data is in accordance with literature.[32]

^{1}H NMR (500 MHz, $CDCl_3$) δ_H 7.94 (1H, dd, *J* 8.0, 1.1, **5-H**), 7.46 (1H, dd, *J* 7.7, 1.8, **2-H**), 7.41 (1H, td, *J* 7.5, 1.2, **3-H**), 7.12 (1H, ddd, *J* 7.9, 7.3, 1.8, **4-H**), 2.61 (3H, s, **7-H**)

^{13}C{^{1}H} NMR (126 MHz, $CDCl_3$) δ_C 202.0 (**C8**), 144.2 (**C1**), 141.0 (**C5**), 132.0 (**C4**), 128.5 (**C2**), 128.2 (**C3**), 91.1 (**C6**), 29.6 (**C7**)

(*S*)-1-(4-Iodophenyl)ethan-1-ol (10a)

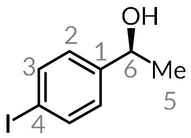

Following **general procedure D**, SAS (30 mol%), *E. coli*/*Gc*APRD (20 mg/mL), NADH (0.8 mM), and (±)-**10a** (1.5 μL, 10 μmol) were added into 1X PBS (pH 7.4, 500 μL, 20 mM) containing 10% (v/v) ^{i}PrOH in a 4 mL glass vial. The mixture was stirred and irradiated by blue LED (405 nm) at RT for 4 h. The reaction yield for **10a** was determined by ^{1}H NMR spectroscopy (48%) with **10b** as a side product (6%). The enantiomeric ratio was determined following **general procedure E**. Characterization data is in accordance with literature.[33]

Following **general procedure A**, $NaBH_4$ (170 mg, 4.5 mmol) and **10b** (738 mg, 3.0 mmol) were added into MeOH (3.0 mL) at 0 °C. The mixture was stirred at RT for 2 h, extracted with EtOAc (3 × 10 mL), and then dried *in vacuo* to afford (±)-**10a** as a brown solid (498 mg, 67%).

Chiral HPLC: Chiralcel OJ-H (hexane : ^{i}PrOH, 95 : 5, flow rate = 1 mL min^{-1}, λ = 254 nm, 30 °C) t_R (*S*): 12.7 min, t_R (*R*): 13.5 min, 58 : 42 er

^{1}H NMR (500 MHz, $CDCl_3$) δ_H 7.70 – 7.64 (2H, m, **3-H**), 7.16 – 7.09 (2H, m, **2-H**), 4.85 (1H, qd, *J* 6.4, 2.4, **6-H**), 1.83 (1H, d, *J* 2.9, O-**H**), 1.47 (3H, d, *J* 6.5, **5-H**)

$^{13}C\{^1H\}$ NMR (126 MHz, $CDCl_3$) δ_C 145.6 (**C1**), 137.7 (**C3**), 127.6 (**C2**), 92.9 (**C4**), 70.0 (**C6**), 25.4 (**C5**)

1-(4-Iodophenyl)ethan-1-one (10b)

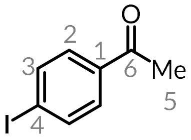

Characterization data is in accordance with literature.[34]

^{1}H NMR (500 MHz, $CDCl_3$) δ_H 7.86 – 7.80 (2H, m, **3-H**), 7.70 – 7.63 (2H, m, **2-H**), 2.57 (3H, s, **5-H**)

$^{13}C\{^1H\}$ NMR (126 MHz, $CDCl_3$) δ_C 197.5 (**C6**), 138.1 (**C3**), 136.5 (**C1**), 129.9 (**C2**), 101.2 (**C4**), 26.6 (**C5**)

(*S*)-1-(3-Methoxyphenyl)ethan-1-ol (11a)

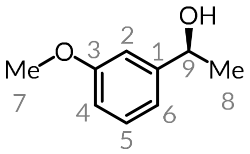

Following **general procedure D**, SAS (30 mol%), *E. coli*/*Gc*APRD (20 mg/mL), NADH (0.8 mM), and (±)-**11a** (1.4 μL, 10 μmol) were added into 1X PBS (pH 7.4, 500 μL, 20 mM) containing 10% (v/v) ^{i}PrOH in a 4 mL glass vial. The mixture was stirred and irradiated by blue LED (405 nm) at RT for 4 h. The reaction yield for **11a** was determined by ^{1}H NMR spectroscopy (50%) with **11b** as a side product (4%). The enantiomeric ratio was determined following **general procedure E**. Characterization data is in accordance with literature.[35]

Chiral HPLC: Chiralcel OD-H (hexane : ^{i}PrOH, 95 : 5, flow rate = 1 mL min^{-1}, λ = 230 nm, 30 °C) t_R (*R*): 13.3 min, t_R (*S*): 15.4 min, 47 : 53 er

^{1}H NMR (400 MHz, CDCl$_3$) δ_H 7.27 (1H, t, *J* 8.1, **5-H**), 6.99 – 6.91 (2H, m, **2-H**, **6-H**), 6.82 (1H, ddd, *J* 8.2, 2.6, 1.1, **4-H**), 4.88 (1H, qd, *J* 6.4, 2.8, **9-H**), 3.82 (3H, s, **7-H**), 1.81 (1H, d, *J* 3.3, O-**H**), 1.50 (3H, d, *J* 6.4, **8-H**)

^{13}C{^{1}H} NMR (126 MHz, CDCl$_3$) δ_C 160.0 (**C3**), 147.7 (**C1**), 129.7 (**C5**), 117.8 (**C6**), 113.1 (**C4**), 111.0 (**C2**), 70.5 (**C9**), 55.4 (**C7**), 25.3 (**C8**)

1-(3-Methoxyphenyl)ethan-1-one (11b)

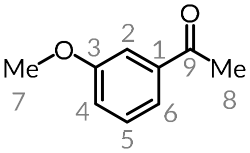

Characterization data is in accordance with literature.[36]

^{1}H NMR (400 MHz, CDCl$_3$) δ_H 7.54 (1H, ddd, *J* 7.6, 1.6, 1.0, **6-H**), 7.49 (1H, dd, *J* 2.7, 1.5, **5-H**), 7.37 (1H, ddd, *J* 8.1, 7.6, 0.4, **2-H**), 7.11 (1H, ddd, *J* 8.2, 2.7, 1.0, **4-H**), 3.86 (3H, s, **7-H**), 2.60 (3H, s, **8-H**)

^{13}C{^{1}H} NMR (126 MHz, CDCl$_3$) δ_C 198.1 (**C9**), 160.0 (**C3**), 138.7 (**C1**), 129.7 (**C5**), 121.3 (**C6**), 119.8 (**C4**), 112.5 (**C2**), 55.6 (**C7**), 26.9 (**C8**)

(*S*)-1-(4-Methoxyphenyl)ethan-1-ol (12a)

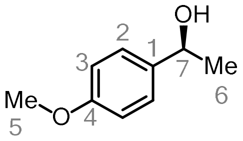

Following **general procedure D**, SAS (30 mol%), *E. coli*/*Gc*APRD (20 mg/mL), NADH (0.8 mM), and (±)-**12a** (1.4 µL, 10 µmol) were added into 1X PBS (pH 7.4, 500 µL, 20 mM) containing 10% (v/v) ^{i}PrOH in a 4 mL glass vial. The mixture was stirred and irradiated by blue LED (405 nm) at RT for 4 h. The reaction yield for **12a** was determined by ^{1}H NMR spectroscopy (32%) with **12b** as a side product (21%). The enantiomeric ratio was determined following **general procedure E**. Characterization data is in accordance with literature.[24]

Chiral HPLC: Chiralcel OD-H (hexane : ^{i}PrOH, 95 : 5, flow rate = 1 mL min^{-1}, λ = 210 nm, 30 °C) t_R (*S*): 11.7 min, t_R (*R*): 13.1 min, 61 : 39 er

^{1}H NMR (500 MHz, $CDCl_3$) δ_H 7.34 – 7.28 (2H, m, **3-H**), 6.92 – 6.86 (2H, m, **2-H**), 4.87 (1H, qd, *J* 6.4, 3.4, **7-H**), 3.81 (3H, s, **5-H**), 1.69 (1H, d, *J* 3.5, O-**H**), 1.49 (3H, d, *J* 6.4, **6-H**)

^{13}C{^{1}H} NMR (126 MHz, $CDCl_3$) δ_C 159.2 (**C4**), 138.1 (**C1**), 126.8 (**C2**), 114.0 (**C3**), 70.2 (**C7**), 55.5 (**C5**), 25.2 (**C6**)

1-(4-Methoxyphenyl)ethan-1-one (12b)

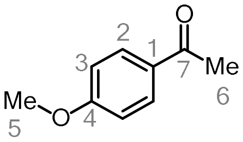

Characterization data is in accordance with literature.[25]

^{1}H NMR (400 MHz, $CDCl_3$) δ_H 7.98 – 7.90 (2H, m, **3-H**), 6.97 – 6.89 (2H, m, **2-H**), 3.87 (3H, s, **5-H**), 2.56 (3H, s, **6-H**)

^{13}C{^{1}H} NMR (126 MHz, $CDCl_3$) δ_C 196.9 (**C7**), 163.6 (**C4**), 130.7 (**C2**), 130.5 (**C1**), 113.8 (**C3**), 55.6 (**C5**), 26.5 (**C6**)

(*S*)-1-(4-(Trifluoromethyl)phenyl)ethan-1-ol (13a)

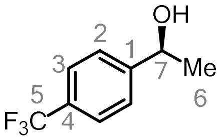

Following **general procedure D**, SAS (30 mol%), *E. coli*/*Gc*APRD (20 mg/mL), NADH (0.8 mM), and (±)-**13a** (1.5 µL, 10 µmol) were added into 1X PBS (pH 7.4, 500 µL, 20 mM) containing 10% (v/v) ^{i}PrOH in a 4 mL glass vial. The mixture was stirred and irradiated by blue LED (405 nm) at RT for 4 h. The reaction yield for **13a** was determined by ^{1}H NMR spectroscopy (69%) with **13b** as a side product (4%). The enantiomeric ratio was determined following **general procedure E**. Characterization data is in accordance with literature.[35]

Chiral HPLC: Chiralcel OJ-H (hexane : ^{i}PrOH, 97 : 3, flow rate = 1 mL min^{-1}, λ = 210 nm, 30 °C) t_R (*S*): 9.3 min, t_R (*R*): 10.0 min, 76 : 24 er

^{1}H NMR (400 MHz, CDCl$_3$) δ_H 7.64 – 7.58 (2H, m, **3-H**), 7.49 (2H, m, **2-H**), 4.97 (1H, qd, *J* 6.5, 3.7, **7-H**), 1.88 (1H, d, *J* 3.7, O-**H**), 1.51 (3H, d, *J* 6.5, **6-H**)

^{13}C{^{1}H} NMR (126 MHz, CDCl$_3$) δ_C 149.8 (**C1**), 129.8 (q, *J* 32.3, **C4**), 125.8 (**C2**), 125.6 (q, *J* 3.9, **C3**), 124.3 (q, *J* 272.0, **C5**), 70.0 (**C7**), 25.6 (**C6**)

^{19}F{^{1}H} NMR (470 MHz, CDCl$_3$) δ_F –62.5 (CF_3)

1-(4-(Trifluoromethyl)phenyl)ethan-1-one (13b)

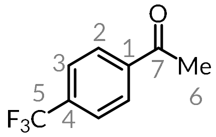

Characterization data is in accordance with literature.[37]

^{1}H NMR (500 MHz, CDCl$_3$) δ_H 8.09 – 8.04 (2H, m, **3-H**), 7.76 – 7.71 (2H, m, **2-H**), 2.65 (3H, s, **6-H**)

^{13}C{^{1}H} NMR (126 MHz, CDCl$_3$) δ_C 197.1 (**C7**), 139.8 (**C1**), 134.6 (q, *J* 32.7, **C4**), 128.8 (**C2**), 125.8 (q, *J* 3.8, **C3**), 123.7 (q, *J* 272.5, **C5**), 26.9 (**C6**)

^{19}F{^{1}H} NMR (470 MHz, CDCl$_3$) δ_F –63.1 (CF_3)

(*S*)-1-(4-Aminophenyl)ethan-1-ol (14a)

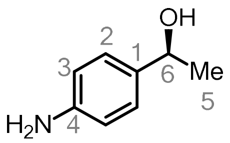

Following **general procedure D**, SAS (30 mol%), *E. coli*/*Gc*APRD (20 mg/mL), NADH (0.8 mM), and (±)-**14a** (1.4 mg, 10 μmol) were added into 1X PBS (pH 7.4, 500 μL, 20 mM) containing 10% (v/v) ^{i}PrOH in a 4 mL glass vial. The mixture was stirred and irradiated by blue LED (405 nm) at RT for 4 h. The reaction yield for **14a** was determined by ^{1}H NMR spectroscopy (43%) with **14b** as a side product (36%). The enantiomeric ratio was determined following **general procedure E**. Characterization data is in accordance with literature.[38]

Following **general procedure A**, $NaBH_4$ (170 mg, 4.5 mmol) and **14b** (406 mg, 3.0 mmol) were added into MeOH (3.0 mL) at 0 °C. The mixture was stirred at RT for 2 h, extracted with $CHCl_3$: ^{i}PrOH (3 : 1, 5 × 10 mL), purified by flash column chromatography (*n*-hexane : EtOAc, 20 : 80), and then dried *in vacuo* to afford (±)-**14a** as a pale yellow solid (316 mg, 77%).

Chiral HPLC: Chiralpak AD-H (hexane : ^{i}PrOH, 85 : 15, flow rate = 1 mL min^{-1}, λ = 254 nm, 30 °C) t_R (*R*): 15.3 min, t_R (*S*): 16.7 min, 33 : 67 er

^{1}H NMR (500 MHz, $CDCl_3$) δ_H 7.20 – 7.15 (2H, m, **3-H**), 6.70 – 6.64 (2H, m, **2-H**), 4.80 (1H, qd, *J* 6.4, 3.3, **6-H**), 3.65 (2H, s, N-**H**), 1.65 (1H, d, *J* 3.5, O-**H**), 1.47 (3H, d, *J* 6.5, **5-H**)

^{13}C{^{1}H} NMR (126 MHz, $CDCl_3$) δ_C 146.0 (**C4**), 136.1 (**C1**), 126.8 (**C2**), 115.2 (**C3**), 70.3 (**C6**), 25.0 (**C5**)

1-(4-Aminophenyl)ethan-1-one (14b)

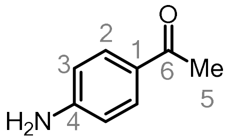

Characterization data is in accordance with literature.[39]

^{1}H NMR (500 MHz, $CDCl_3$) δ_H 7.84 – 7.78 (2H, m, **2-H**), 6.68 – 6.62 (2H, m, **3-H**), 4.10 (2H, s, N-**H**), 2.51 (3H, s, **5-H**)

^{13}C{^{1}H} NMR (126 MHz, $CDCl_3$) δ_C 196.6 (**C6**), 151.2 (**C4**), 131.0 (**C2**), 128.1 (**C1**), 113.9 (**C3**), 26.2 (**C5**)

(*S*)-1-(*p*-Tolyl)ethan-1-ol (15a)

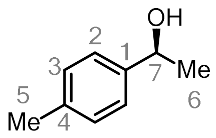

Following **general procedure D**, SAS (30 mol%), *E. coli*/*Gc*APRD (20 mg/mL), NADH (0.8 mM), and (±)-**15a** (1.4 μL, 10 μmol) were added into 1X PBS (pH 7.4, 500 μL, 20 mM) containing 10% (v/v) ^{i}PrOH in a 4 mL glass vial. The mixture was stirred and irradiated by blue LED (405 nm) at RT for 4 h. The reaction yield for **15a** was determined by ^{1}H NMR spectroscopy (28%) with side products **15b** (10%) and an unidentified aldehyde (5%). The enantiomeric ratio was determined following **general procedure E**. Characterization data is in accordance with literature.[38]

Following **general procedure A**, $NaBH_4$ (170 mg, 4.5 mmol) and **15b** (401 uL, 3.0 mmol) were added into MeOH (3.0 mL) at 0 °C. The mixture was stirred at RT for 2 h, extracted with EtOAc (3 × 10 mL), purified by flash column chromatography (*n*-hexane : EtOAc, 50 : 50), and then dried *in vacuo* to afford (±)-**15a** as a pale yellow oil (276 mg, 68%).

Chiral HPLC: Chiralcel OJ-H (hexane : ^{i}PrOH, 95 : 5, flow rate = 1 mL min^{-1}, λ = 210 nm, 30 °C) t_R (*S*): 10.3 min, t_R (*R*): 11.7 min, 92 : 8 er

^{1}H NMR (500 MHz, $CDCl_3$) δ_H 7.27 (2H, m, **2-H**), 7.17 (2H, d, *J* 7.9, **3-H**), 4.88 (1H, qd, *J* 6.4, 3.4, **7-H**), 2.35 (3H, s, **5-H**), 1.72 (1H, d, *J* 3.5, O-**H**), 1.49 (3H, d, *J* 6.4, **6-H**)

$^{13}C\{^1H\}$ NMR (126 MHz, $CDCl_3$) δ_C 143.0 (**C1**), 137.3 (**C4**), 129.3 (**C3**), 125.5 (**C2**), 70.4 (**C7**), 25.2 (**C6**), 21.2 (**C5**)

1-(*p*-Tolyl)ethan-1-one (15b)

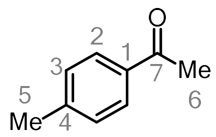

Characterization data is in accordance with literature.[36]

^{1}H NMR (500 MHz, $CDCl_3$) δ_H 7.88 – 7.83 (2H, m, **3-H**), 7.24 (1H, app. s, **2-H**), 2.57 (3H, s, **6-H**), 2.41 (3H, s, **5-H**)

$^{13}C\{^1H\}$ NMR (126 MHz, $CDCl_3$) δ_C 198.0 (**C7**), 144.0 (**C4**), 134.8 (**C1**), 129.4 (**C3**), 128.6 (**C2**), 26.6 (**C6**), 21.7 (**C5**)

(*S*)-1-(4-(*tert*-Butyl)phenyl)ethan-1-ol (16a)

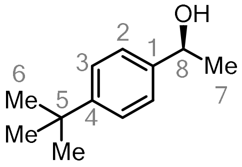

Following **general procedure D**, SAS (30 mol%), *E. coli*/*Gc*APRD (20 mg/mL), NADH (0.8 mM), and (±)-**16a** (1.8 mg, 10 μmol) were added into 1X PBS (pH 7.4, 500 μL, 20 mM) containing 10% (v/v) ^{i}PrOH in a 4 mL glass vial. The mixture was stirred and irradiated by blue LED (405 nm) at RT for 4 h. The reaction yield for **16a** was determined by ^{1}H NMR spectroscopy (26%) with **16b** as a side product (36%). The enantiomeric ratio was determined following **general procedure E**. Characterization data is in accordance with literature.[38]

Following **general procedure A**, $NaBH_4$ (57 mg, 1.5 mmol) and **16b** (183 uL, 1.0 mmol) were added into MeOH (1.0 mL) at 0 °C. The mixture was stirred at RT for 2 h, extracted with EtOAc (3 × 10 mL), and then dried *in vacuo* to afford (±)-**16a** as a white solid (62 mg, 35%).

Chiral HPLC: Chiralpak IC (hexane : ^{i}PrOH, 99 : 1, flow rate = 1 mL min^{-1}, λ = 210 nm, 30 °C) t_R (*S*): 19.0 min, t_R (*R*): 22.5 min, 90 : 10 er

^{1}H NMR (400 MHz, $CDCl_3$) δ_H 7.42 – 7.29 (4H, m, **2-H**, **3-H**), 4.89 (1H, qd, *J* 6.5, 3.6, **8-H**), 1.72 (1H, d, *J* 3.6, O-**H**), 1.50 (3H, d, *J* 6.5, **7-H**), 1.32 (9H, s, **6-H**)

^{13}C{^{1}H} NMR (126 MHz, $CDCl_3$) δ_C 150.6 (**C4**), 142.9 (**C1**), 125.6 (**C2**), 125.3 (**C3**), 70.4 (**C8**), 34.7 (**C5**), 31.5 (**C6**), 25.1 (**C7**)

1-(4-(*tert*-Butyl)phenyl)ethan-1-one (16b)

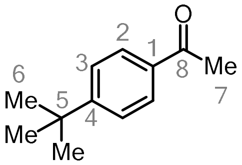

Characterization data is in accordance with literature.[36]

^{1}H NMR (500 MHz, $CDCl_3$) δ_H 7.92 – 7.88 (2H, m, **3-H**), 7.50 – 7.46 (2H, m, **2-H**), 2.59 (3H, s, **7-H**), 1.35 (9H, s, **6-H**)

^{13}C{^{1}H} NMR (126 MHz, $CDCl_3$) δ_C 198.0 (**C8**), 157.0 (**C4**), 134.8 (**C1**), 128.4 (**C2**), 125.7 (**C3**), 35.3 (**C5**), 31.2 (**C6**), 26.7 (**C7**)

(*S*)-4-(1-Hydroxyethyl)benzonitrile (17a)

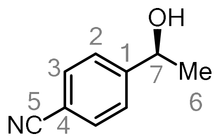

Following **general procedure D**, SAS (30 mol%), *E. coli*/*Gc*APRD (20 mg/mL), NADH (0.8 mM), and (±)-**17a** (1.3 μL, 10 μmol) were added into 1X PBS (pH 7.4, 500 μL, 20 mM) containing 10% (v/v) ^{i}PrOH in a 4 mL glass vial. The mixture was stirred and irradiated by blue LED (405 nm) at RT for 4 h. The reaction yield for **17a** was determined by ^{1}H NMR spectroscopy (37%) with **17b** as a side product (1%). The enantiomeric ratio was determined following **general procedure E**. Characterization data is in accordance with literature.[38]

Chiral HPLC: Chiralcel OJ-H (hexane : ^{i}PrOH, 90 : 10, flow rate = 1 mL min^{-1}, λ = 270 nm, 30 °C) t_R (*S*): 14.5 min, t_R (*R*): 17.1 min, 69 : 31 er

^{1}H NMR (400 MHz, CDCl$_3$) δ_H 7.70 – 7.61 (2H, m, **3-H**), 7.53 – 7.45 (2H, m, **2-H**), 4.97 (1H, qd, *J* 6.5, 3.7, **7-H**), 1.88 (1H, dd, *J* 3.8, 1.7, O-**H**), 1.50 (3H, d, *J* 6.5, **6-H**)

^{13}C{^{1}H} NMR (126 MHz, CDCl$_3$) δ_C 151.2 (**C1**), 132.5 (**C3**), 126.2 (**C2**), 119.0 (**C5**), 111.3 (**C4**), 69.9 (**C7**), 25.6 (**C6**)

4-Acetylbenzonitrile (17b)

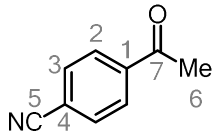

Characterization data is in accordance with literature.[40]

^{1}H NMR (500 MHz, CDCl$_3$) δ_H 8.04 (2H, d, *J* 8.2, **2-H**), 7.78 (2H, d, *J* 8.1, **3-H**), 2.65 (3H, s, **6-H**)

^{13}C{^{1}H} NMR (126 MHz, CDCl$_3$) δ_C 196.7 (**C7**), 140.1 (**C1**), 132.7 (**C3**), 128.8 (**C2**), 118.1 (**C5**), 116.6 (**C4**), 26.9 (**C6**)

(*S*)-4-(1-Hydroxyethyl)phenol (18a)

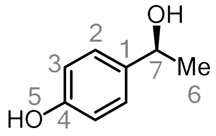

Following **general procedure D**, SAS (30 mol%), *E. coli*/*Gc*APRD (20 mg/mL), NADH (0.8 mM), and (±)-**18a** (1.4 mg, 10 μmol) were added into 1X PBS (pH 7.4, 500 μL, 20 mM) containing 10% (v/v) ^{i}PrOH in a 4 mL glass vial. The mixture was stirred and irradiated by blue LED (405 nm) at RT for 4 h. The reaction yield for **18a** was determined by ^{1}H NMR spectroscopy (34%) with **18b** as a side product (20%). The enantiomeric ratio was determined following **general procedure E** using 200 μL of the sample dissolved in DMSO-d_6. Characterization data is in accordance with literature.[41]

Following **general procedure A**, $NaBH_4$ (567 mg, 15.0 mmol) and **18b** (408 mg, 3.0 mmol) were added into MeOH (3.0 mL) at 0 °C. The mixture was stirred at RT for 30 h, heated to 60 °C for 2 h with continuous stirring, extracted with EtOAc (3 × 10 mL), and then dried *in vacuo* to afford (±)-**18a** as a white solid (357 mg, 87%).

Chiral HPLC: Chiralcel OD-H (hexane : ^{i}PrOH, 90 : 10, flow rate = 1 mL min^{-1}, λ = 230 nm, 30 °C) t_R (*S*): 13.7 min, t_R (*R*): 15.1 min, 70 : 30 er

^{1}H NMR (500 MHz, DMSO-*d*$_6$) δ_H 9.17 (1H, s, **5-H**), 7.14 – 7.08 (2H, m, **2-H**), 6.71 – 6.65 (2H, m, **3-H**), 4.91 (1H, d, *J* 4.2, O-**H**), 4.60 (1H, qd, *J* 6.4, 4.0, **7-H**), 1.26 (3H, d, *J* 6.4, **6-H**)

^{13}C{^{1}H} NMR (126 MHz, DMSO-*d*$_6$) δ_C 156.0 (**C4**), 137.7 (**C1**), 126.4 (**C2**), 114.6 (**C3**), 67.8 (**C7**), 26.0 (**C6**)

1-(4-Hydroxyphenyl)ethan-1-one (18b)

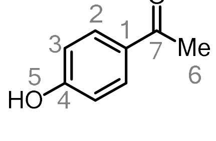

Characterization data is in accordance with literature.[42]

^{1}H NMR (500 MHz, DMSO-*d*$_6$) δ_H 10.33 (1H, s, **5-H**), 7.86 – 7.79 (2H, m, **2-H**), 6.87 – 6.81 (2H, m, **3-H**), 2.47 (3H, s, **6-H**)

^{13}C{^{1}H} NMR (126 MHz, DMSO-*d*$_6$) δ_C 196.0 (**C7**), 162.0 (**C4**), 130.7 (**C2**), 128.6 (**C1**), 115.2 (**C3**), 26.3 (**C6**)

(*S*)-1-([1,1'-Biphenyl]-4-yl)ethan-1-ol (19a)

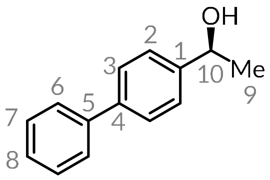

Following **general procedure D**, SAS (30 mol%), *E. coli*/*Gc*APRD (20 mg/mL), NADH (0.8 mM), and (±)-**19a** (2.0 mg, 10 μmol) were added into 1X PBS (pH 7.4, 500 μL, 20 mM) containing 15% (v/v) ^{i}PrOH in a 4 mL glass vial. The mixture was stirred and irradiated by blue LED (405 nm) at RT for 4 h. The reaction yield for **19a** was determined by ^{1}H NMR spectroscopy (64%) with **19b** as a side product (2%). The enantiomeric ratio was determined following **general procedure E**. Characterization data is in accordance with literature.[24]

Following **general procedure A**, $NaBH_4$ (170 mg, 4.5 mmol) and **19b** (589 mg, 3.0 mmol) were added into MeOH (3.0 mL) at 0 °C. The mixture was stirred at RT for 2 h, extracted with EtOAc (3 × 10 mL), and then dried *in vacuo* to afford (±)-**19a** as a white solid (415 mg, 70%).

Chiral HPLC: Chiralpak AD-H (hexane : ^{i}PrOH, 95 : 5, flow rate = 1 mL min^{-1}, λ = 230 nm, 30 °C) t_R (*S*): 11.8 min, t_R (*R*): 12.9 min, 51 : 49 er

^{1}H NMR (500 MHz, $CDCl_3$) δ_H 7.62 – 7.56 (4H, m, **3-H**, **6-H**), 7.48 – 7.41 (4H, m, **2-H**, **7-H**), 7.35 (1H, t, *J* 7.4, **8-H**), 5.01 – 4.92 (1H, m, **10-H**), 1.79 (1H, d, *J* 3.6, **O-H**), 1.55 (3H, d, *J* 6.4, **9-H**)

^{13}C{^{1}H} NMR (126 MHz, $CDCl_3$) δ_C 145.0 (**C1**), 141.0 (**C5**), 140.6 (**C4**), 128.9 (**C7**), 127.4 (**C3**, **C8**), 127.3 (**C6**), 126.0 (**C2**), 70.4 (**C10**), 25.3 (**C9**)

1-([1,1'-Biphenyl]-4-yl)ethan-1-one (19b)

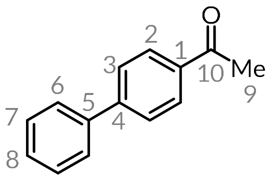

Characterization data is in accordance with literature.[36]

^{1}H NMR (500 MHz, $CDCl_3$) δ_H 8.07 – 8.01 (2H, m, **2-H**), 7.72 – 7.67 (2H, m, **3-H**), 7.66 – 7.61 (2H, m, **6-H**), 7.51 – 7.44 (2H, m, **7-H**), 7.44 – 7.37 (1H, m, **8-H**), 2.64 (3H, s, **9-H**)

^{13}C{^{1}H} NMR (126 MHz, $CDCl_3$) δ_C 197.9 (**C10**), 146.0 (**C4**), 140.0 (**C5**), 136.0 (**C1**), 129.11 (**C2** or **C7**), 129.07 (**C2** or **C7**), 128.4 (**C8**), 127.43 (**C3** or **C6**), 127.38 (**C3** or **C6**), 26.8 (**C9**)

(*S*)-1-(Pyridin-2-yl)ethan-1-ol (20a)

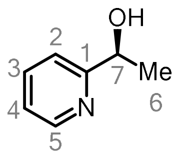

Following **general procedure D**, SAS (30 mol%), *E. coli*/*Gc*APRD (20 mg/mL), NADH (0.8 mM), and (±)-**20a** (1.1 µL, 10 µmol) were added into 1X PBS (pH 7.4, 500 µL, 20 mM) containing 10% (v/v) ^{i}PrOH in a 4 mL glass vial. The mixture was stirred and irradiated by blue LED (405 nm) at RT for 4 h. The reaction yield for **20a** was determined by ^{1}H NMR spectroscopy (39%). No side products were observed. The enantiomeric ratio was determined following **general procedure E**. Characterization data is in accordance with literature.[38]

Chiral HPLC: Chiralcel OD-H (hexane : ^{i}PrOH, 95 : 5, flow rate = 1 mL min^{-1}, λ = 254 nm, 30 °C) t_R (*R*): 8.8 min, t_R (*S*): 9.8 min, 41 : 59 er

^{1}H NMR (400 MHz, CDCl$_3$) δ_H 8.54 (1H, ddd, *J* 4.9, 1.8, 1.0, **5-H**), 7.69 (1H, td, *J* 7.7, 1.8, **3-H**), 7.31 – 7.24 (1H, m, **2-H**), 7.20 (1H, dddd, *J* 7.5, 4.9, 1.2, 0.6, **4-H**), 4.94 – 4.84 (1H, m, **7-H**), 4.27 (1H, d, *J* 4.7, O-**H**), 1.51 (3H, d, *J* 6.5, **6-H**)

^{13}C{^{1}H} NMR (126 MHz, CDCl$_3$) δ_C 163.1 (**C1**), 148.3 (**C5**), 136.9 (**C3**), 122.4 (**C2**), 119.9 (**C4**), 68.9 (**C7**), 24.4 (**C6**)

(*S*)-1-(Pyridin-3-yl)ethan-1-ol (21a)

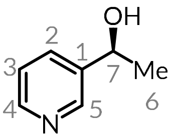

Following **general procedure D**, SAS (30 mol%), *E. coli*/*Gc*APRD (20 mg/mL), NADH (0.8 mM), and (±)-**21a** (1.1 µL, 10 µmol) were added into 1X PBS (pH 7.4, 500 µL, 20 mM) containing 10% (v/v) ^{i}PrOH in a 4 mL glass vial. The mixture was stirred and irradiated by blue LED (405 nm) at RT for 4 h. The reaction yield for **21a** was determined by ^{1}H NMR spectroscopy (74%) with **21b** as a side product (3%). The enantiomeric ratio was determined following **general procedure E**. Characterization data is in accordance with literature.[33]

Chiral HPLC: Chiralcel OJ-H (hexane : ^{i}PrOH, 90 : 10, flow rate = 1 mL min^{-1}, λ = 210 nm, 30 °C) t_R (*S*): 7.0 min, t_R (*R*): 9.0 min, 58 : 42 er

^{1}H NMR (400 MHz, $CDCl_3$) δ_H 8.54 (1H, dt, *J* 2.3, 0.7, **5-H**), 8.47 (1H, dd, *J* 4.8, 1.7, **4-H**), 7.77 – 7.69 (1H, m, **2-H**), 7.31 – 7.23 (1H, m, **3-H**), 4.94 (1H, q, *J* 6.5, **7-H**), 2.79 (1H, s, **O-H**), 1.52 (3H, d, *J* 6.5, **6-H**)

^{13}C{^{1}H} NMR (126 MHz, $CDCl_3$) δ_C 148.8 (**C5**), 147.5 (**C4**), 141.3 (**C1**), 133.3 (**C2**), 123.7 (**C3**), 68.1 (**C7**), 25.4 (**C6**)

1-(Pyridin-3-yl)ethan-1-one (21b)

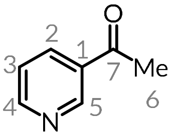

Characterization data is in accordance with literature.[30]

^{1}H NMR (400 MHz, $CDCl_3$) δ_H 9.17 (1H, dd, *J* 2.3, 0.9, **5-H**), 8.78 (1H, dd, *J* 4.8, 1.7, **4-H**), 8.23 (1H, ddd, *J* 8.0, 2.3, 1.8, **2-H**), 7.42 (1H, ddd, *J* 8.0, 4.8, 0.9, **3-H**), 2.64 (3H, s, **6-H**)

^{13}C{^{1}H} NMR (126 MHz, $CDCl_3$) δ_C 196.9 (**C7**), 153.7 (**C5**), 150.1 (**C4**), 135.6 (**C2**), 132.4 (**C1**), 123.8 (**C3**), 26.9 (**C6**)

(*S*)-1-(Pyridin-4-yl)ethan-1-ol (22a)

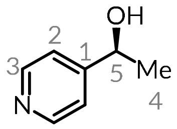

Following **general procedure D**, SAS (30 mol%), *E. coli*/*Gc*APRD (20 mg/mL), NADH (0.8 mM), and (±)-**22a** (1.2 mg, 10 μmol) were added into 1X PBS (pH 7.4, 500 μL, 20 mM) containing 10% (v/v) ^{i}PrOH in a 4 mL glass vial. The mixture was stirred and irradiated by blue LED (405 nm) at RT for 4 h. The reaction yield for **22a** was determined by ^{1}H NMR spectroscopy (64%). No side products were observed. The enantiomeric ratio was determined following **general procedure E**. Characterization data is in accordance with literature.[43]

Chiral HPLC: Chiralcel OD-H column (hexane : ^{i}PrOH, 93 : 7, flow rate = 1 mL min^{-1}, λ = 254 nm, 30 °C) t_R (*S*): 14.6 min, t_R (*R*): 16.7 min, 55 : 45 er

^{1}H NMR (500 MHz, $CDCl_3$) δ_H 8.57 – 8.52 (2H, m, **3-H**), 7.32 – 7.27 (2H, m, **2-H**), 4.90 (1H, q, *J* 6.5, **5-H**), 1.50 (3H, d, *J* 6.5, **4-H**)

^{13}C{^{1}H} NMR (126 MHz, $CDCl_3$) δ_C 154.8 (**C1**), 150.0 (**C3**), 120.5 (**C2**), 69.1 (**C5**), 25.3 (**C4**)

(*S*)-1-(Pyrimidin-4-yl)ethan-1-ol (23a)

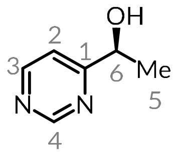

Following **general procedure D**, SAS (30 mol%), *E. coli*/*Gc*APRD (20 mg/mL), NADH (0.8 mM), and (±)-**23a** (1.2 mg, 10 μmol) were added into 1X PBS (pH 7.4, 500 μL, 20 mM) containing 10% (v/v) ^{i}PrOH in a 4 mL glass vial. The mixture was stirred and irradiated by blue LED (405 nm) at RT for 4 h. The reaction yield for **23a** was determined by ^{1}H NMR spectroscopy (57%). No side products were observed. The enantiomeric ratio was determined following **general procedure E**. Characterization data is in accordance with literature.[44]

Following **general procedure A**, $NaBH_4$ (68 mg, 1.8 mmol) and **23b** (147 mg, 1.2 mmol) were added into MeOH (3.0 mL) at 0 °C. The mixture was stirred at RT for 2 h, extracted with $CHCl_3$: ^{i}PrOH (3 : 1, 5 × 10 mL), purified by flash column chromatography (acetone : MeOH, 100 : 0 to 0 : 100), and then dried *in vacuo* to afford (±)-**23a** as an orange oil (85 mg, 57%).

Chiral HPLC: Chiralcel OJ-H column (hexane : ^{i}PrOH, 95 : 5, flow rate = 1 mL min^{-1}, λ = 254 nm, 30 °C) t_R (*R*): 11.1 min, t_R (*S*): 12.2 min, 48 : 52 er

^{1}H NMR (500 MHz, $CDCl_3$) δ_H 9.18 (1H, d, *J* 1.4, **4-H**), 8.72 (1H, d, *J* 5.2, **3-H**), 7.39 – 7.34 (1H, m, **2-H**), 4.88 (1H, q, *J* 6.7, **6-H**), 3.60 (1H, s, O-**H**), 1.53 (3H, d, *J* 6.7, **5-H**)

$^{13}C\{^1H\}$ NMR (126 MHz, $CDCl_3$) δ_C 171.7 (**C1**), 158.2 (**C4**), 157.3 (**C3**), 117.4 (**C2**), 68.7 (**C6**), 23.8 (**C5**)

(*S*)-1-(Thiophen-2-yl)ethan-1-ol (24a)

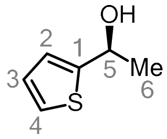

Following **general procedure D**, SAS (30 mol%), *E. coli*/*Gc*APRD (20 mg/mL), NADH (0.8 mM), and (±)-**24a** (1.1 µL, 10 µmol) were added into 1X PBS (pH 7.4, 500 µL, 20 mM) containing 10% (v/v) ^{i}PrOH in a 4 mL glass vial. The mixture was stirred and irradiated by blue LED (405 nm) at RT for 4 h. The reaction yield for **24a** was determined by ^{1}H NMR spectroscopy (18%) with **24b** as a side product (32%). The enantiomeric ratio was determined following **general procedure E**. Characterization data is in accordance with literature.[35]

Chiral HPLC: Chiralcel OJ-H column (hexane : ^{i}PrOH, 95 : 5, flow rate = 1 mL min^{-1}, λ = 210 nm, 30 °C) t_R (*S*): 11.6 min, t_R (*R*): 14.1 min, 93 : 7 er

^{1}H NMR (400 MHz, CDCl$_3$) δ_H 7.24 (1H, dd, *J* 4.9, 1.4, **4-H**), 7.03 – 6.93 (2H, m, **2-H**, **3-H**), 5.14 (1H, qdd, *J* 6.4, 4.7, 0.8, **5-H**), 1.97 (1H, d, *J* 4.6, O-**H**), 1.61 (3H, d, *J* 6.4, **6-H**)

^{13}C{^{1}H} NMR (126 MHz, CDCl$_3$) δ_C 150.0 (**C1**), 126.8 (**C4**), 124.6 (**C3**), 123.4 (**C2**), 66.5 (**C5**), 25.4 (**C6**)

1-(Thiophen-2-yl)ethan-1-one (24b)

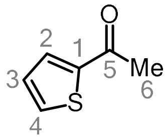

Characterization data is in accordance with literature.[30]

^{1}H NMR (400 MHz, CDCl$_3$) δ_H 7.70 (1H, dd, *J* 3.8, 1.2, **4-H**), 7.63 (1H, dd, *J* 4.9, 1.2, **3-H**), 7.13 (1H, dd, *J* 5.0, 3.8, **2-H**), 2.57 (3H, s, **6-H**)

^{13}C{^{1}H} NMR (126 MHz, CDCl$_3$) δ_C 190.9 (**C5**), 144.7 (**C1**), 133.9 (**C4**), 132.6 (**C2**), 128.2 (**C3**), 27.1 (**C6**)

(*S*)-1-Phenylpropan-1-ol (25a)

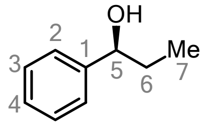

Following **general procedure D**, SAS (30 mol%), *E. coli*/*Gc*APRD (20 mg/mL), NADH (0.8 mM), and (±)-**25a** (1.4 µL, 10 µmol) were added into 1X PBS (pH 7.4, 500 µL, 20 mM) containing 10% (v/v) ^{i}PrOH in a 4 mL glass vial. The mixture was stirred and irradiated by blue LED (405 nm) at RT for 4 h. The reaction yield for **25a** was determined by ^{1}H NMR spectroscopy (38%) with **25b** (26%), benzyl alcohol (4%), and benzaldehyde (trace) as side products. The enantiomeric ratio was determined following **general procedure E**. Characterization data is in accordance with literature.[24]

Chiral HPLC: Chiralcel OJ-H column (hexane : ^{i}PrOH, 93 : 7, flow rate = 0.5 mL min^{-1}, λ = 210 nm, 30 °C) t_R (*S*): 15.4 min, t_R (*R*): 16.3 min, 96 : 4 er

^{1}H NMR (500 MHz, CDCl$_3$) δ$_H$ 7.39 – 7.32 (4H, m, **2-H**, **3-H**), 7.32 – 7.25 (1H, m, **4-H**), 4.61 (1H, ddd, *J* 7.1, 6.0, 3.4, **5-H**), 1.89 – 1.70 (3H, m, **6-H**, O-**H**), 0.92 (3H, t, *J* 7.4, **7-H**)

^{13}C{^{1}H} NMR (126 MHz, CDCl$_3$) δ$_C$ 144.7 (**C1**), 128.6 (**C3**), 127.7 (**C2**), 126.1 (**C4**), 76.2 (**C5**), 32.0 (**C6**), 10.3 (**C7**)

Propiophenone (25b)

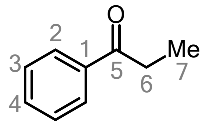

Characterization data is in accordance with literature.[31]

^{1}H NMR (400 MHz, CDCl$_3$) δ$_H$ 8.01 – 7.93 (2H, m, **2-H**), 7.60 – 7.51 (1H, m, **4-H**), 7.50 – 7.41 (2H, m, **3-H**), 3.01 (2H, q, *J* 7.2, **6-H**), 1.23 (3H, t, *J* 7.2, **7-H**)

^{13}C{^{1}H} NMR (126 MHz, CDCl$_3$) δ$_C$ 201.0 (**C5**), 137.1 (**C1**), 133.0 (**C4**), 128.7 (**C2**), 128.1 (**C3**), 31.9 (**C6**), 8.4 (**C7**)

(*S*)-1-phenylbutan-1-ol (26a)

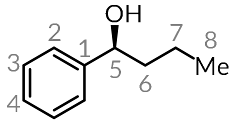

Following **general procedure D**, SAS (30 mol%), *E. coli*/*Gc*APRD (20 mg/mL), NADH (0.8 mM), and (±)-**26a** (1.5 μL, 10 μmol) were added into 1X PBS (pH 7.4, 500 μL, 20 mM) containing 10% (v/v) ^{i}PrOH in a 4 mL glass vial. The mixture was stirred and irradiated by blue LED (405 nm) at RT for 4 h. The reaction yield for **26a** was determined by ^{1}H NMR spectroscopy (11%) with **26b** as a side product (31%). The enantiomeric ratio was determined following **general procedure E**. Characterization data is in accordance with literature.[45]

Following **general procedure A**, $NaBH_4$ (114 mg, 3.0 mmol) and **26b** (293 uL, 1.0 mmol) were added into MeOH (2.0 mL) at 0 °C. The mixture was stirred at RT for 2 h, extracted with $CHCl_2$ (3 × 10 mL), and then dried *in vacuo* to afford (±)-**26a** as a colorless oil (216 mg, 72%).

Chiral HPLC: Chiralpak AS-H (hexane : ^{i}PrOH, 99 : 1, flow rate = 0.5 mL min^{-1}, λ = 210 nm, 30 °C) t_R (*R*): 20.0 min, t_R (*S*): 21.4 min, 54 : 46 er

^{1}H NMR (500 MHz, $CDCl_3$) δ_H 7.39 – 7.32 (4H, m, **2-H**, **3-H**), 7.28 (1H, dt, *J* 8.8, 4.2, **4-H**), 4.72 – 4.65 (1H, m, **5-H**), 1.85 – 1.64 (3H, m, **6-H, O-H**), 1.50 – 1.24 (2H, m, **7-H**), 0.94 (3H, t, *J* 7.4, **8-H**)

^{13}C{^{1}H} NMR (126 MHz, $CDCl_3$) δ_C 145.1 (**C1**), 128.6 (**C3**), 127.6 (**C4**), 126.0 (**C2**), 74.6 (**C5**), 41.4 (**C6**), 19.2 (**C7**), 14.1 (**C8**)

1-Phenylbutan-1-one (26b)

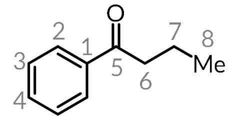

Characterization data is in accordance with literature.[46]

^{1}H NMR (500 MHz, $CDCl_3$) δ_H 7.99 – 7.92 (2H, m, **2-H**), 7.58 – 7.51 (1H, m, 4-**H**), 7.49 – 7.41 (2H, m, **3-H**), 2.94 (2H, t, *J* 7.3, **6-H**), 1.77 (2H, h, *J* 7.4, **7-H**), 1.00 (3H, t, *J* 7.4, **8-H**)

^{13}C{^{1}H} NMR (126 MHz, $CDCl_3$) δ_C 200.5 (**C5**), 137.2 (**C1**), 133.0 (**C4**), 128.7 (**C3**), 128.2 (**C2**), 40.6 (**C6**), 17.9 (**C7**), 14.0 (**C8**)

9 References

(1) Bertani, G. Studies on lysogenesis I: the mode of phage liberation by lysogenic Escherichia coli. *J. Bacteriol.* **1951**, *62* (3), 293-300.

(2) Tartoff, K. D.; Hobbs, C. A. Improved media for growing plasmid and cosmid clones. *Bethesda Res. Lab. Focus* **1987**, *9*, 12.

(3) Wallace, S.; Balskus, E. P. Interfacing microbial styrene production with a biocompatible cyclopropanation reaction. *Angew. Chem. Int. Ed.* **2015**, *54* (24), 7106-7109.

(4) Salis, H. M.; Mirsky, E. A.; Voigt, C. A. Automated design of synthetic ribosome binding sites to control protein expression. *Nat. Biotechnol.* **2009**, *27* (10), 946-950. . Espah Borujeni, A.; Channarasappa, A. S.; Salis, H. M. Translation rate is controlled by coupled trade-offs between site accessibility, selective RNA unfolding and sliding at upstream standby sites. *Nucleic Acids Res.* **2014**, *42* (4), 2646-2659. . Espah Borujeni, A.; Salis, H. M. Translation initiation is controlled by RNA folding kinetics via a ribosome drafting mechanism. *J. Am. Chem. Soc.* **2016**, *138* (22), 7016-7023. . Espah Borujeni, A.; Cetnar, D.; Farasat, I.; Smith, A.; Lundgren, N.; Salis, H. M. Precise quantification of translation inhibition by mRNA structures that overlap with the ribosomal footprint in N-terminal coding sequences. *Nucleic Acids Res.* **2017**, *45* (9), 5437-5448. . Reis, A. C.; Salis, H. M. An automated model test system for systematic development and improvement of gene expression models. *ACS synthetic biology* **2020**, *9* (11), 3145-3156. . Cetnar, D. P.; Salis, H. M. Systematic quantification of sequence and structural determinants controlling mRNA stability in bacterial operons. *ACS Synthetic Biology* **2021**, *10* (2), 318-332.

(5) Cohen, S. N.; Chang, A. C.; Hsu, L. Nonchromosomal antibiotic resistance in bacteria: genetic transformation of Escherichia coli by R-factor DNA. *Proc. Natl. Acad. Sci. USA* **1972**, *69* (8), 2110-2114.

(6) Gasteiger, E.; Gattiker, A.; Hoogland, C.; Ivanyi, I.; Appel, R. D.; Bairoch, A. ExPASy: the proteomics server for in-depth protein knowledge and analysis. *Nucleic Acids Res.* **2003**, *31* (13), 3784-3788.

(7) Park, S. T.; Feenstra, J. S.; Zewail, A. H. Ultrafast electron diffraction: Excited state structures and chemistries of aromatic carbonyls. *The Journal of chemical physics* **2006**, *124* (17).

(8) Yamamoto, K.; Arita, K.; Shiota, M.; Kuriyama, M.; Onomura, O. Electrochemical formal homocoupling of sec-alcohols. *Beilstein J. Org. Chem.* **2022**, *18* (1), 1062-1069. . Wan, J. L.;

Huang, J. M. Electrochemical Dearomative Amination of Phenol Derivatives: Access to Spirooxazolidinones. *Adv. Synth. Catal.* **2023**, *365* (8), 1211-1216. . Juhl, M.; Lee, J. W. Umpolung reactivity of aldehydes toward carbon dioxide. *Angew. Chem. Int. Ed.* **2018**, *57* (38), 12318-12322.

(9) O'Boyle, N. M.; Banck, M.; James, C. A.; Morley, C.; Vandermeersch, T.; Hutchison, G. R. Open Babel: An open chemical toolbox. *Journal of cheminformatics* **2011**, *3*, 1-14.

(10) Koesoema, A. A.; Sugiyama, Y.; Xu, Z.; Standley, D. M.; Senda, M.; Senda, T.; Matsuda, T. Structural basis for a highly (S)-enantioselective reductase towards aliphatic ketones with only one carbon difference between side chain. *Appl. Microbiol. Biotechnol.* **2019**, *103* (23), 9543-9553, OriginalPaper. DOI: doi:10.1007/s00253-019-10093-w

(11) Zhang, Y.; Sanner, M. F. AutoDock CrankPep: combining folding and docking to predict protein–peptide complexes. *Bioinformatics* **2019**, *35* (24), 5121-5127.

(12) Forli, S.; Huey, R.; Pique, M. E.; Sanner, M. F.; Goodsell, D. S.; Olson, A. J. Computational protein–ligand docking and virtual drug screening with the AutoDock suite. *Nature protocols* **2016**, *11* (5), 905-919.

(13) Trott, O.; Olson, A. J. AutoDock Vina: improving the speed and accuracy of docking with a new scoring function, efficient optimization, and multithreading. *J. Comput. Chem.* **2010**, *31* (2), 455-461. DOI: 10.1002/jcc.21334 . Eberhardt, J.; Santos-Martins, D.; Tillack, A. F.; Forli, S. AutoDock Vina 1.2. 0: New docking methods, expanded force field, and python bindings. *J. Chem. Inf. Model.* **2021**, *61* (8), 3891-3898. DOI: 10.1021/acs.jcim.1c00203

(14) Abramson, J.; Adler, J.; Dunger, J.; Evans, R.; Green, T.; Pritzel, A.; Ronneberger, O.; Willmore, L.; Ballard, A. J.; Bambrick, J.; et al. Accurate structure prediction of biomolecular interactions with AlphaFold 3. *Nature* **2024**, *630* (8016), 493-500. DOI: 10.1038/s41586-024-07487-w . Chen, X.; Zhang, Y.; Lu, C.; Ma, W.; Guan, J.; Gong, C.; Yang, J.; Zhang, H.; Zhang, K.; Wu, S.; et al. Protenix - Advancing Structure Prediction Through a Comprehensive AlphaFold3 Reproduction. *bioRxiv* **2025**, 2025.2001.2008.631967. DOI: 10.1101/2025.01.08.631967

(15) Madeira, F.; Madhusoodanan, N.; Lee, J.; Eusebi, A.; Niewielska, A.; Tivey, A. R.; Lopez, R.; Butcher, S. The EMBL-EBI Job Dispatcher sequence analysis tools framework in 2024. *Nucleic Acids Res.* **2024**, gkae241.

(16) Troshin, P. V.; Procter, J. B.; Barton, G. J. Java bioinformatics analysis web services for multiple sequence alignment—JABAWS: MSA. *Bioinformatics* **2011**, *27* (14), 2001-2002.

(17) *EvoluChem™ LEDs*. https://hepatochem.com/photoreactors-leds-accessories/photoreactor-leds-evoluchem/ (accessed 2024-10-20).

(18) Yamamoto, T.; Nakata, Y.; Cao, C.; Sugiyama, Y.; Asanuma, Y.; Kanamaru, S.; Matsuda, T. Acetophenone reductase with extreme stability against a high concentration of organic compounds or an elevated temperature. *Appl. Microbiol. Biotechnol.* **2013**, *97* (24), 10413-10421. DOI: 10.1007/s00253-013-4801-5

(19) Peletier, L. A.; Gabrielsson, J. Impact of enzyme turnover on the dynamics of the Michaelis–Menten model. *Math. Biosci.* **2022**, *346*, 108795. DOI: 10.1016/j.mbs.2022.108795

(20) Bloh, J. Z. A holistic approach to model the kinetics of photocatalytic reactions. *Frontiers in chemistry* **2019**, *7*, 128.

(21) *Escherichia coli and Salmonella. Cellular and molecular biology*; American Society for Microbiology, 1996.

(22) Rosano, G. L.; Ceccarelli, E. A. Recombinant protein expression in Escherichia coli: advances and challenges. *Frontiers in microbiology* **2014**, *5*, 172.

(23) Hansch, C.; Leo, A.; Taft, R. A survey of Hammett substituent constants and resonance and field parameters. *Chem. Rev.* **1991**, *91* (2), 165-195. DOI: DOI 10.1021/cr00002a004

(24) Rudzka, A.; Antos, N.; Reiter, T.; Kroutil, W.; Borowiecki, P. One-pot sequential two-step photo-biocatalytic deracemization of sec-alcohols combining photocatalytic oxidation and bioreduction. *ACS Catal.* **2024**, *14* (3), 1808-1823. DOI: 10.1021/acscatal.3c05100

(25) Nikitas, N. F.; Tzaras, D. I.; Triandafillidi, I.; Kokotos, C. G. Photochemical oxidation of benzylic primary and secondary alcohols utilizing air as the oxidant. *Green Chem.* **2020**, *22* (2), 471-477. DOI: 10.1039/c9gc03000j

(26) Liu, Y.; Yan, J.; Yuan, Q.; Ma, L.; Zhou, L.; He, Y.; Liu, G.; Yue, X.; Jiang, Y. Enzymatic reduction of halogenated aryl ketones in an aqueous micellar solution with enhanced catalytic performance. *Green Chem.* **2024**, *26* (11), 6666-6674.

(27) Yadav, J. S.; Reddy, B. V. S.; Sreelakshmi, C.; Rao, A. B. Enantioselective reduction of prochiral ketones employing sprouted Pisum sativa as biocatalyst. *Synthesis* **2009**, *2009* (11), 1881-1885.

(28) Chen, Y.-X.; He, J.-T.; Wu, M.-C.; Liu, Z.-L.; Tang, K.; Xia, P.-J.; Chen, K.; Xiang, H.-Y.; Chen, X.-Q.; Yang, H. Photochemical organocatalytic aerobic cleavage of C= C bonds enabled by charge-transfer complex formation. *Org. Lett.* **2022**, *24* (22), 3920-3925.

(29) Rodríguez-Fernández, L.; Lavandera, I.; Gotor-Fernández, V. Photocatalytic oxidative cleavage of alkenes followed by carbonyl stereoselective bioreduction for the synthesis of enantioenriched secondary alcohols. *Adv. Synth. Catal.* **2024**, *366* (4), 900-908.

(30) Al-Romaizan, A. N.; Gangwar, M. K.; Verma, A.; Bawaked, S. M.; Saleh, T. S.; Al-Ammari, R. H.; Butcher, R. J.; Siddiqui, I. R.; Mostafa, M. M. M. Catalytic Acceptorless Dehydrogenation (CAD) of Secondary Benzylic Alcohols into Value-Added Ketones Using Pd (II)–NHC Complexes. *Molecules* **2023**, *28* (13), 4992.

(31) Qi, R.; Bai, T.; Tang, S.; Hou, M.; Zhang, Z.; Xie, W.; Deng, Y.; Zhou, H.; Qiu, G. Solvent-promoted photochemical carbonylation of benzylic C–H bonds under iron catalysis. *Org. Biomol. Chem.* **2023**, *21* (26), 5382-5386.

(32) Lu, Y.; Kasahara, A.; Hyodo, T.; Ohara, K.; Yamaguchi, K.; Otani, Y.; Ohwada, T. Isolation and reactions of imidoyl fluorides generated from oxime using the diethylaminosulfur trifluoride/tetrahydrofuran (DAST–THF) system. *Org. Lett.* **2023**, *25* (19), 3482-3486.

(33) Wang, W.; Tachibana, R.; Zou, Z.; Chen, D.; Zhang, X.; Lau, K.; Pojer, F.; Ward, T. R.; Hu, X. Manganese Transfer Hydrogenases Based on the Biotin-Streptavidin Technology. *Angew. Chem.* **2023**, *62* (43), e202311896.

(34) Liang, J.-X.; Yang, P.-F.; Shu, W. Synthesis of (Hetero) aryl/Alkenyl Iodides via Ni-Catalyzed Finkelstein Reaction from Bromides or Chlorides. *Organometallics* **2022**, *41* (24), 3795-3800.

(35) Zhang, L.; Liu, C.; Sun, M.; Liang, C.; Cao, L.; Yao, X.; Ma, Y.; Cheng, R.; Ye, J. Iridium-catalyzed asymmetric hydrogenation of simple ketones with tridentate PNN ligands bearing unsymmetrical vicinal diamines. *J. Org. Chem.* **2023**, *88* (5), 2942-2951.

(36) Zhang, Z.; Lv, Y.; Ji, L.; Chen, P.; Han, S.; Zhu, Y.; Li, L.; Jia, Z.; Loh, T. P. Triaryl Carbenium Ion Pair Mediated Electrocatalytic Benzylic C– H Oxygenation in Air. *Angew. Chem. Int. Ed.* **2024**, *63* (28), e202406588.

(37) Abuhafez, N.; Ehlers, A. W.; de Bruin, B.; Gramage-Doria, R. Markovnikov-Selective Cobalt-Catalyzed Wacker-Type Oxidation of Styrenes into Ketones under Ambient Conditions Enabled by Hydrogen Bonding. *Angew. Chem.* **2024**, *63* (3), e202316825.
(38) Swamy, P. C. A.; Varenikov, A.; de Ruiter, G. Chiral Imidazo [1, 5-a] pyridine–Oxazolines: A Versatile Family of NHC Ligands for the Highly Enantioselective Hydrosilylation of Ketones. *Organometallics* **2020**, *39* (2), 247-257.
(39) Karpova, L.; Daniel, M.; Kancherla, R.; Muralirajan, K.; Maity, B.; Rueping, M. Excited-State Nickel-Catalyzed Amination of Aryl Bromides: Synthesis of Diphenylamines and Primary Anilines. *Org. Lett.* **2024**, *26* (8), 1657-1661.
(40) Duran-Camacho, G.; Hethcox, J. C. Nickel-Catalyzed Cyanation of (Hetero) aryl Bromides Using DABAL-Me3 as a Soluble Reductant. *Org. Lett.* **2022**, *24* (45), 8397-8400.
(41) Kišić, A.; Stephan, M.; Mohar, B. ansa-Ruthenium (II) Complexes of R2NSO2DPEN-(CH2) n (η6-Aryl) Conjugate Ligands for Asymmetric Transfer Hydrogenation of Aryl Ketones. *Adv. Synth. Catal.* **2015**, *357* (11), 2540-2546.
(42) Ni, P.; Yang, L.; Shen, Y.; Zhang, L.; Ma, Y.; Sun, M.; Cheng, R.; Ye, J. Synthesis of Phenols from Aryl Ammonium Salts under Mild Conditions. *J. Org. Chem.* **2022**, *87* (19), 12677-12687.
(43) Zhang, L.; Lu, Z.; Rander, A. R.; Williams, T. J. An ambient pressure, direct hydrogenation of ketones. *Chem. Commun.* **2023**, *59* (52), 8107-8110.
(44) Kaur, M.; Cooper, J. C.; Van Humbeck, J. F. Site-selective benzylic C–H hydroxylation in electron-deficient azaheterocycles. *Org. Biomol. Chem.* **2024**, *22* (24), 4888-4894.
(45) Paolillo, J. M.; Duke, A. D.; Gogarnoiu, E. S.; Wise, D. E.; Parasram, M. Anaerobic hydroxylation of C (sp3)–H bonds enabled by the synergistic nature of photoexcited nitroarenes. *J. Am. Chem. Soc.* **2023**, *145* (5), 2794-2799.
(46) Luján, A. P.; Bhat, M. F.; Saravanan, T.; Poelarends, G. J. Exploring the Substrate Scope and Catalytic Promiscuity of Nitroreductase-Like Enzymes. *Adv. Synth. Catal.* **2024**, *366* (22), 4679-4687.

10 NMR Spectral data

(*S*)-1-(4-Fluorophenyl)ethan-1-ol (1a)

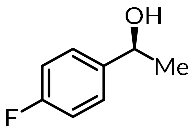

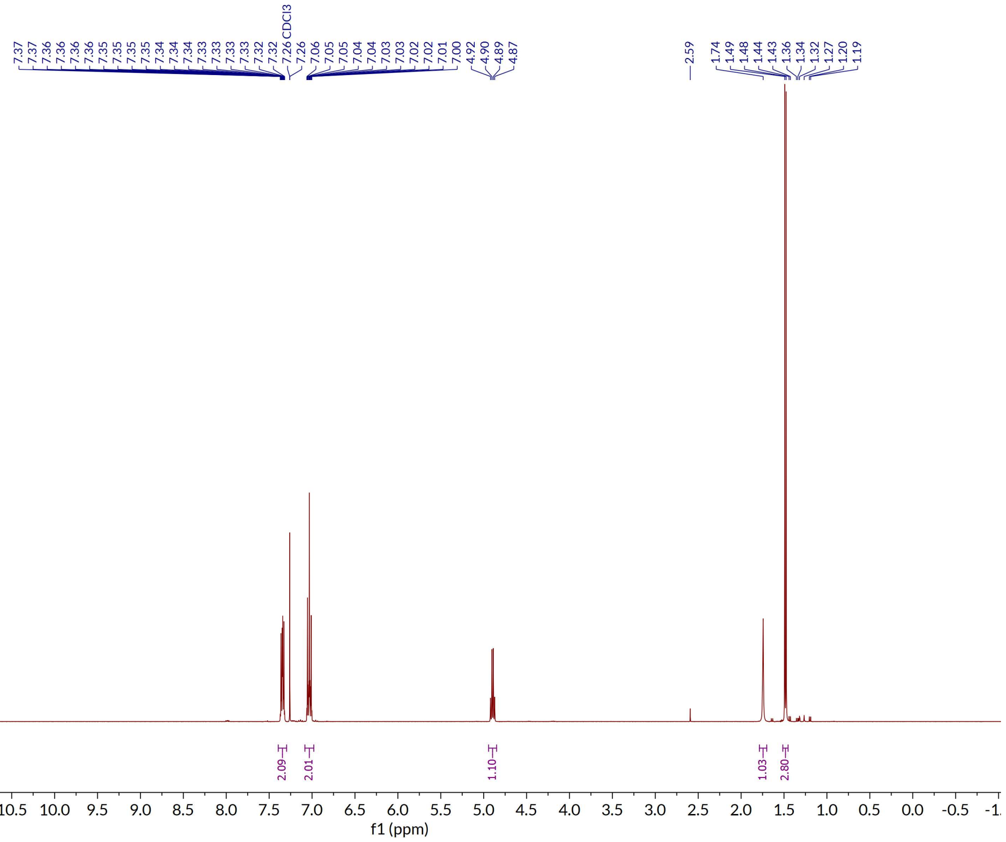

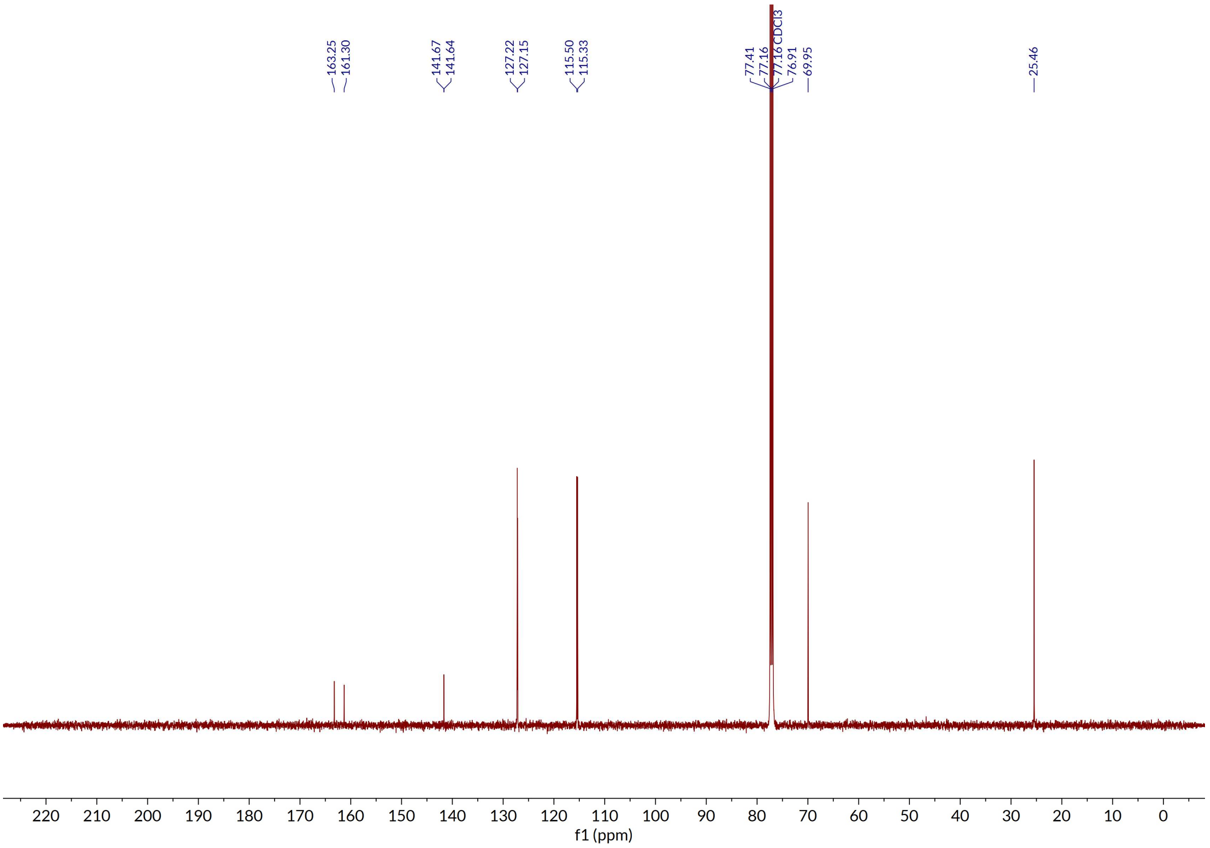

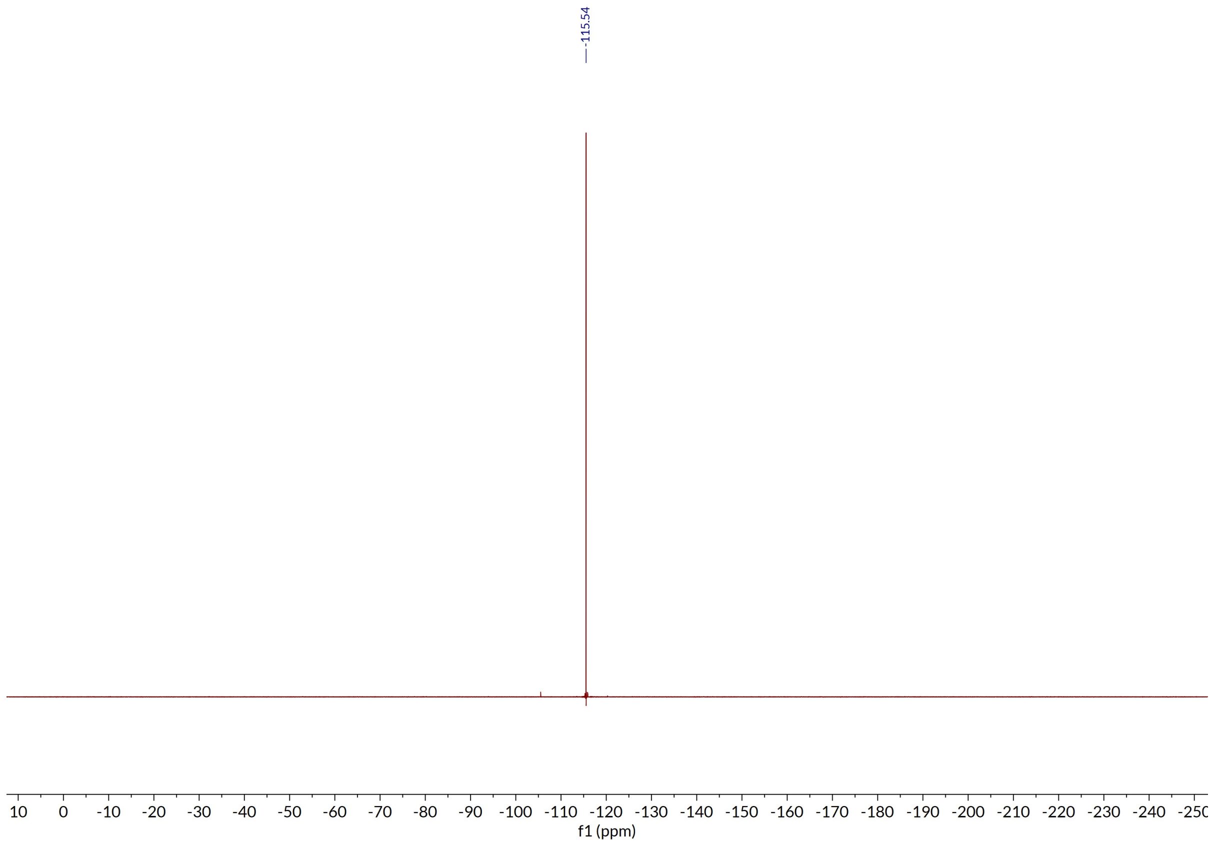

1-(4-Fluorophenyl)ethan-1-one (1b)

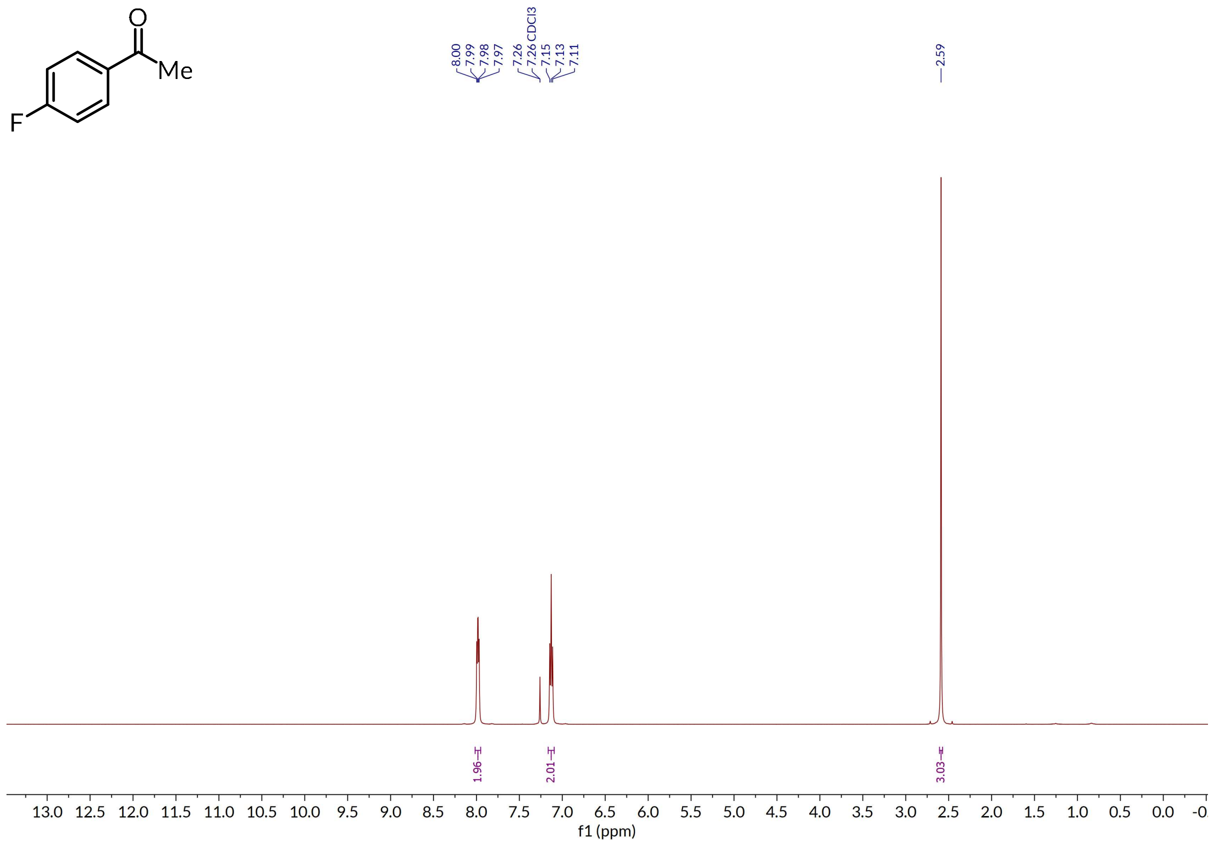

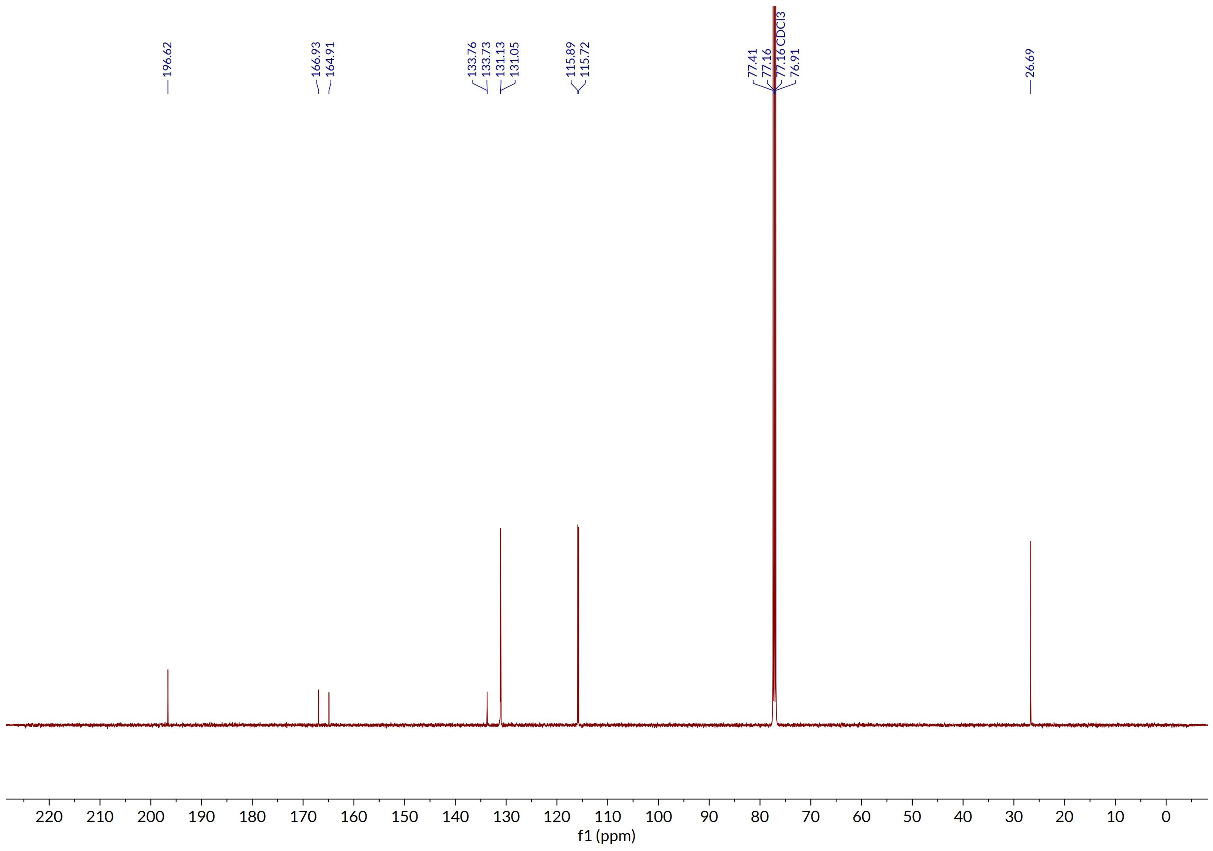
196.62
166.93
164.91
133.76
133.73
131.13
131.05
115.89
115.72
77.41
77.16
77.16 CDCl3
76.91
26.69
220 210 200 190 180 170 160 150 140 130 120 110 100 90 80 70 60 50 40 30 20 10 0
f1 (ppm)

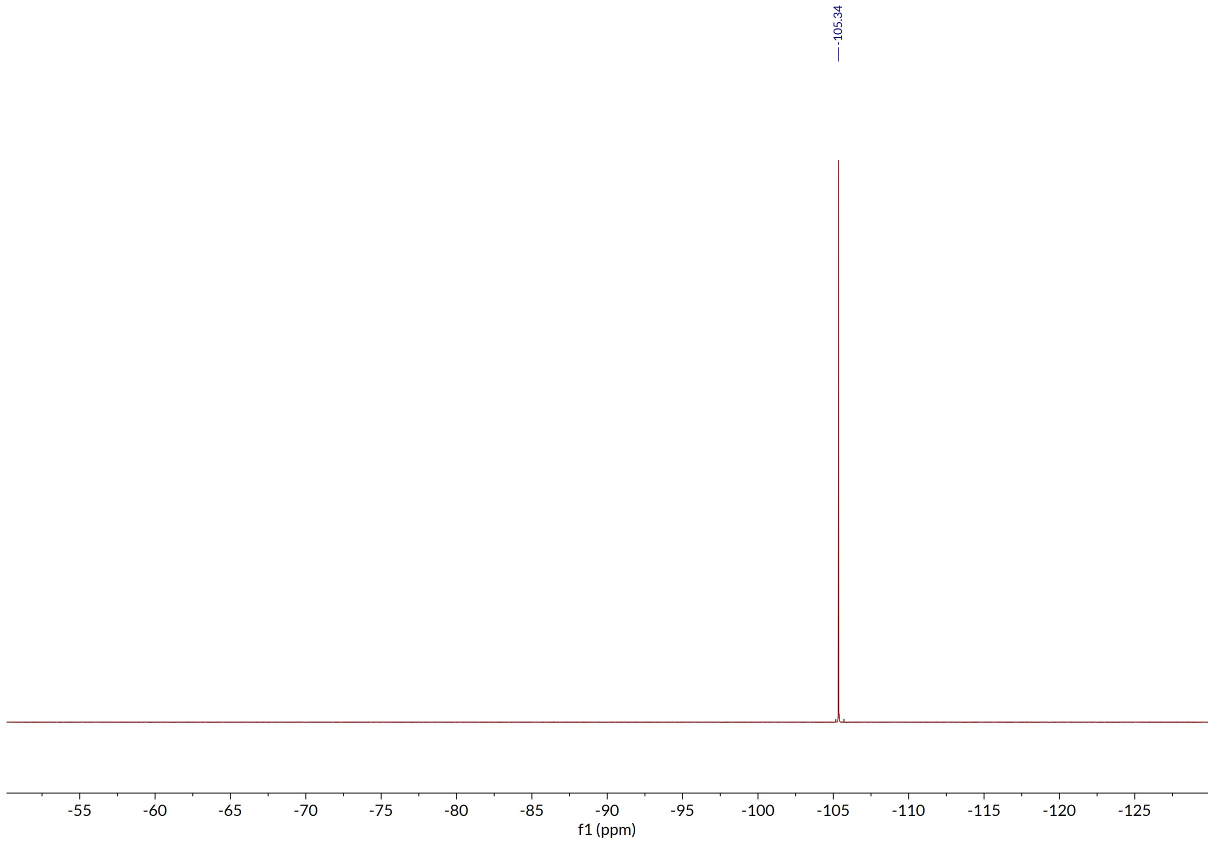
-105.34
-55 -60 -65 -70 -75 -80 -85 -90 -95 -100 -105 -110 -115 -120 -125
f1 (ppm)

(*S*)-1-Phenylethan-1-ol (2a)

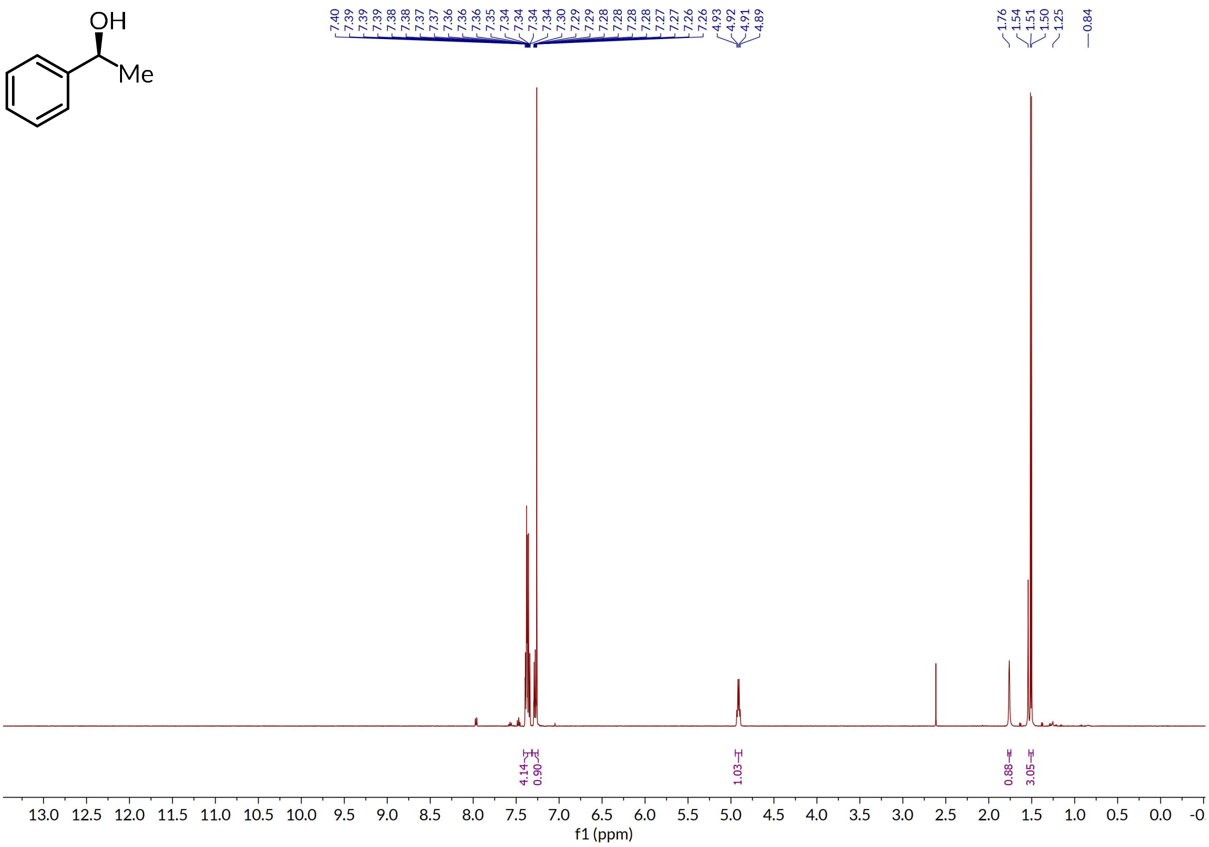

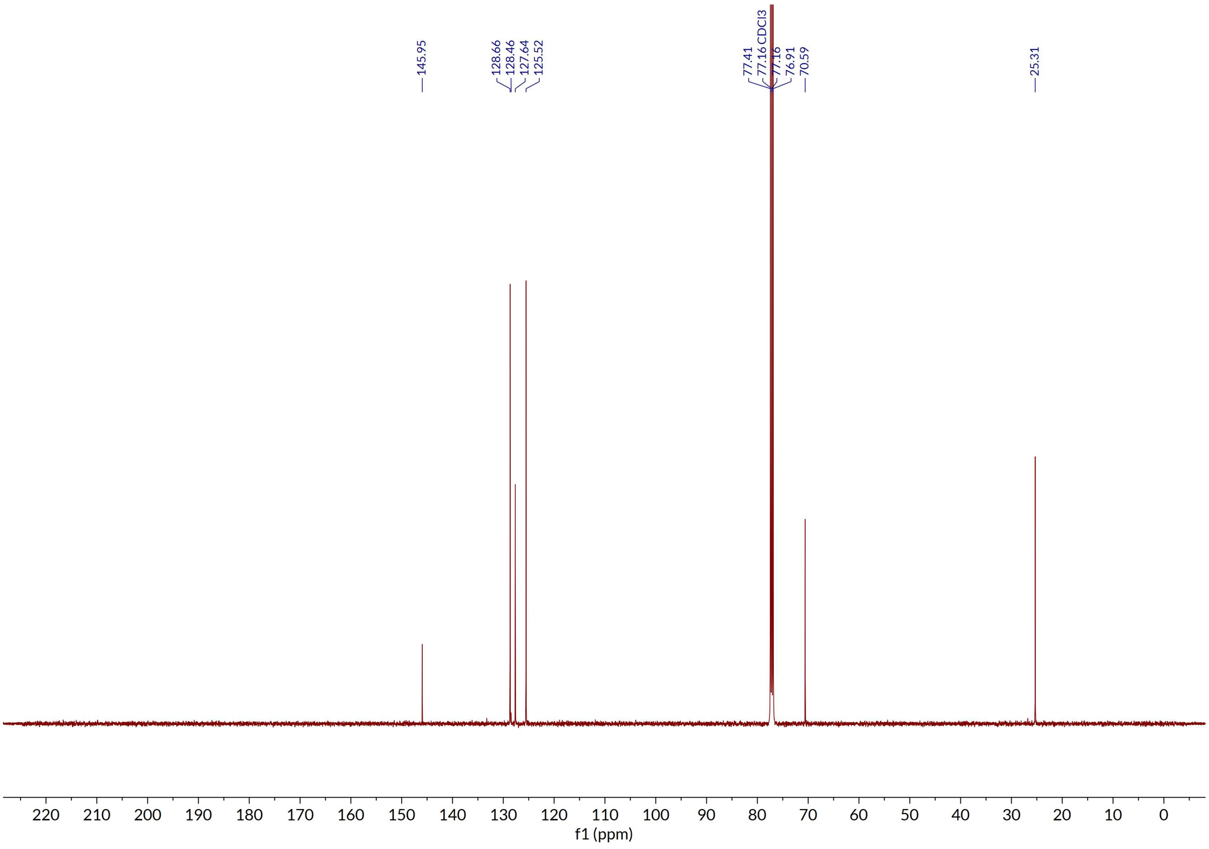

Acetophenone (2b)

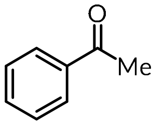

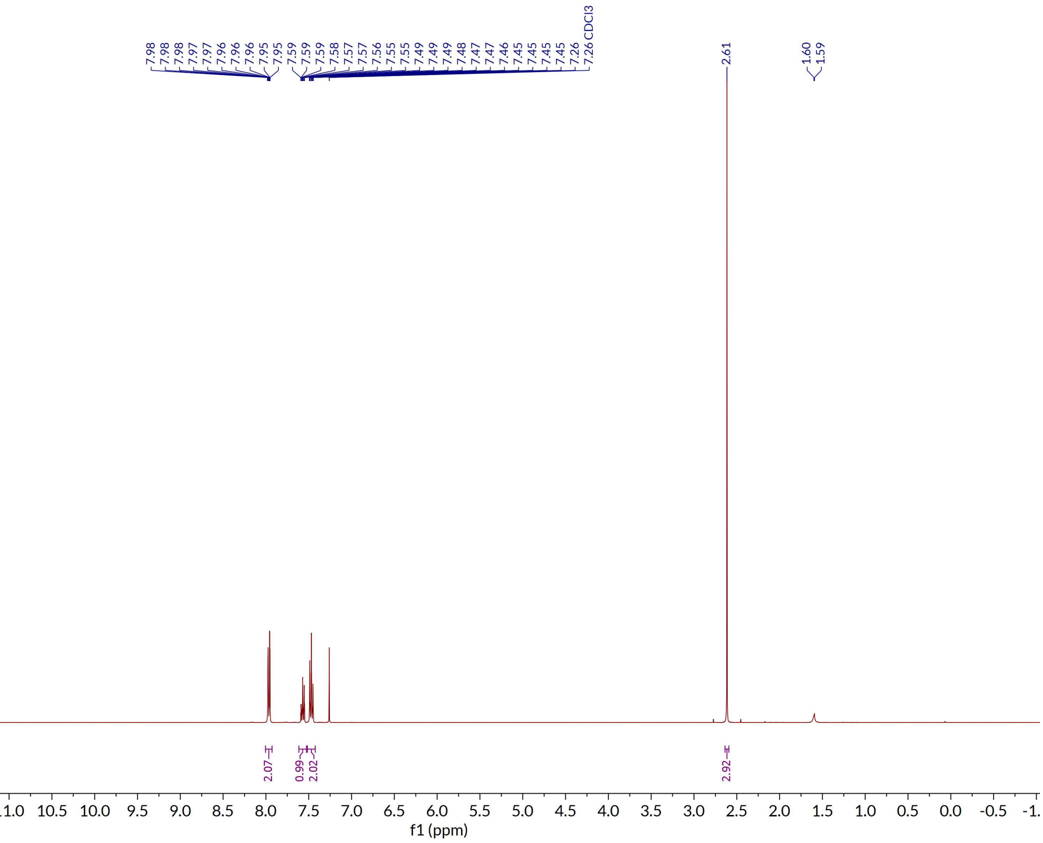

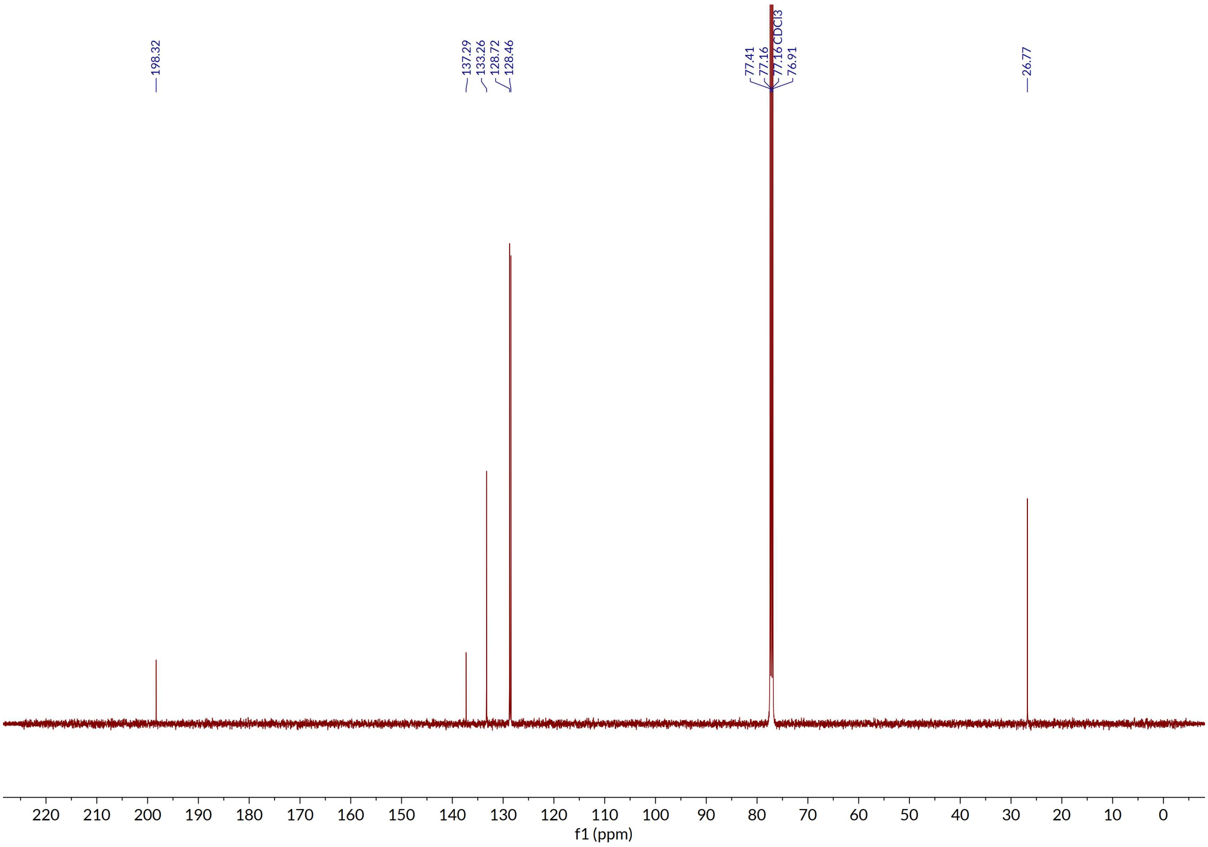

(*S*)-1-(2-Fluorophenyl)ethan-1-ol (3a)

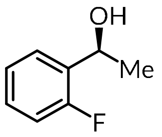

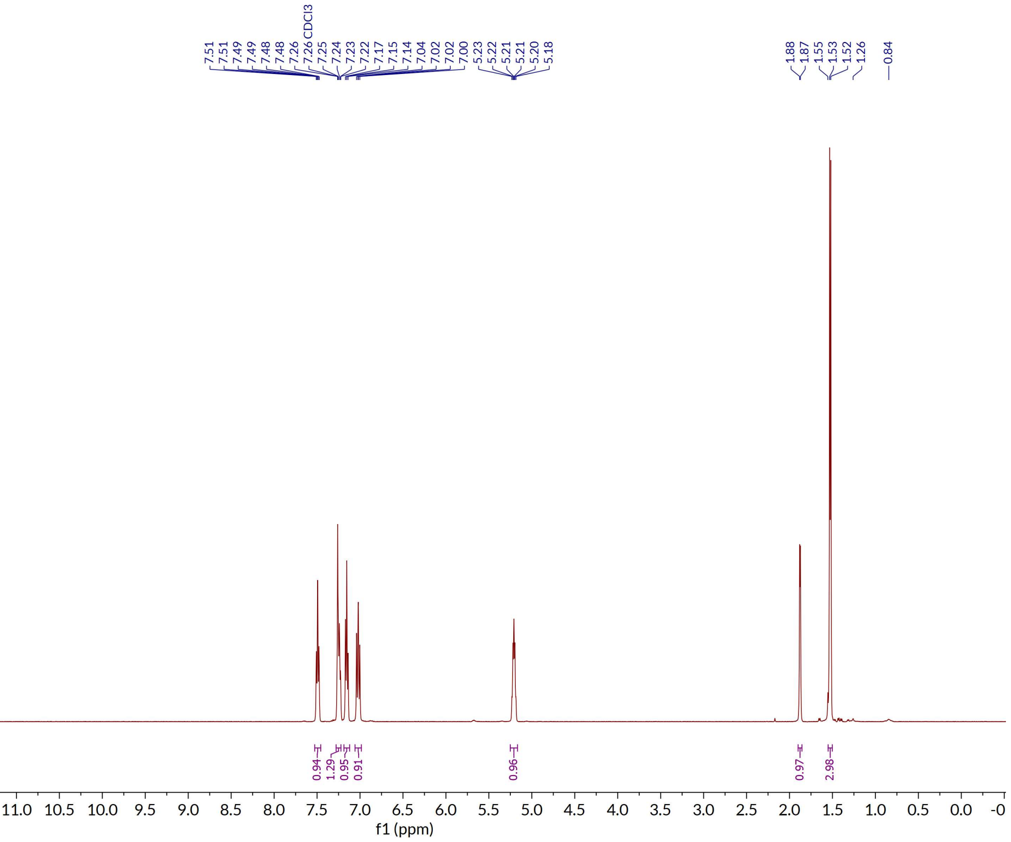

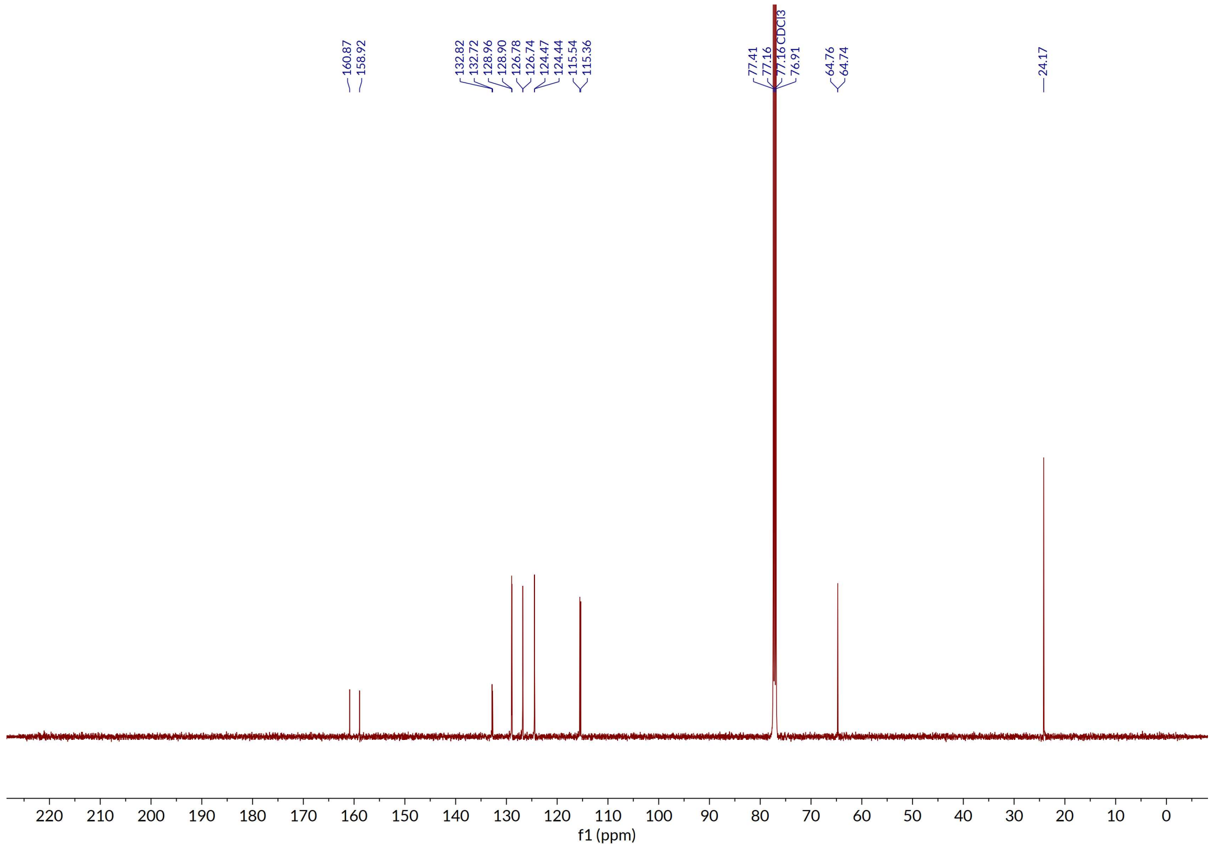

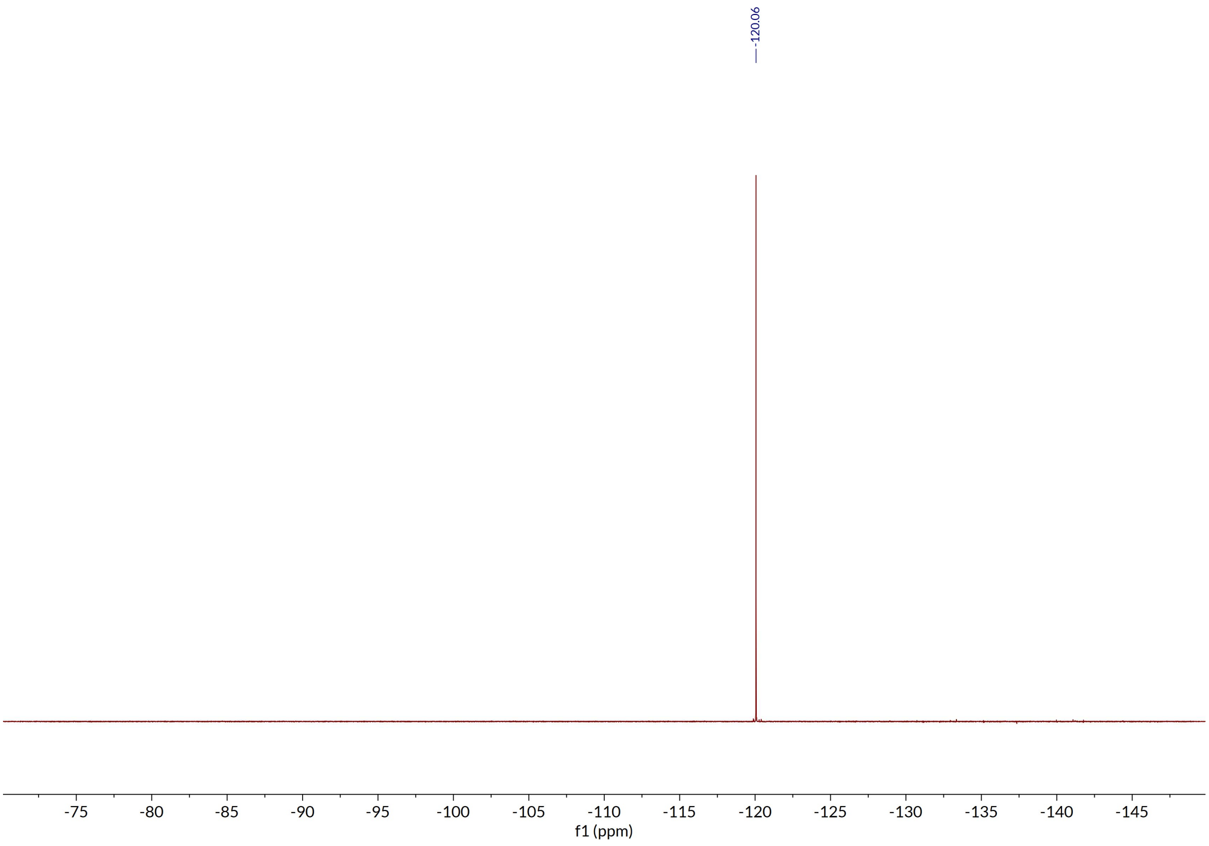

(*S*)-1-(4-Chlorophenyl)ethan-1-ol (4a)

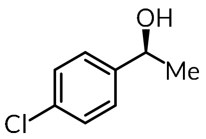

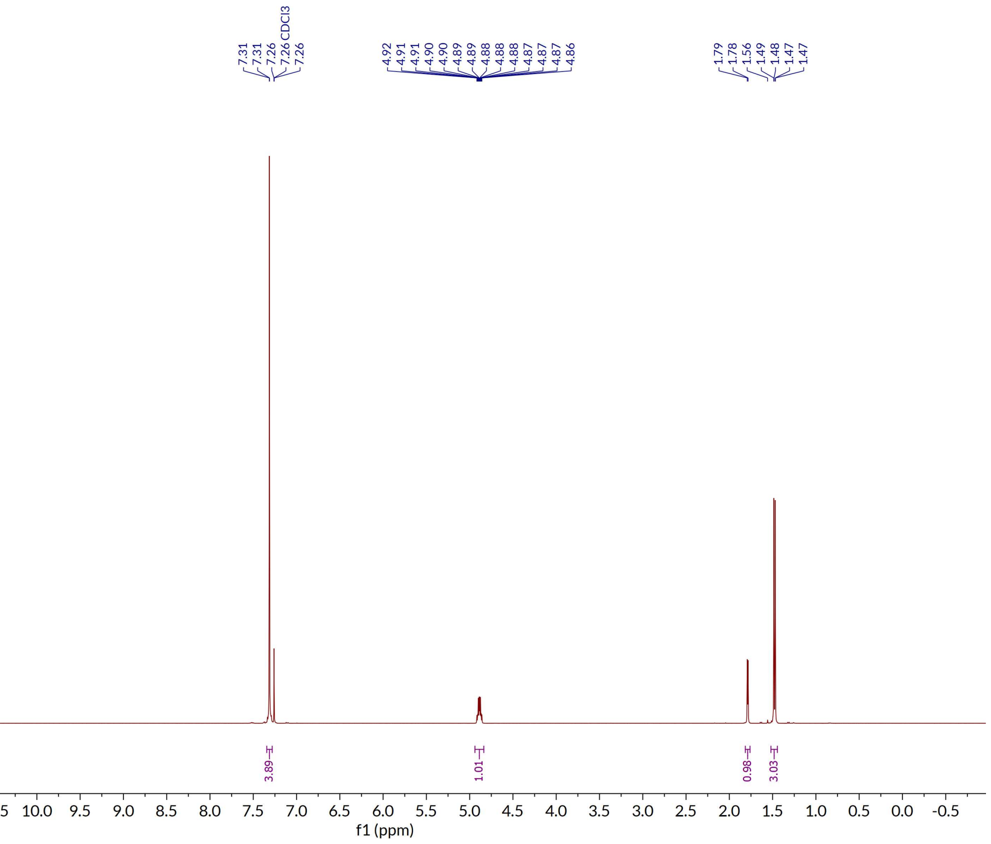

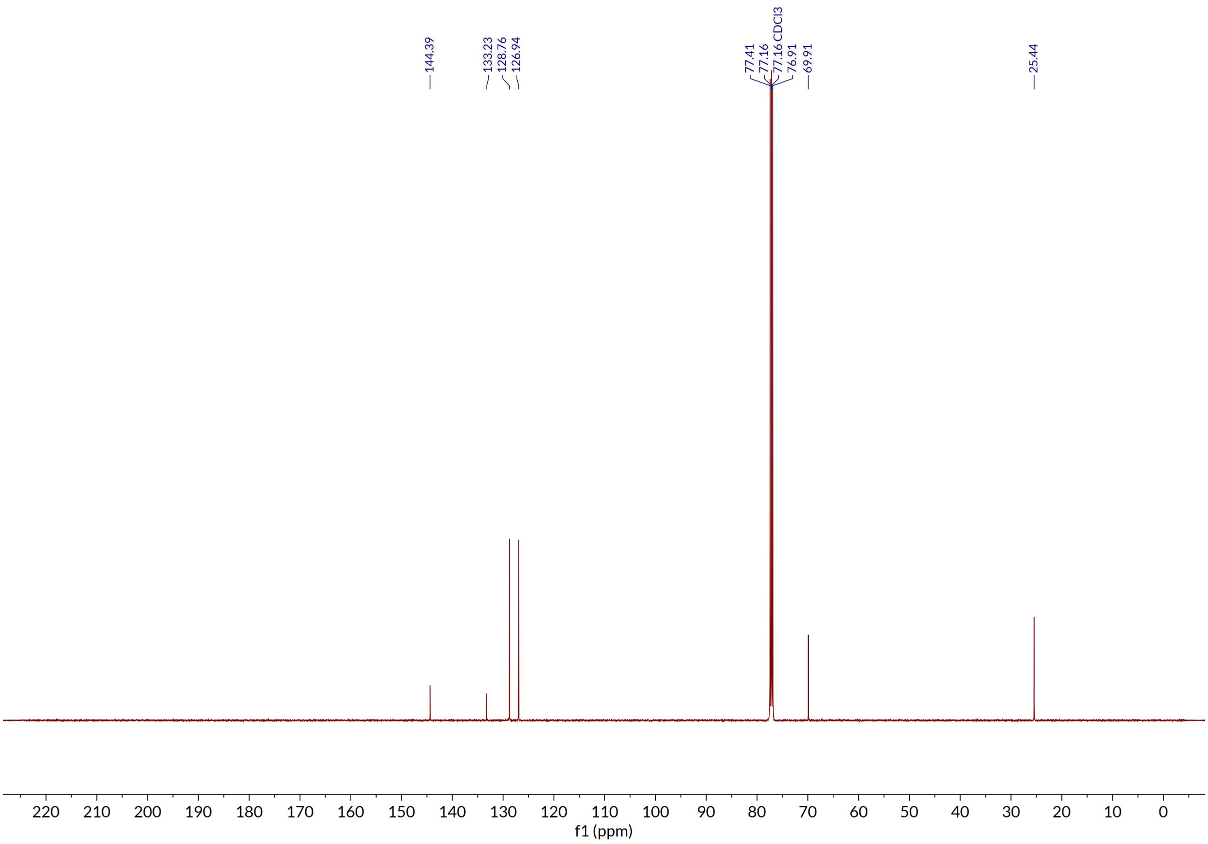

1-(4-Chlorophenyl)ethan-1-one (4b)

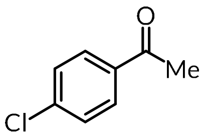

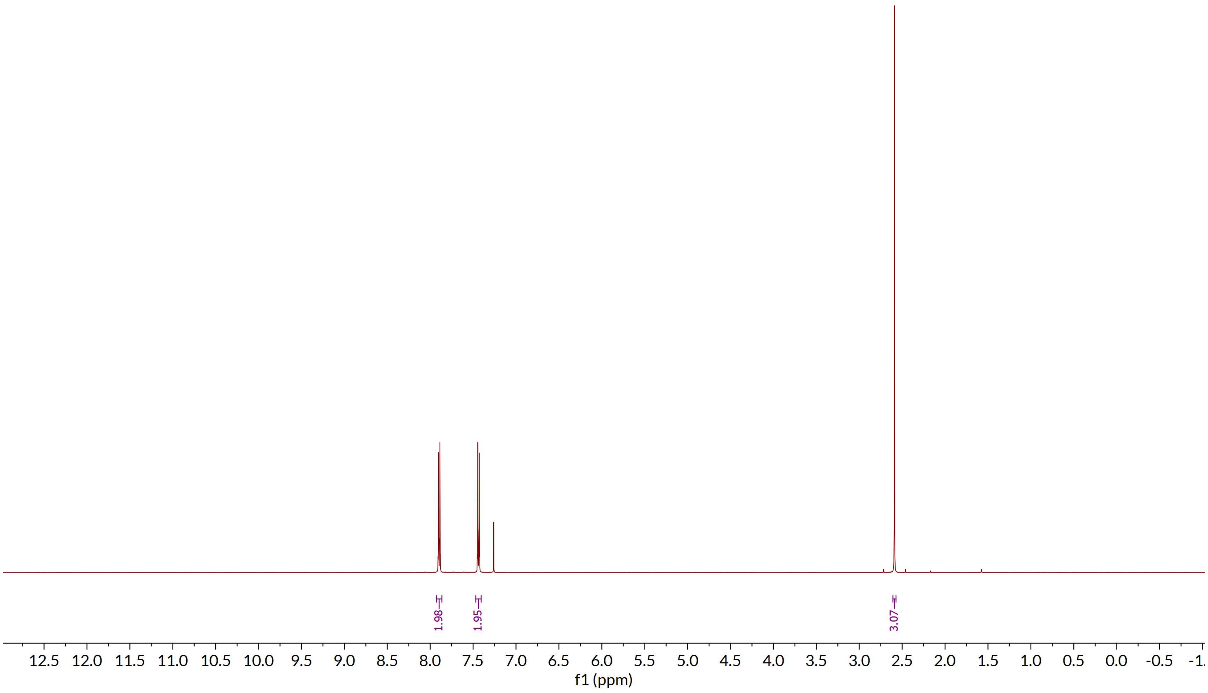

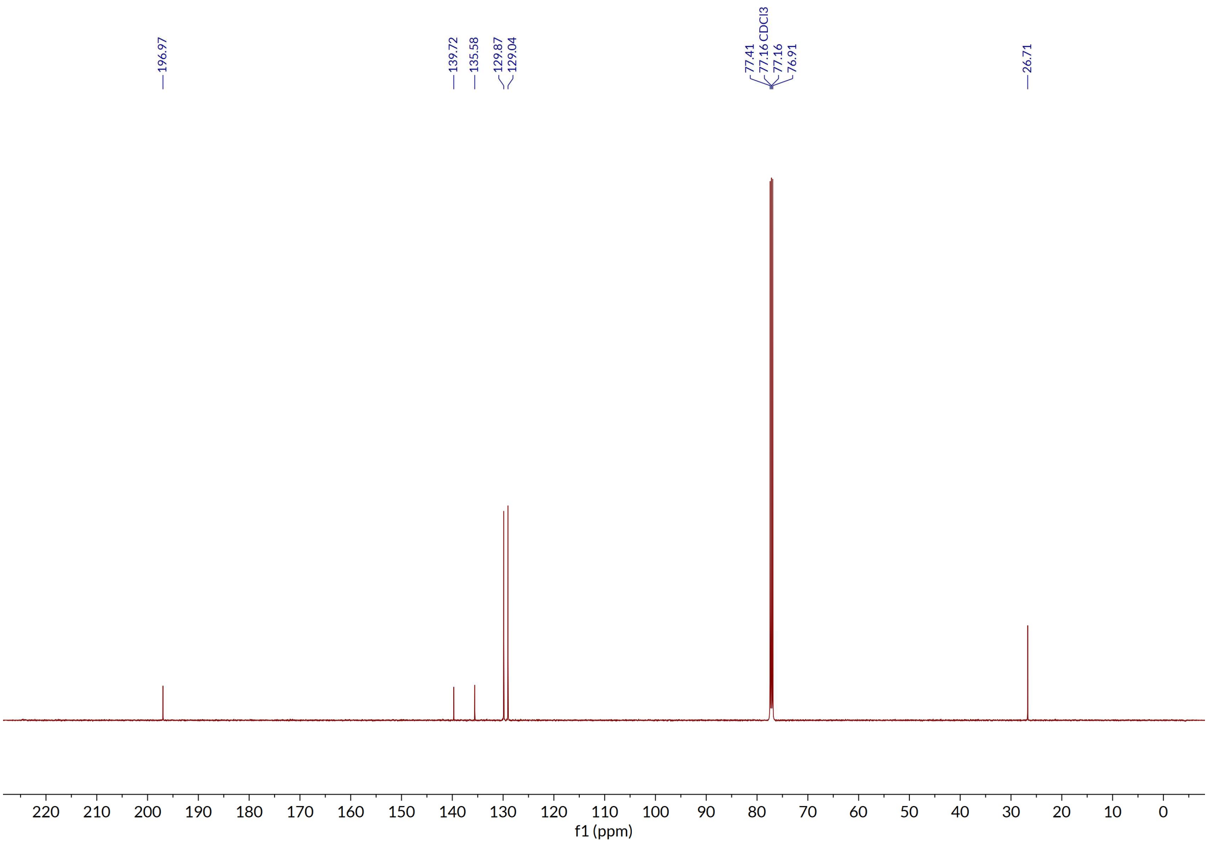

(*S*)-1-(3-Chlorophenyl)ethan-1-ol (5a)

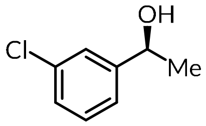

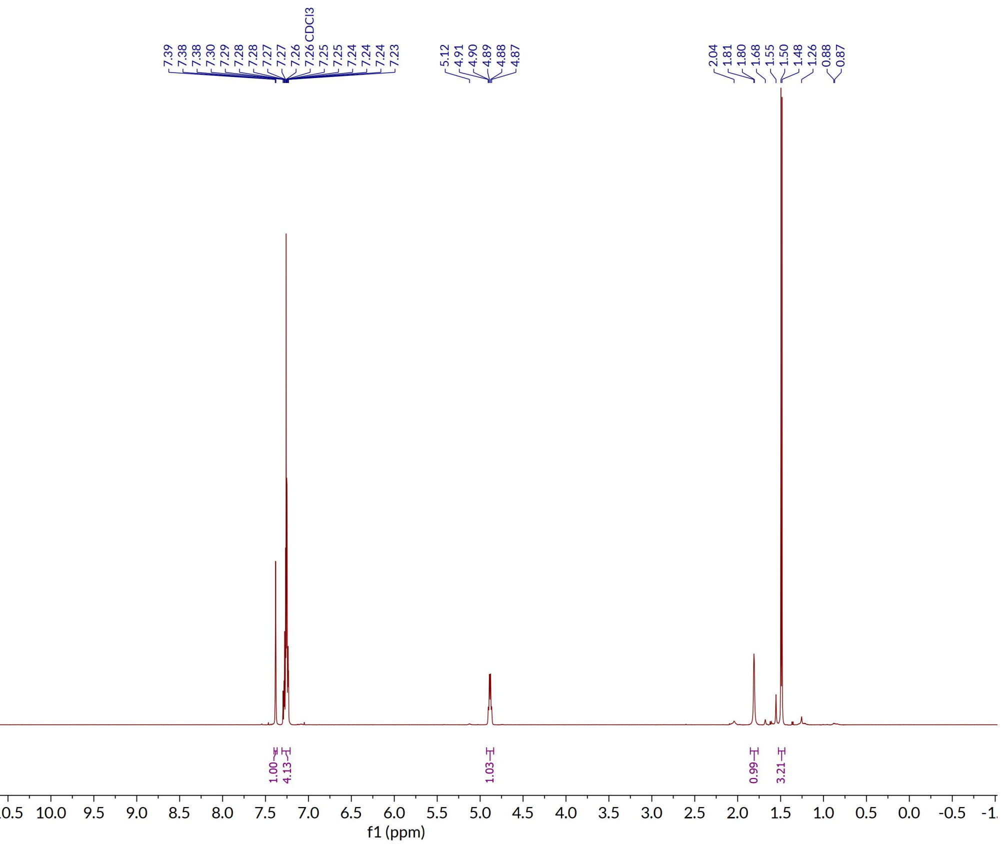

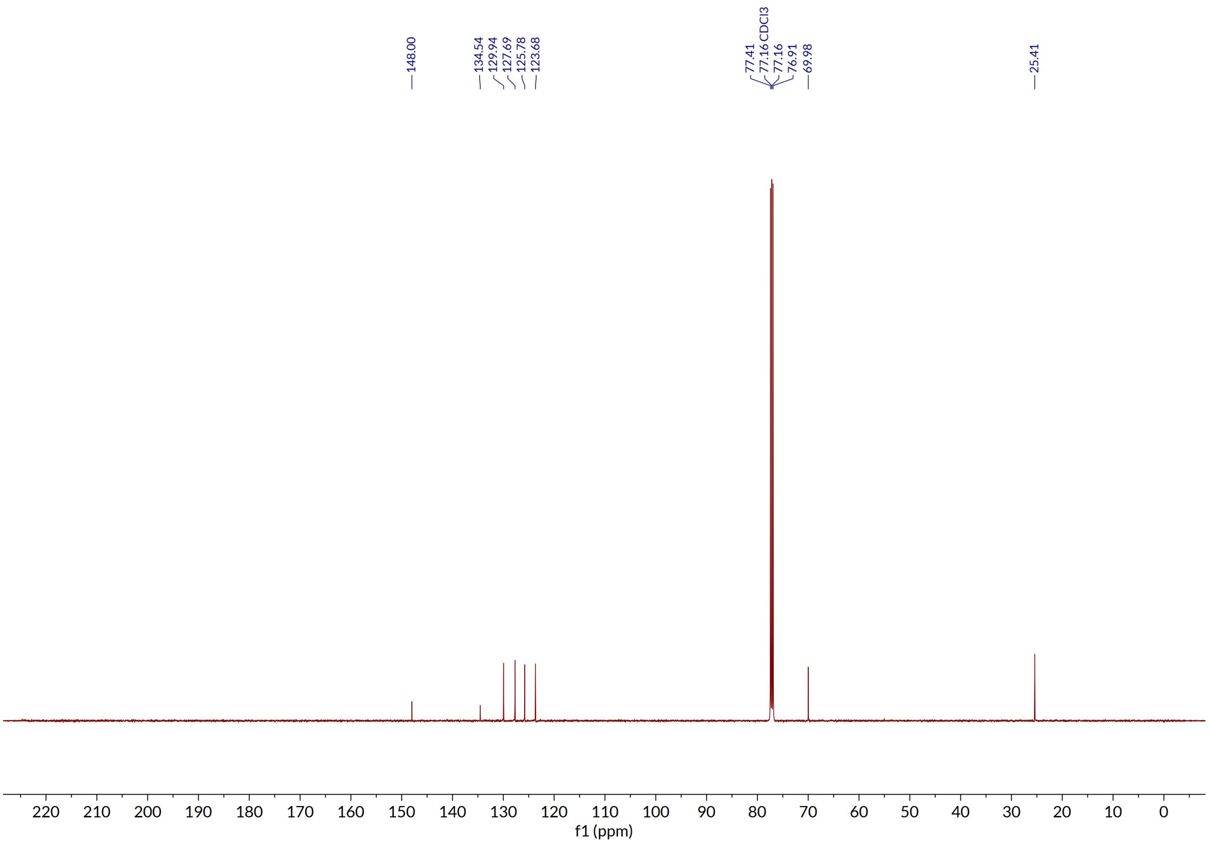

1-(3-Chlorophenyl)ethan-1-one (5b)

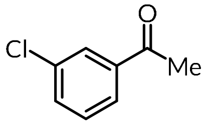

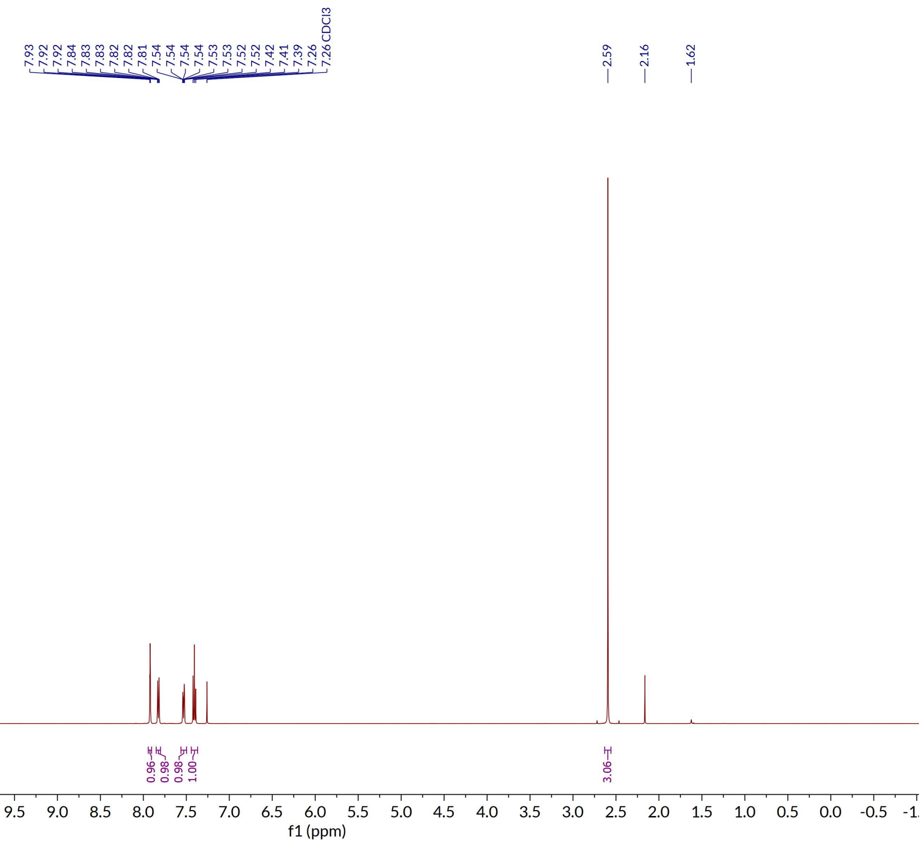

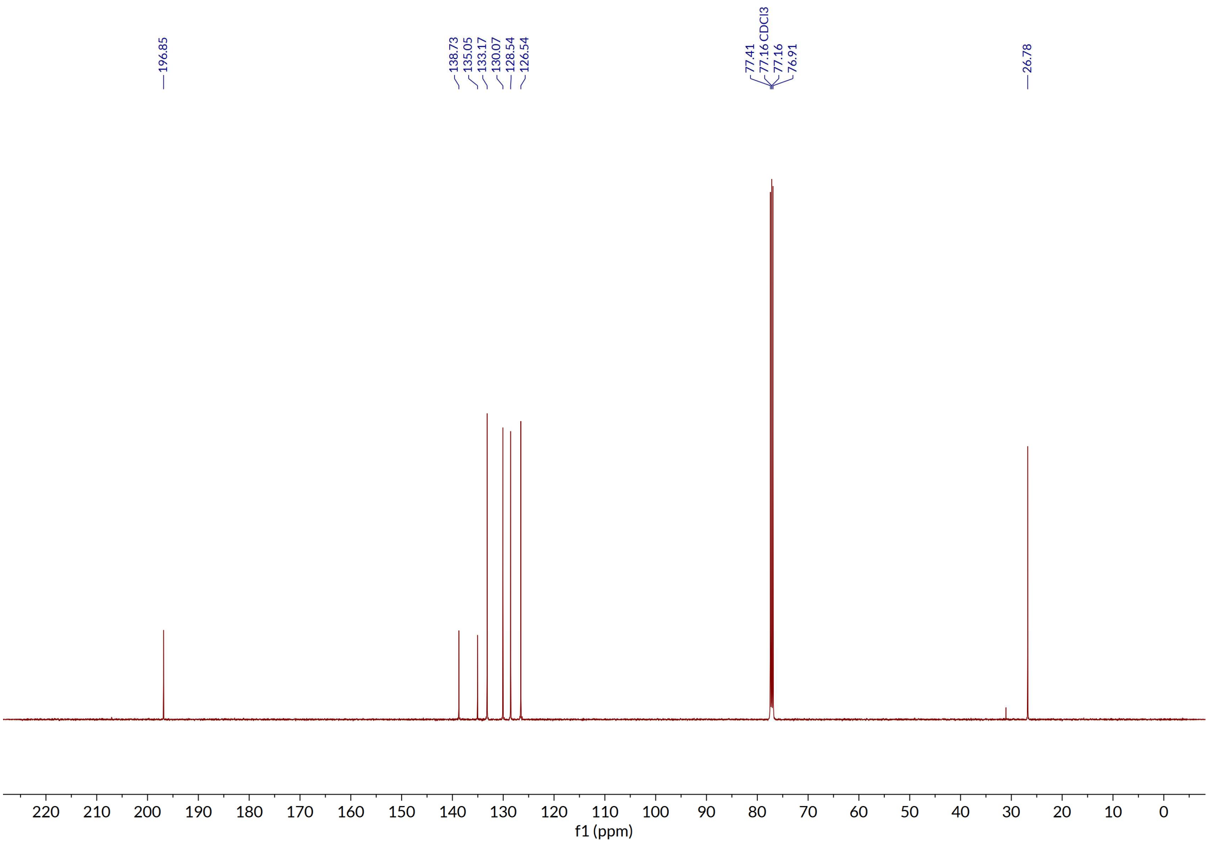

(*S*)-1-(2-Bromophenyl)ethan-1-ol (6a)

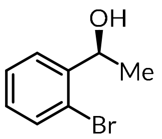

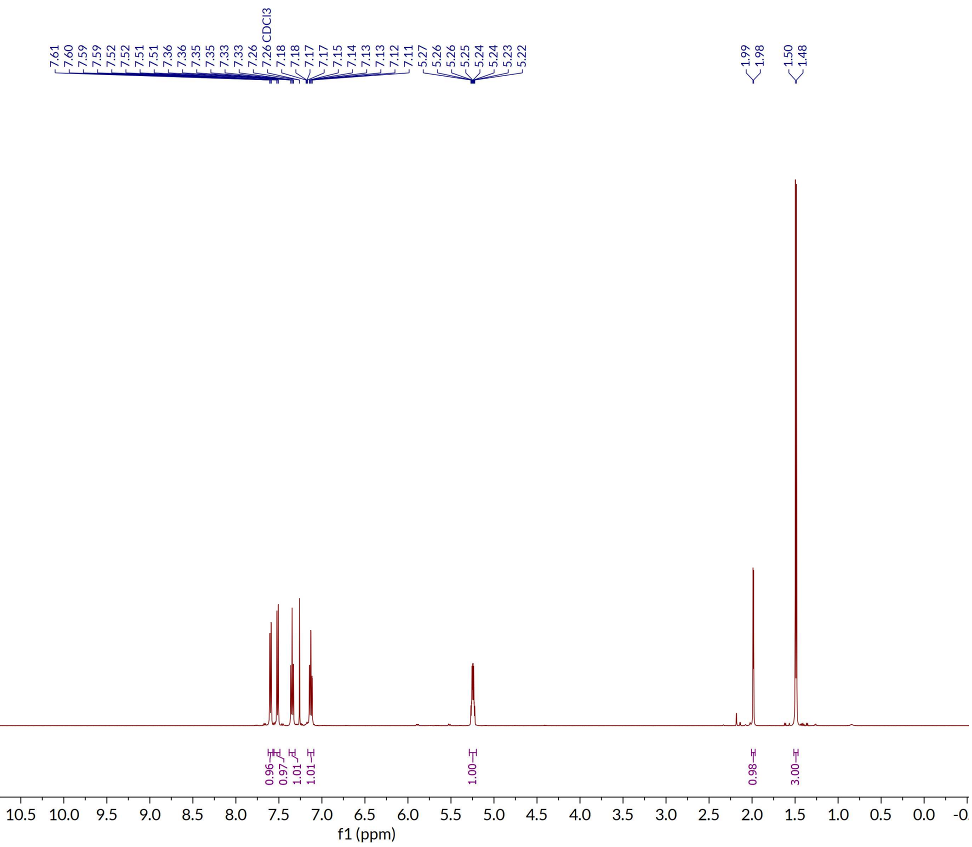

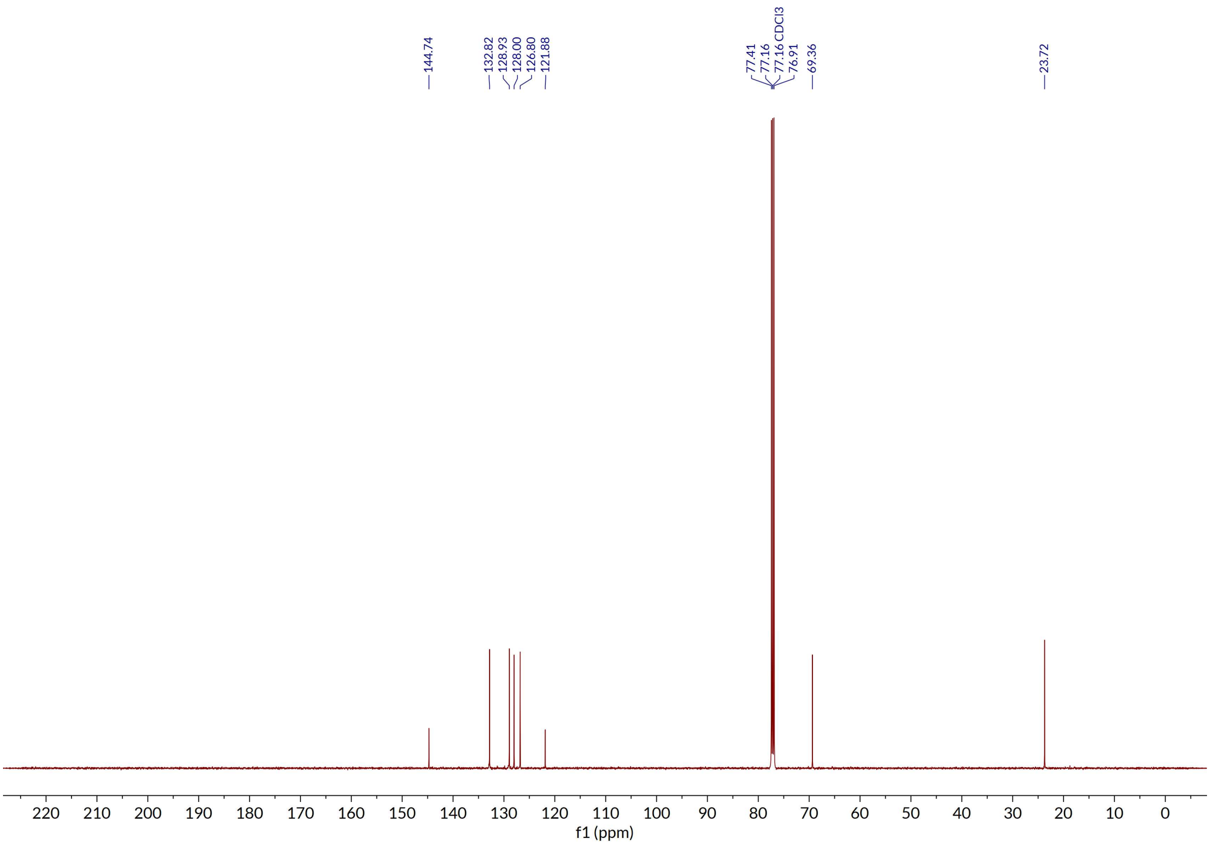

(*S*)-1-(3-Bromophenyl)ethan-1-ol (7a)

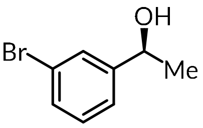

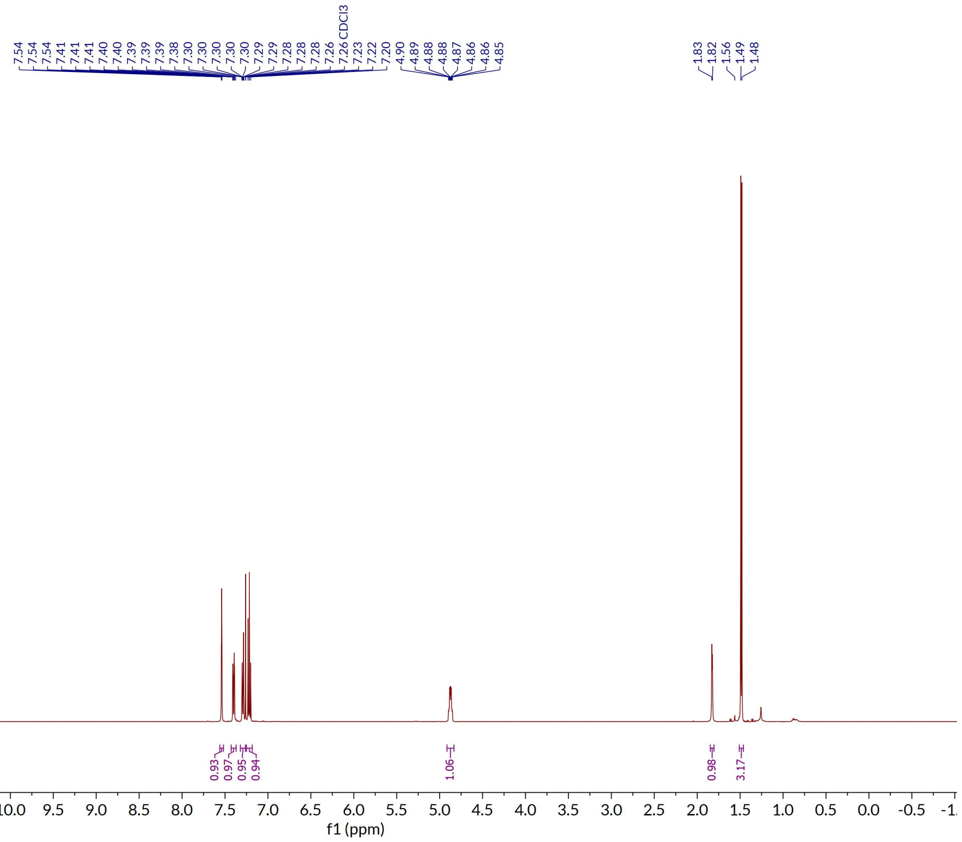

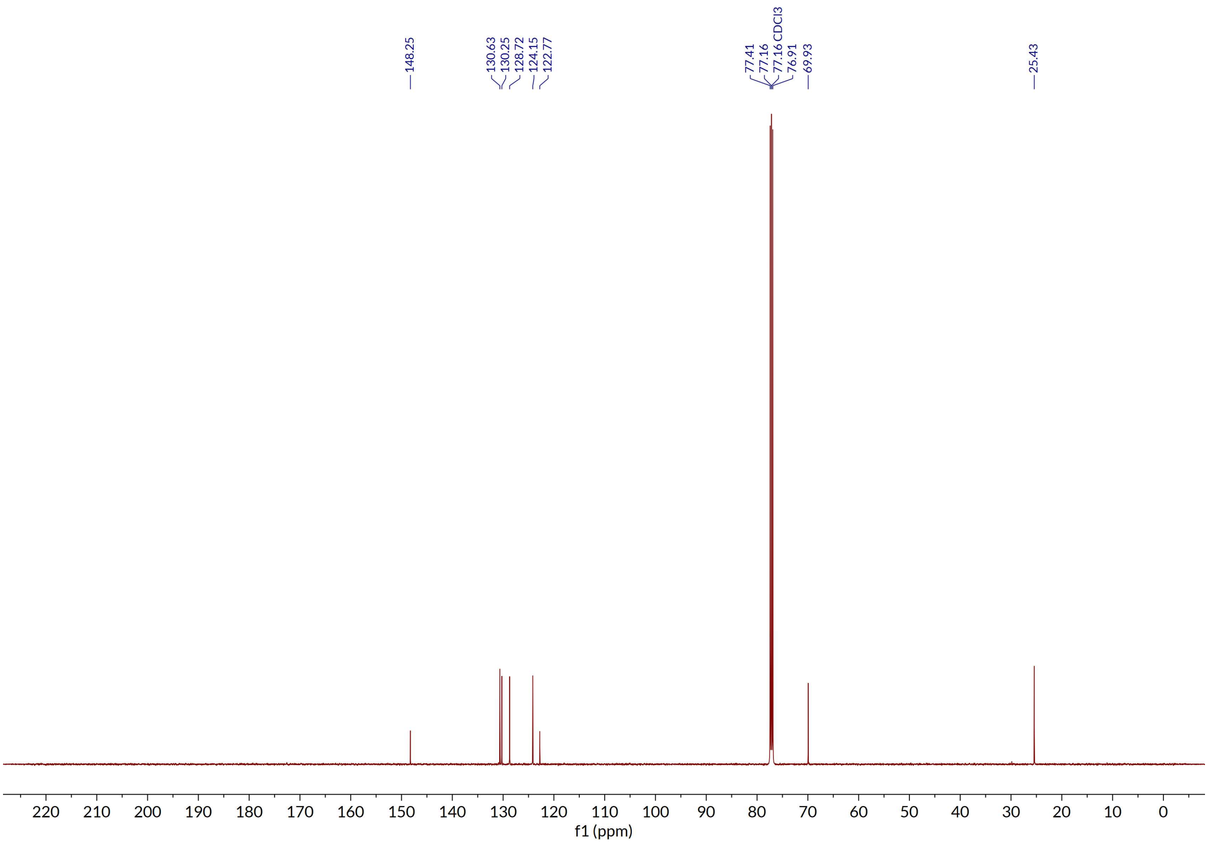

(*S*)-1-(4-Bromophenyl)ethan-1-ol (8a)

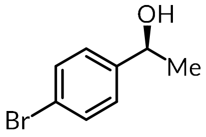

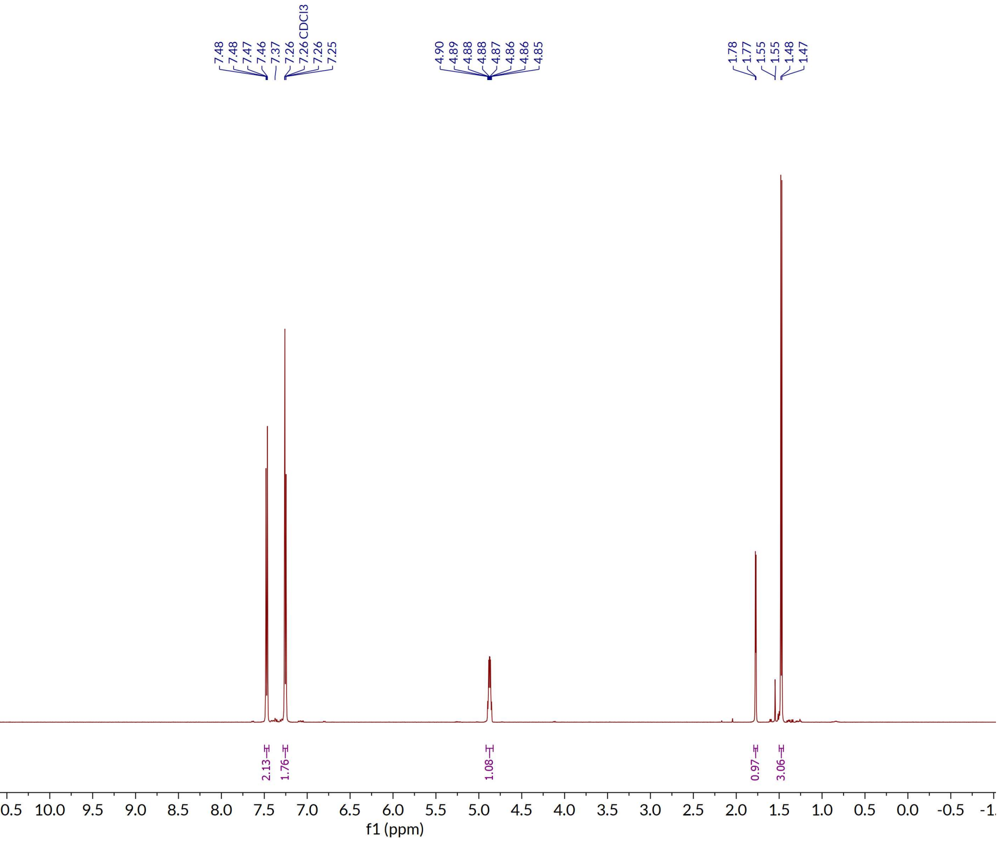

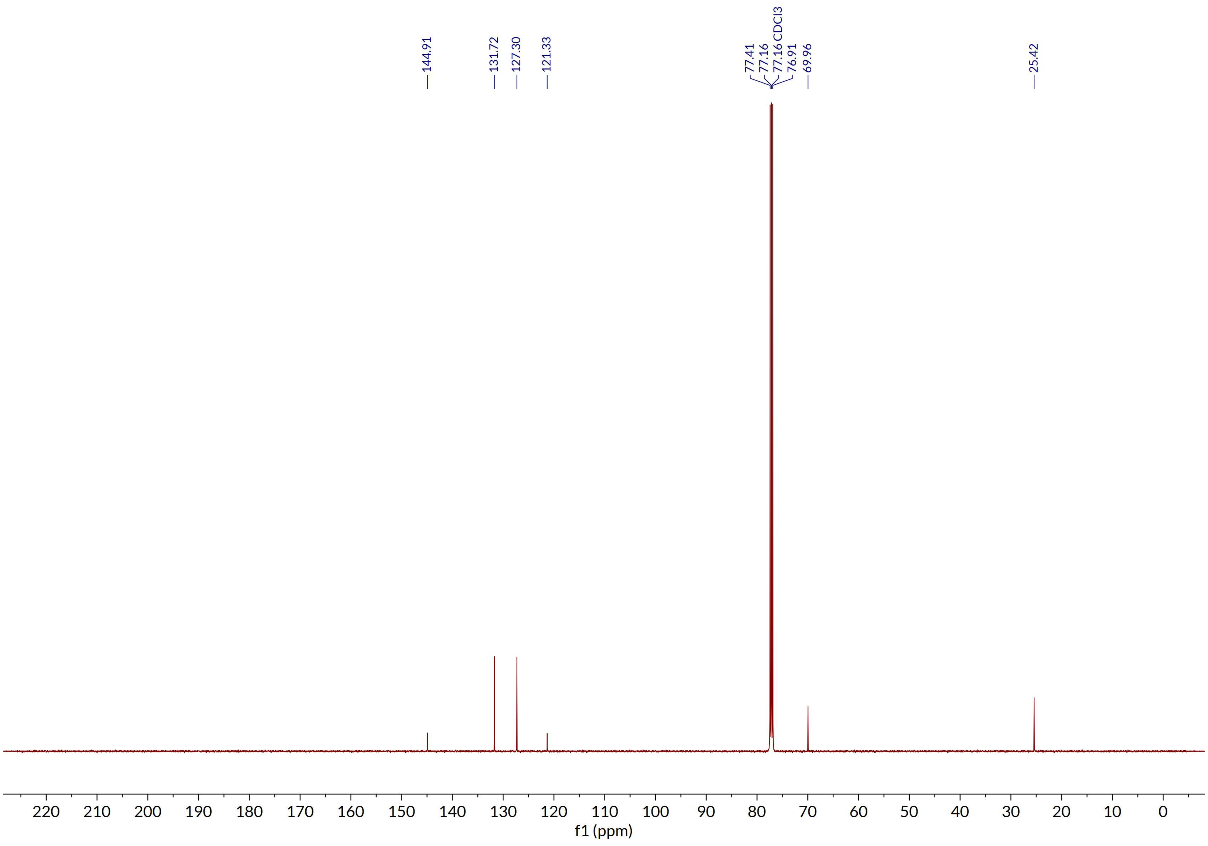

1-(4-Bromophenyl)ethan-1-one (8b)

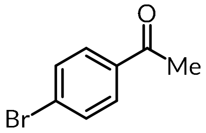

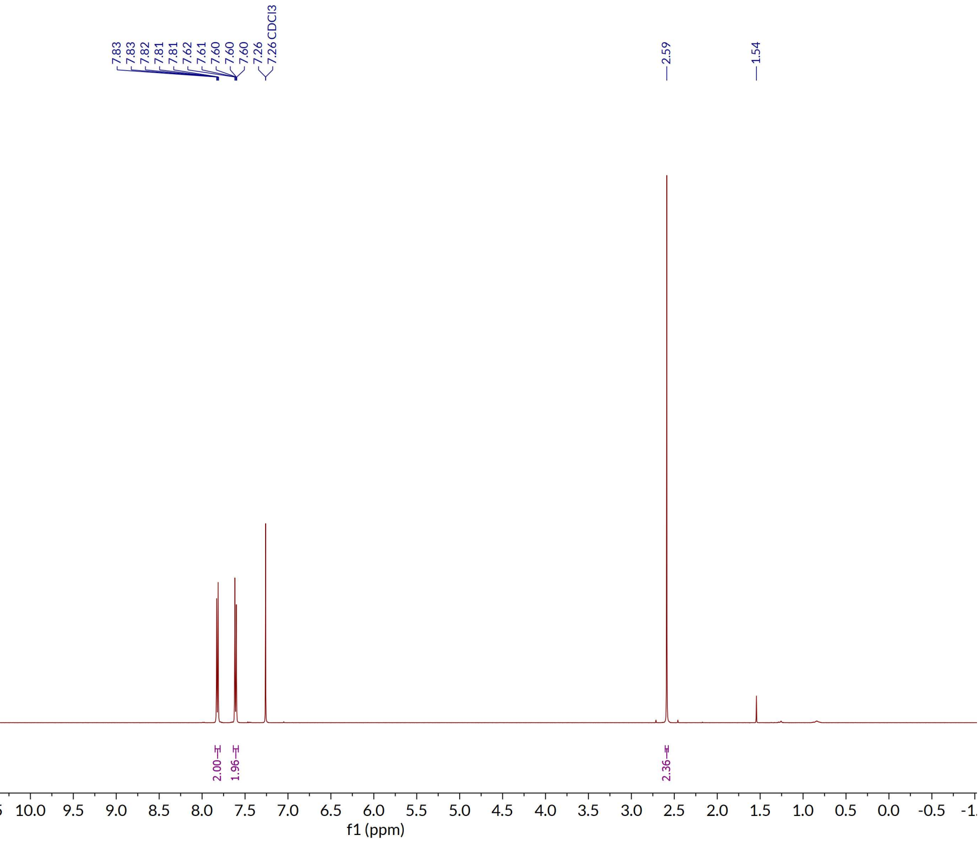

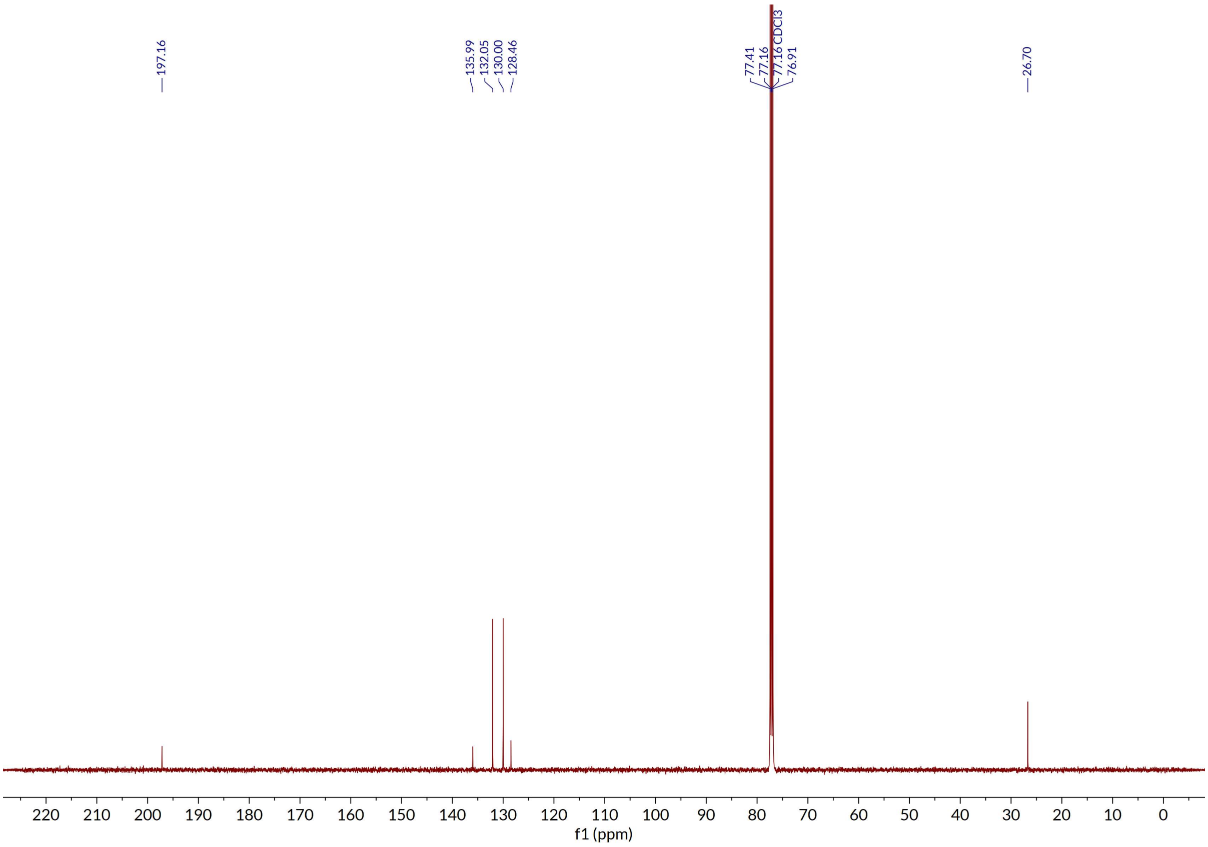

(*S*)-1-(2-Iodophenyl)ethan-1-ol (9a)

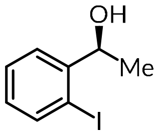

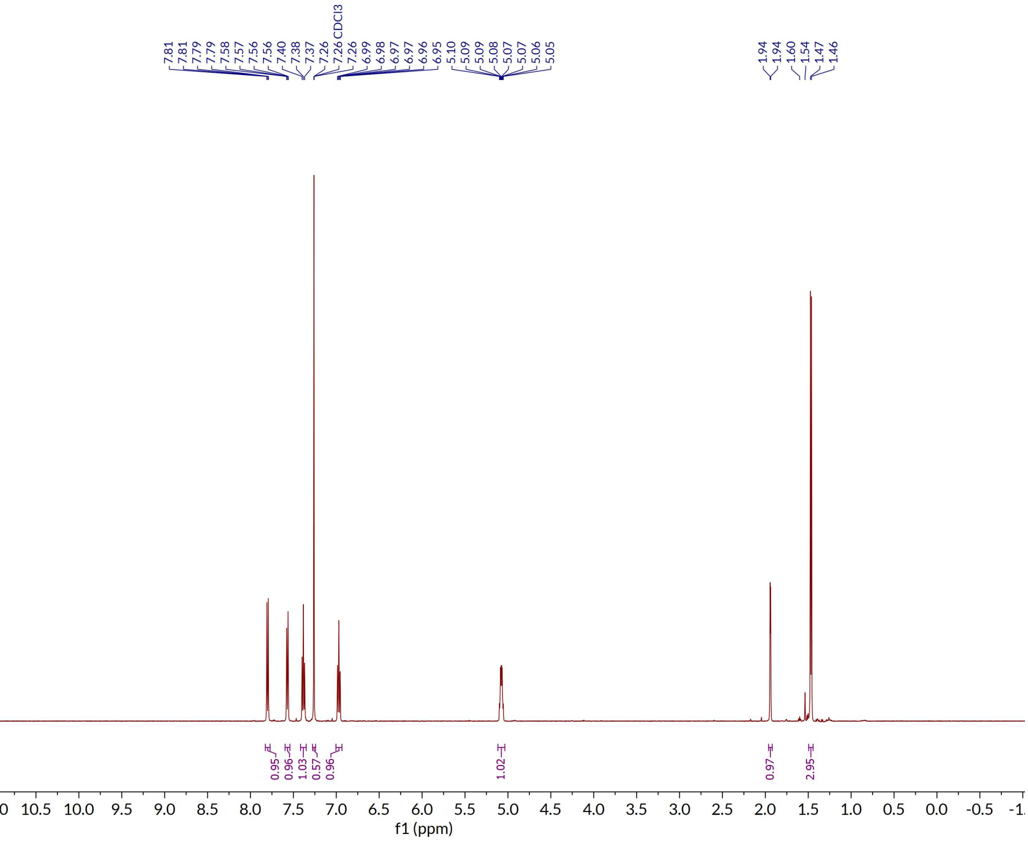

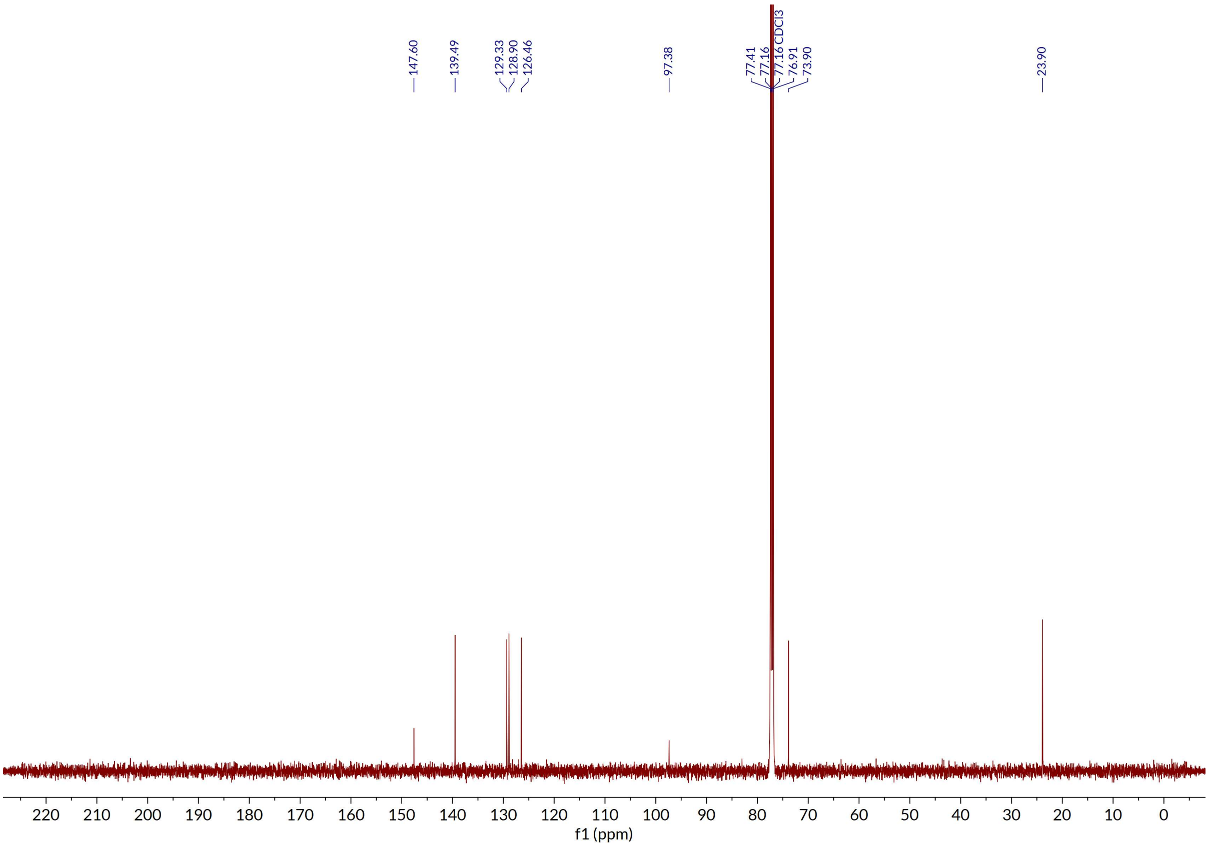

1-(2-Iodophenyl)ethan-1-one (9b)

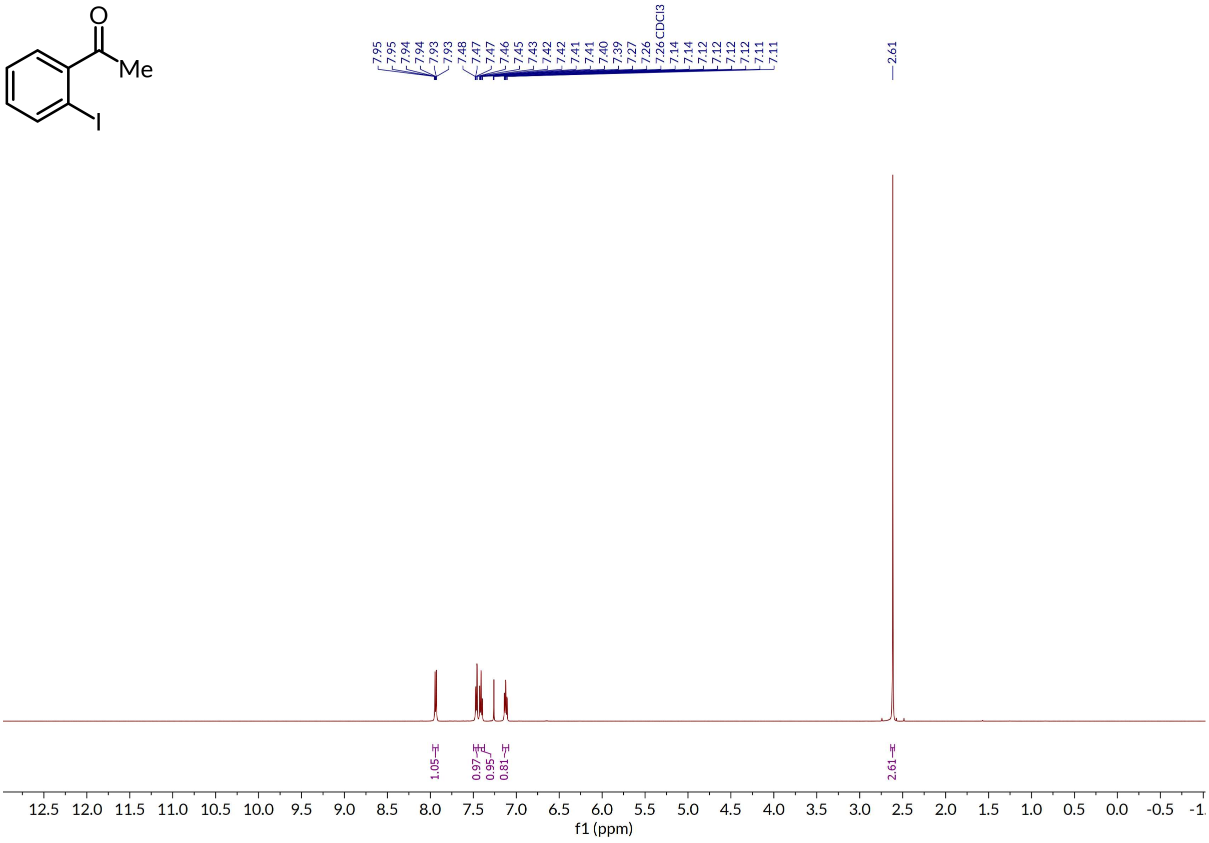

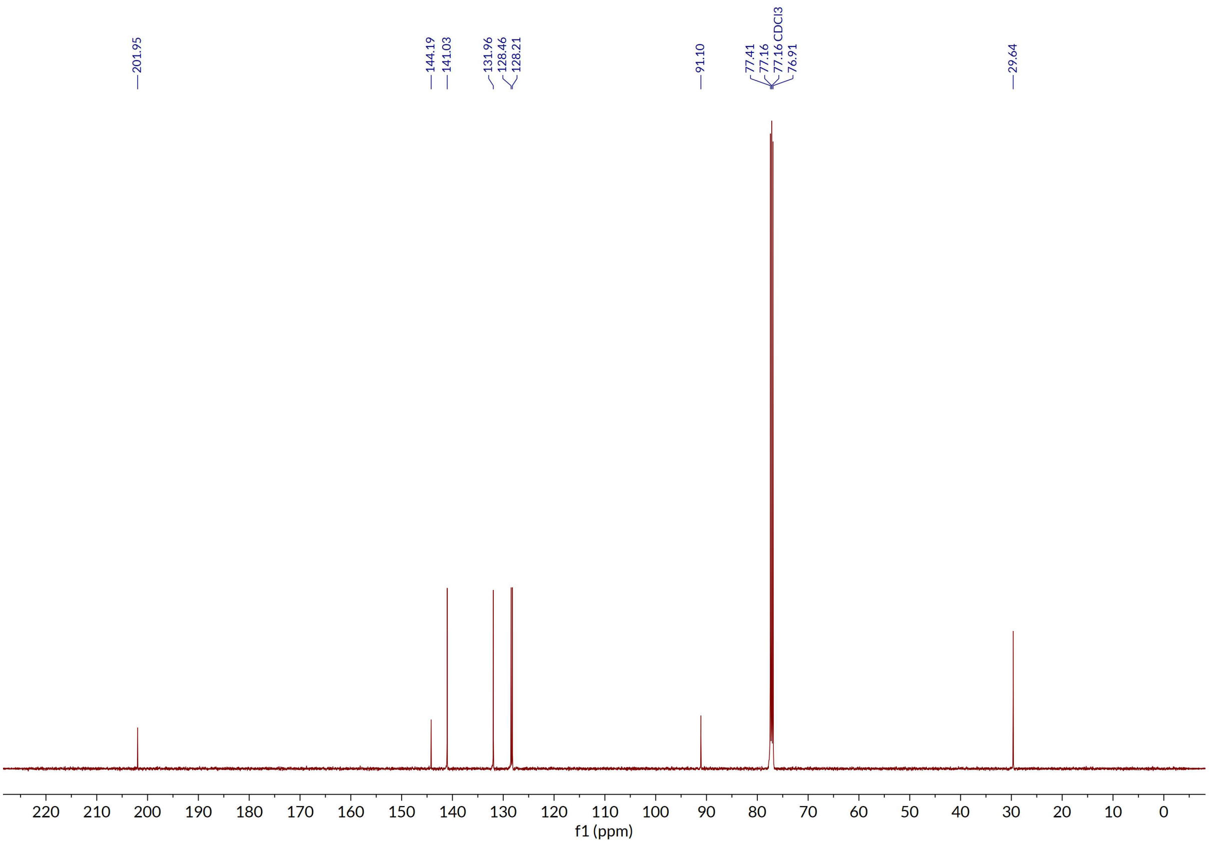

(*S*)-1-(4-Iodophenyl)ethan-1-ol (10a)

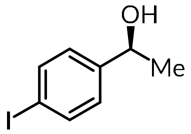

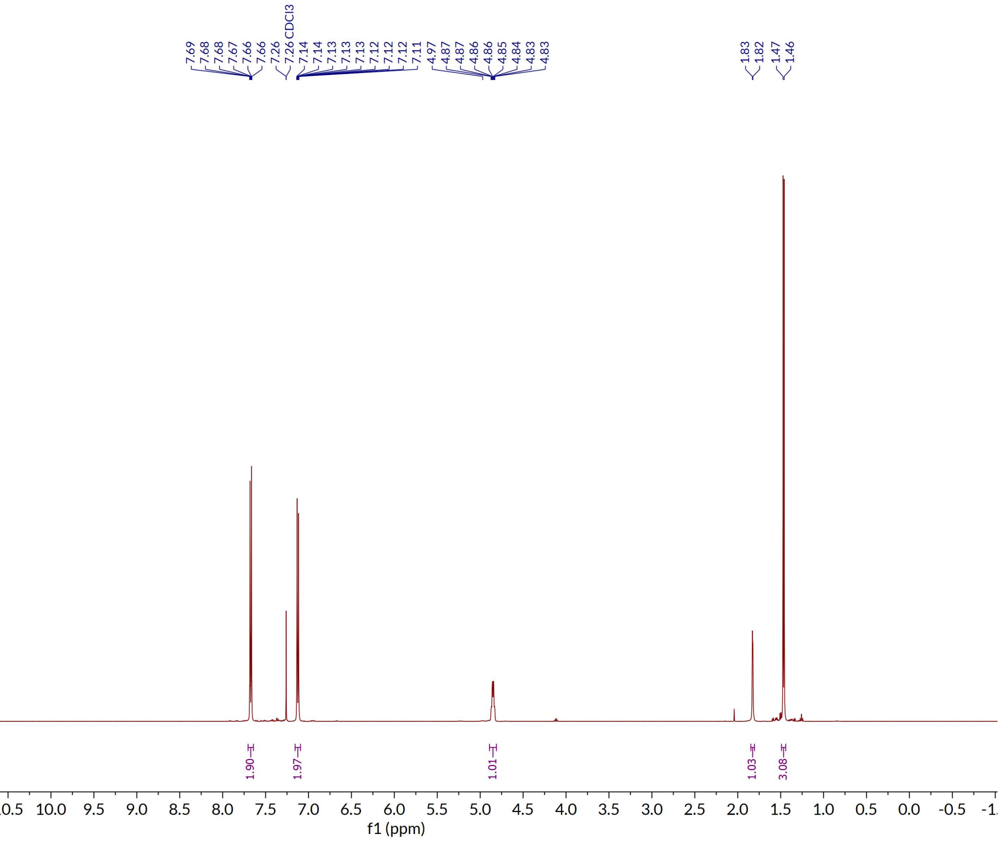

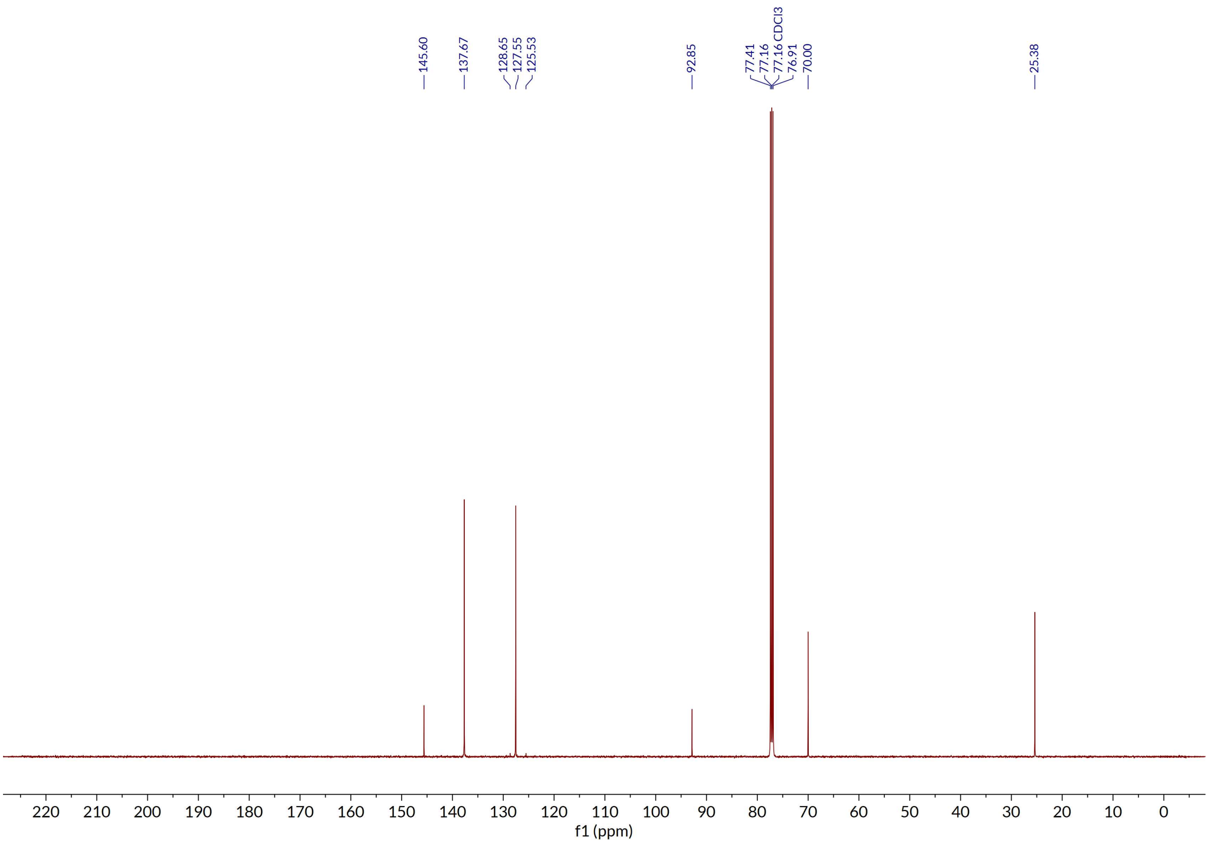

1-(4-Iodophenyl)ethan-1-one (10b)

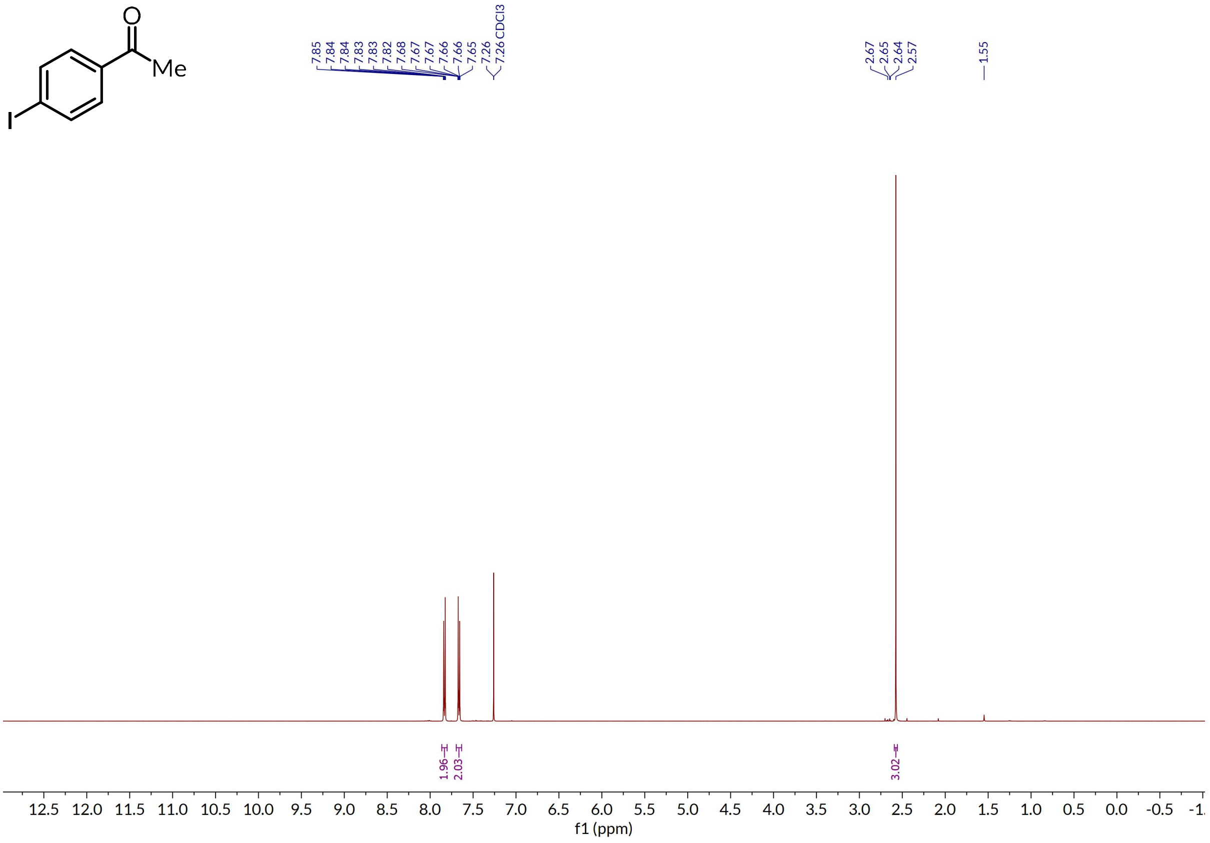

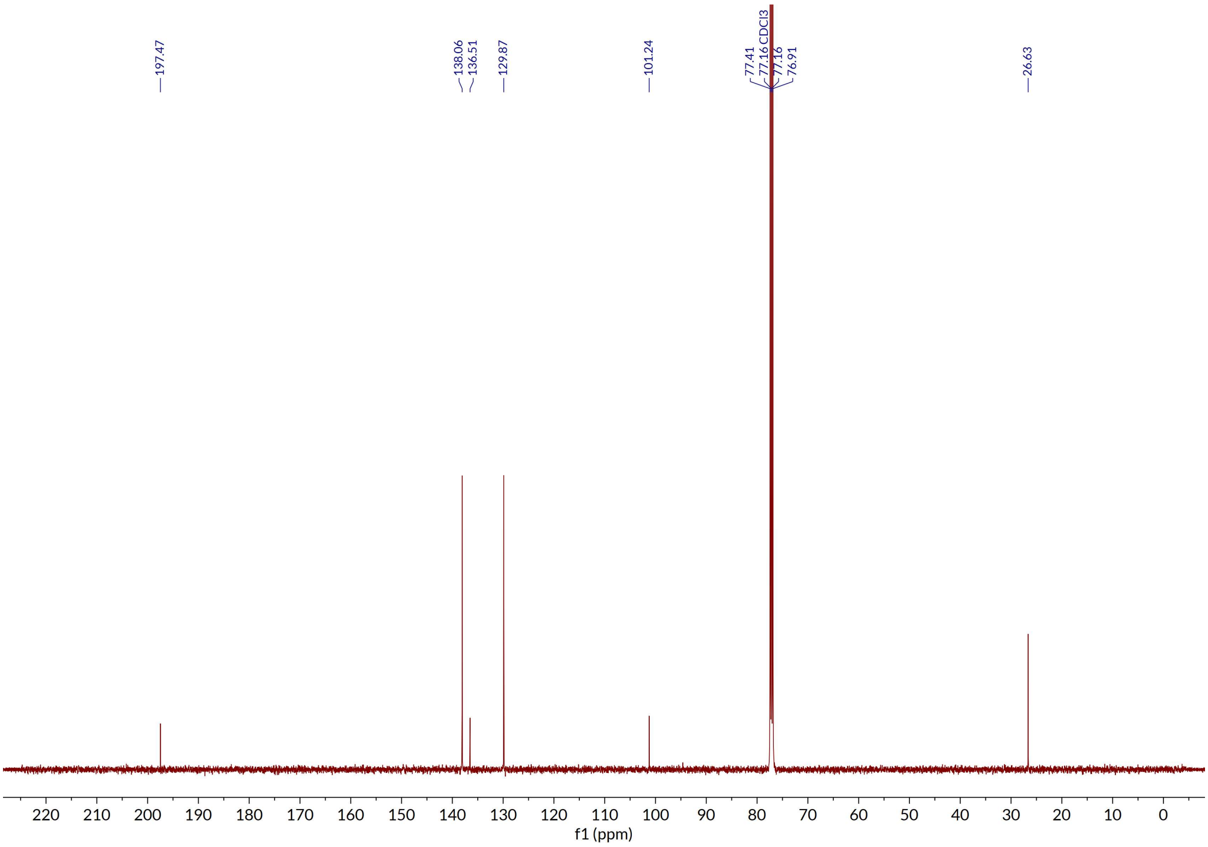

(*S*)-1-(3-Methoxyphenyl)ethan-1-ol (11a)

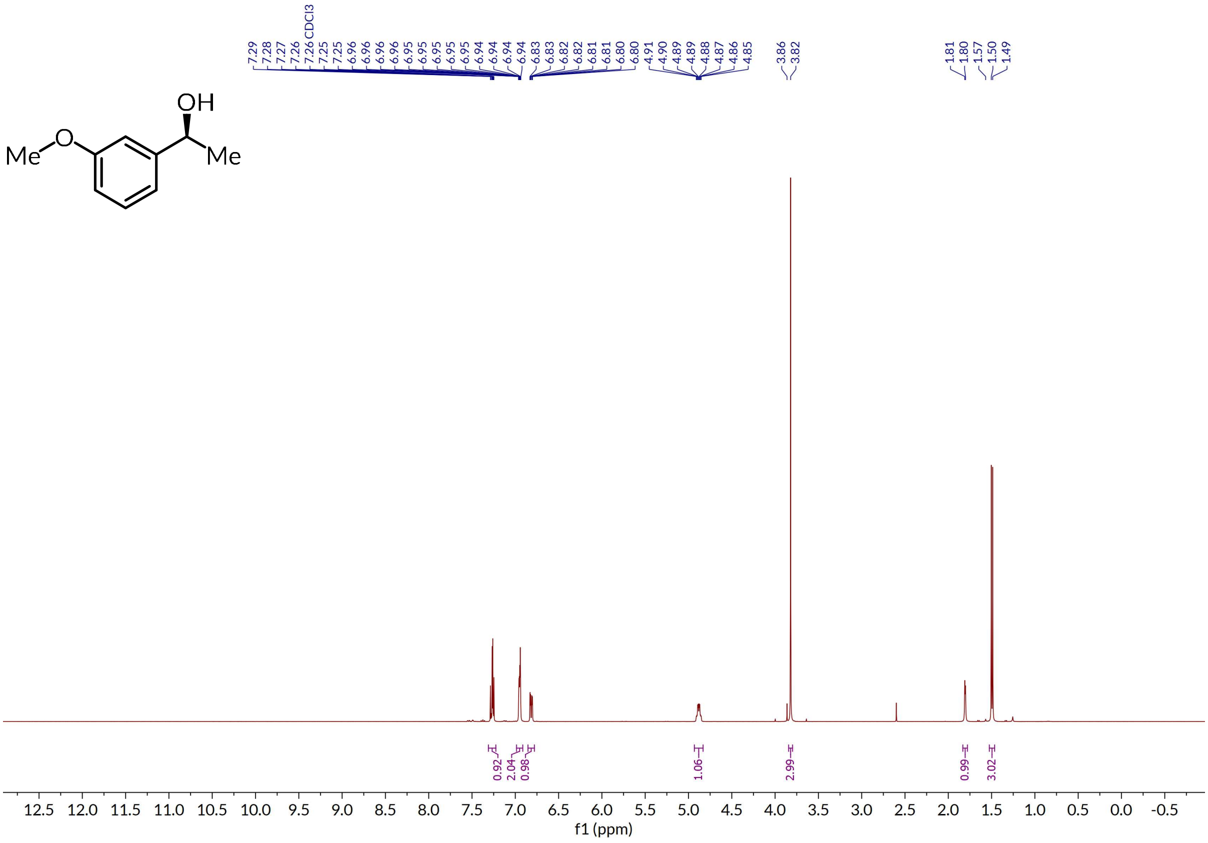

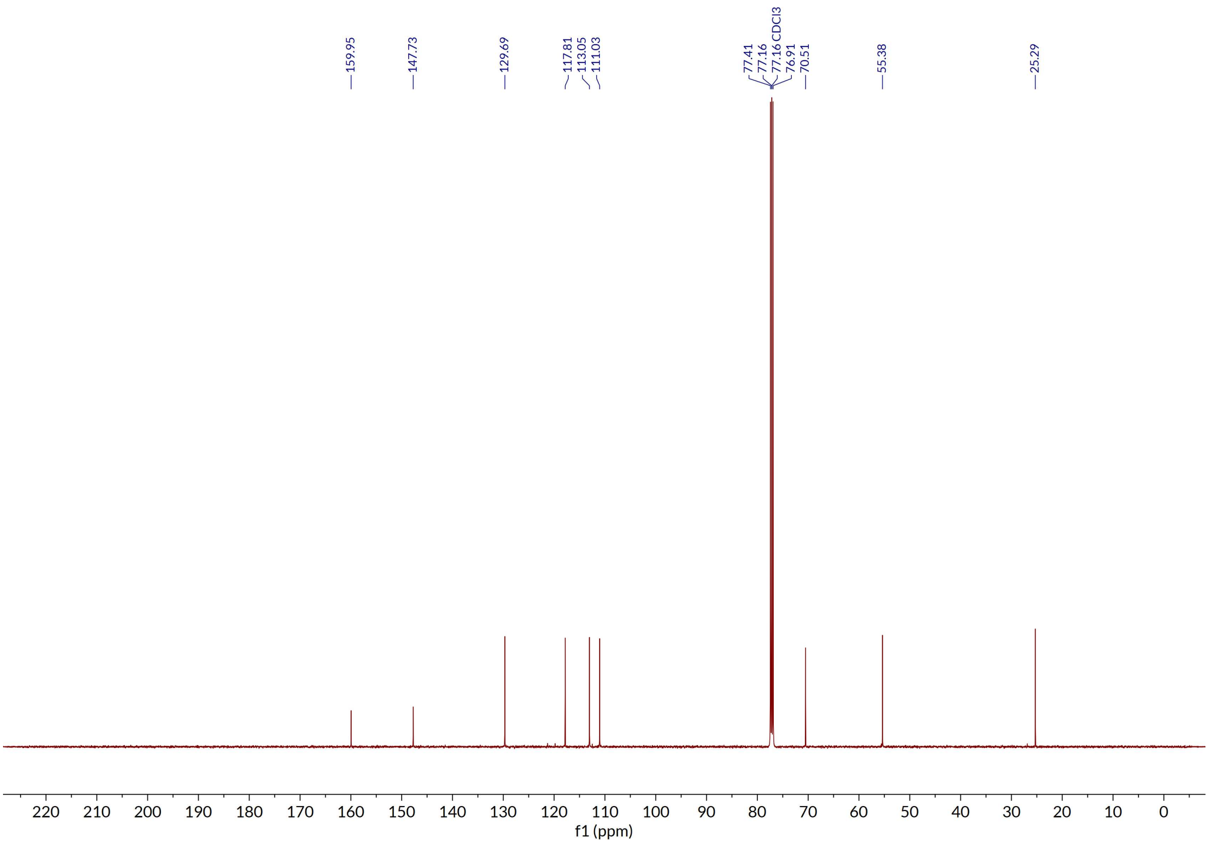

1-(3-Methoxyphenyl)ethan-1-one (11b)

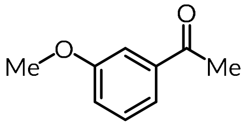

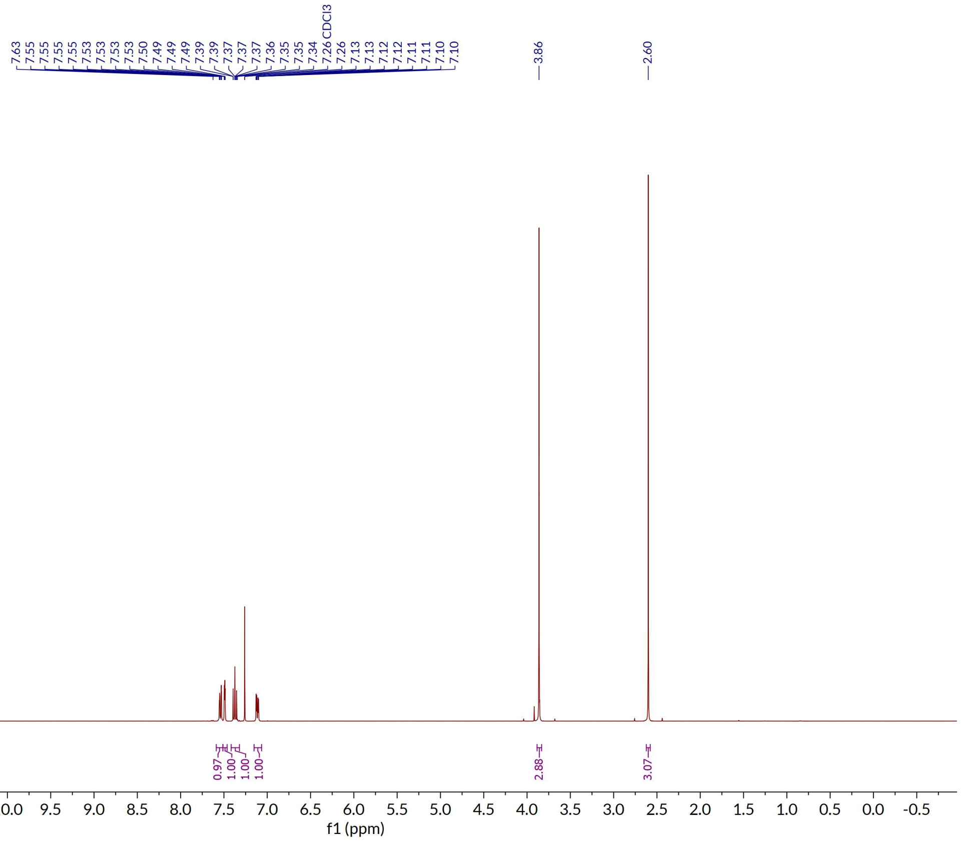

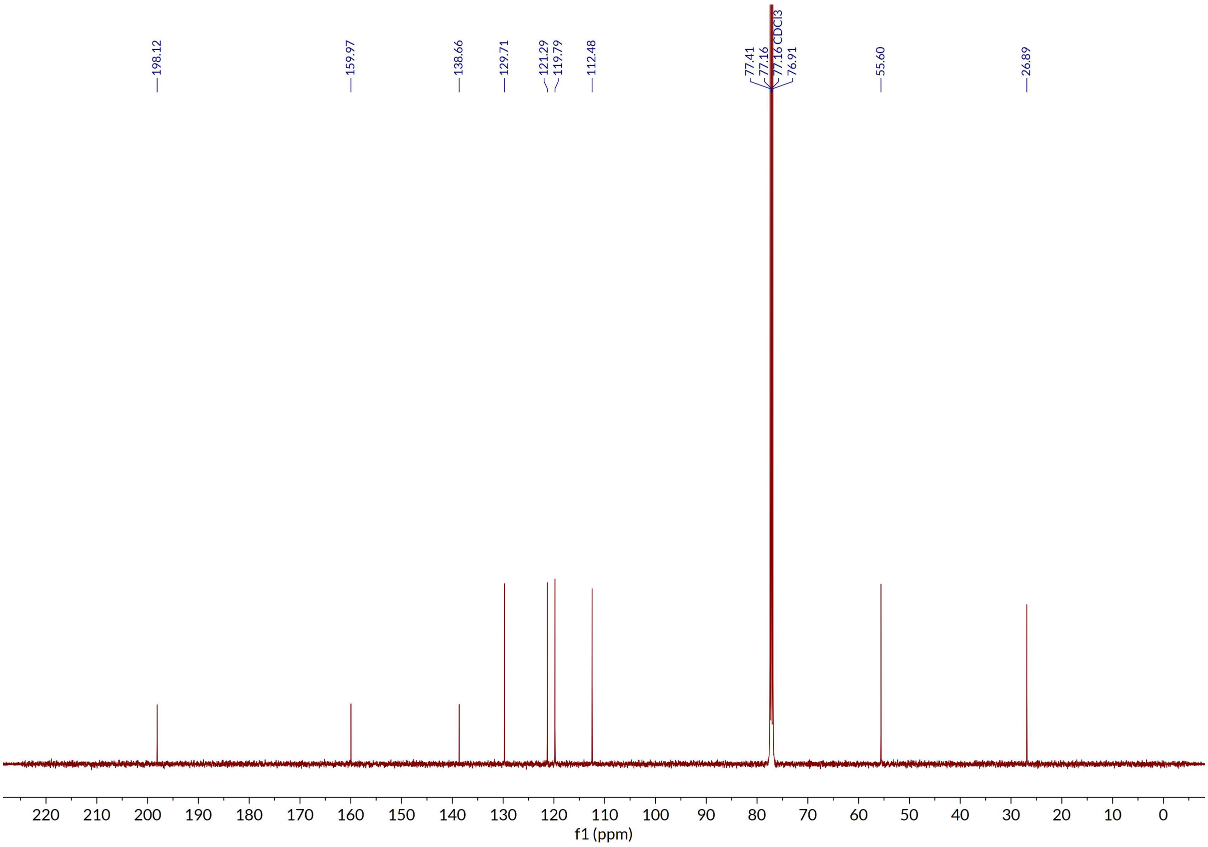

(*S*)-1-(4-Methoxyphenyl)ethan-1-ol (12a)

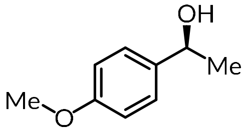

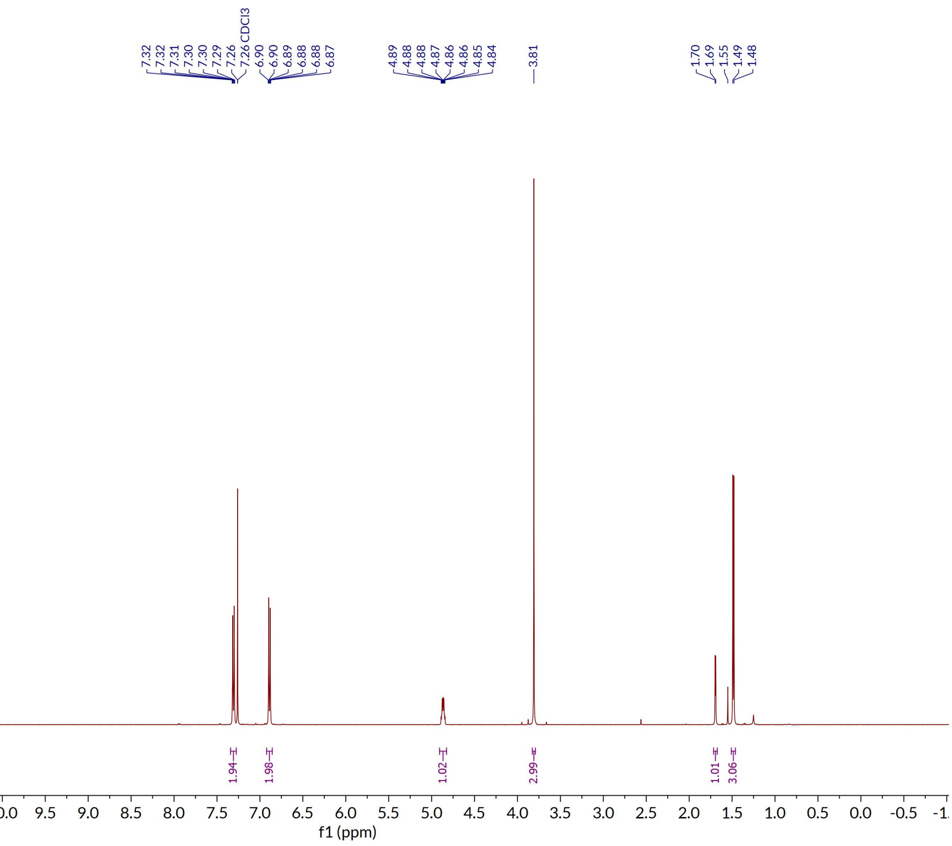

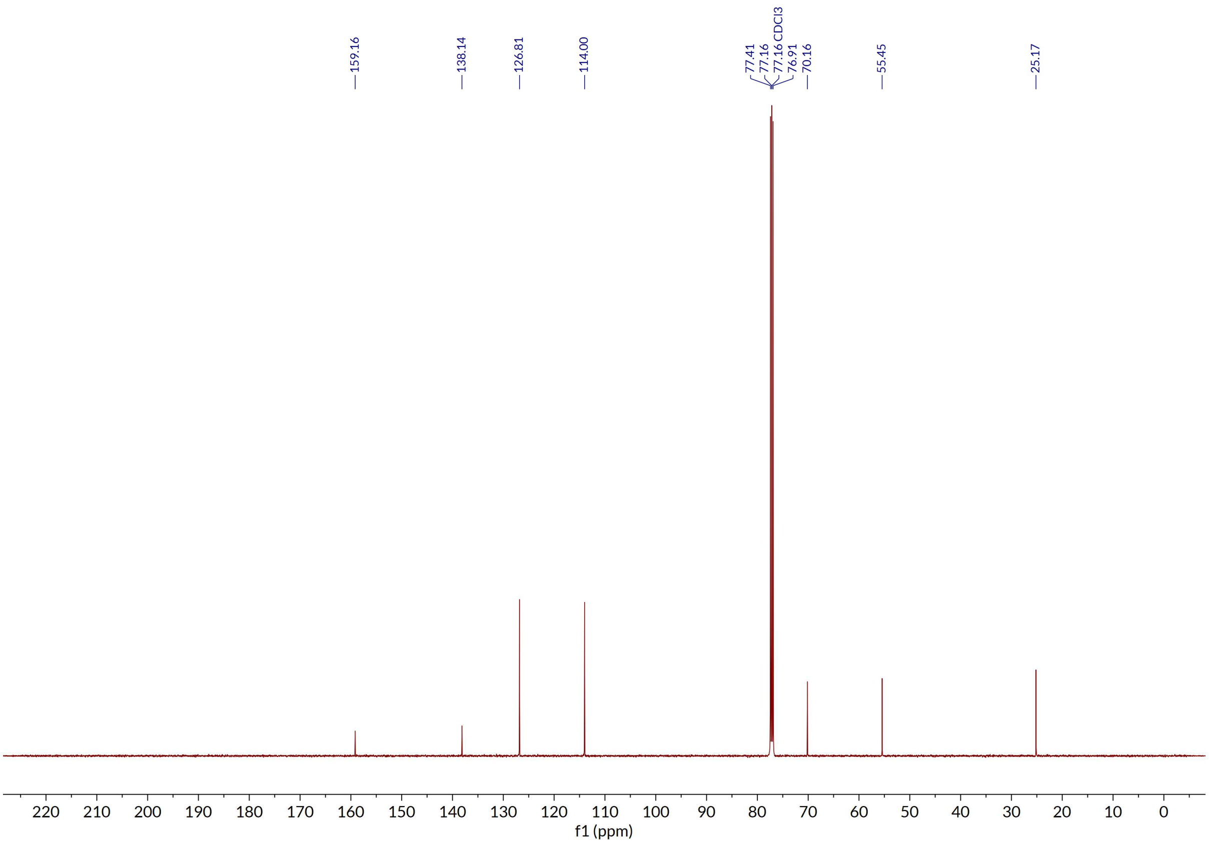

1-(4-Methoxyphenyl)ethan-1-one (12b)

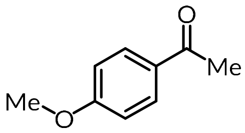

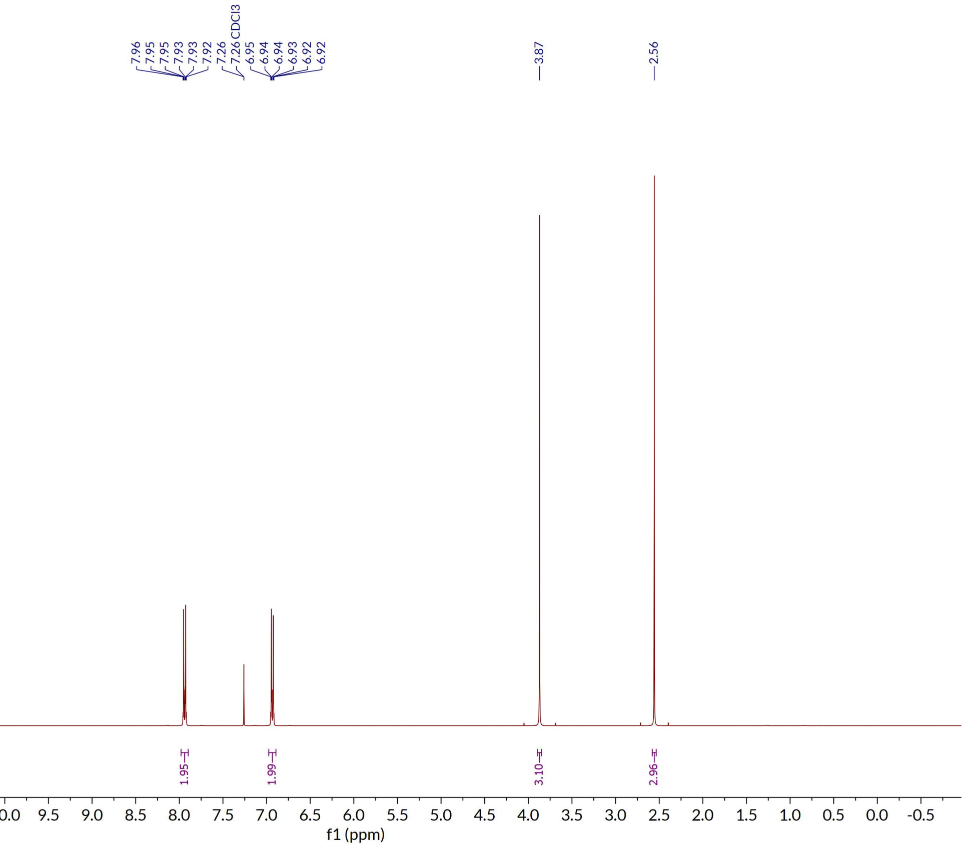

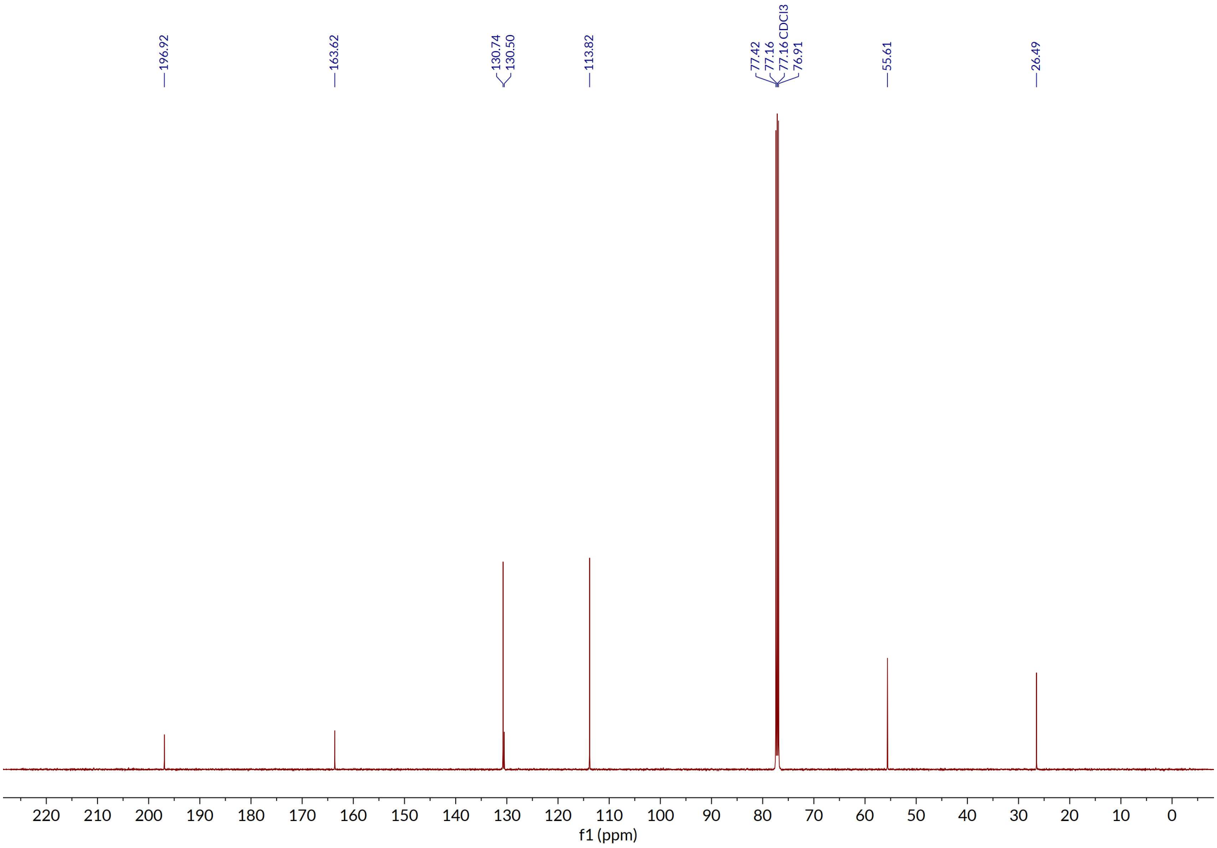

(*S*)-1-(4-(Trifluoromethyl)phenyl)ethan-1-ol (13a)

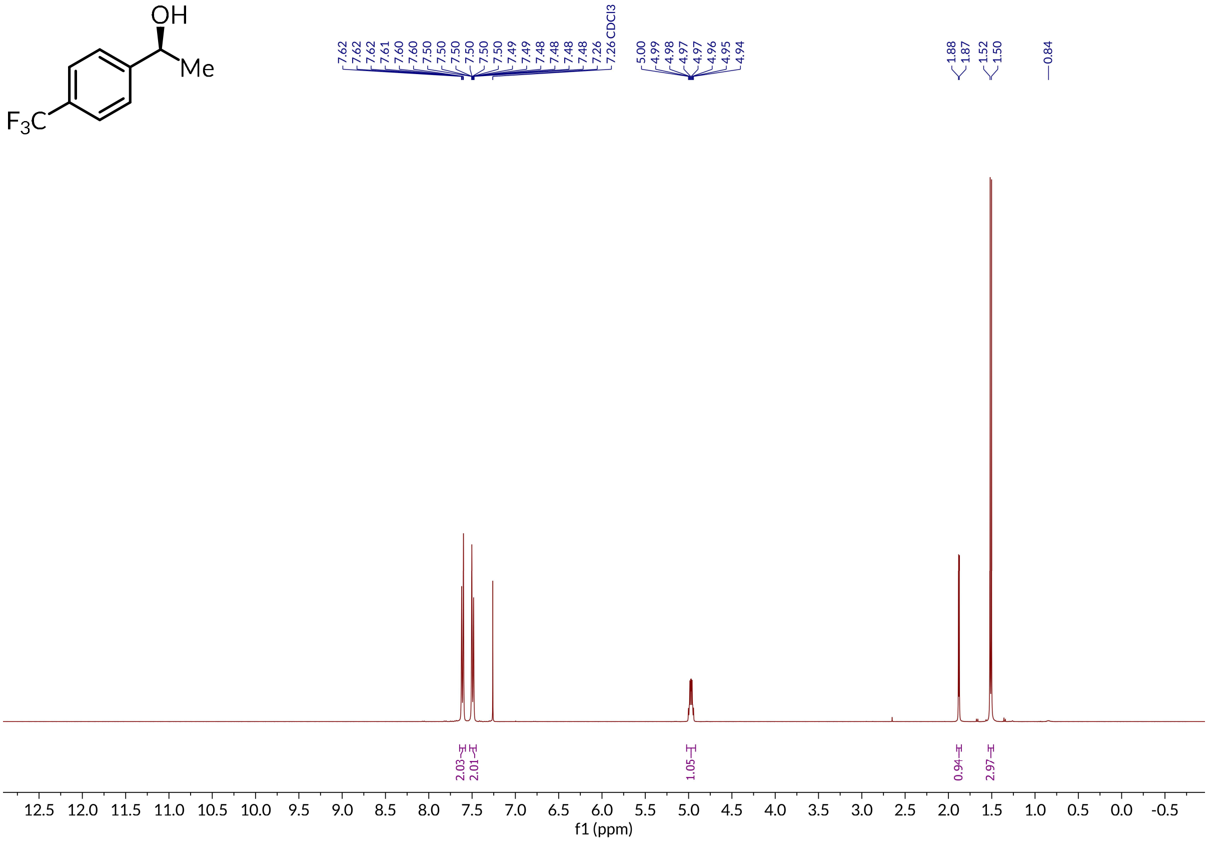

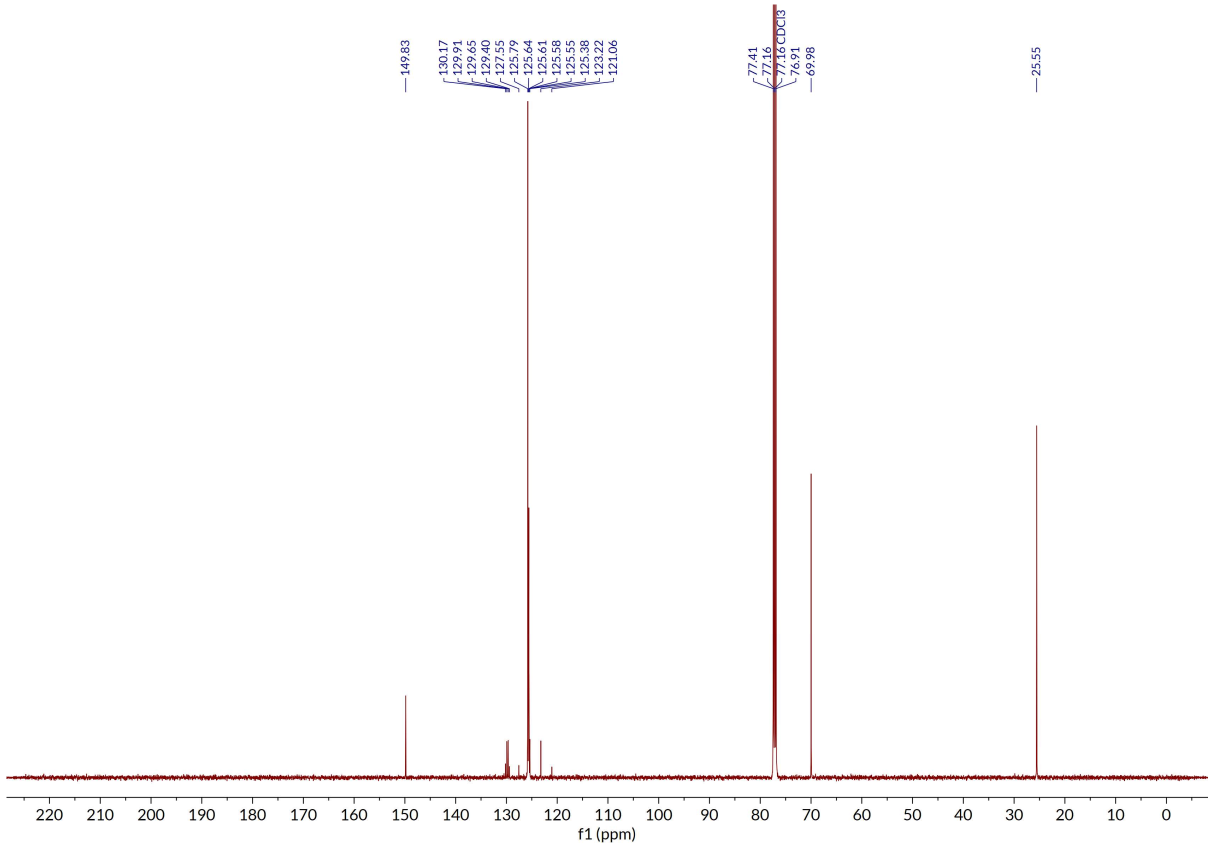
149.83
130.17
129.91
129.65
129.40
127.55
125.79
125.64
125.61
125.58
125.55
125.38
123.22
121.06
77.41
77.16
77.16 CDCl3
76.91
69.98
25.55
220 210 200 190 180 170 160 150 140 130 120 110 100 90 80 70 60 50 40 30 20 10 0
f1 (ppm)

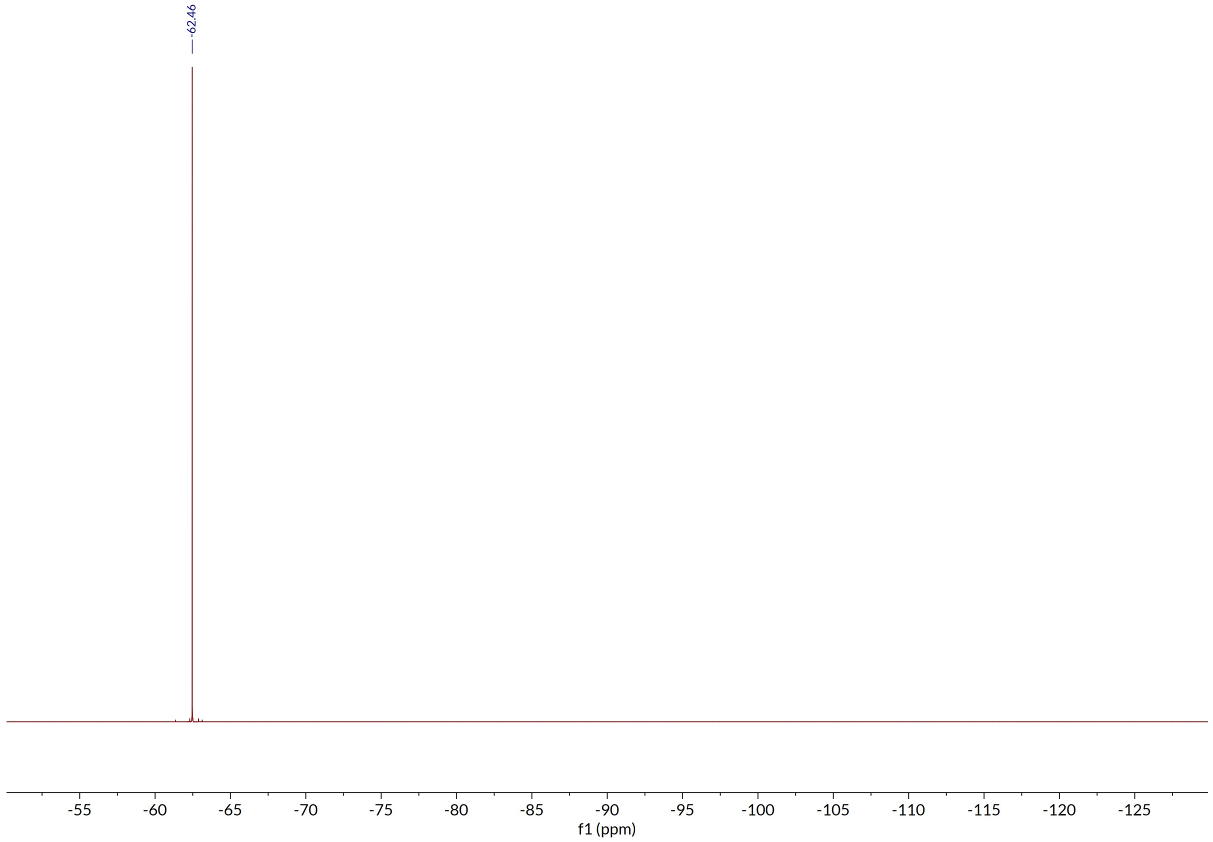
-62.46
-55 -60 -65 -70 -75 -80 -85 -90 -95 -100 -105 -110 -115 -120 -125
f1 (ppm)

1-(4-(Trifluoromethyl)phenyl)ethan-1-one (13b)

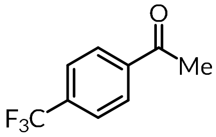

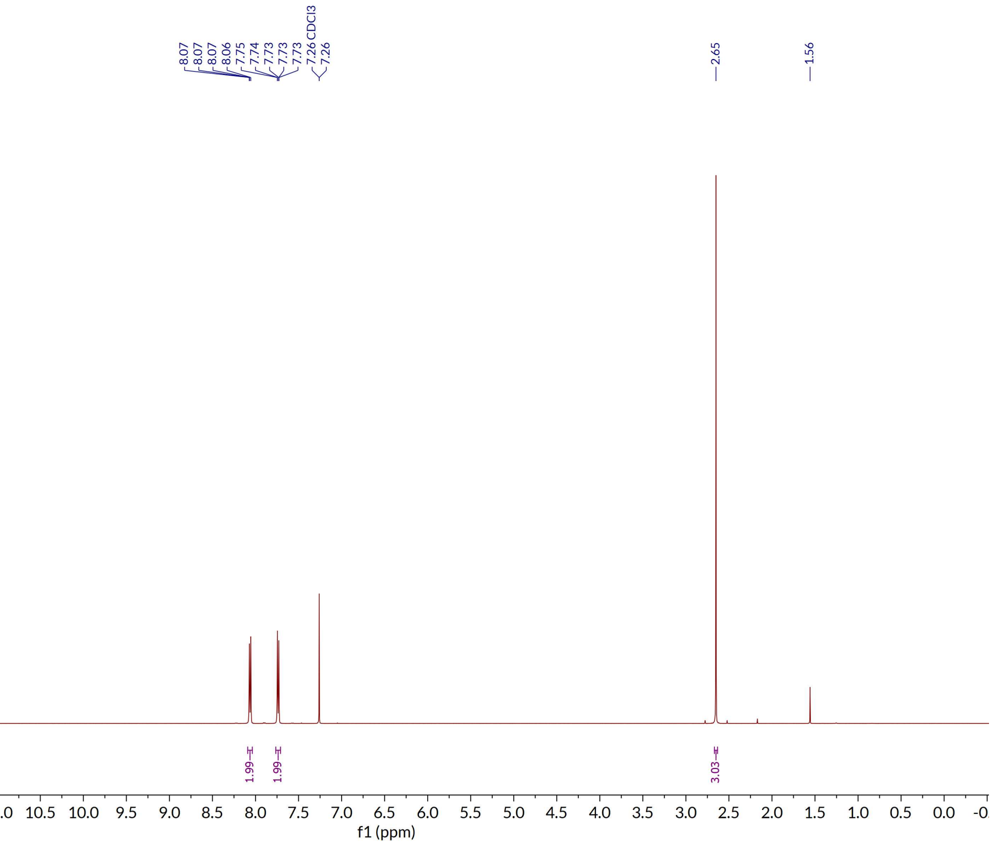

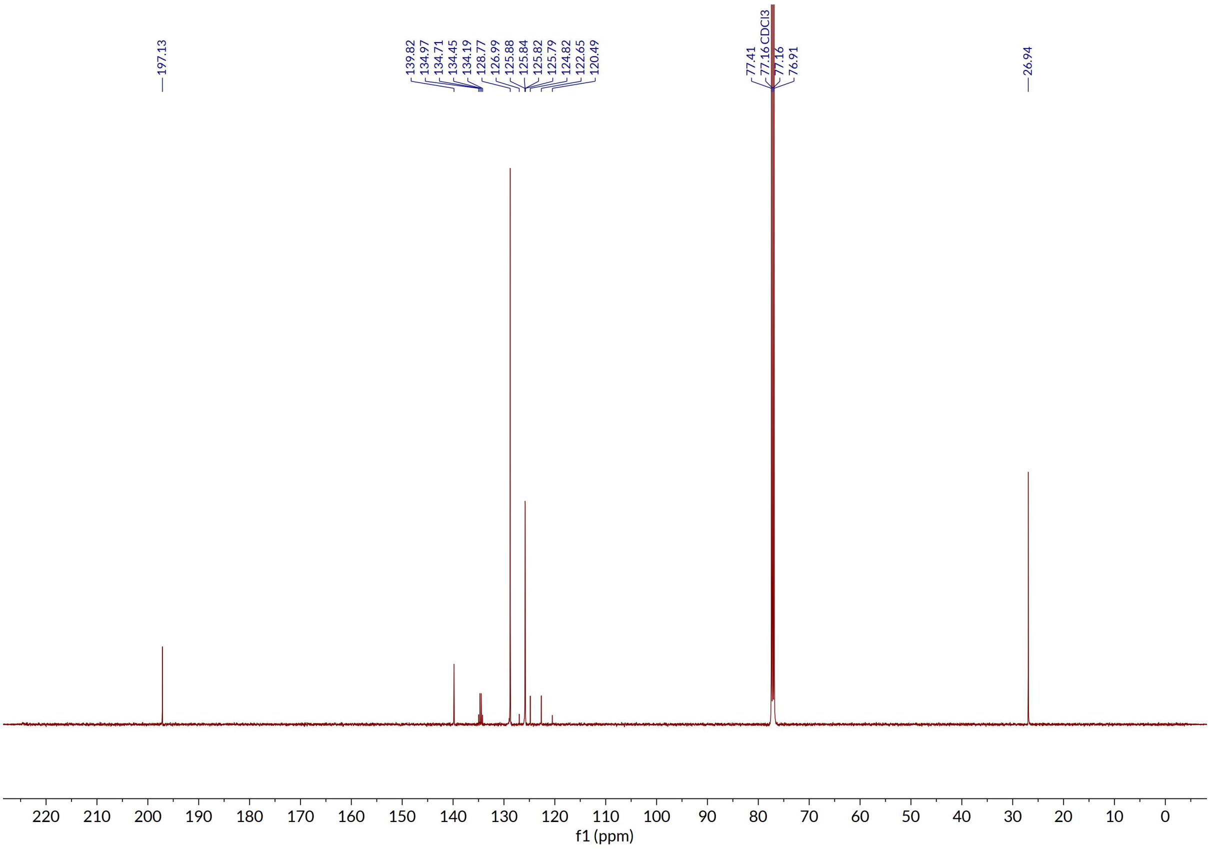

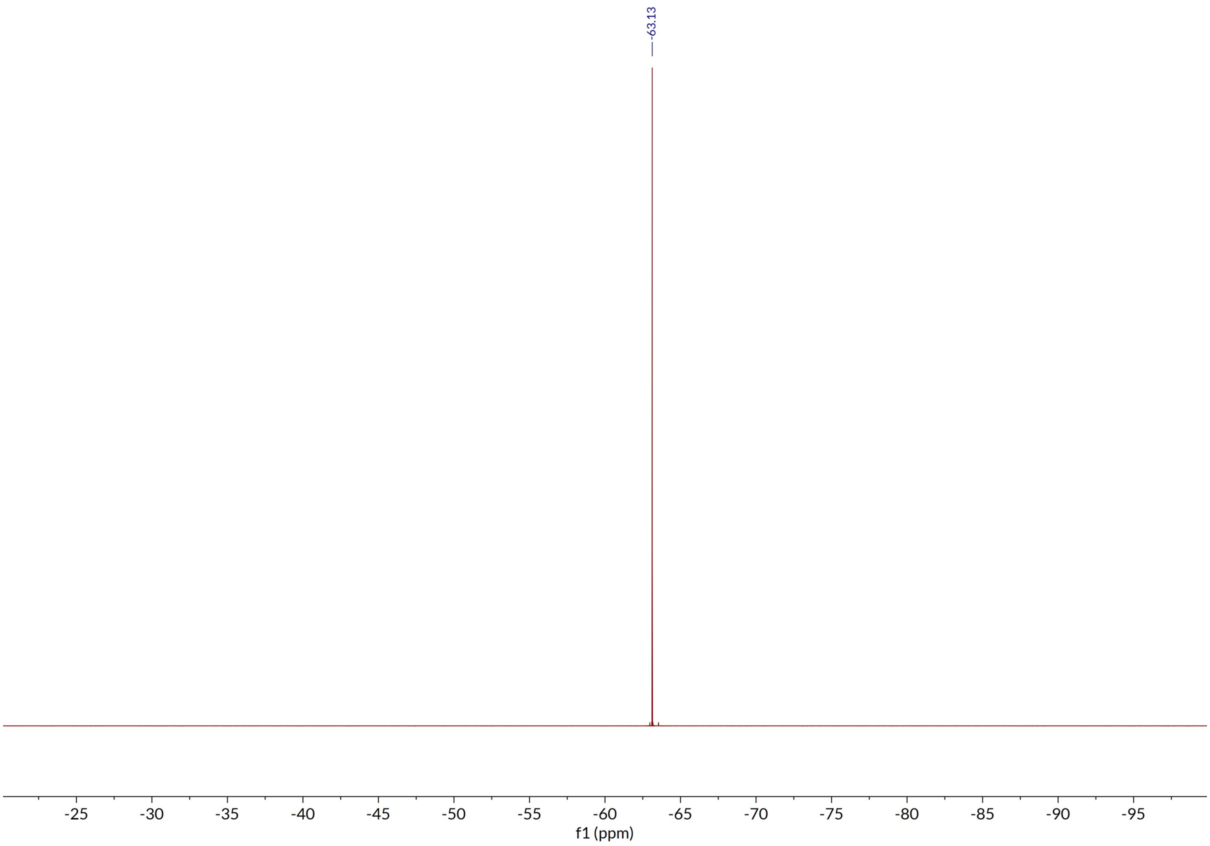

(*S*)-1-(4-Aminophenyl)ethan-1-ol (14a)

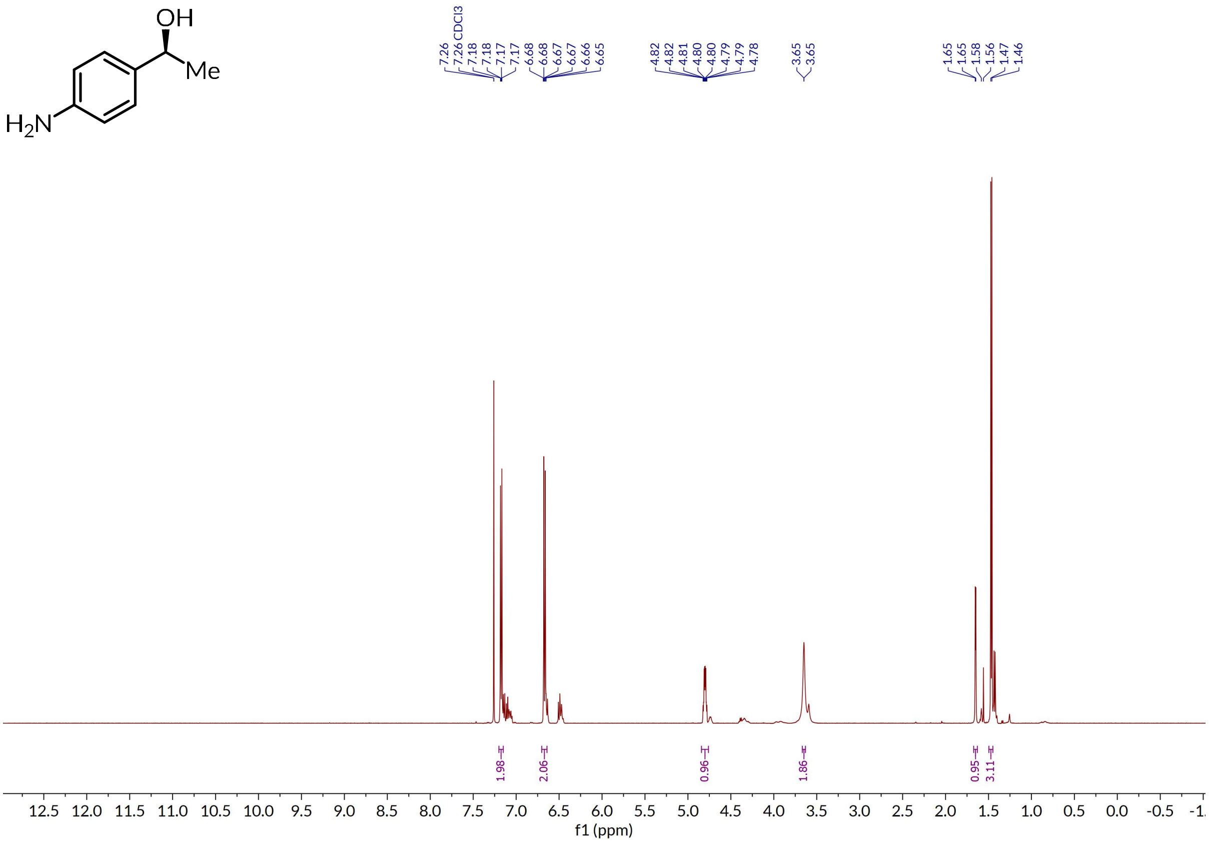

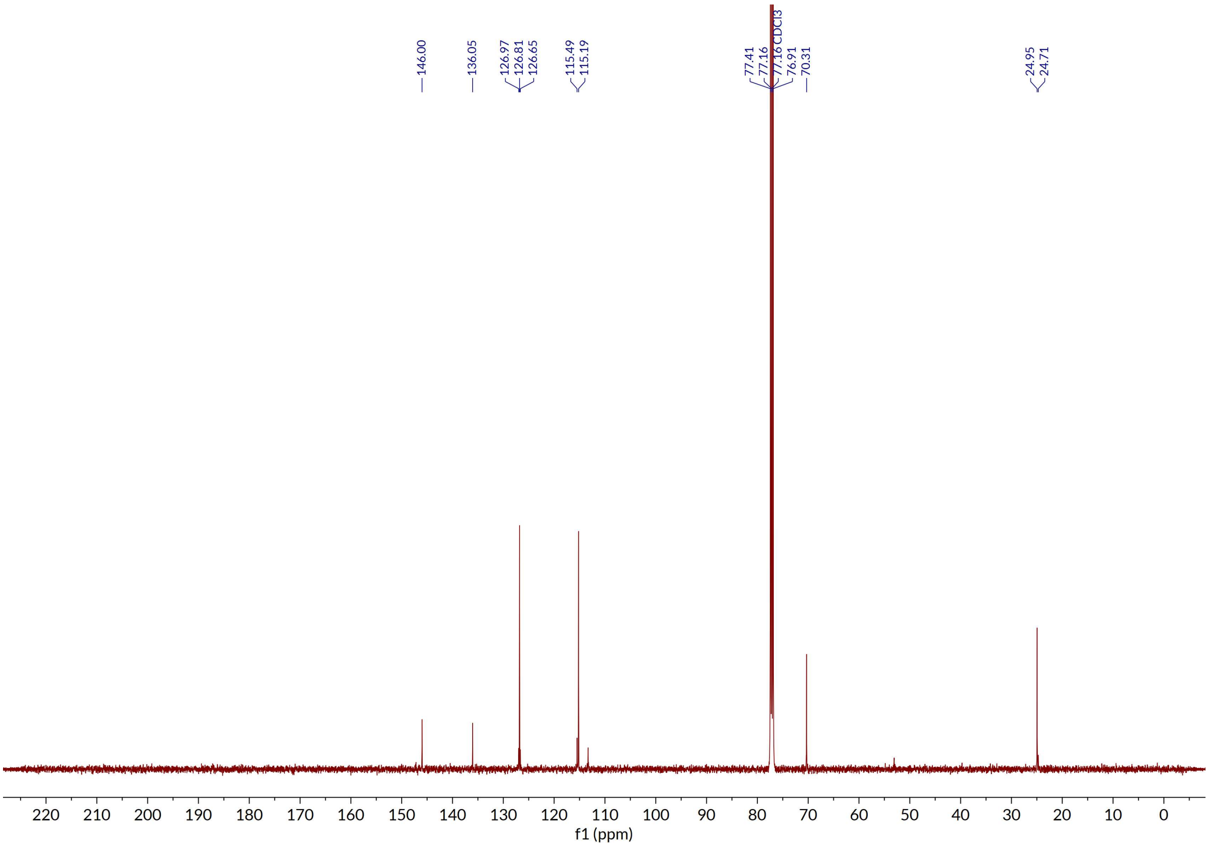

1-(4-Aminophenyl)ethan-1-one (14b)

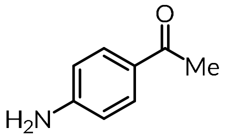

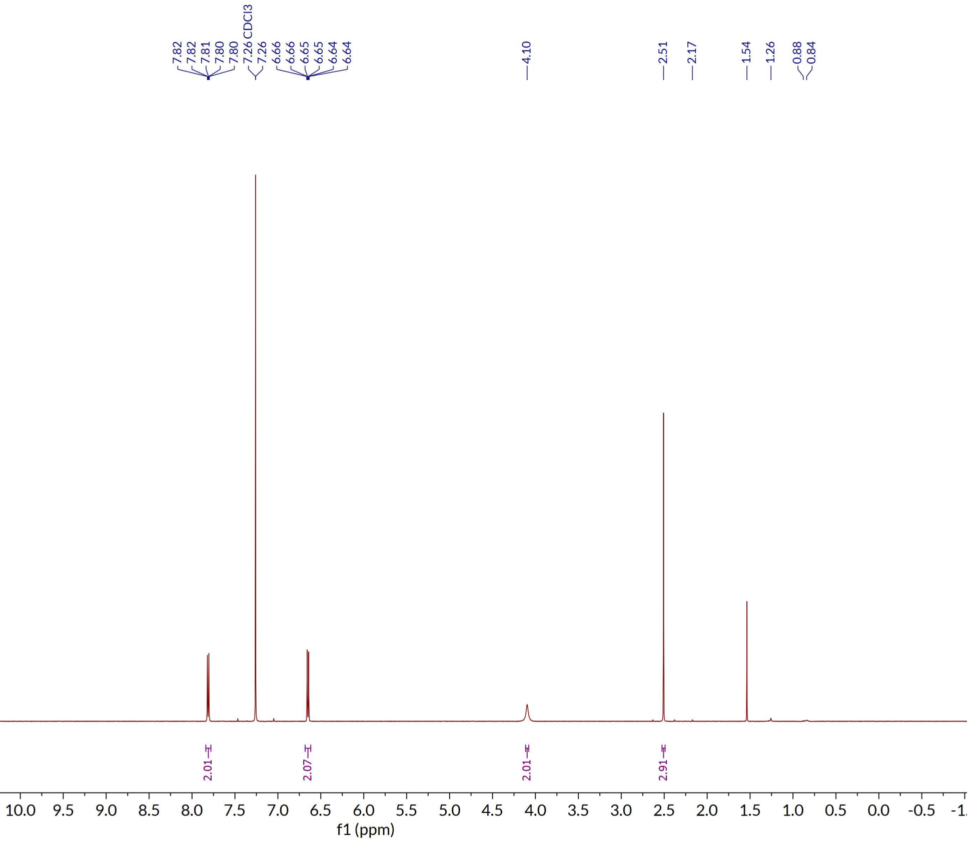

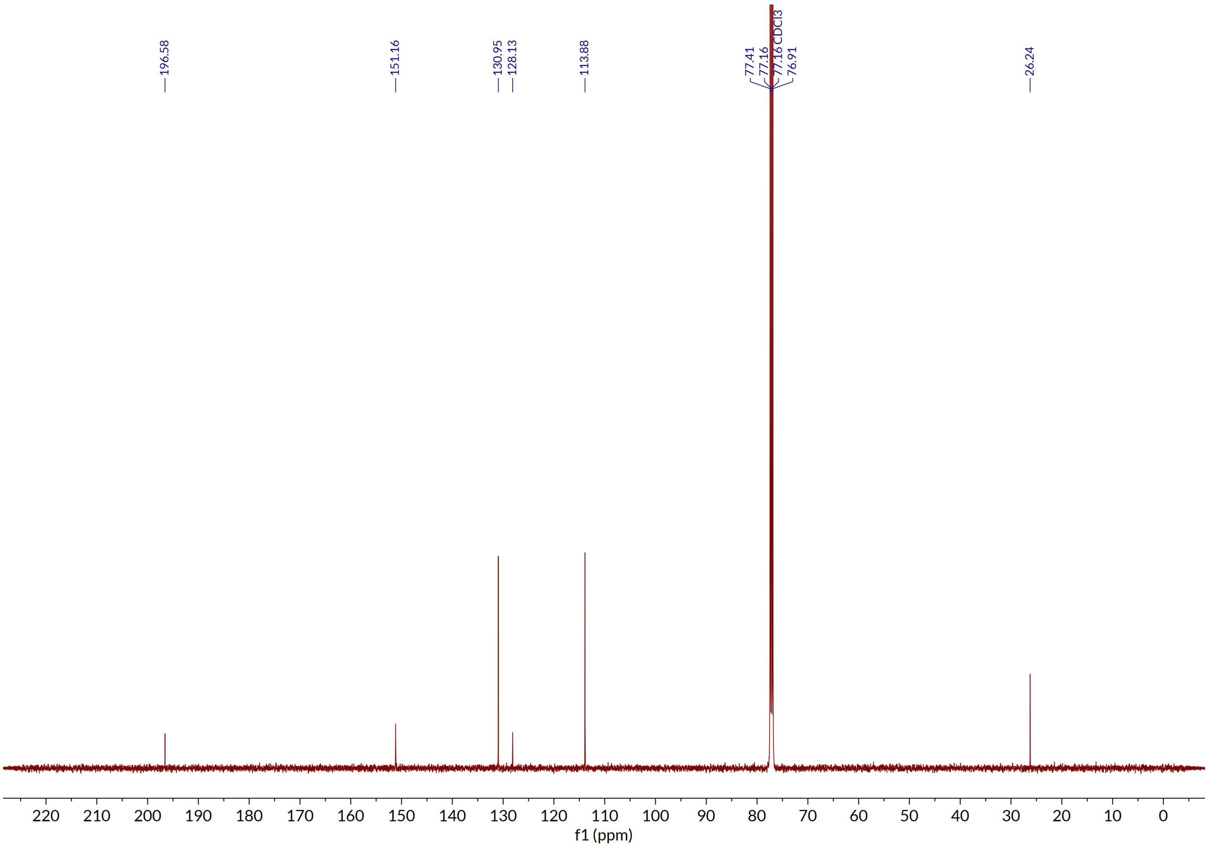

(*S*)-1-(*p*-Tolyl)ethan-1-ol (15a)

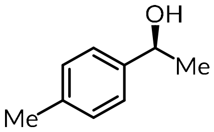

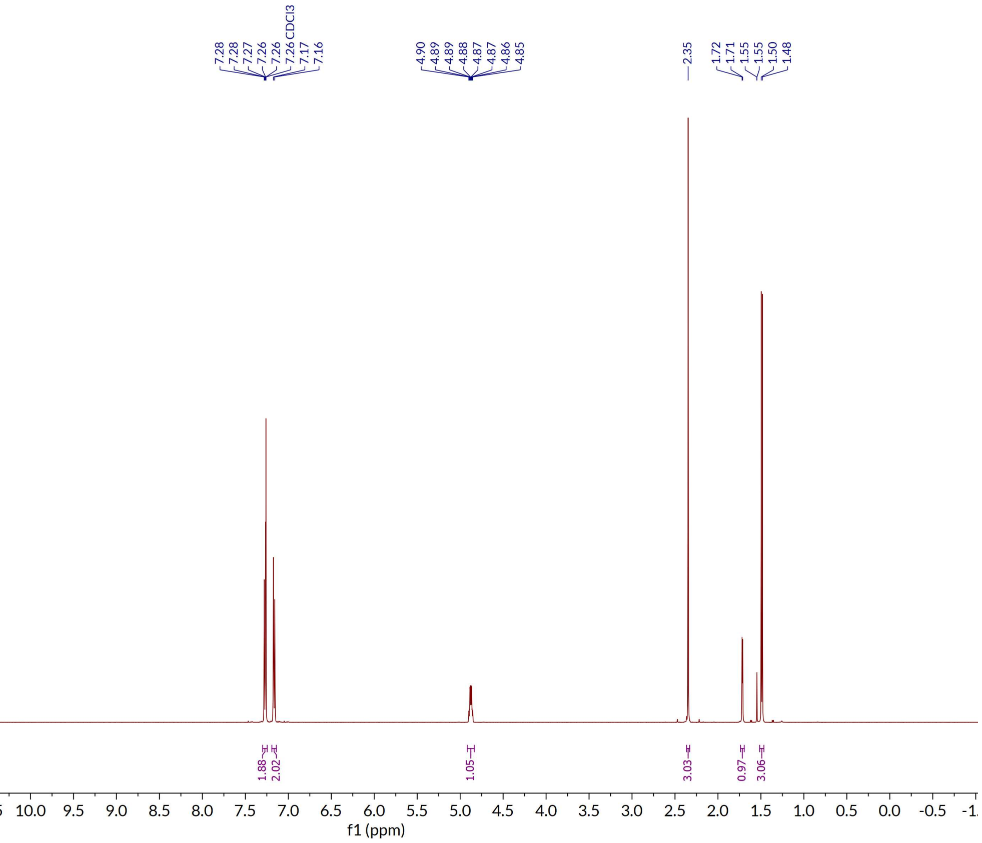

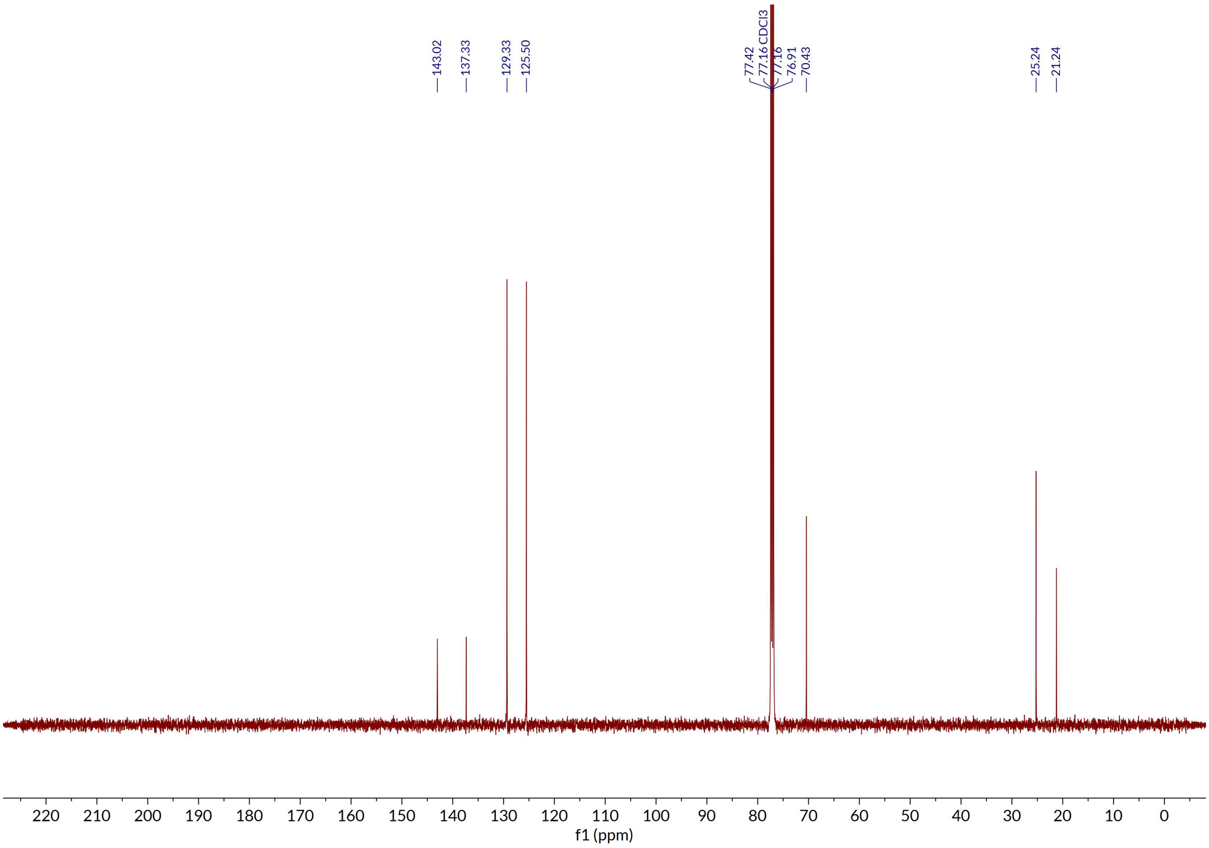

1-(*p*-Tolyl)ethan-1-one (15b)

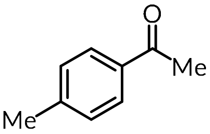

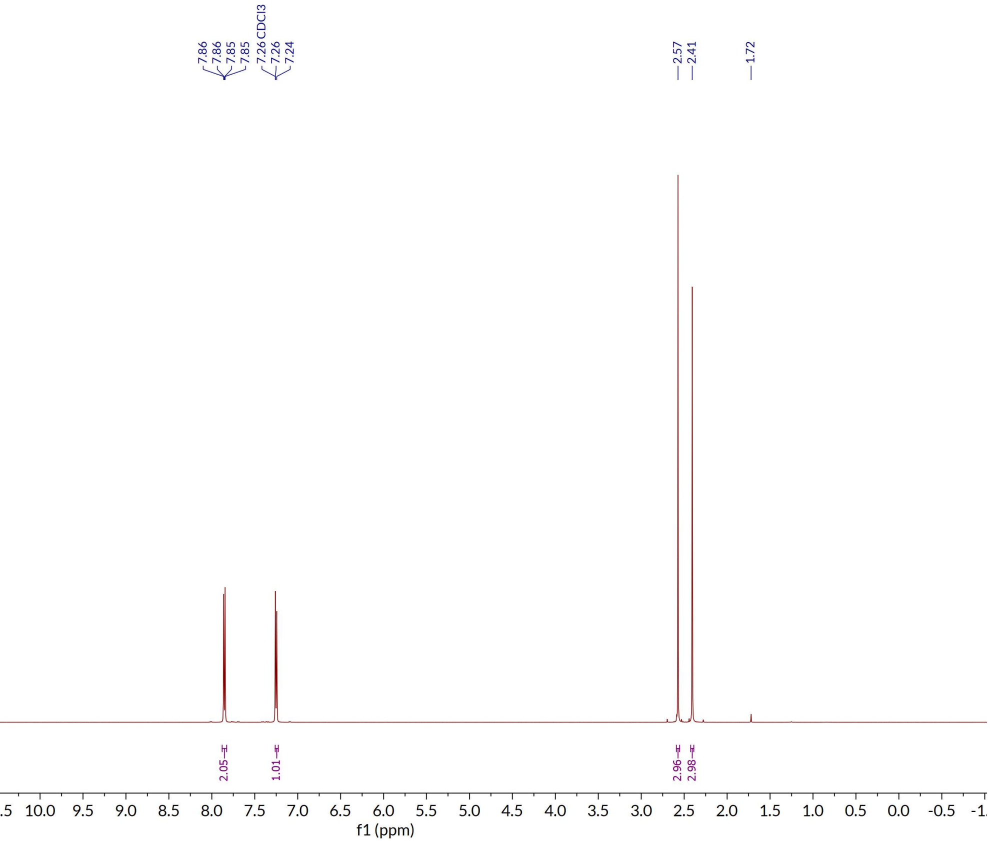

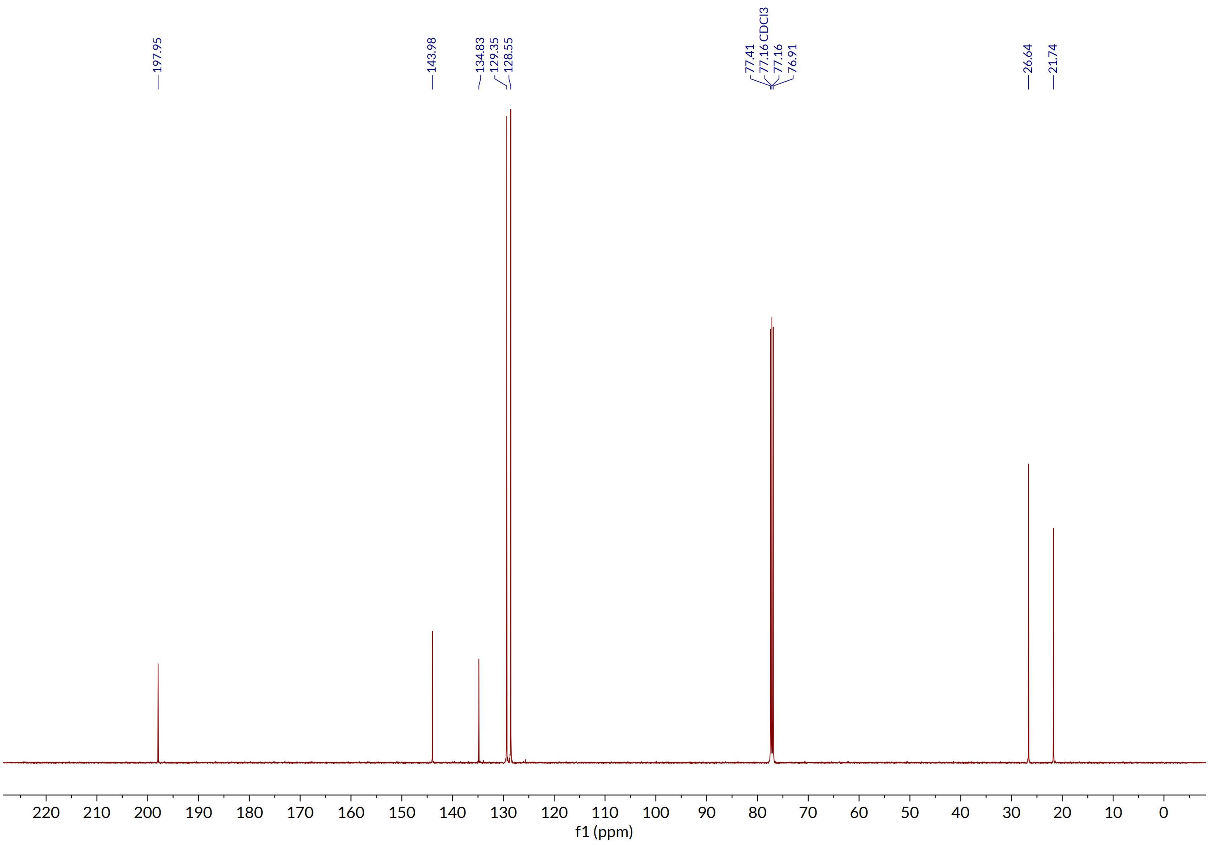

(*S*)-1-(4-(*tert*-Butyl)phenyl)ethan-1-ol (16a)

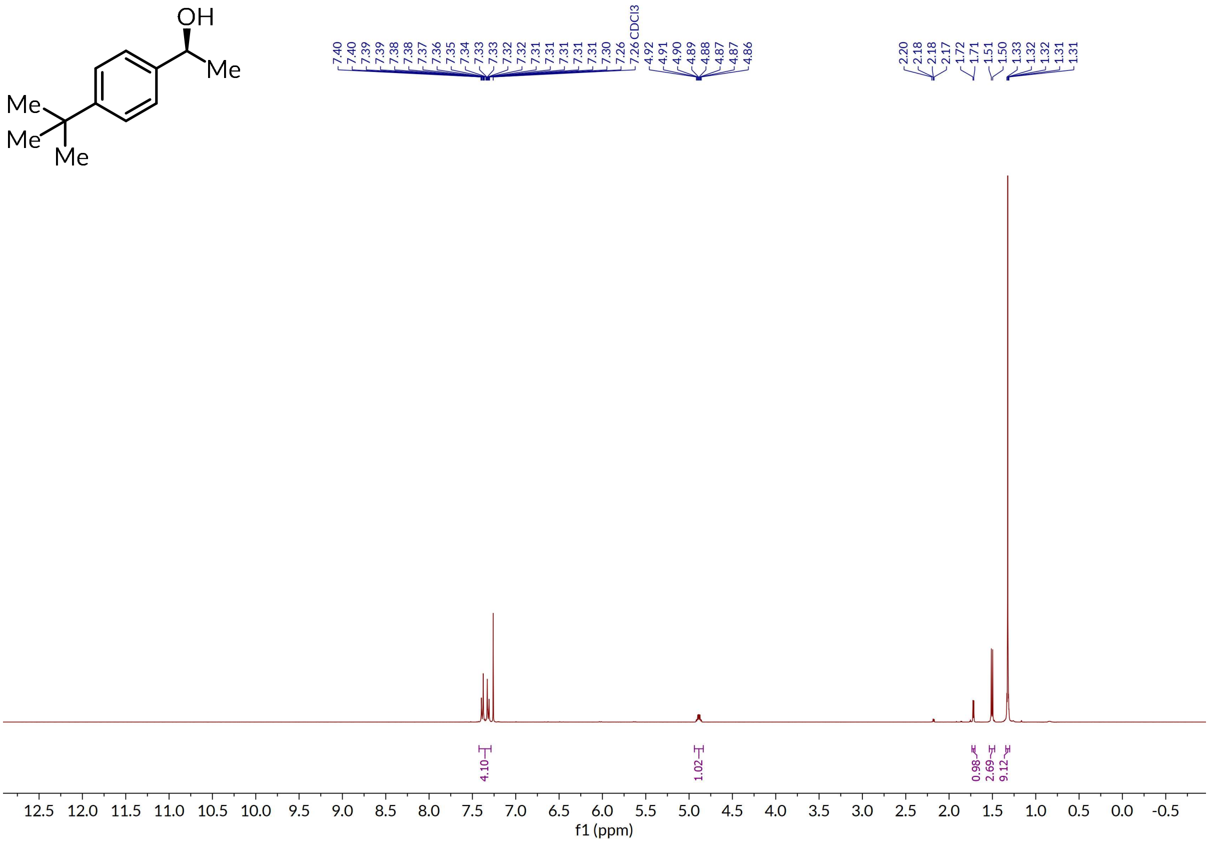

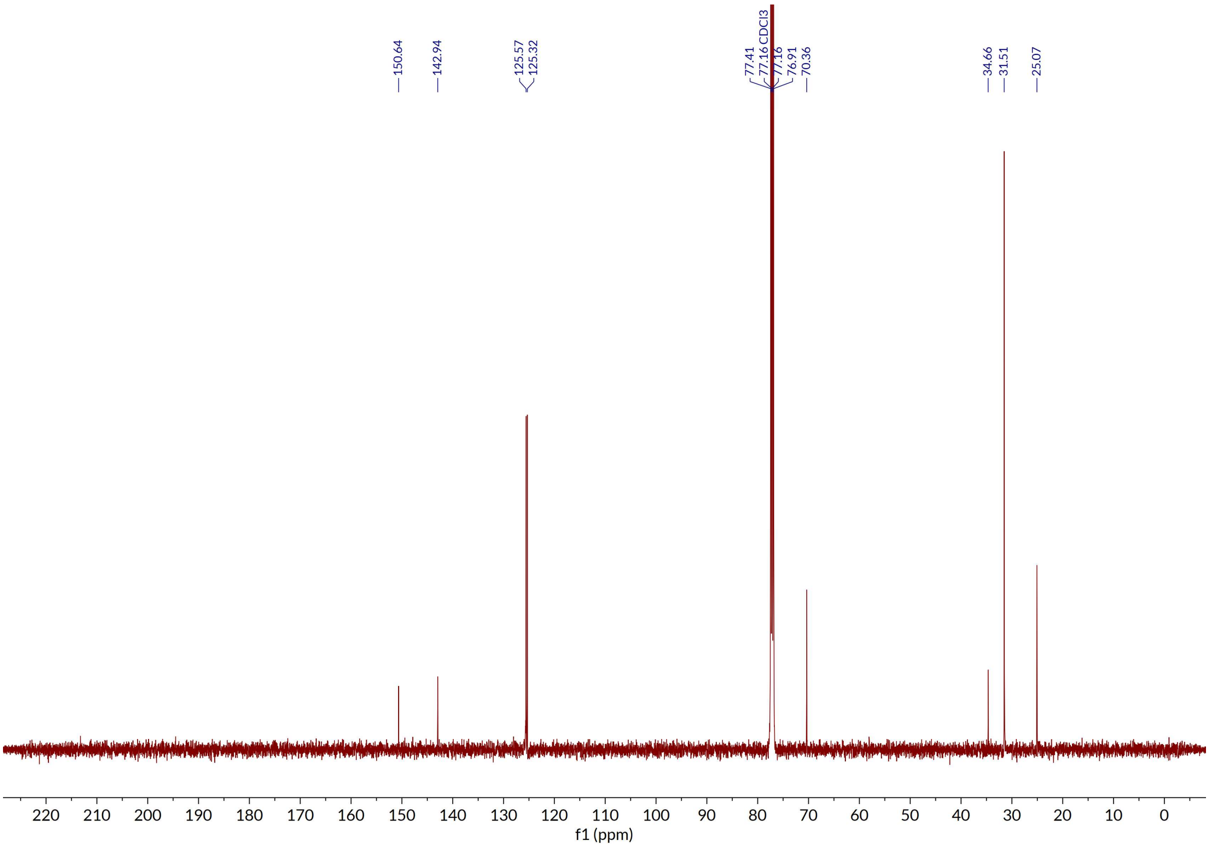

1-(4-(*tert*-Butyl)phenyl)ethan-1-one (16b)

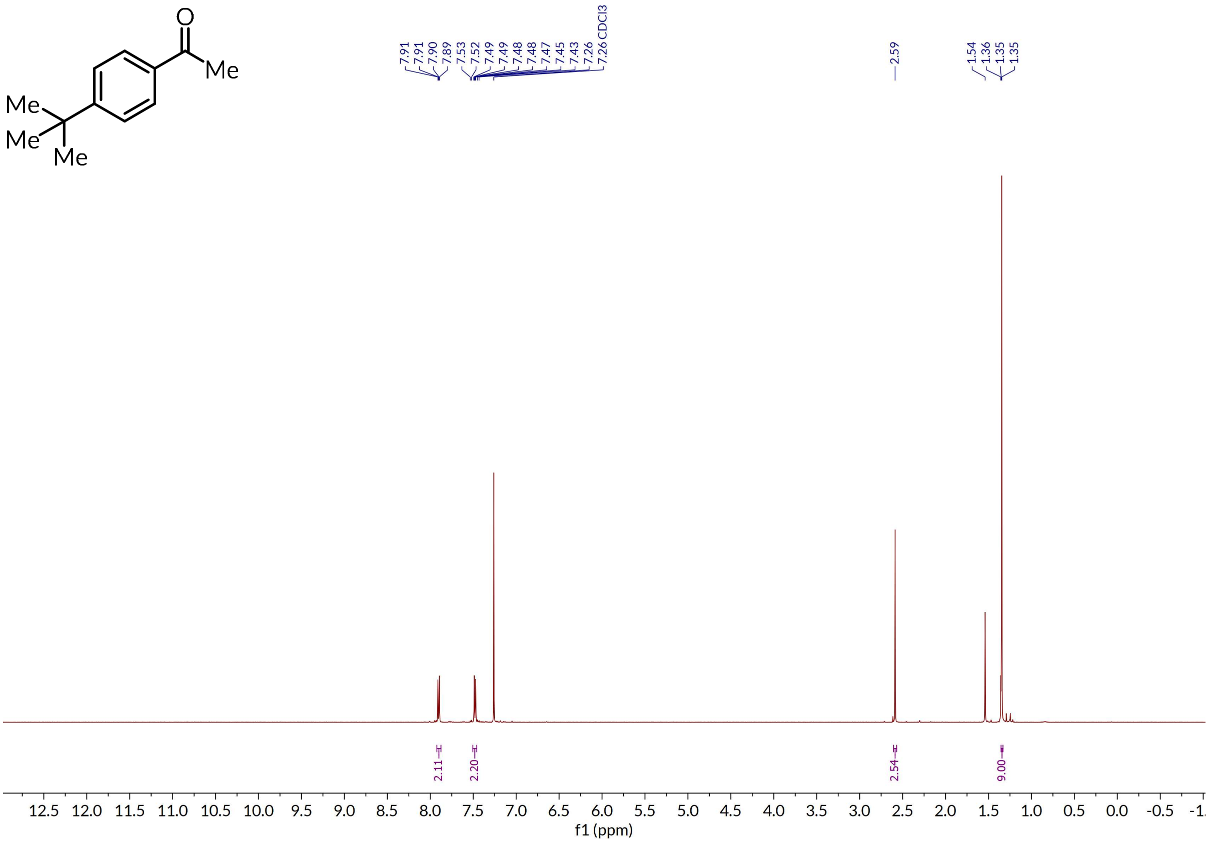

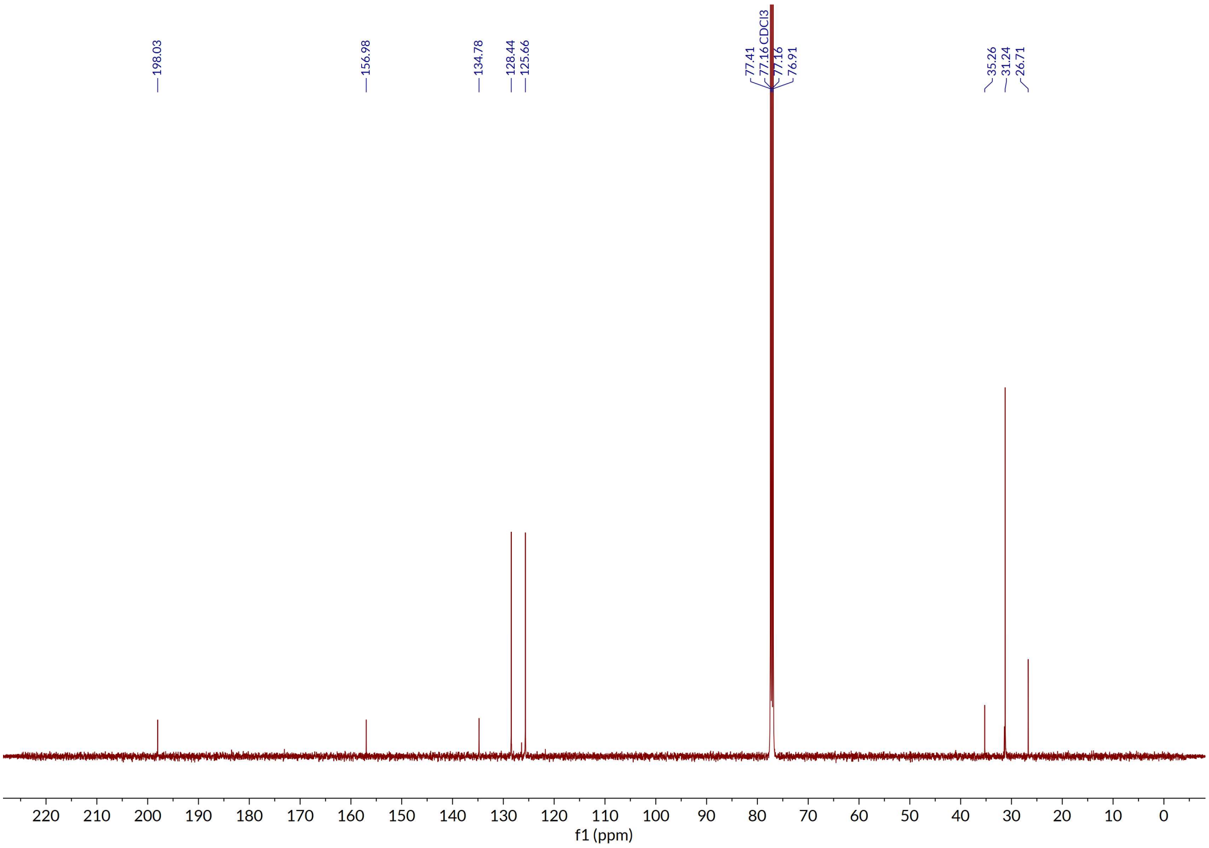

(*S*)-4-(1-Hydroxyethyl)benzonitrile (17a)

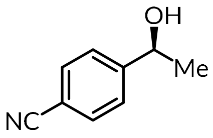

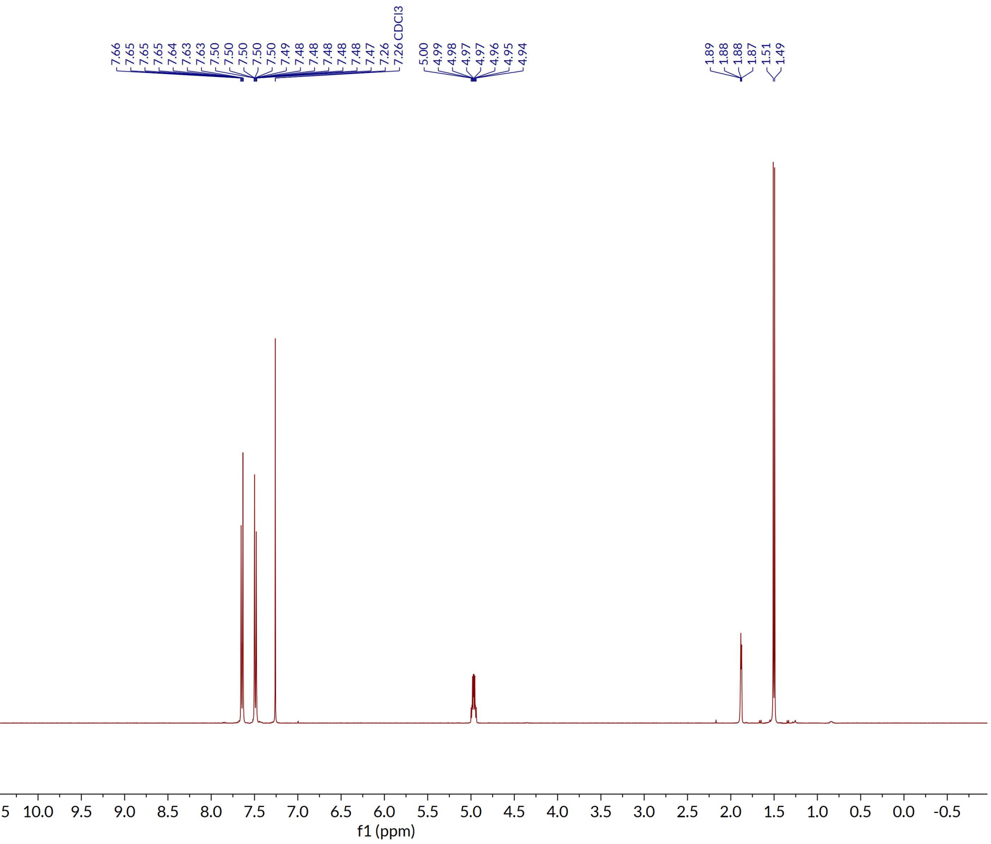

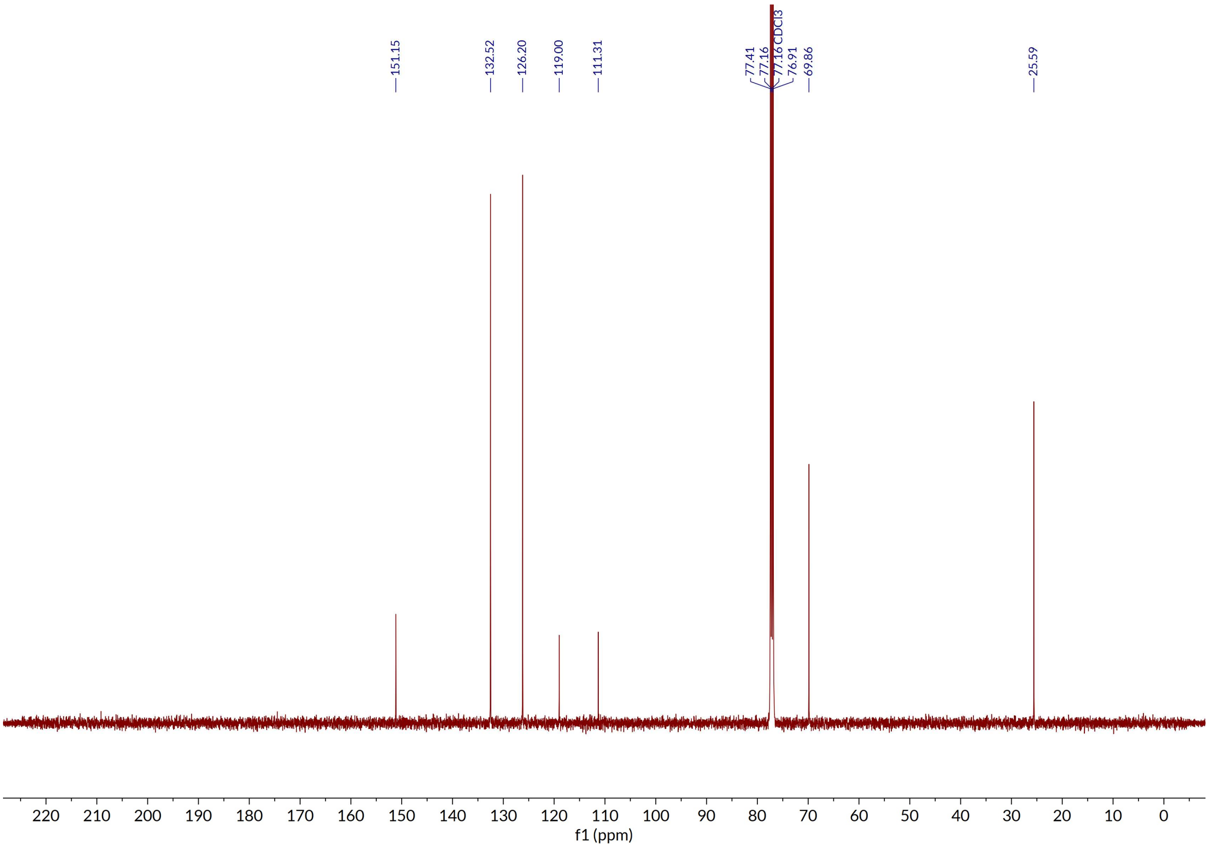

4-Acetylbenzonitrile (17b)

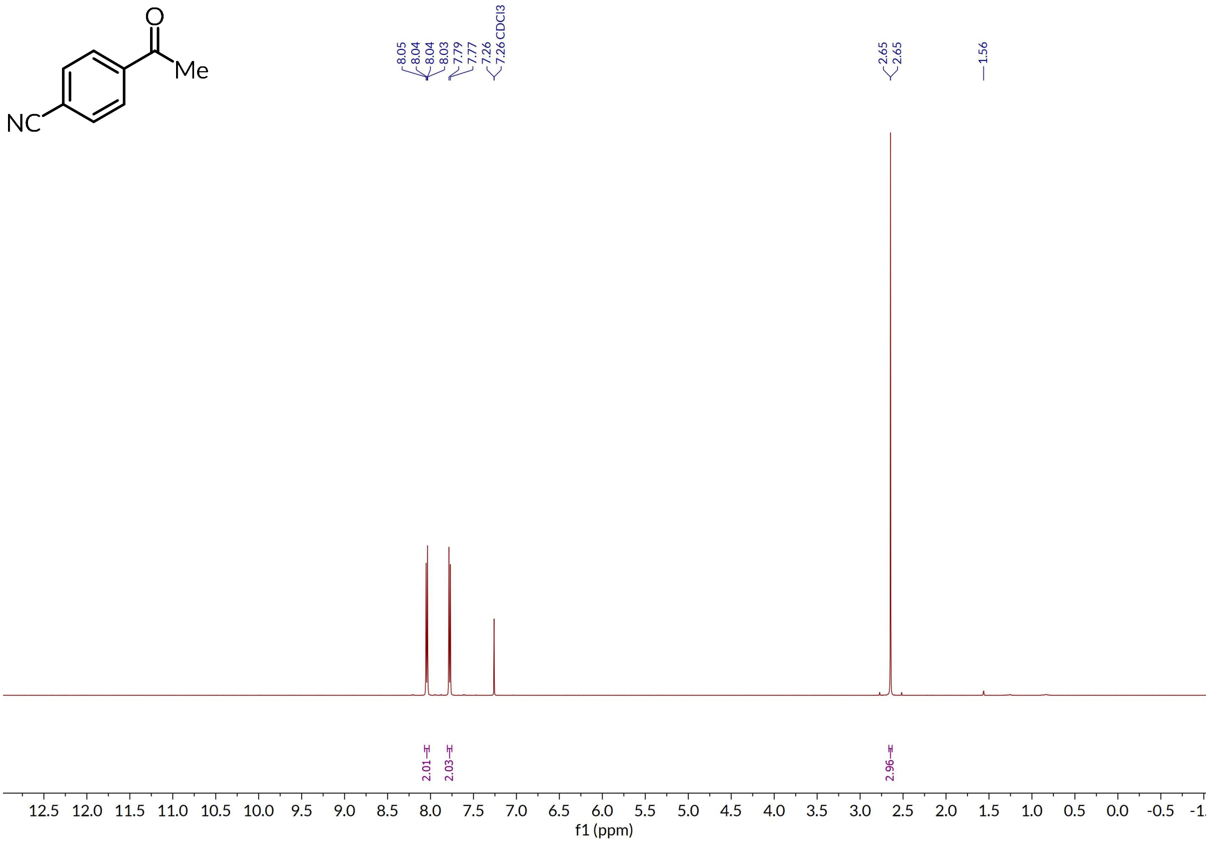

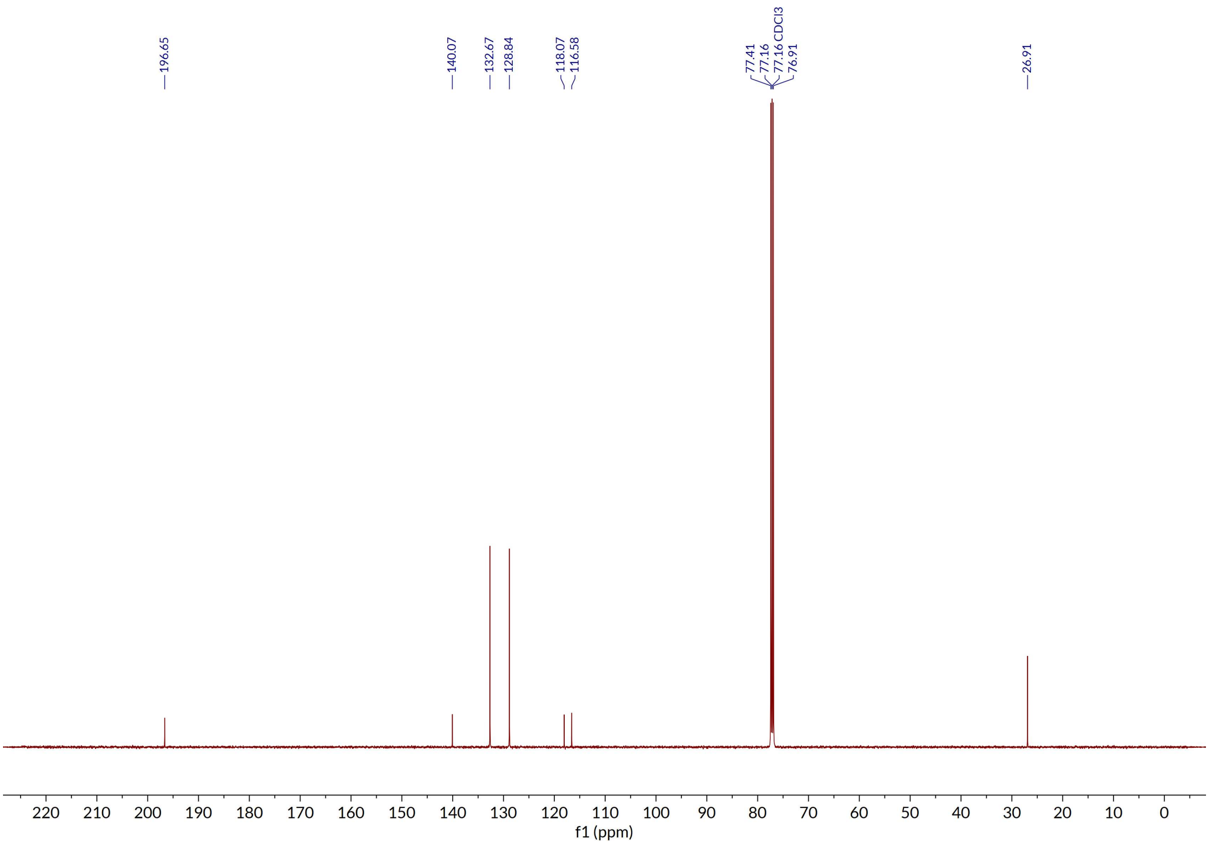

(*S*)-4-(1-Hydroxyethyl)phenol (18a)

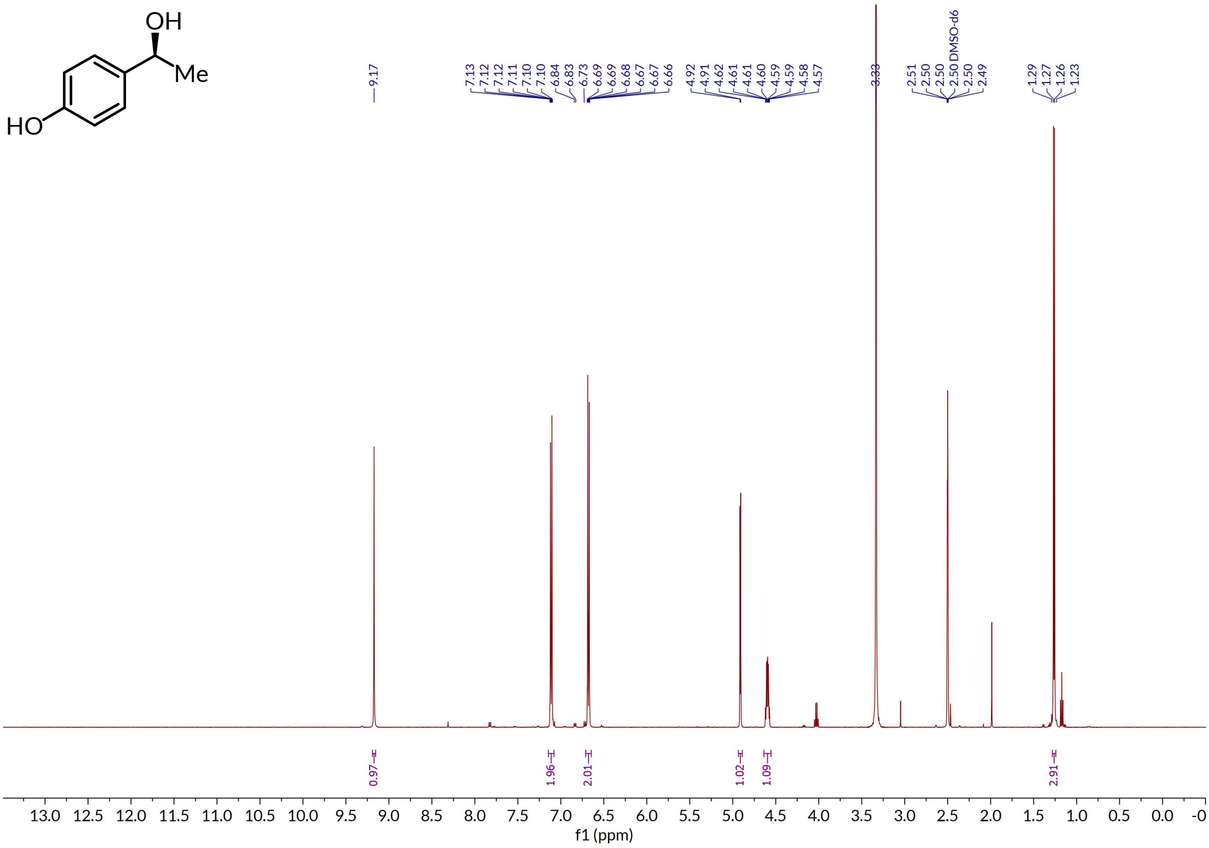

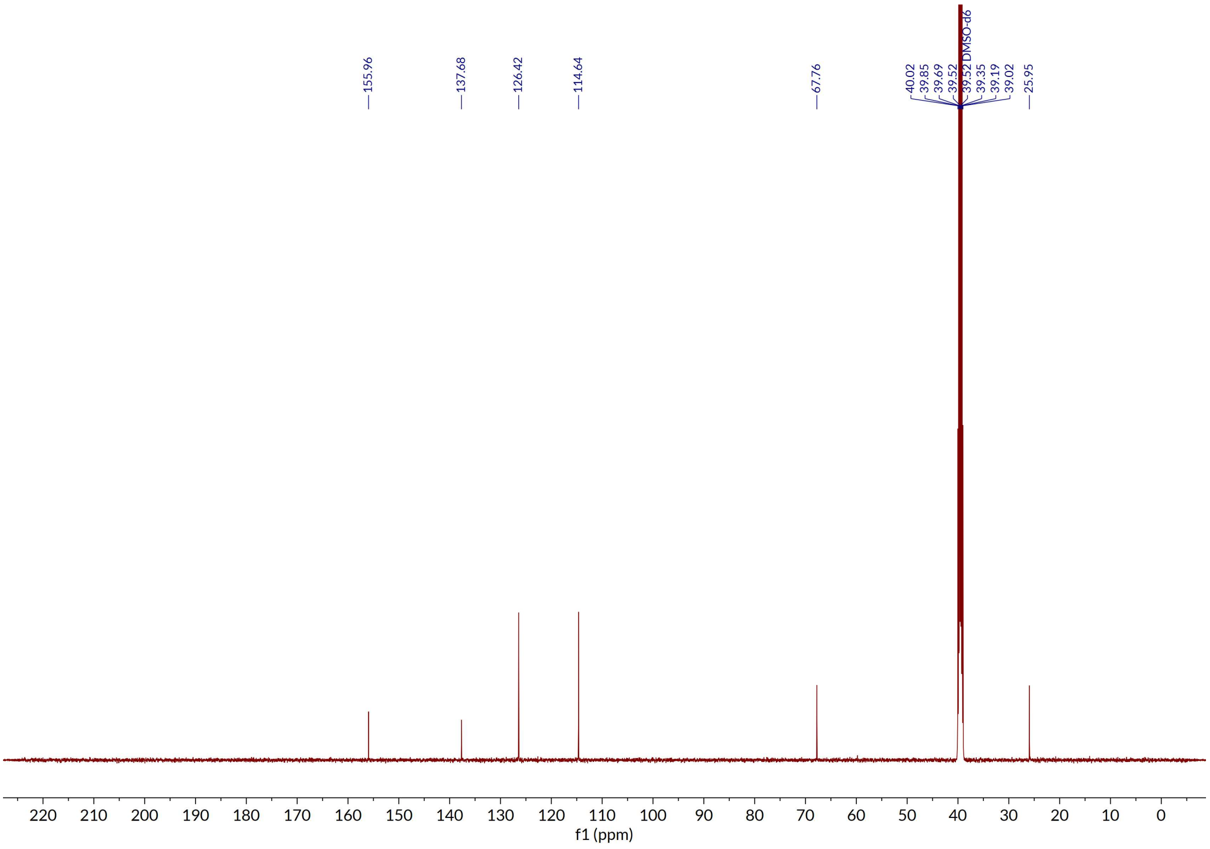

1-(4-Hydroxyphenyl)ethan-1-one (18b)

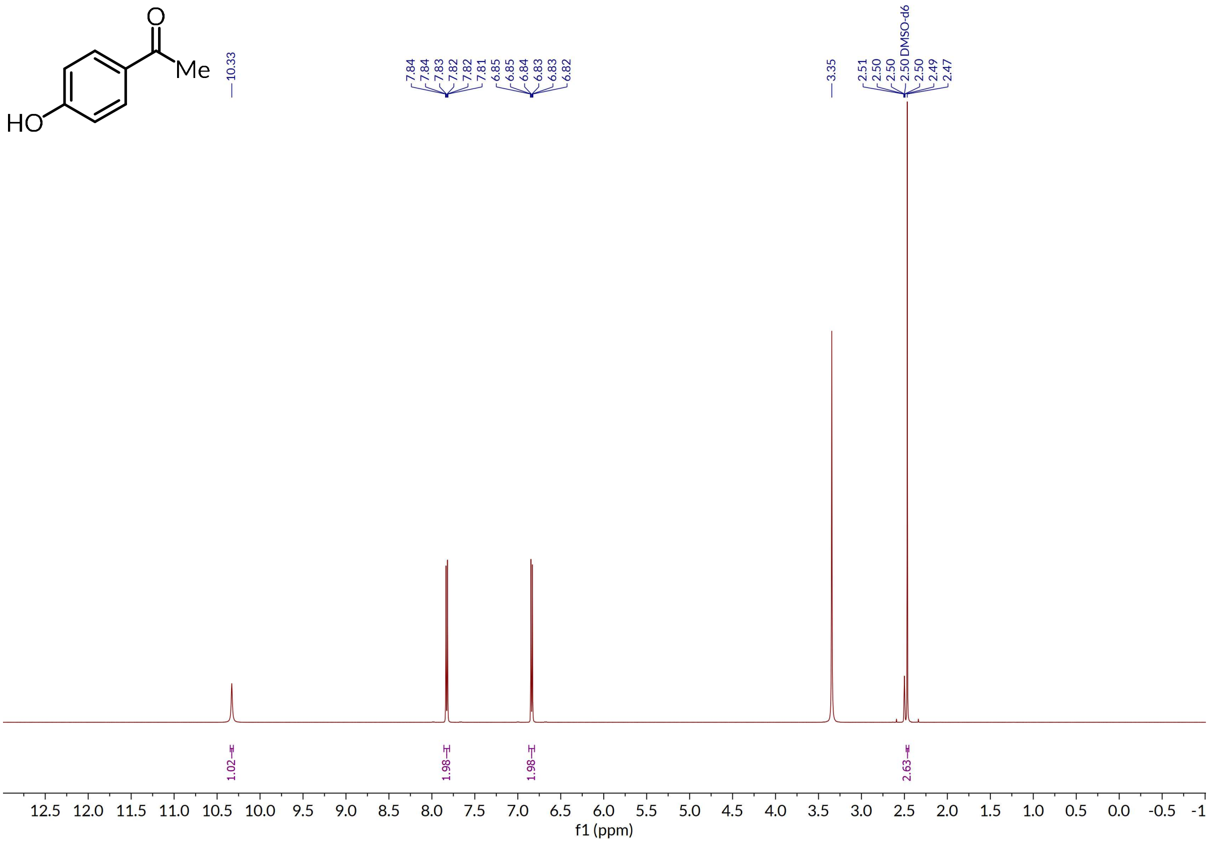

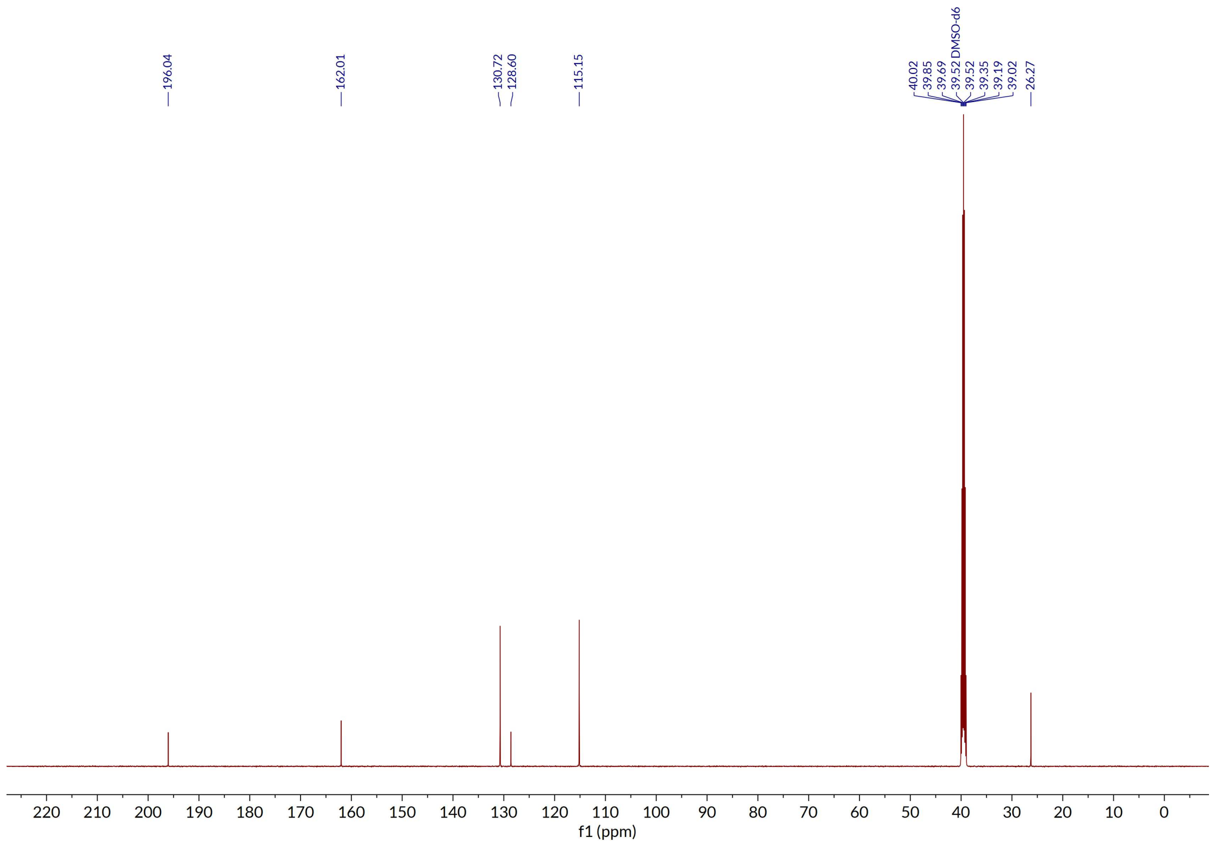

(*S*)-1-([1,1'-Biphenyl]-4-yl)ethan-1-ol (19a)

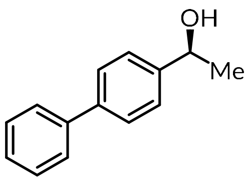

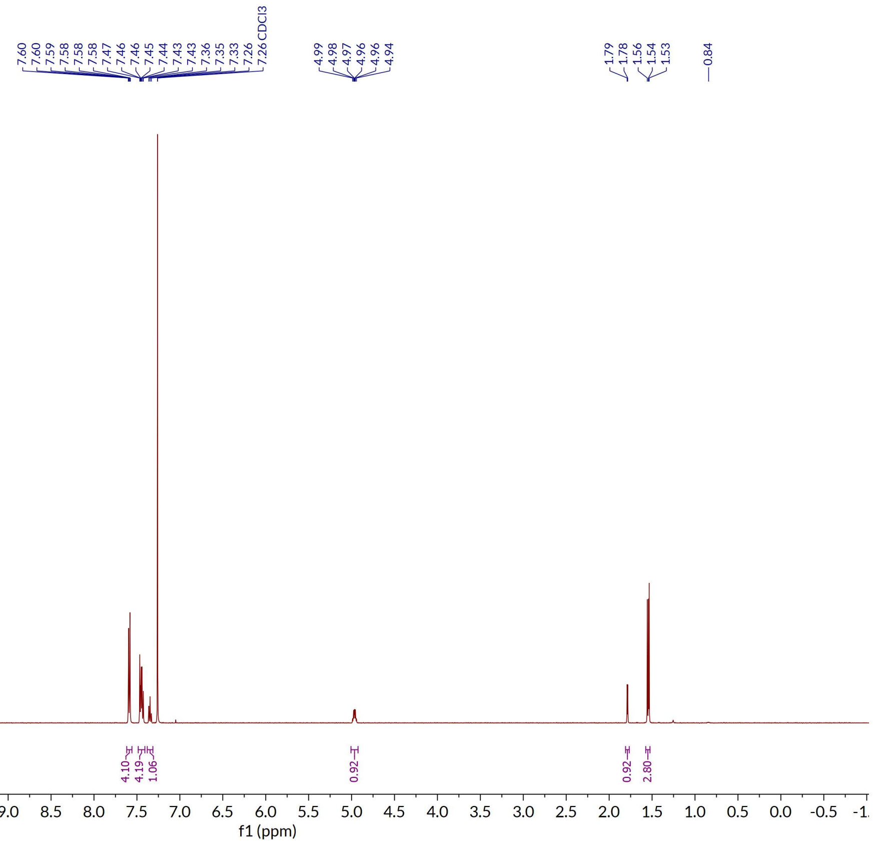

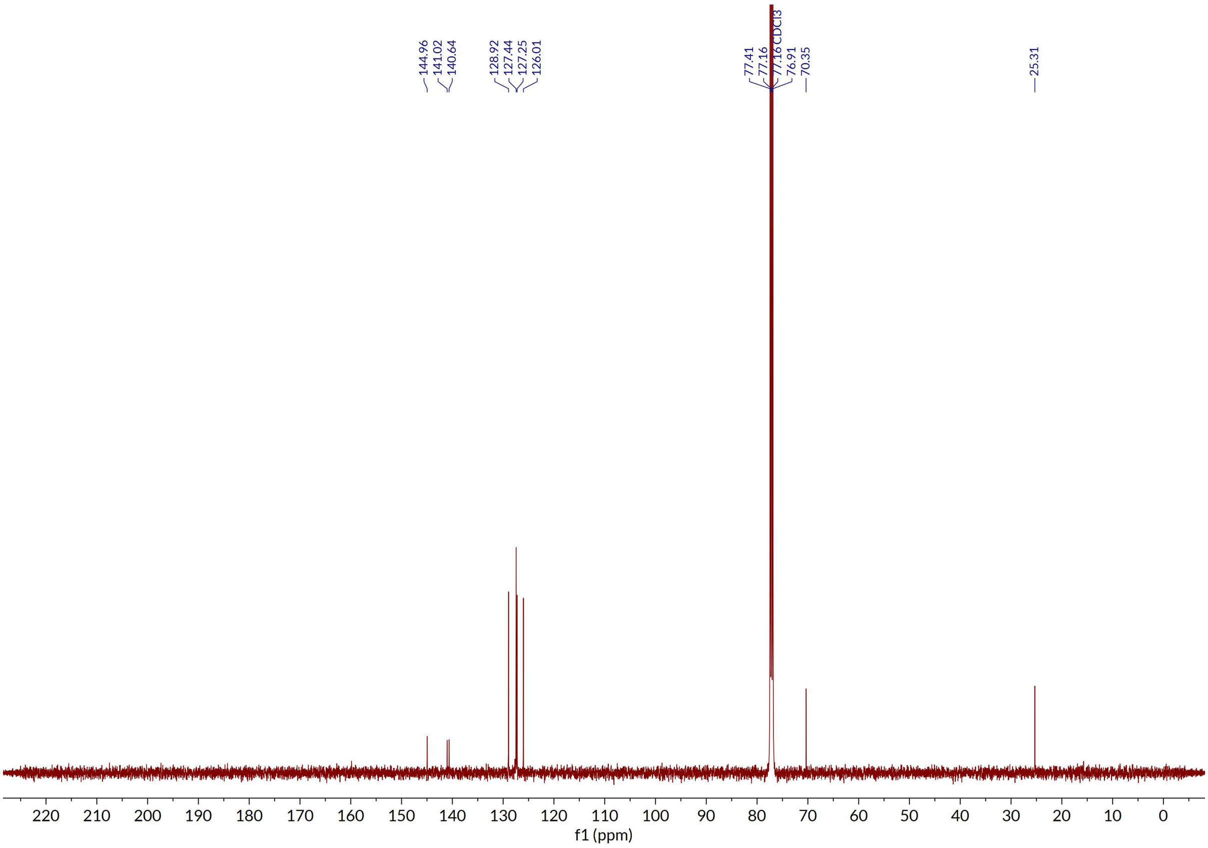

1-([1,1'-Biphenyl]-4-yl)ethan-1-one (19b)

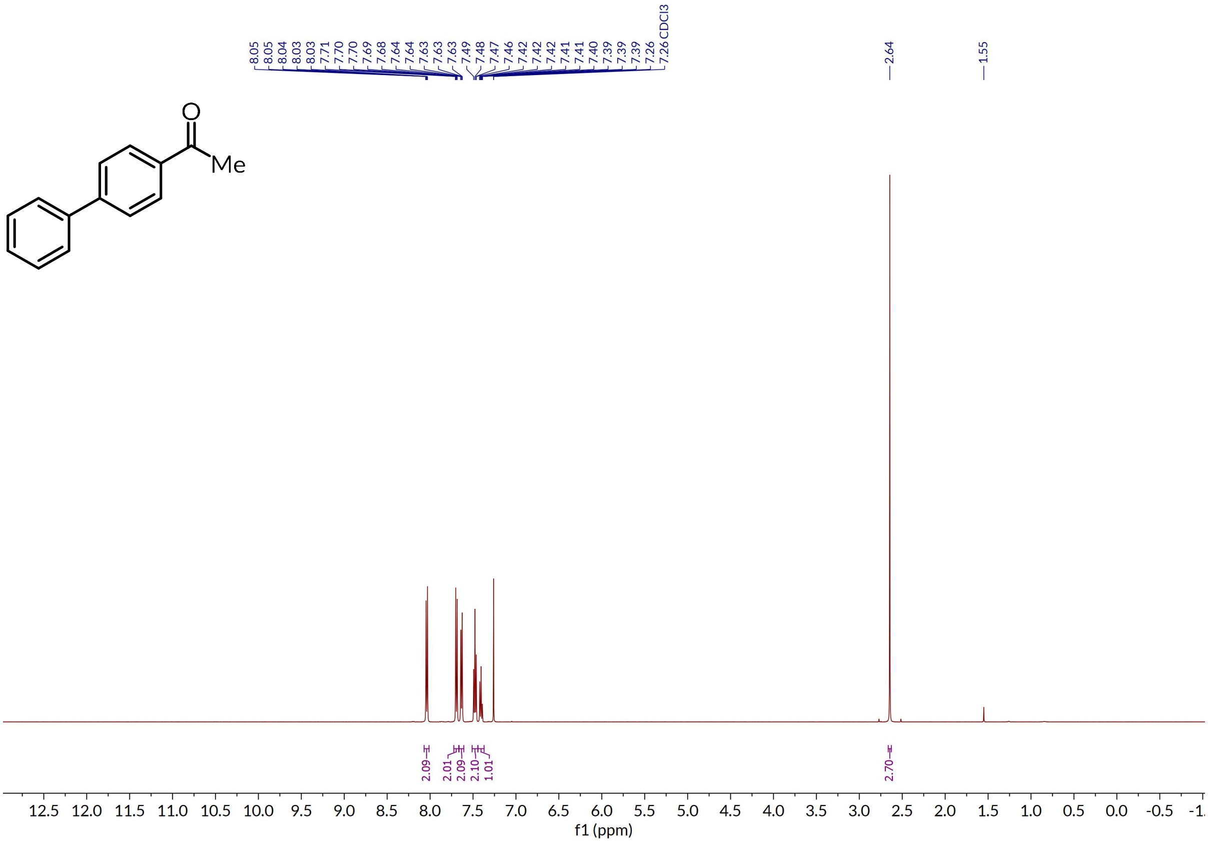

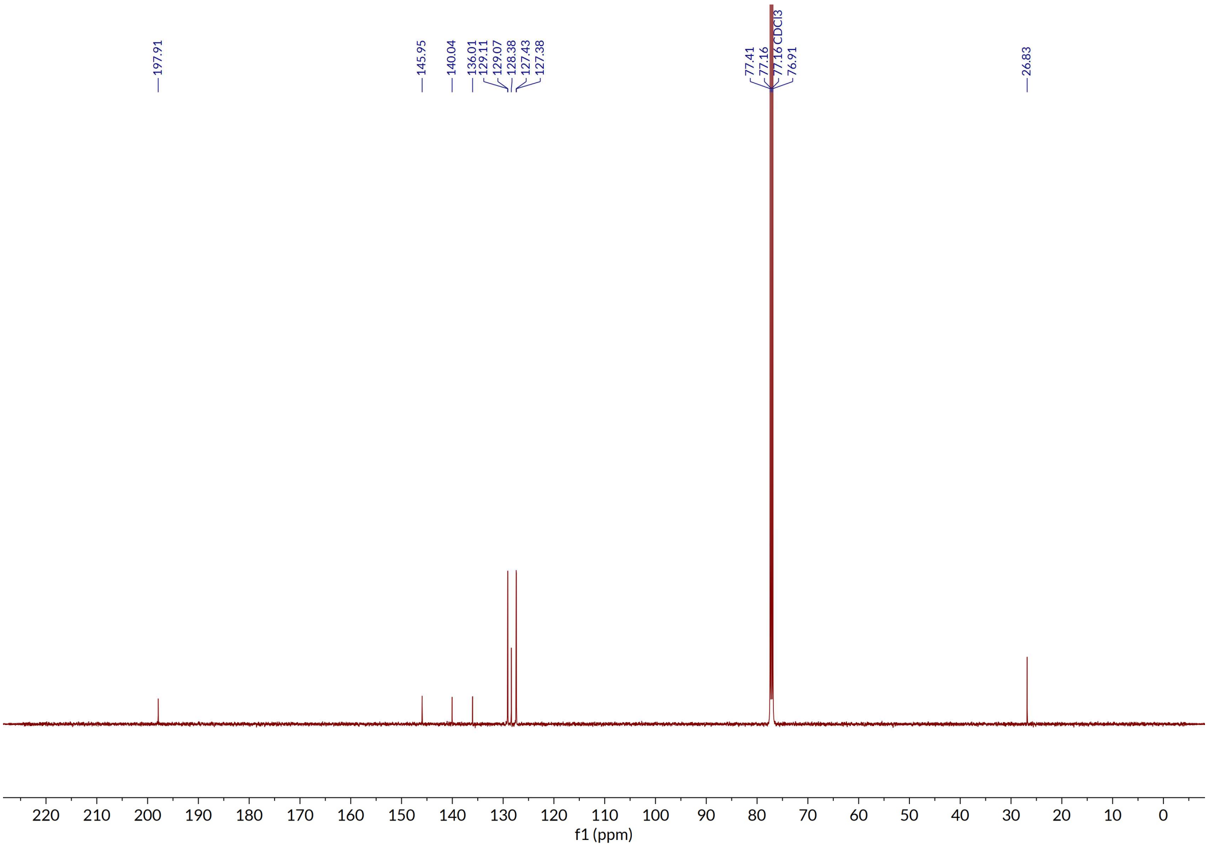

(*S*)-1-(Pyridin-2-yl)ethan-1-ol (20a)

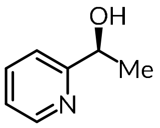

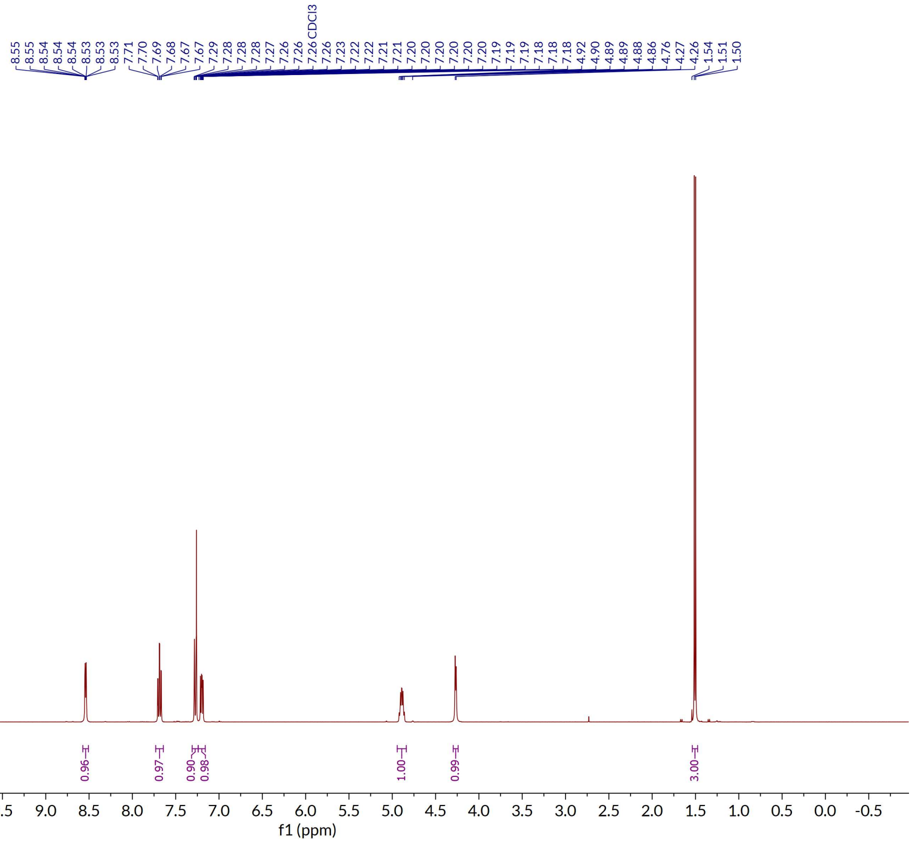

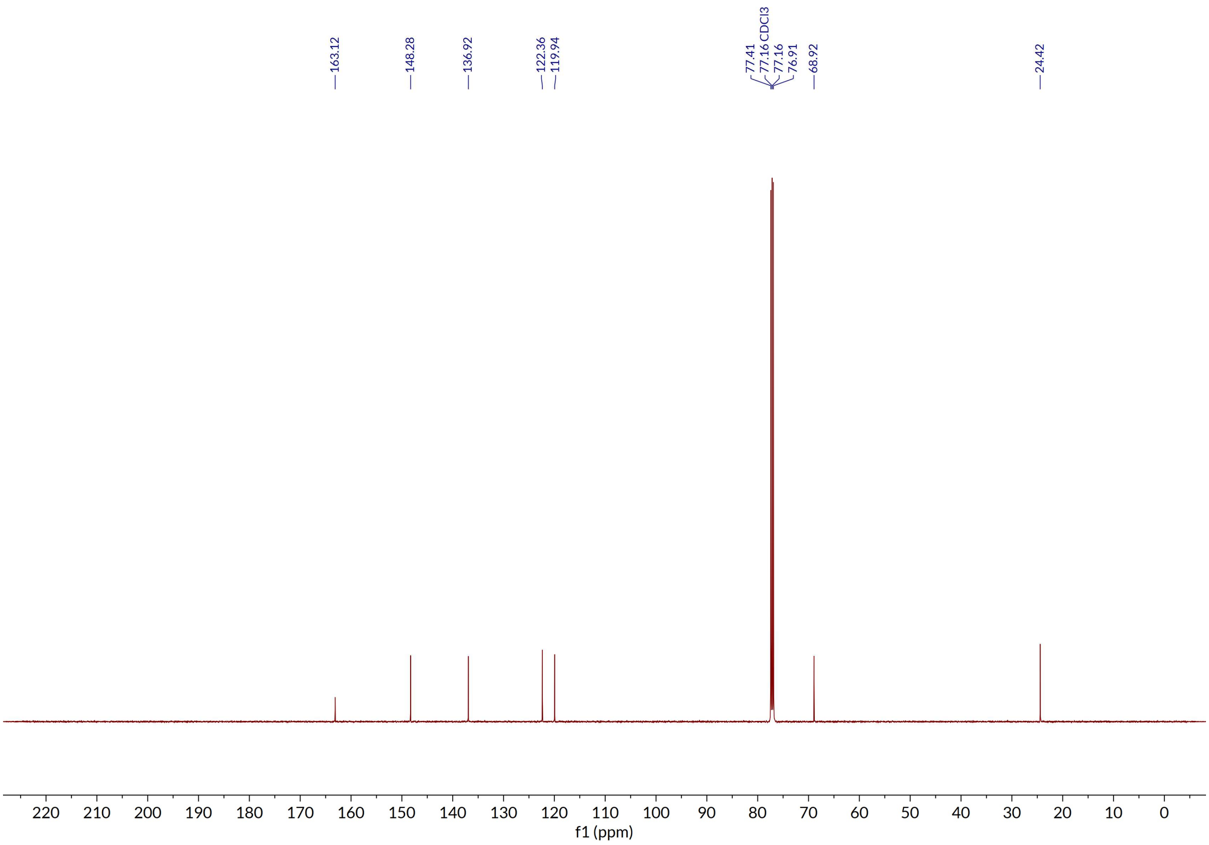

(*S*)-1-(Pyridin-3-yl)ethan-1-ol (21a)

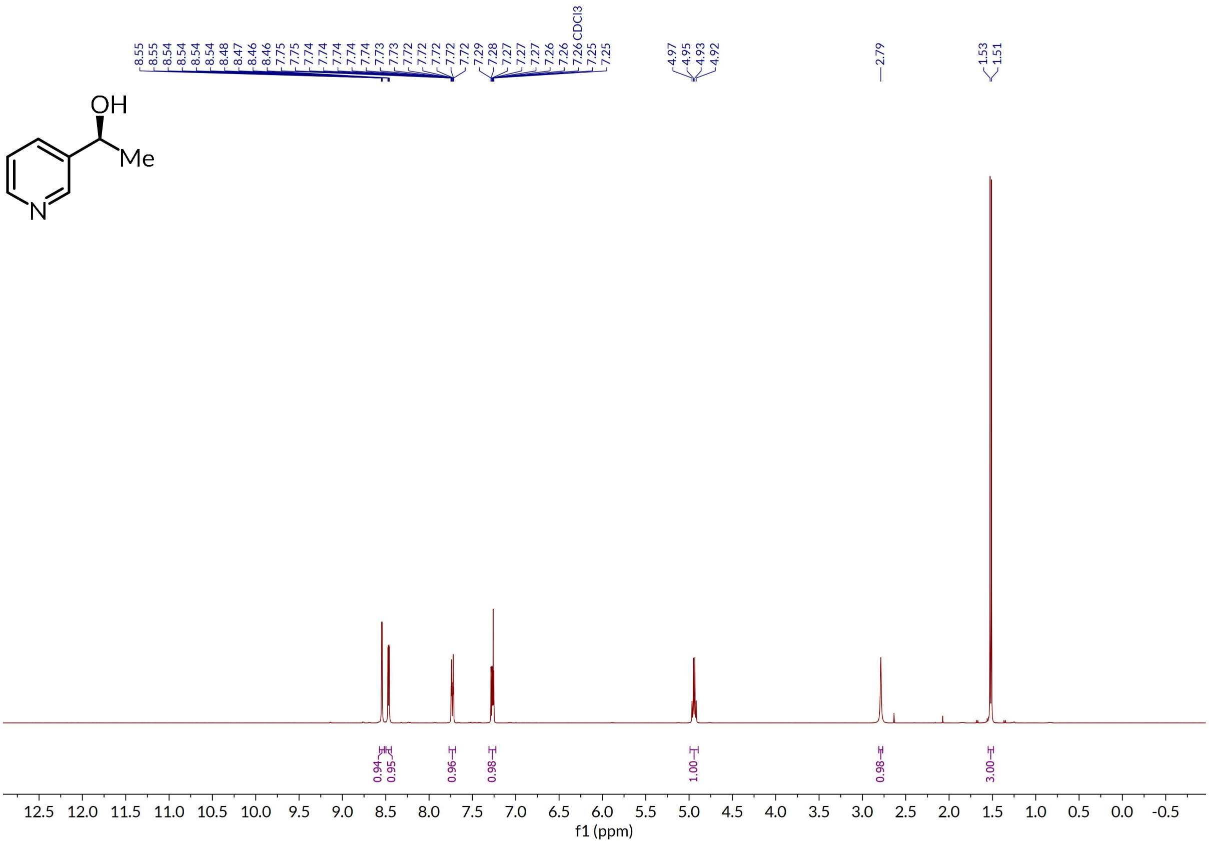

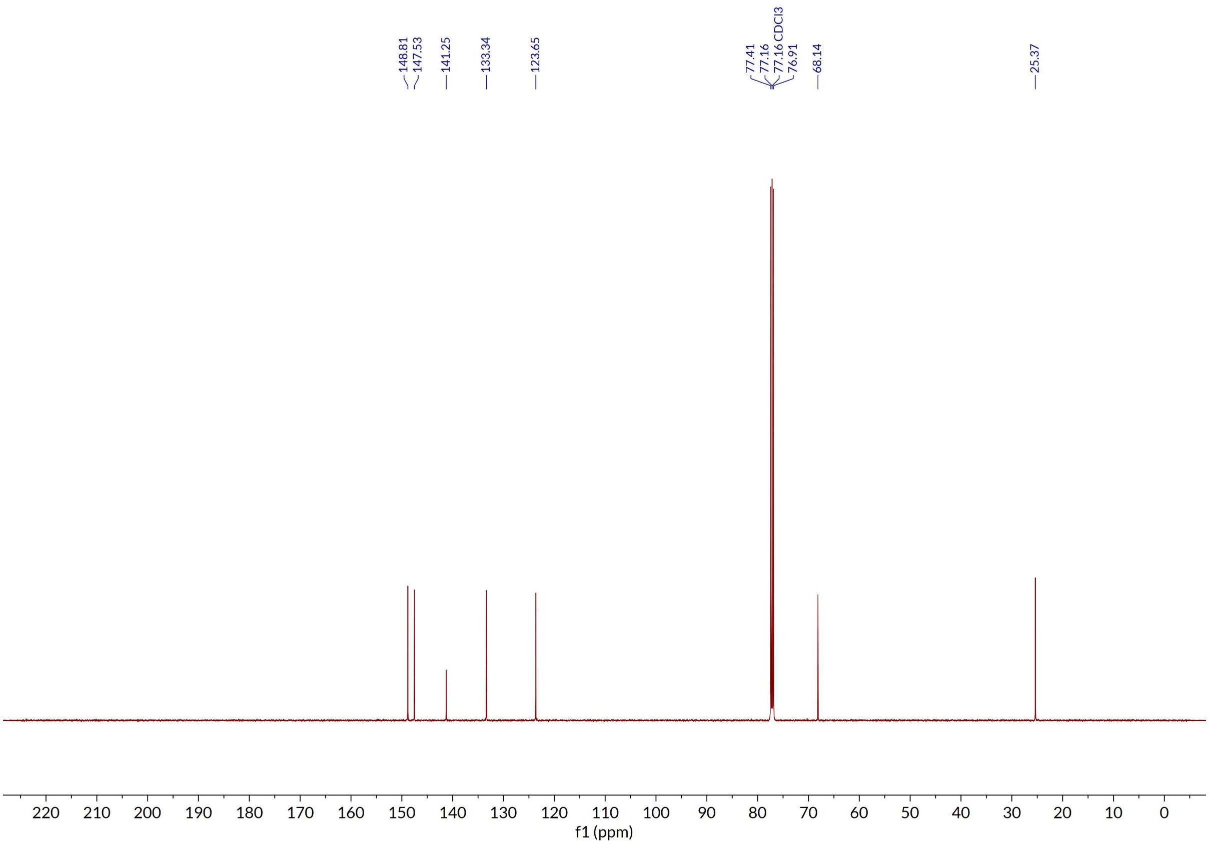

1-(Pyridin-3-yl)ethan-1-one (21b)

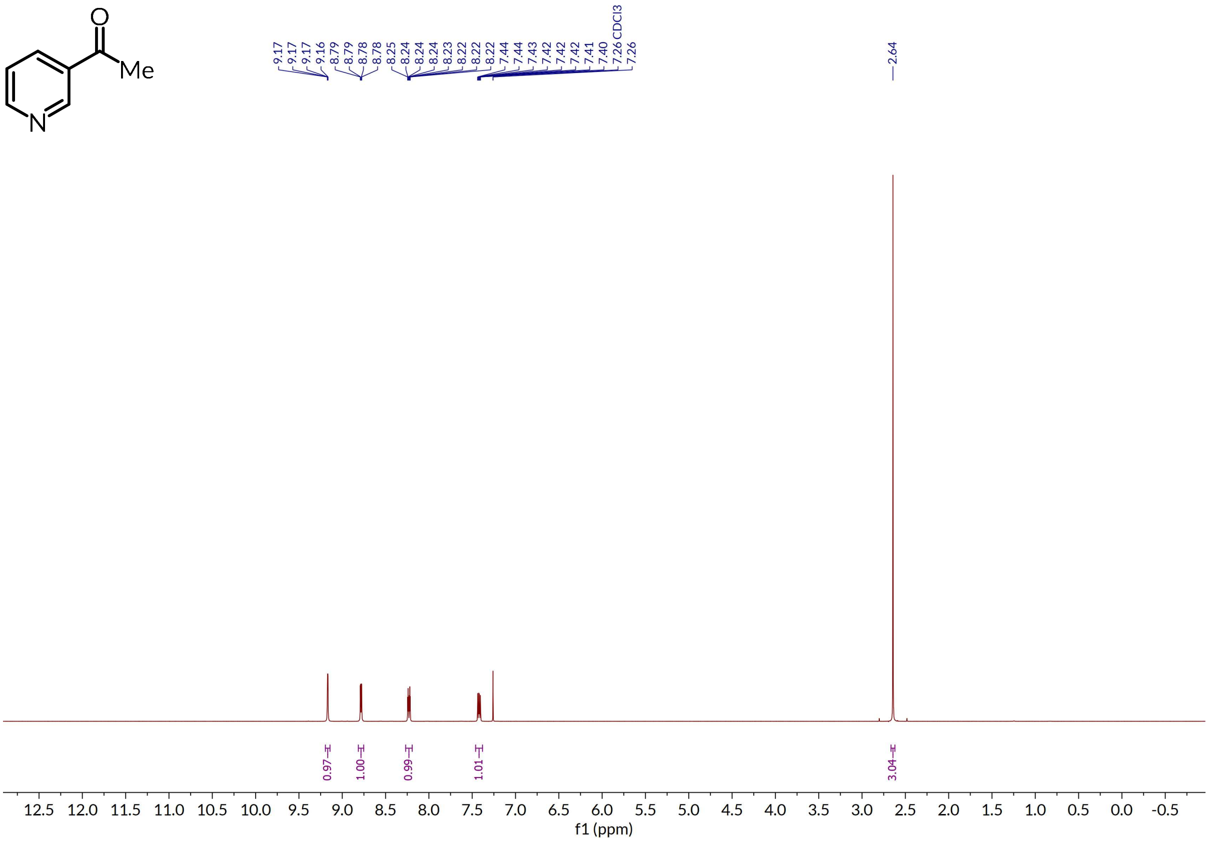

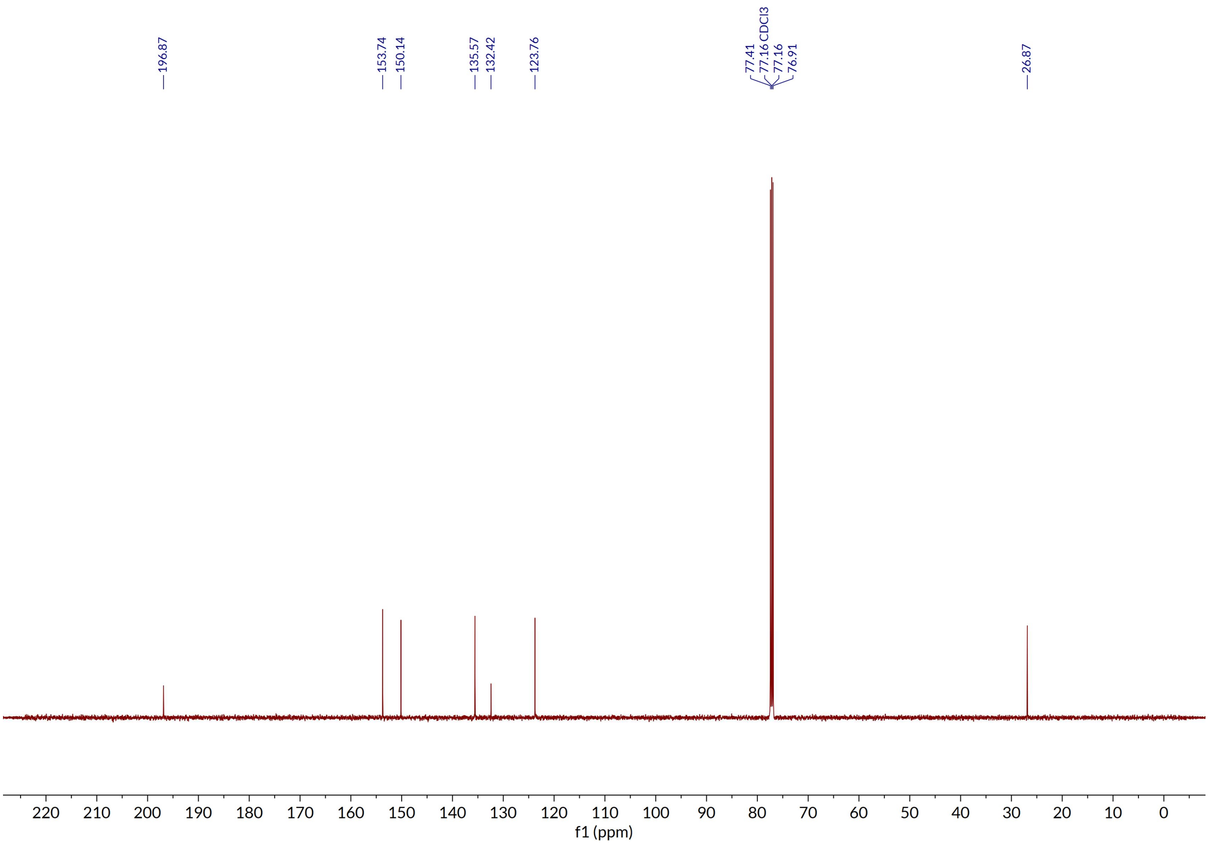

(*S*)-1-(Pyridin-4-yl)ethan-1-ol (22a)

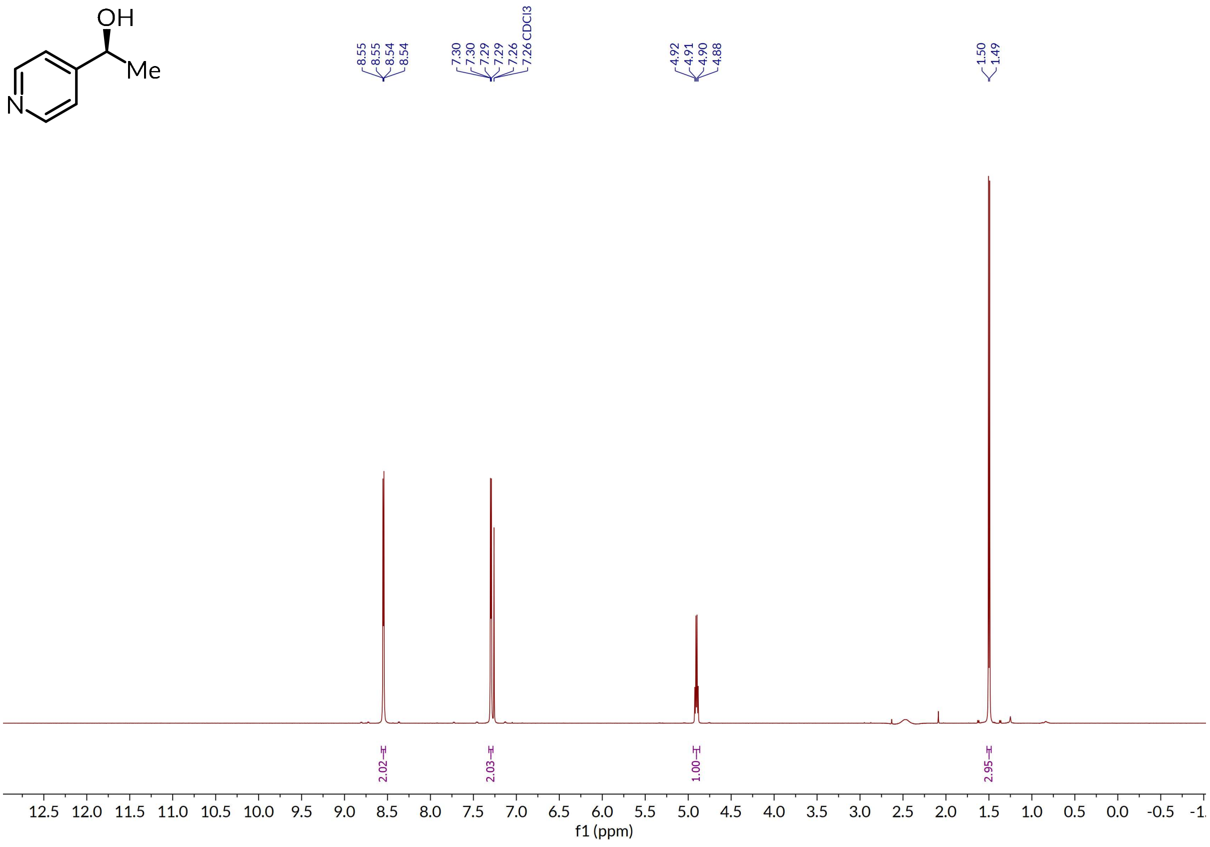

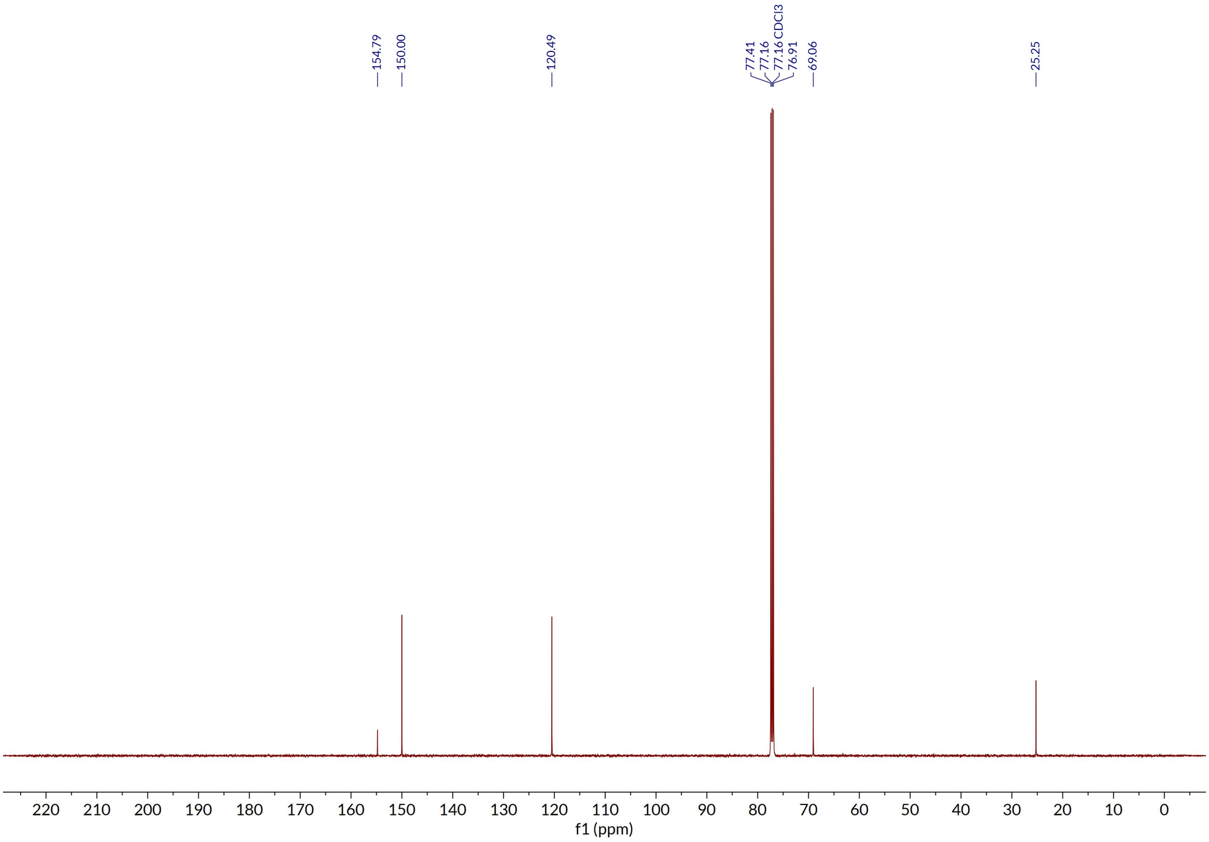

(*S*)-1-(Pyrimidin-4-yl)ethan-1-ol (23a)

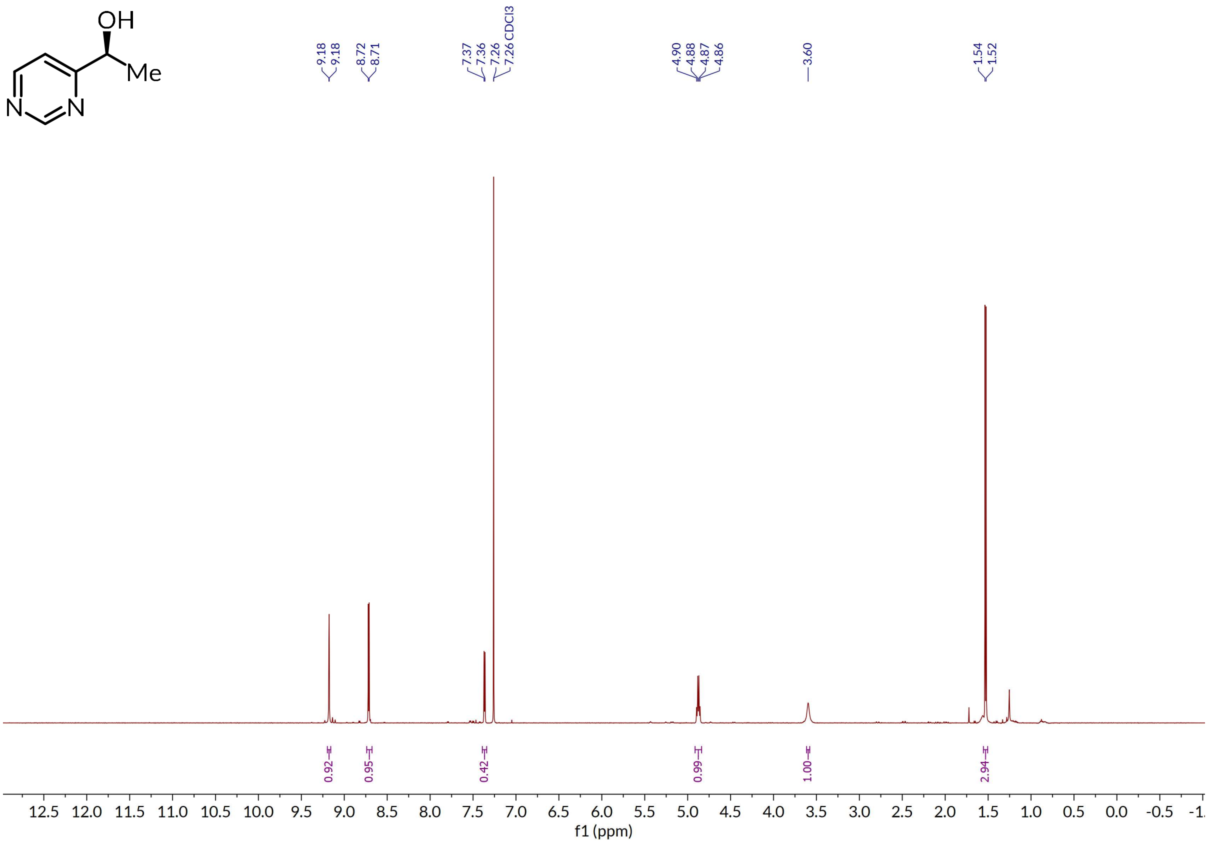

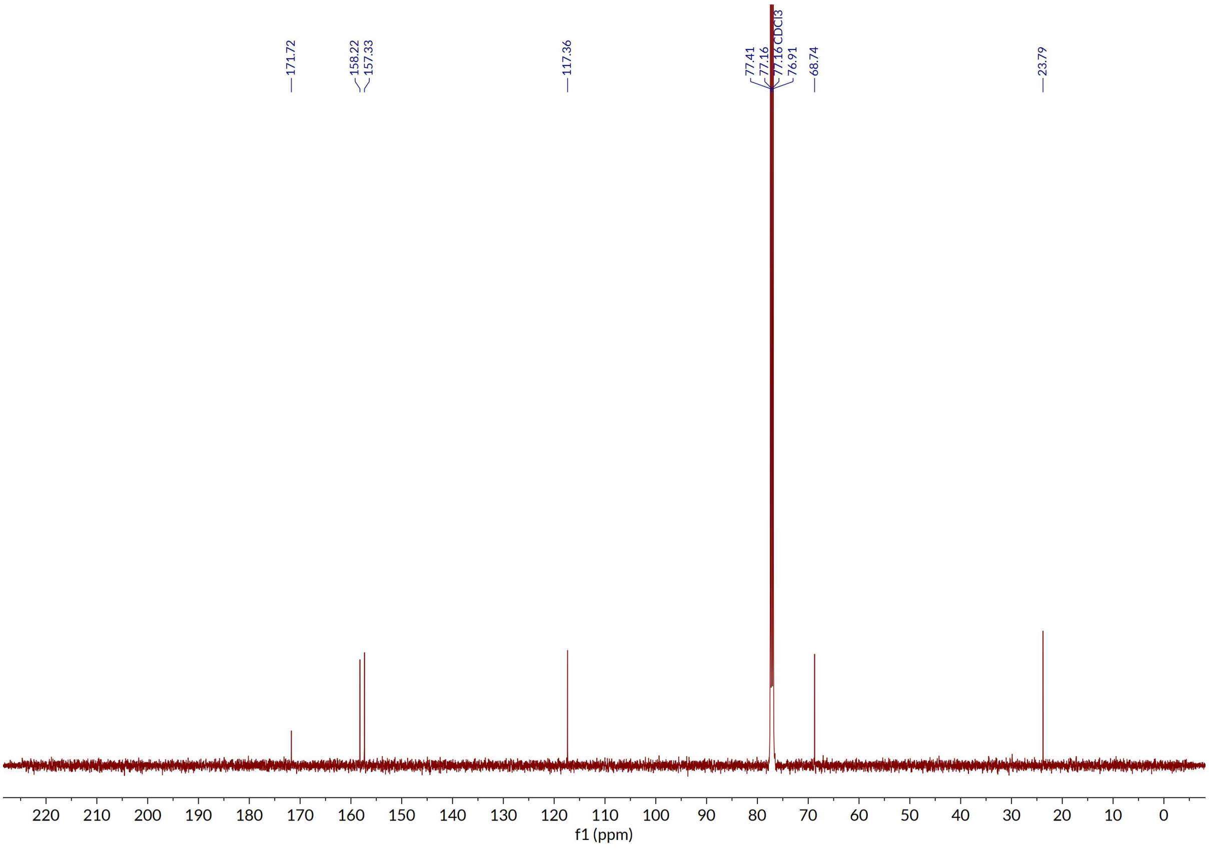

(*S*)-1-(Thiophen-2-yl)ethan-1-ol (24a)

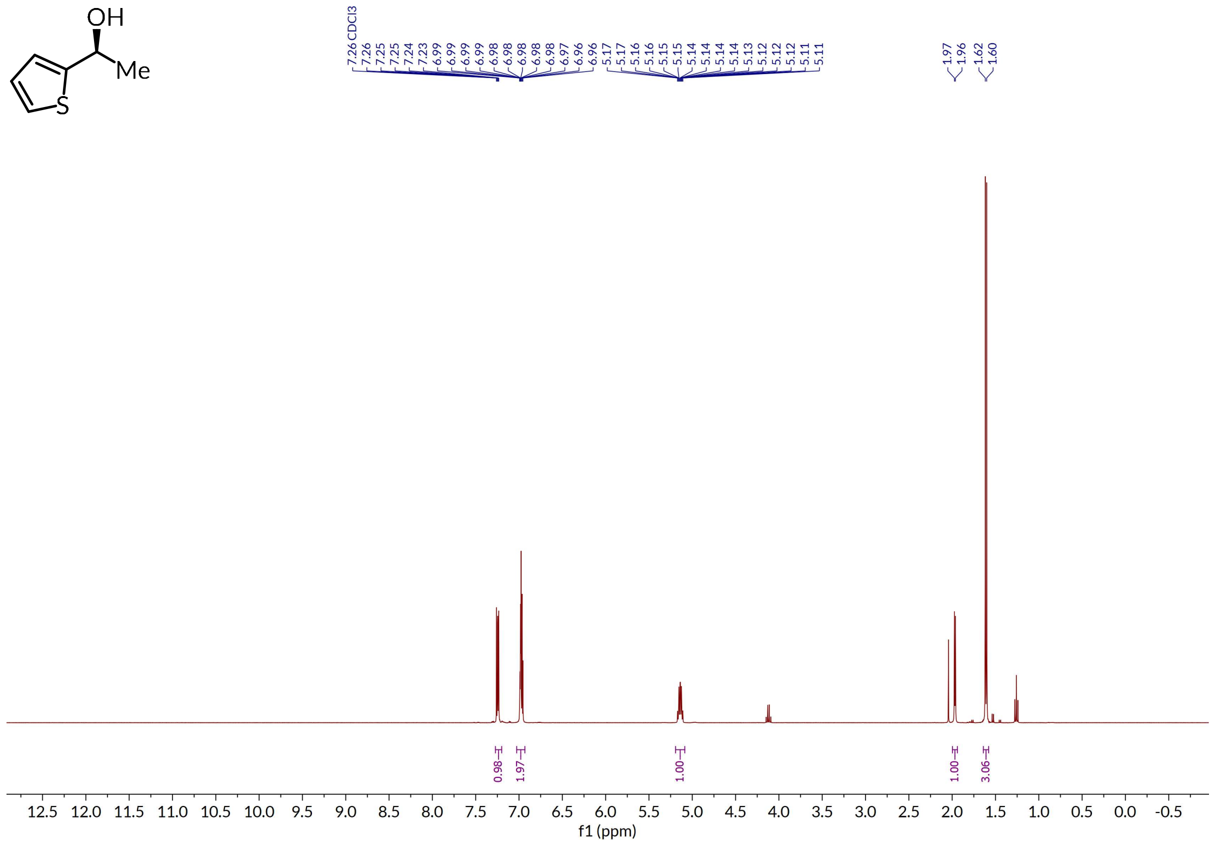

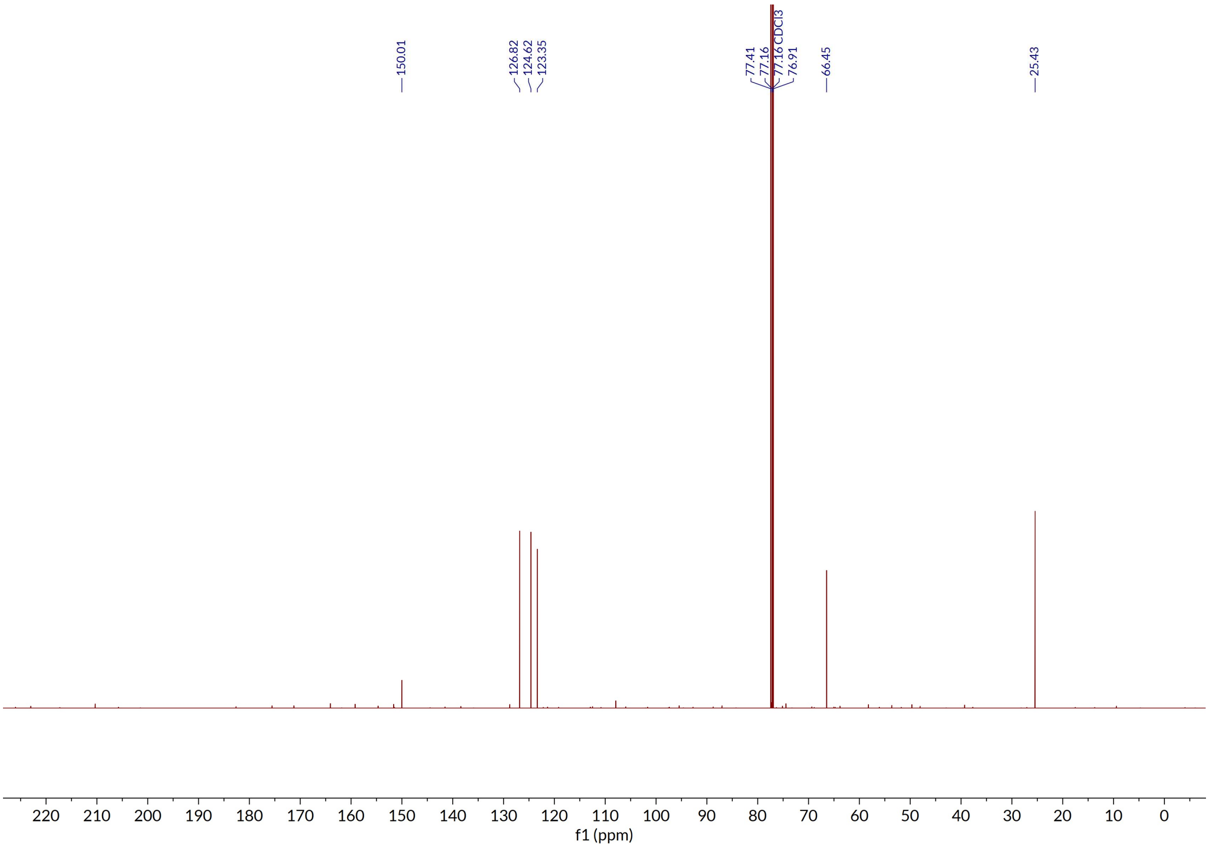

1-(Thiophen-2-yl)ethan-1-one (24b)

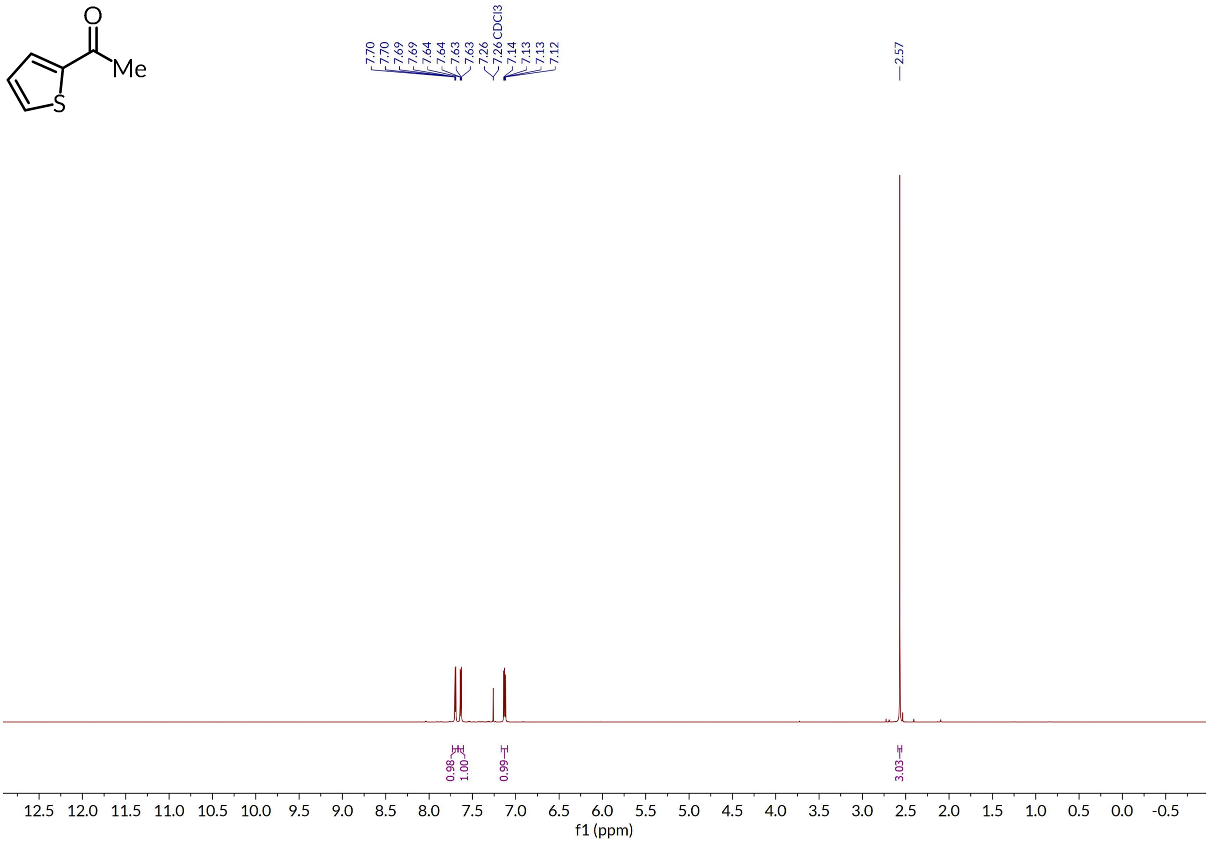

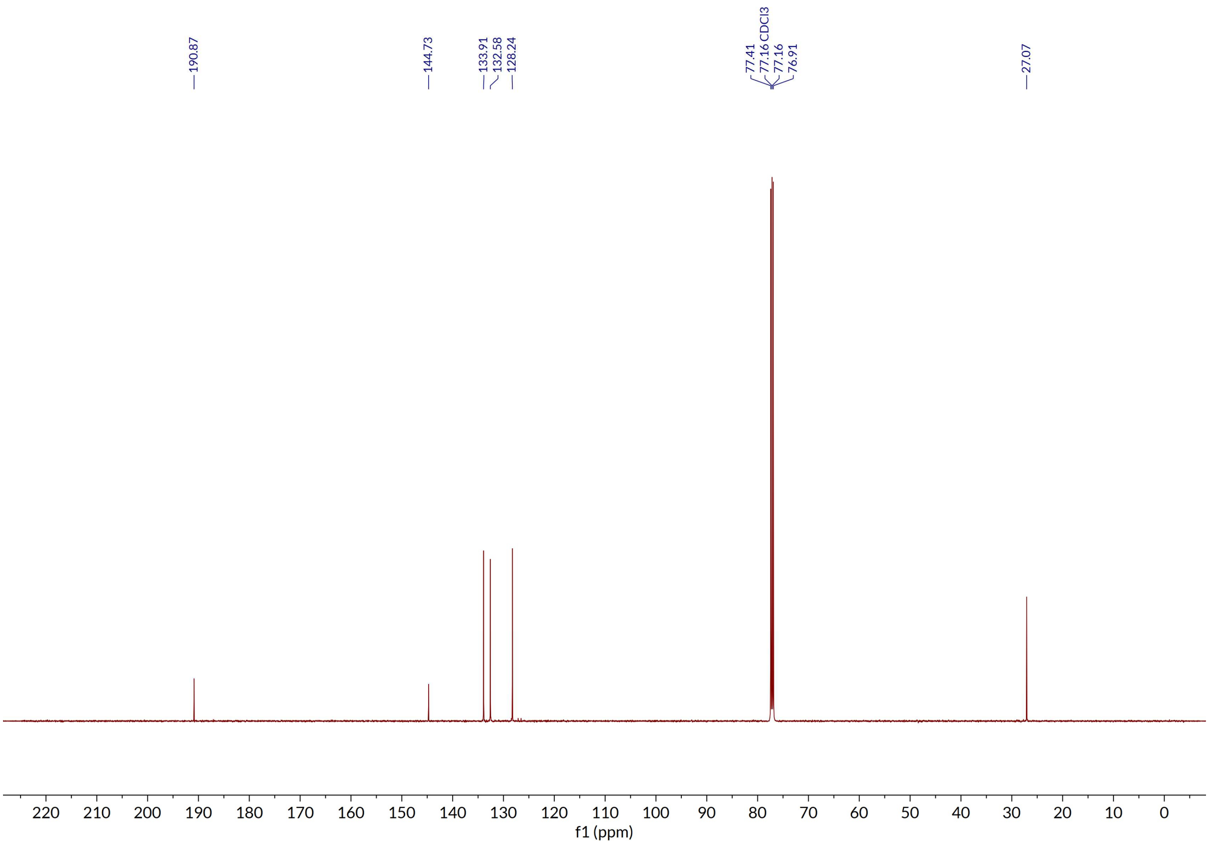

(*S*)-1-Phenylpropan-1-ol (25a)

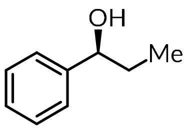

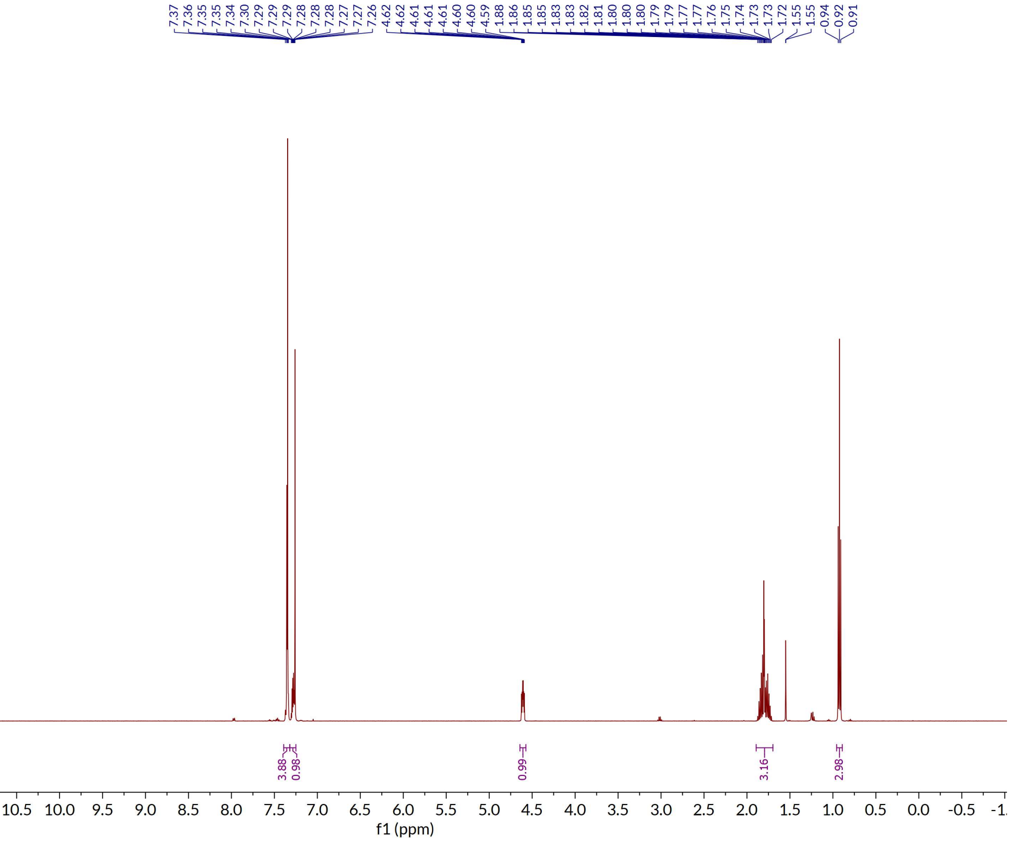

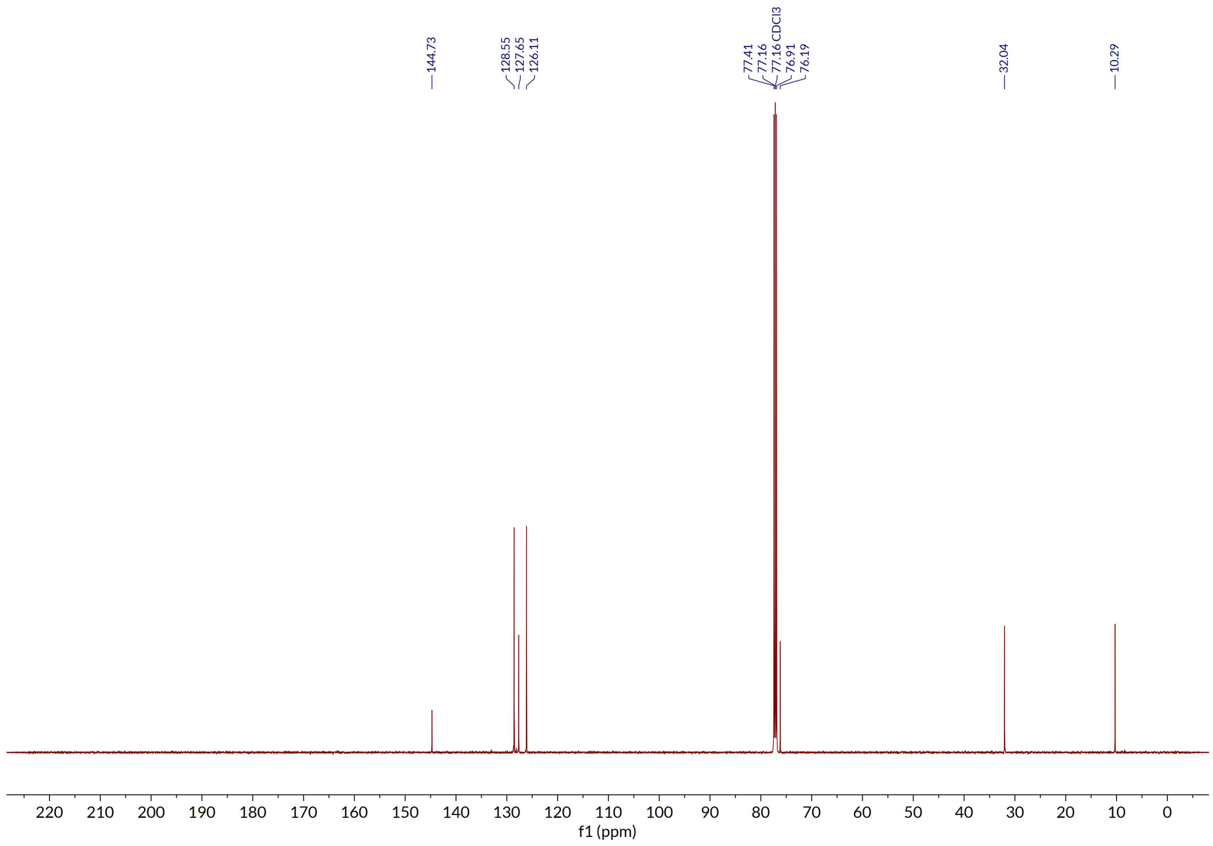

Propiophenone (25b)

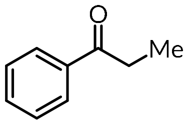

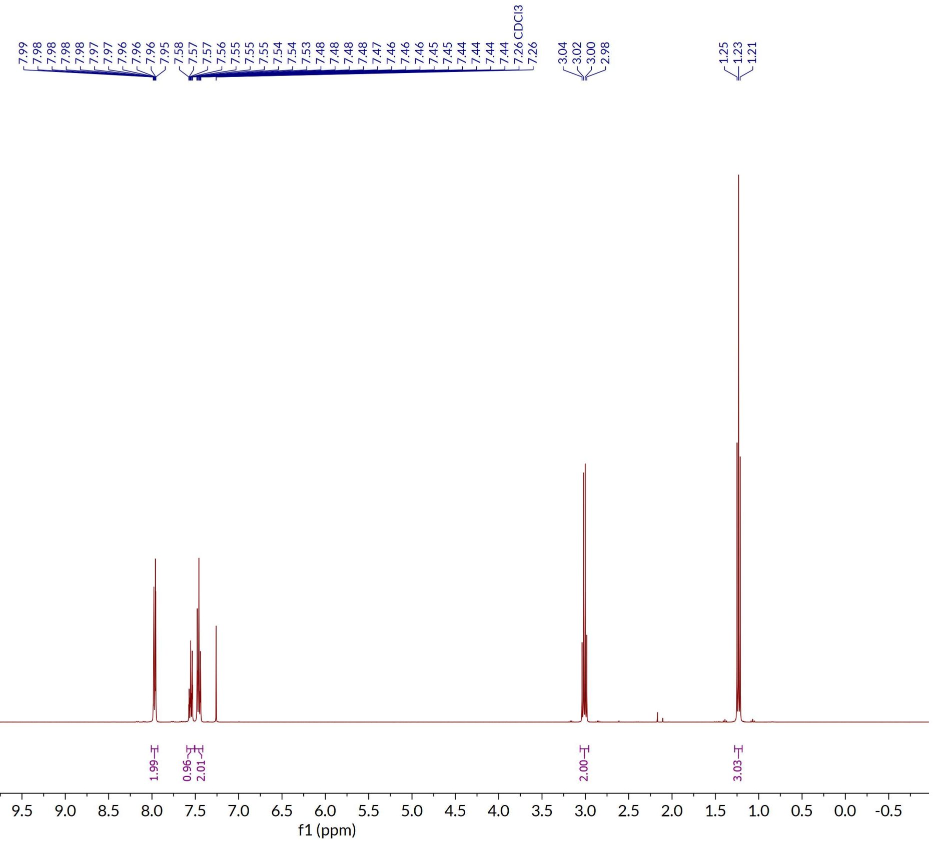

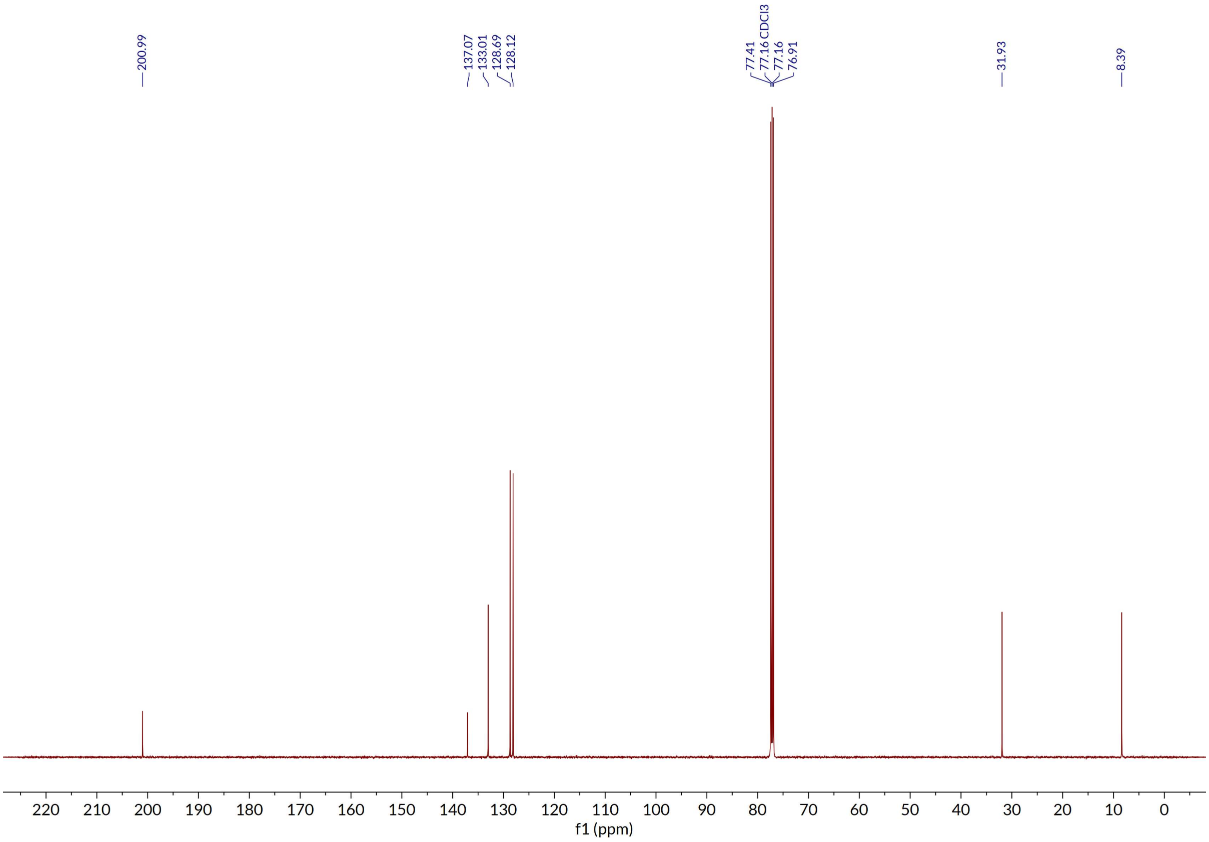

(*S*)-1-phenylbutan-1-ol (26a)

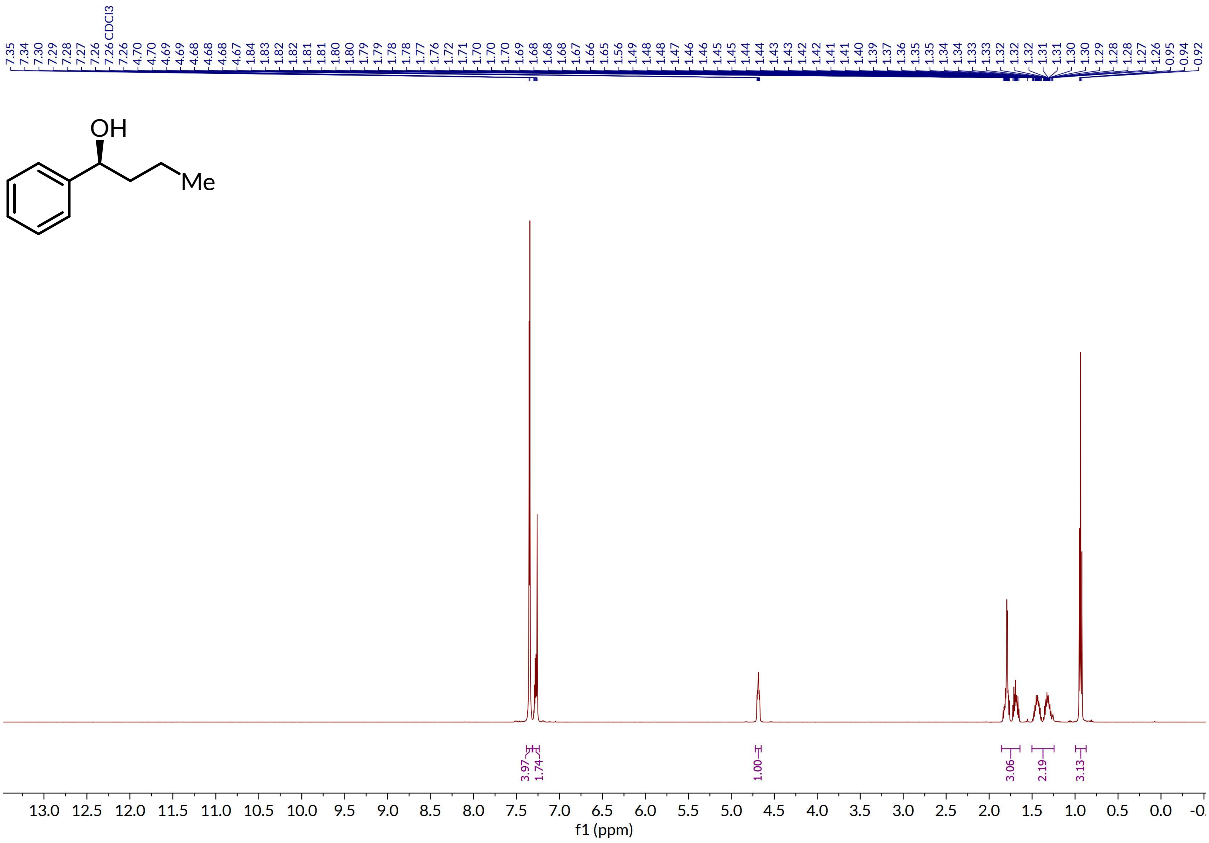

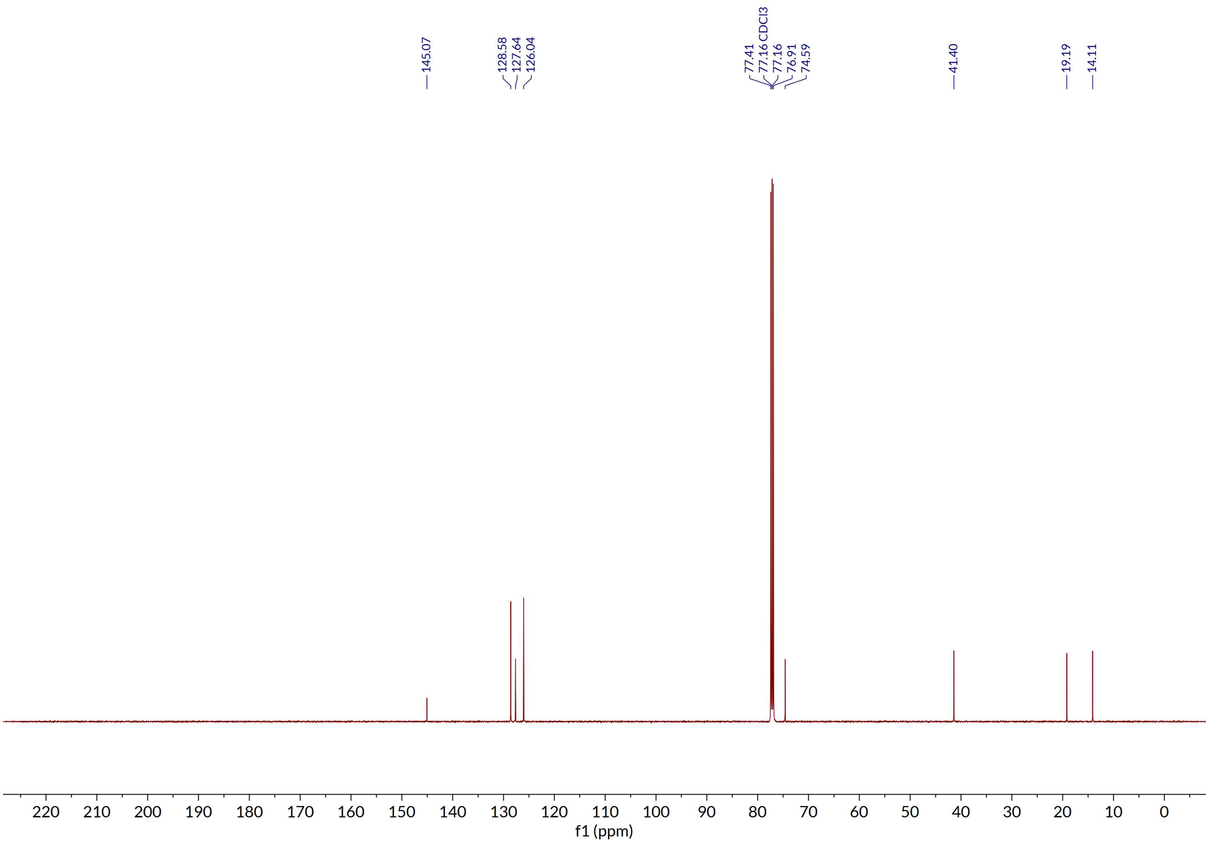

1-Phenylbutan-1-one (26b)

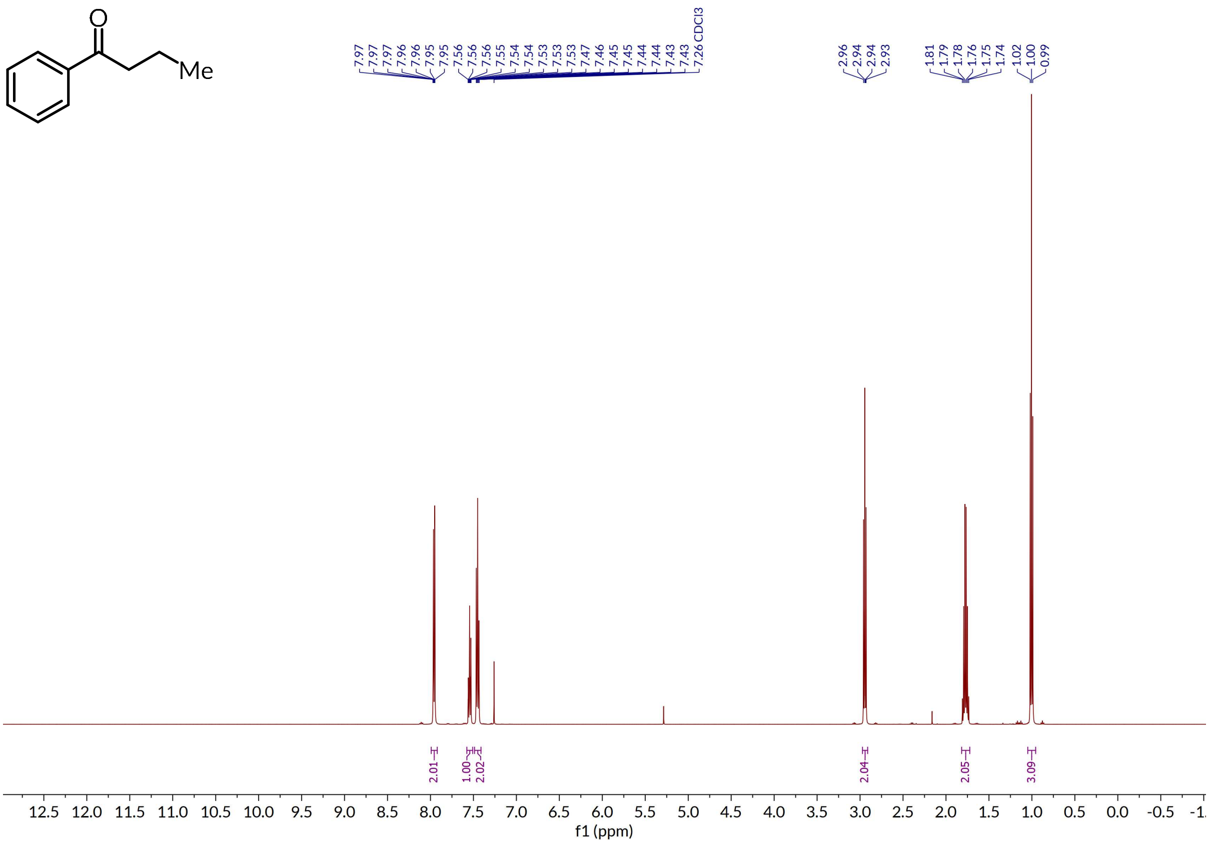

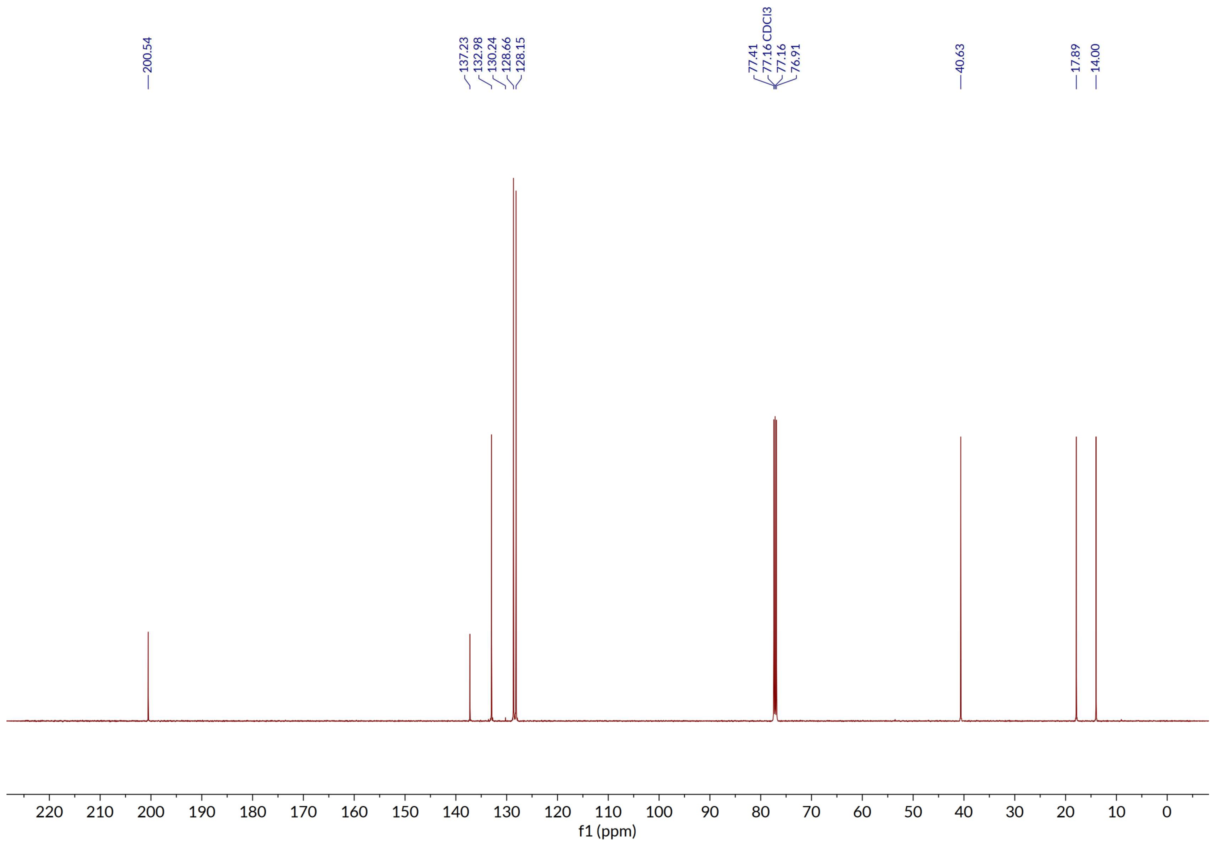
200.54
137.23
132.98
130.24
128.66
128.15
77.41
77.16 CDCl3
77.16
76.91
40.63
17.89
14.00
220 210 200 190 180 170 160 150 140 130 120 110 100 90 80 70 60 50 40 30 20 10 0
f1 (ppm)

11 Chiral HPLC traces

(*S*)-1-(4-Fluorophenyl)ethan-1-ol (1a)

Racemic

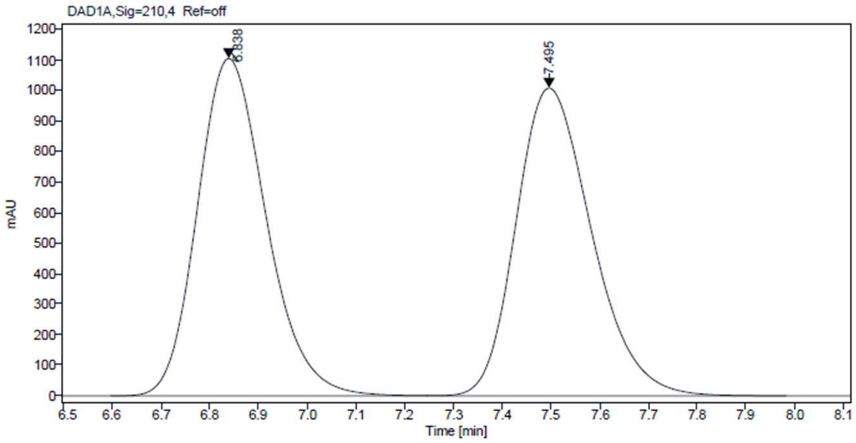

Signal: DAD1A,Sig=210,4 Ref=off

RT [min]	Type	Width [min]	Area	Height	Area%	Name
6.838	MM m	0.6502	10842.0202	1106.9173	49.9294	
7.495	MM m	0.7355	10872.6953	1009.8191	50.0706	

Enantioenriched

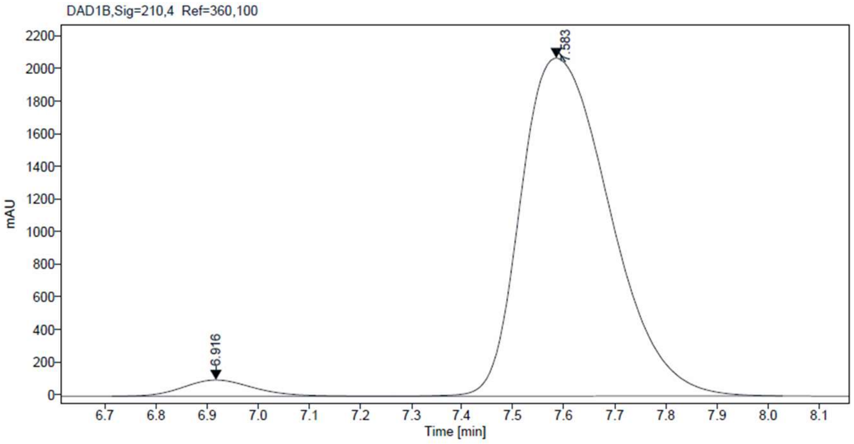

Signal: DAD1B,Sig=210,4 Ref=360,100

RT [min]	Type	Width [min]	Area	Height	Area%	Name
6.916	MM m	0.5480	956.5772	99.6116	3.6659	
7.583	MM m	0.7673	25137.5076	2075.9338	96.3341	

(*S*)-1-Phenylethan-1-ol (2a)

Racemic

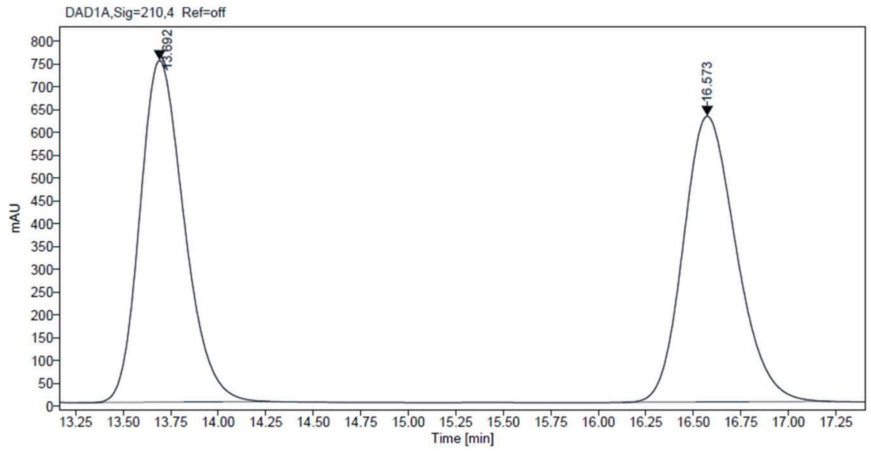

Signal: DAD1A,Sig=210,4 Ref=off

RT [min]	Type	Width [min]	Area	Height	Area%	Name
13.692	MM m	1.0045	12097.1142	749.1978	49.9709	
16.573	MM m	1.0924	12111.2131	626.6679	50.0291	

Enantioenriched

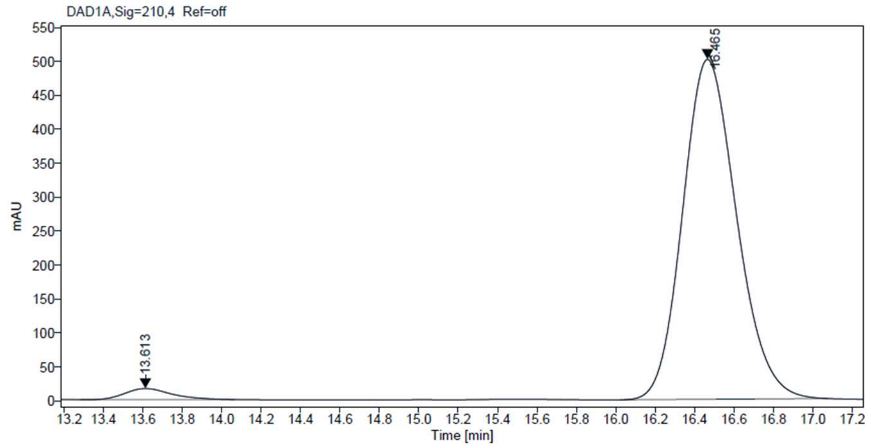

Signal: DAD1A,Sig=210,4 Ref=off

RT [min]	Type	Width [min]	Area	Height	Area%	Name
13.613	MM m	0.7830	269.9807	16.3666	2.7565	
16.465	MM m	1.0542	9524.3543	500.8166	97.2435	

(*S*)-1-(2-Fluorophenyl)ethan-1-ol (3a)

Racemic

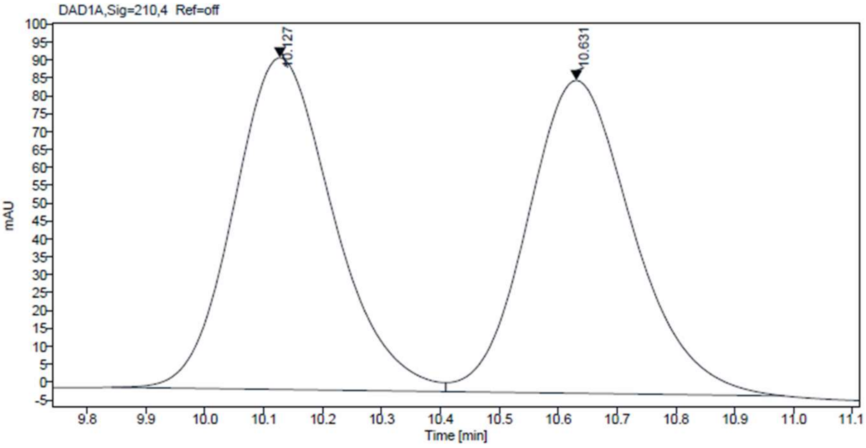

Signal: DAD1A,Sig=210,4 Ref=off

RT [min]	Type	Width [min]	Area	Height	Area%	Name
10.127	MM m	0.5673	1089.2035	92.7332	50.0191	
10.631	MM m	0.5752	1088.3726	87.4411	49.9809	

Enantioenriched

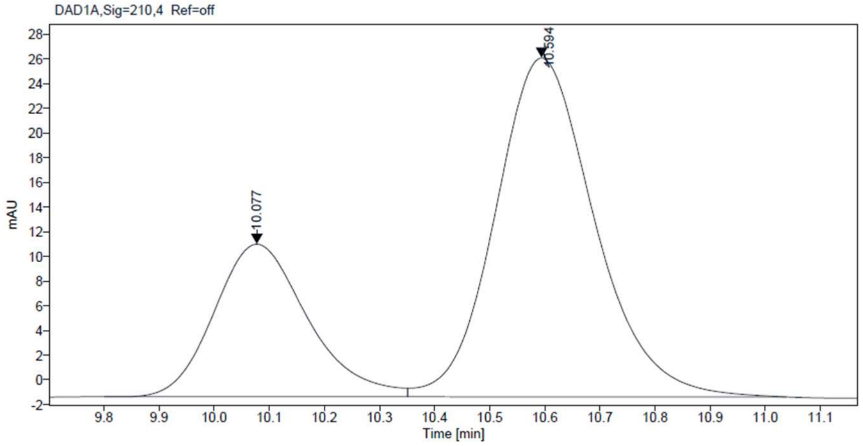

Signal: DAD1A,Sig=210,4 Ref=off

RT [min]	Type	Width [min]	Area	Height	Area%	Name
10.077	MM m	0.5509	145.7160	12.3649	29.7553	
10.594	MM m	0.6874	343.9980	27.4707	70.2447	

(*S*)-1-(4-Chlorophenyl)ethan-1-ol (4a)

Racemic

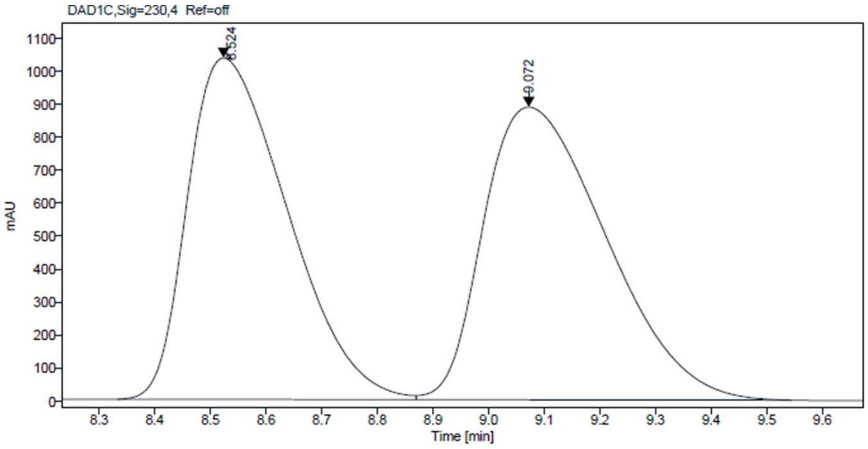

Signal: DAD1C,Sig=230,4 Ref=off

RT [min]	Type	Width [min]	Area	Height	Area%	Name
8.524	MM m	0.5371	12886.2240	1034.8714	48.6983	
9.072	MM m	0.6741	13575.1447	887.1541	51.3017	

Enantioenriched

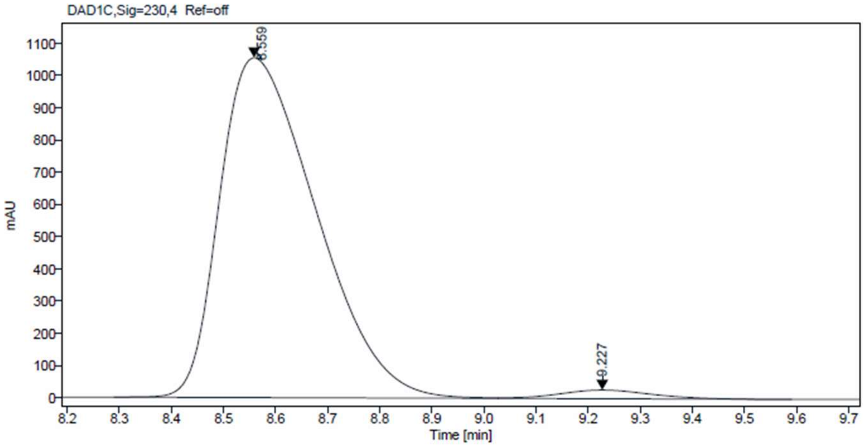

Signal: DAD1C,Sig=230,4 Ref=off

RT [min]	Type	Width [min]	Area	Height	Area%	Name
8.559	MM m	0.7381	13544.5582	1054.6927	97.6279	
9.227	MM m	0.5632	329.0944	26.9612	2.3721	

1-(3-Chlorophenyl)ethan-1-ol (5a)

Racemic

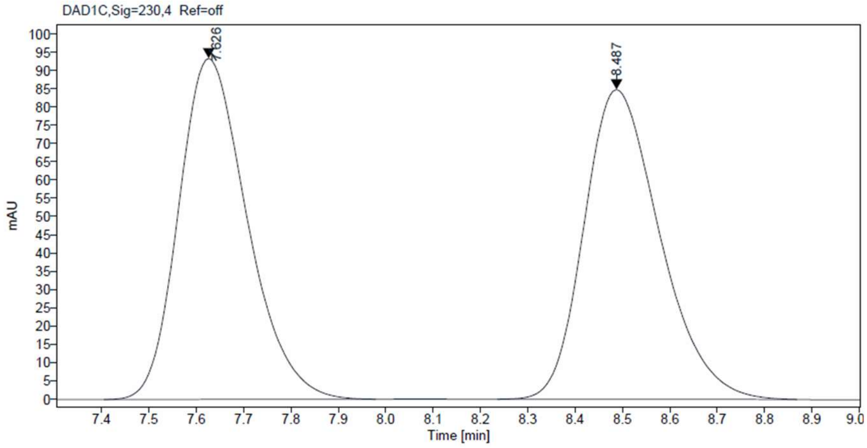

Signal: DAD1C,Sig=230,4 Ref=off

RT [min]	Type	Width [min]	Area	Height	Area%	Name
7.626	MM m	0.5745	939.2609	93.4254	49.8560	
8.487	MM m	0.6330	944.6853	84.8555	50.1440	

Enantioenriched

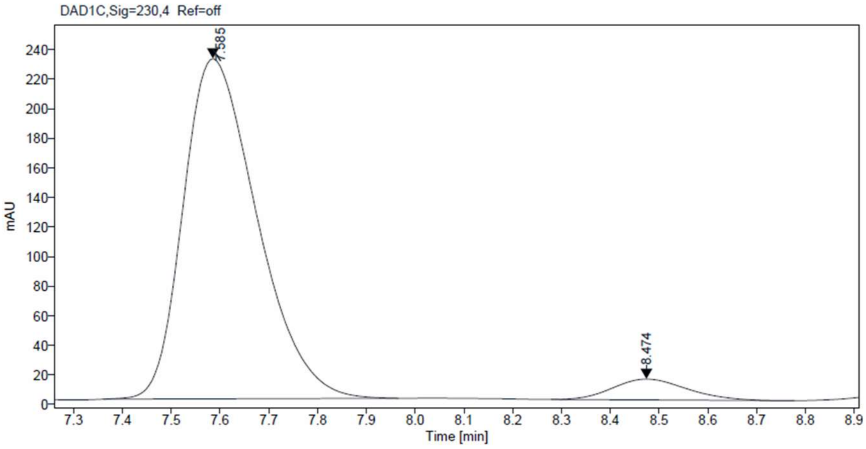

Signal: DAD1C,Sig=230,4 Ref=off

RT [min]	Type	Width [min]	Area	Height	Area%	Name
7.585	MM m	0.6055	2417.2926	230.1006	94.1704	
8.474	MM m	0.4988	149.6418	14.1191	5.8296	

(*S*)-1-(2-Bromophenyl)ethan-1-ol (6a)

Racemic

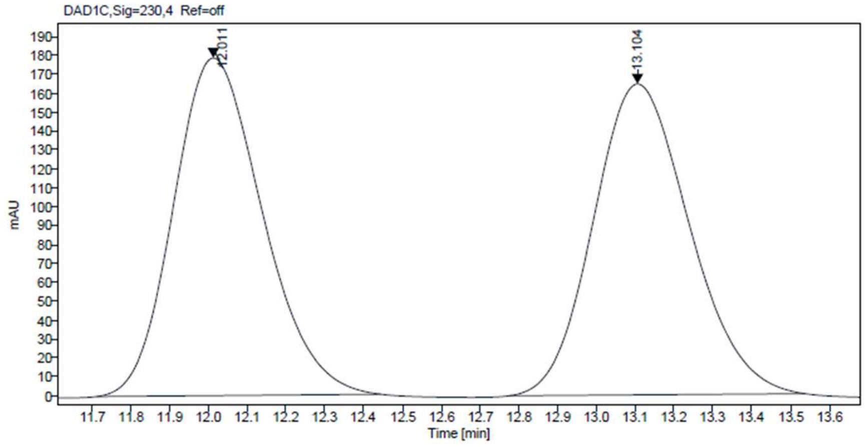

Signal: DAD1C,Sig=230,4 Ref=off

RT [min]	Type	Width [min]	Area	Height	Area%	Name
12.011	MM m	0.7319	2846.7434	178.5855	50.1748	
13.104	MM m	0.7623	2826.9081	164.4484	49.8252	

Enantioenriched

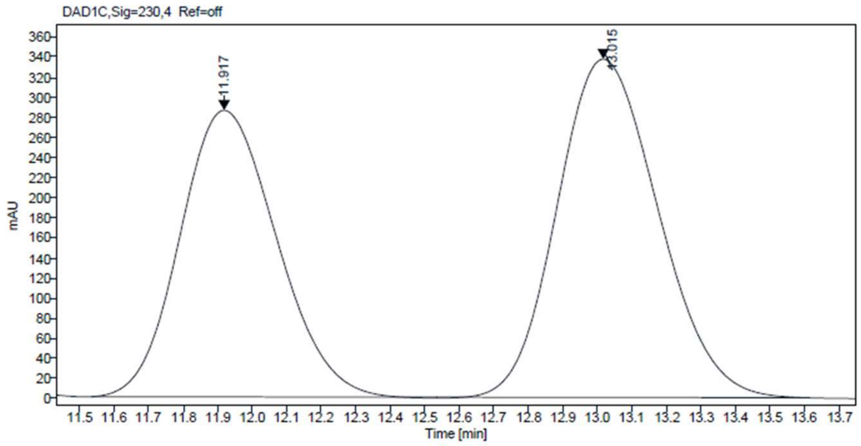

Signal: DAD1C,Sig=230,4 Ref=off

RT [min]	Type	Width [min]	Area	Height	Area%	Name
11.917	MM m	1.0008	5535.6645	285.4600	44.6866	
13.015	MM m	1.0663	6852.0974	337.0368	55.3134	

(*S*)-1-(3-Bromophenyl)ethan-1-ol (7a)

Racemic

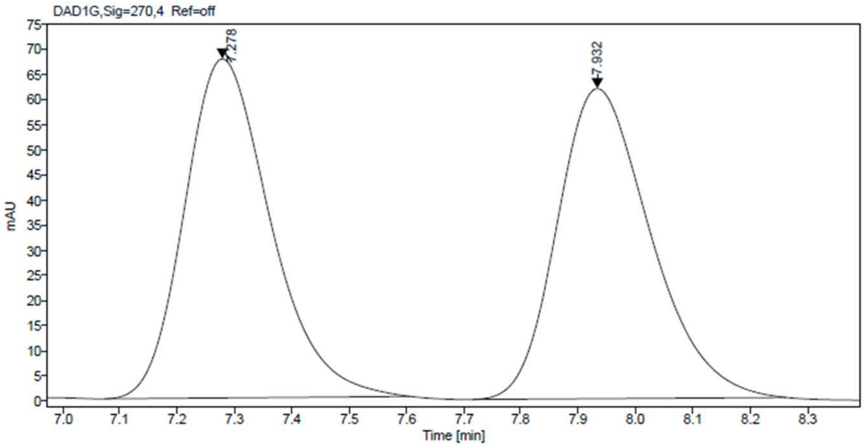

Signal: DAD1G,Sig=270,4 Ref=off

RT [min]	Type	Width [min]	Area	Height	Area%	Name
7.278	MM m	0.5338	704.2219	67.5783	50.1904	
7.932	MM m	0.5473	698.8795	61.8110	49.8096	

Enantioenriched

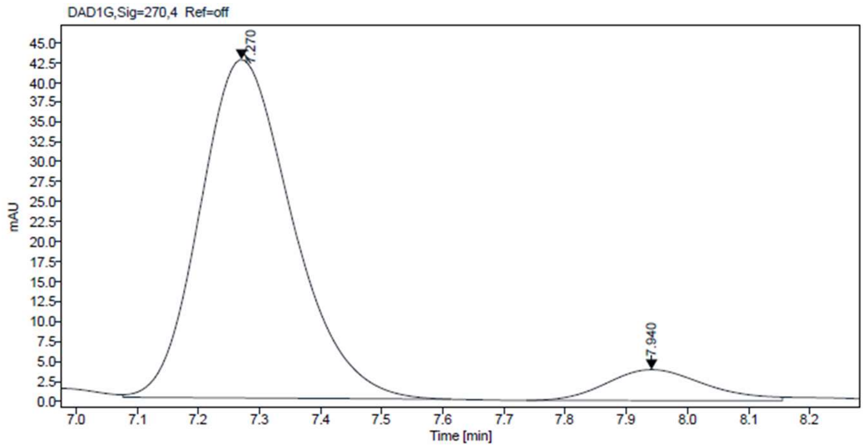

Signal: DAD1G,Sig=270,4 Ref=off

RT [min]	Type	Width [min]	Area	Height	Area%	Name
7.270	MM m	0.5366	444.0381	42.3904	91.1151	
7.940	MM m	0.4185	43.2994	3.8679	8.8849	

(*S*)-1-(4-Bromophenyl)ethan-1-ol (8a)

Racemic

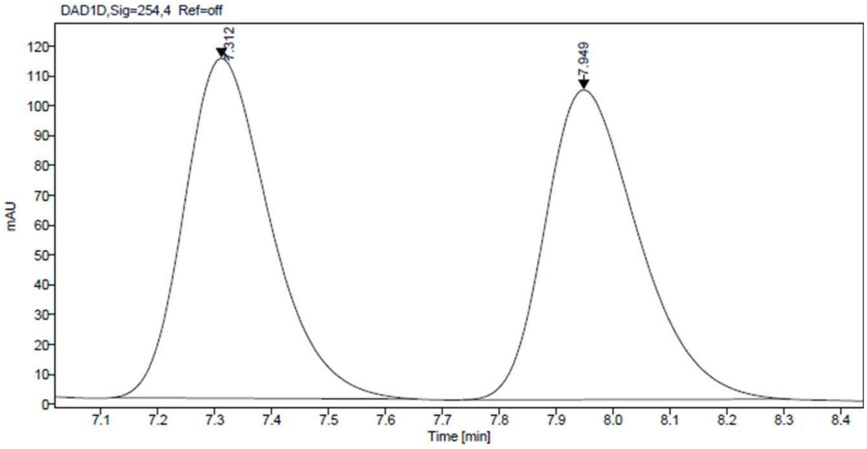

Signal: DAD1D,Sig=254,4 Ref=off

RT [min]	Type	Width [min]	Area	Height	Area%	Name
7.312	MM m	0.5371	1195.2392	114.0296	50.1982	
7.949	MM m	0.5746	1185.8007	103.9227	49.8018	

Enantioenriched

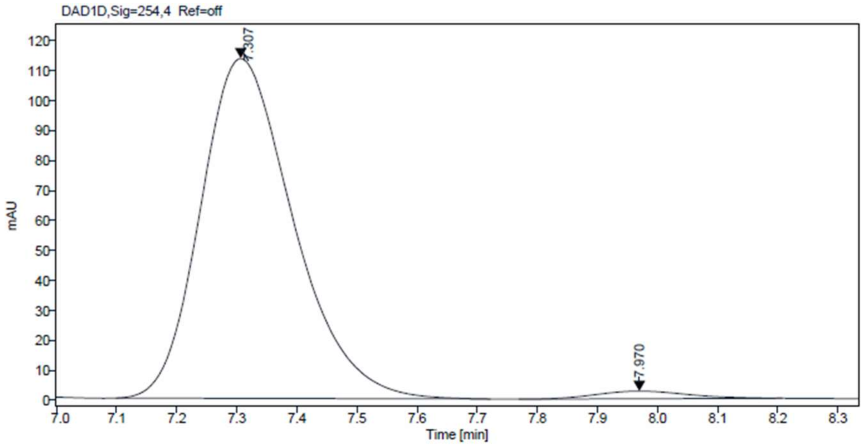

Signal: DAD1D,Sig=254,4 Ref=off

RT [min]	Type	Width [min]	Area	Height	Area%	Name
7.307	MM m	0.6235	1215.4621	113.4511	97.8327	
7.970	MM m	0.4390	26.9261	2.5493	2.1673	

(*S*)-1-(2-Iodophenyl)ethan-1-ol (9a)

Racemic

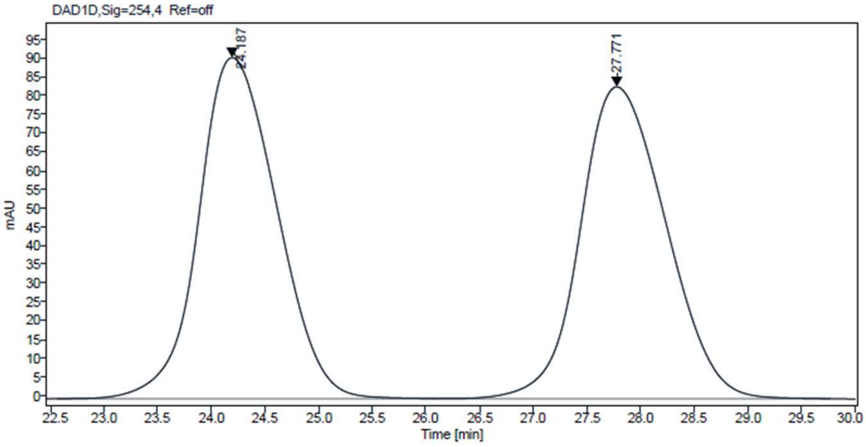

Signal: DAD1D,Sig=254,4 Ref=off

RT [min]	Type	Width [min]	Area	Height	Area%	Name
24.187	MM m	3.5098	4603.8747	90.8616	49.9881	
27.771	MM m	3.6948	4606.0617	83.0938	50.0119	

Enantioenriched

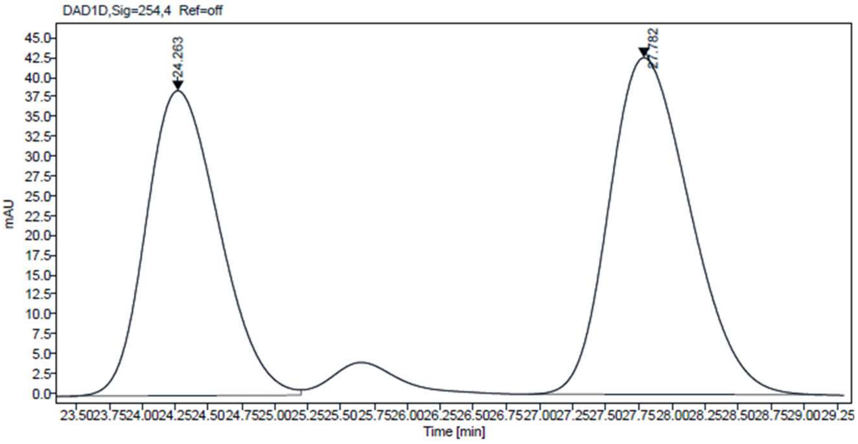

Signal: DAD1D,Sig=254,4 Ref=off

RT [min]	Type	Width [min]	Area	Height	Area%	Name
24.263	MM m	1.7429	1479.6009	38.5881	44.9848	
27.782	MM m	2.2143	1809.5132	42.6045	55.0152	

(*S*)-1-(4-Iodophenyl)ethan-1-ol (10a)

Racemic

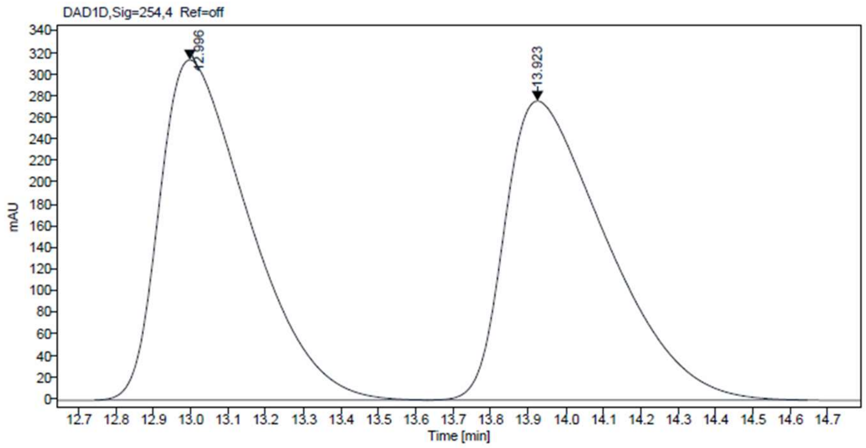

Signal: DAD1D,Sig=254,4 Ref=off

RT [min]	Type	Width [min]	Area	Height	Area%	Name
12.996	MM m	0.8879	5224.5297	314.4477	49.9882	
13.923	MM m	1.0124	5226.9863	276.3573	50.0118	

Enantioenriched

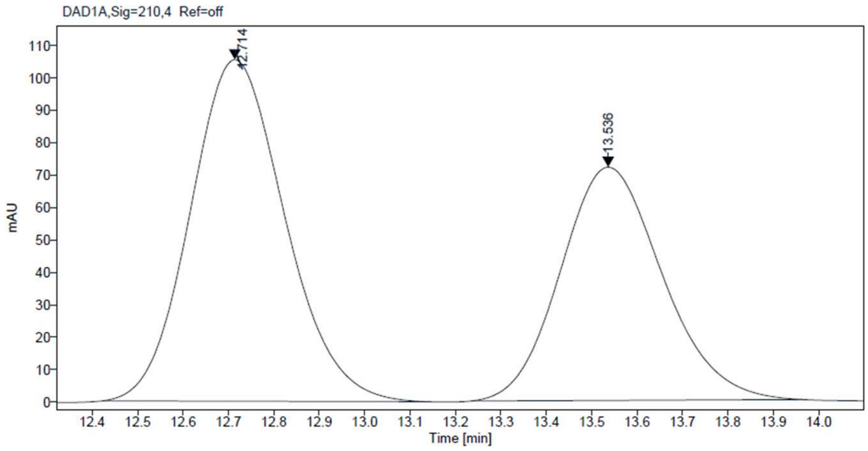

Signal: DAD1A,Sig=210,4 Ref=off

RT [min]	Type	Width [min]	Area	Height	Area%	Name
12.714	MM m	0.7125	1523.8543	105.3191	57.6397	
13.536	MM m	0.7305	1119.9052	71.9379	42.3603	

(*S*)-1-(3-Methoxyphenyl)ethan-1-ol (11a)

Racemic

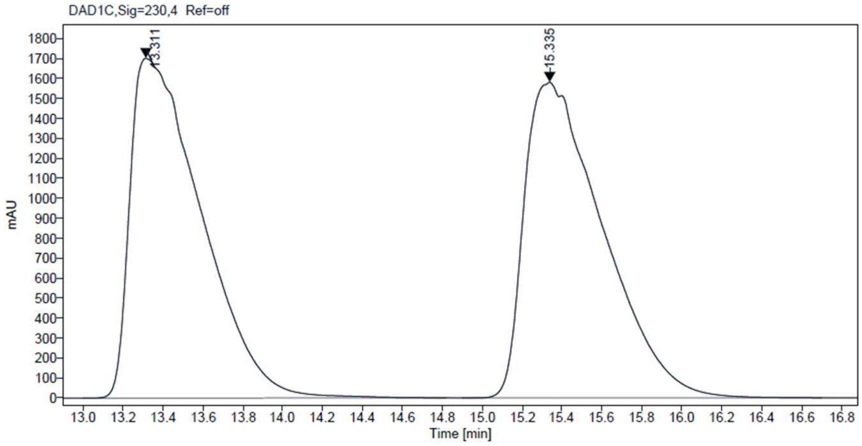

Signal: DAD1C,Sig=230,4 Ref=off

RT [min]	Type	Width [min]	Area	Height	Area%	Name
13.311	MM m	1.8223	42297.4645	1703.5981	49.4077	
15.335	MM m	1.8048	43311.5187	1582.4097	50.5923	

Enantioenriched

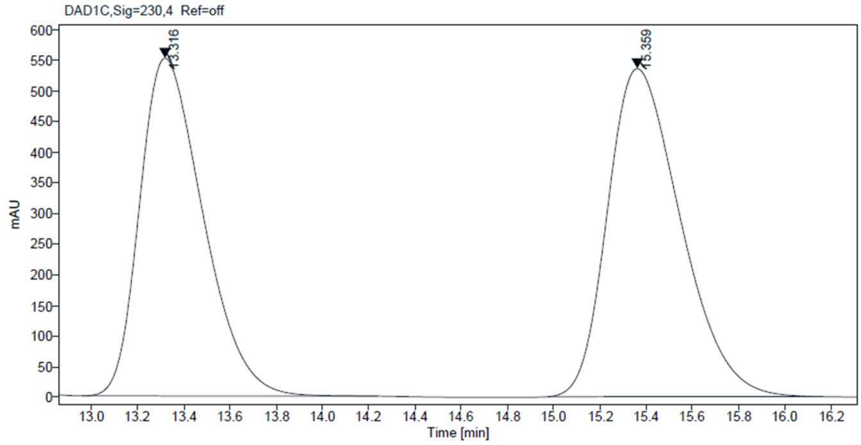

Signal: DAD1C,Sig=230,4 Ref=off

RT [min]	Type	Width [min]	Area	Height	Area%	Name
13.316	MM m	1.2810	10507.2768	551.7723	47.1644	
15.359	MM m	1.1708	11770.6986	535.9346	52.8356	

(*S*)-1-(4-Methoxyphenyl)ethan-1-ol (12a)

Racemic

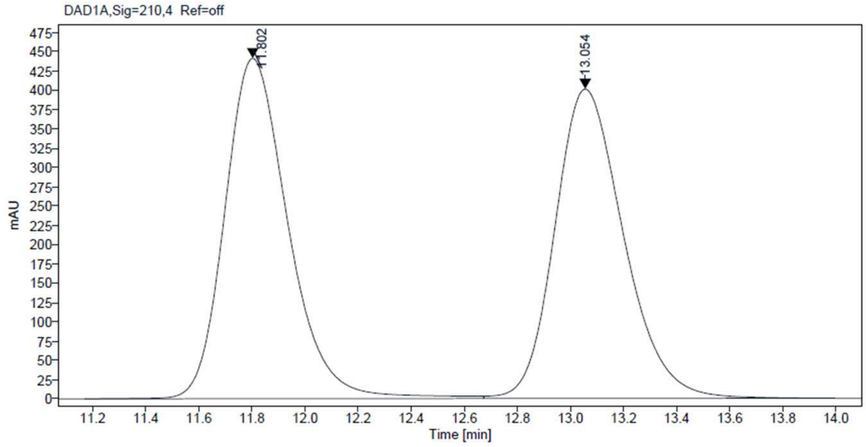

Signal: DAD1A,Sig=210,4 Ref=off

RT [min]	Type	Width [min]	Area	Height	Area%	Name
11.802	MM m	1.5028	7297.6770	440.8163	50.3421	
13.054	MM m	1.2650	7198.5009	400.6441	49.6579	

Enantioenriched

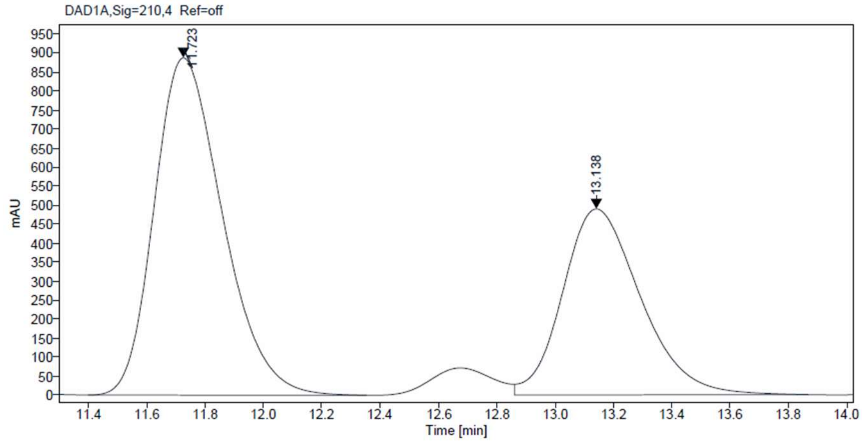

Signal: DAD1A,Sig=210,4 Ref=off

RT [min]	Type	Width [min]	Area	Height	Area%	Name
11.723	MM m	0.9552	14419.7180	886.7551	61.3863	
13.138	MM m	1.0079	9070.4157	489.0203	38.6137	

(*S*)-1-(4-(Trifluoromethyl)phenyl)ethan-1-ol (13a)

Racemic

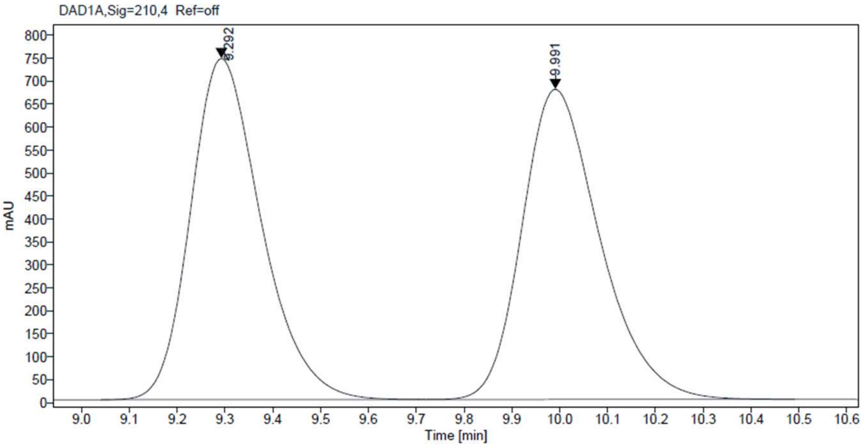

Signal: DAD1A,Sig=210,4 Ref=off

RT [min]	Type	Width [min]	Area	Height	Area%	Name
9.292	MM m	0.6637	7640.8524	743.2469	49.9768	
9.991	MM m	0.7873	7647.9600	675.5823	50.0232	

Enantioenriched

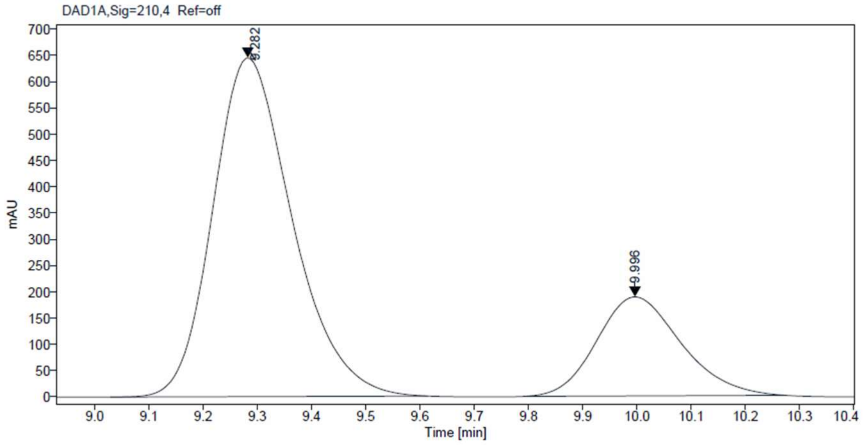

Signal: DAD1A,Sig=210,4 Ref=off

RT [min]	Type	Width [min]	Area	Height	Area%	Name
9.282	MM m	0.5850	6561.1935	644.9908	76.2992	
9.996	MM m	0.4799	2038.1047	188.5560	23.7008	

(*S*)-1-(4-Aminophenyl)ethan-1-ol (14a)

Racemic

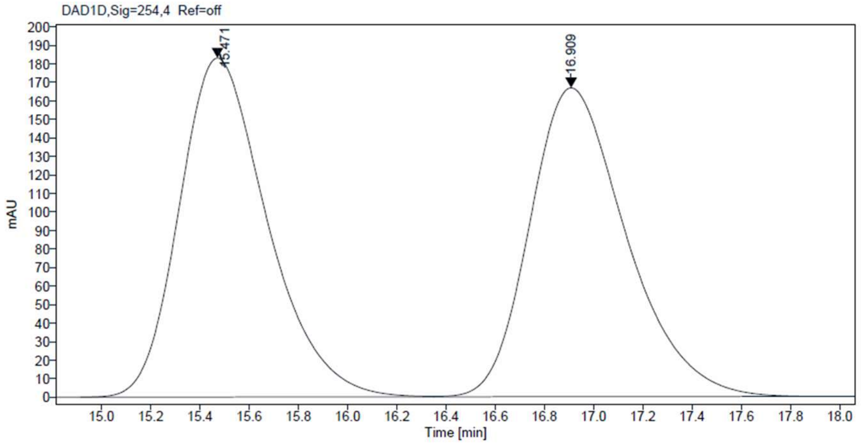

Signal: DAD1D,Sig=254,4 Ref=off

RT [min]	Type	Width [min]	Area	Height	Area%	Name
15.471	MM m	1.4361	4547.2909	183.0229	50.0333	
16.909	MM m	1.5488	4541.2354	166.7329	49.9667	

Enantioenriched

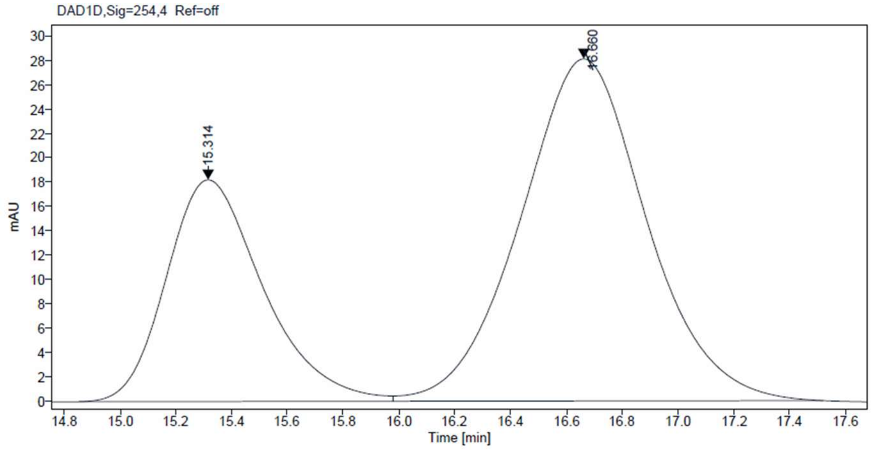

Signal: DAD1D,Sig=254,4 Ref=off

RT [min]	Type	Width [min]	Area	Height	Area%	Name
15.314	MM m	1.1251	438.6803	18.1938	33.2407	
16.660	MM m	1.5425	881.0270	28.0745	66.7593	

(*S*)-1-(*p*-Tolyl)ethan-1-ol (15a)

Racemic

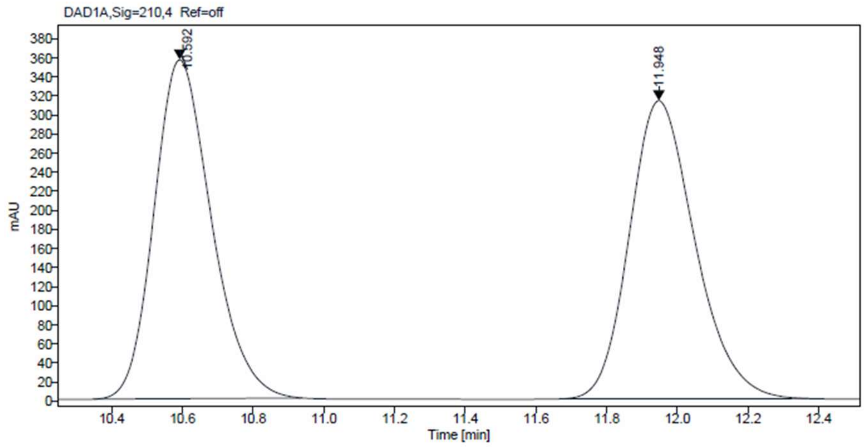

Signal: DAD1A,Sig=210,4 Ref=off

RT [min]	Type	Width [min]	Area	Height	Area%	Name
10.592	MM m	0.5910	4009.1983	355.3309	49.9085	
11.948	MM m	0.7052	4023.9061	312.5586	50.0915	

Enantioenriched

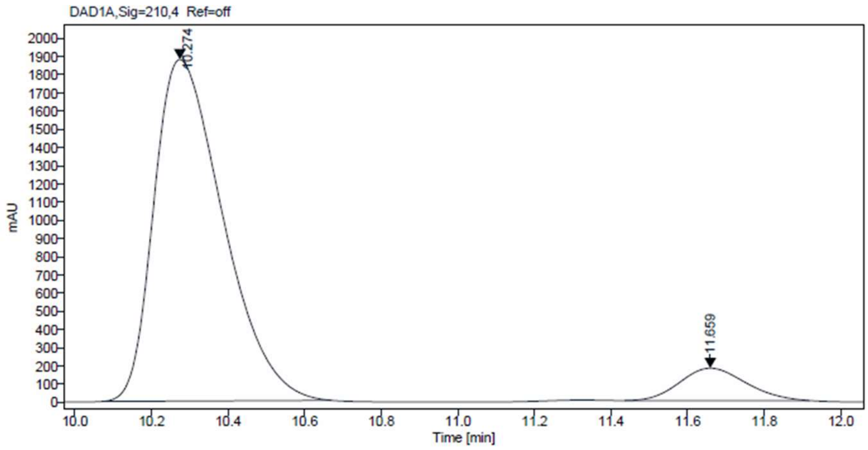

Signal: DAD1A,Sig=210,4 Ref=off

RT [min]	Type	Width [min]	Area	Height	Area%	Name
10.274	MM m	0.5993	23546.8966	1877.7785	91.7088	
11.659	MM m	0.4822	2128.8300	177.9332	8.2912	

(*S*)-1-(4-(*tert*-Butyl)phenyl)ethan-1-ol (16a)

Racemic

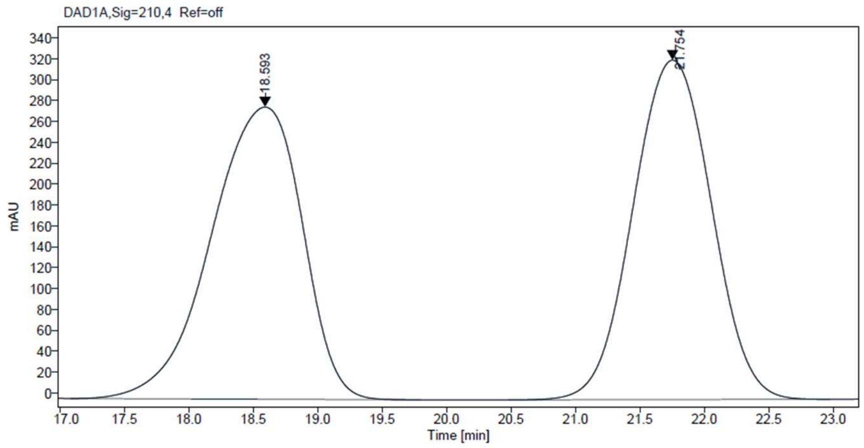

Signal: DAD1A,Sig=210,4 Ref=off

RT [min]	Type	Width [min]	Area	Height	Area%	Name
18.593	MM m	2.7154	13811.2417	279.8542	50.0645	
21.754	MM m	2.4975	13775.6615	325.1298	49.9355	

Enantioenriched

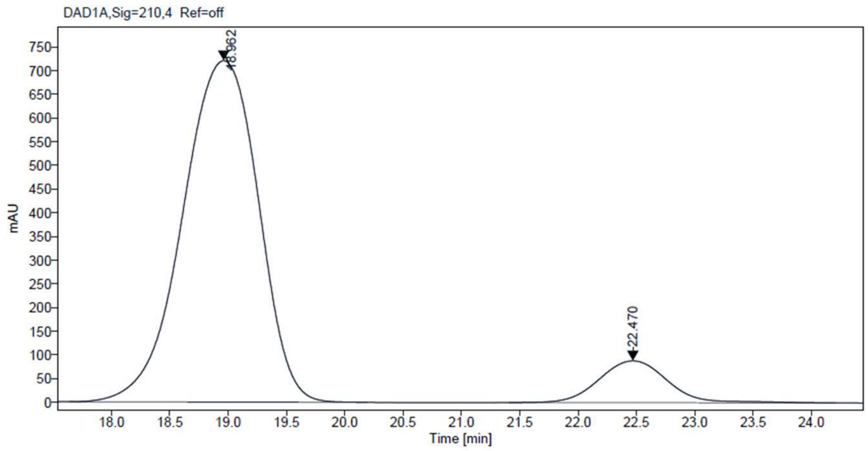

Signal: DAD1A,Sig=210,4 Ref=off

RT [min]	Type	Width [min]	Area	Height	Area%	Name
18.962	MM m	2.5562	32456.5539	720.3884	90.0211	
22.470	MM m	2.7992	3597.8481	88.2248	9.9789	

(*S*)-4-(1-Hydroxyethyl)benzonitrile (17a)

Racemic

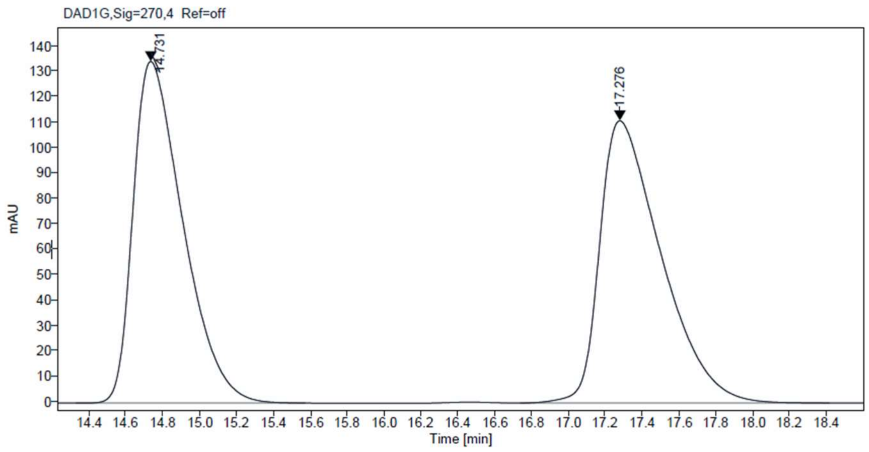

Signal: DAD1G,Sig=270,4 Ref=off

RT [min]	Type	Width [min]	Area	Height	Area%	Name
14.731	MM m	1.2449	2523.7442	134.4407	49.5276	
17.276	MM m	1.6775	2571.8872	111.0738	50.4724	

Enantioenriched

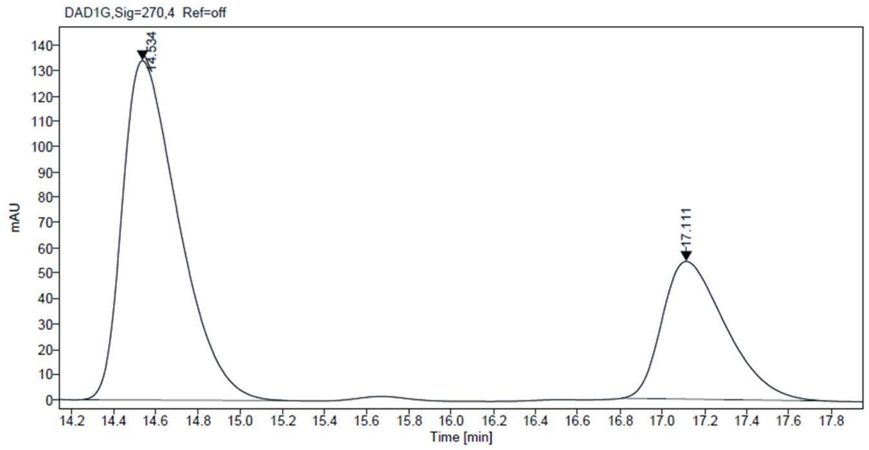

Signal: DAD1G,Sig=270,4 Ref=off

RT [min]	Type	Width [min]	Area	Height	Area%	Name
14.534	MM m	0.9875	2480.7335	134.1201	68.5337	
17.111	MM m	0.9680	1138.9968	54.3972	31.4663	

(*S*)-4-(1-Hydroxyethyl)phenol (18a)

Racemic

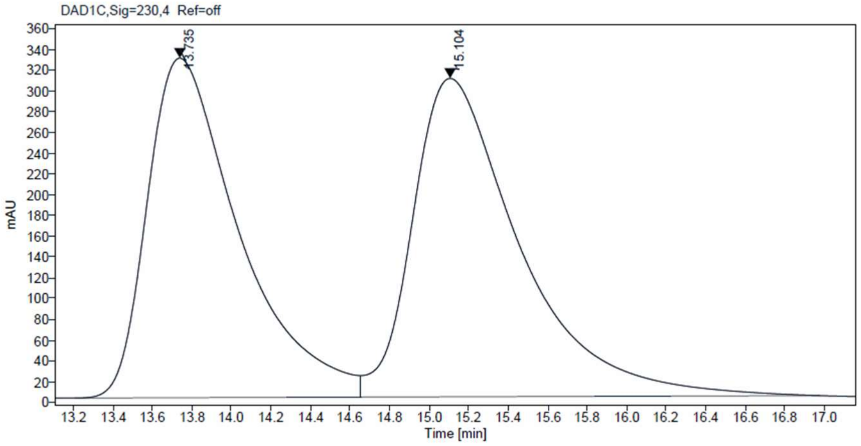

Signal: DAD1C,Sig=230,4 Ref=off

RT [min]	Type	Width [min]	Area	Height	Area%	Name
13.735	MM m	1.4457	10566.3216	327.0170	47.7775	
15.104	MM m	2.3276	11549.3800	306.6785	52.2225	

Enantioenriched

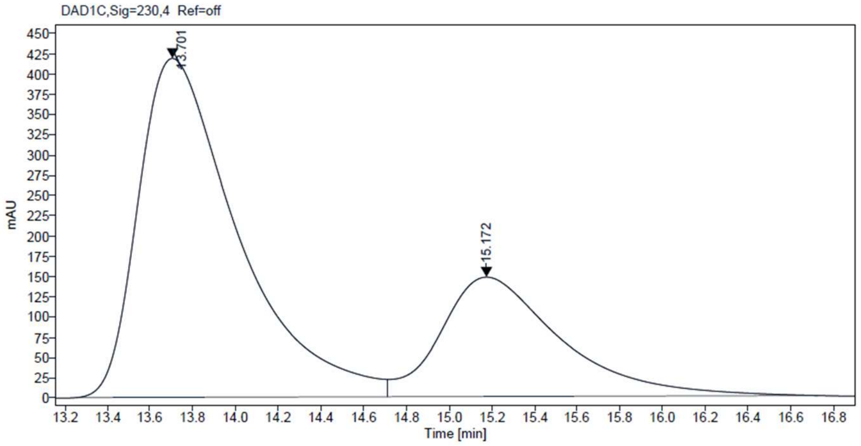

Signal: DAD1C,Sig=230,4 Ref=off

RT [min]	Type	Width [min]	Area	Height	Area%	Name
13.701	MM m	1.4562	13562.3085	418.0527	70.2673	
15.172	MM m	2.0163	5738.7064	147.1993	29.7327	

(*S*)-1-([1,1'-Biphenyl]-4-yl)ethan-1-ol (19a)

Racemic

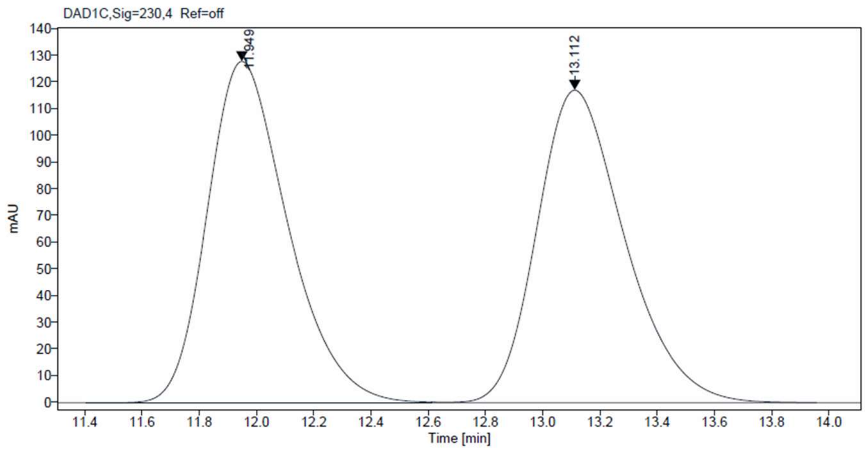

Signal: DAD1C,Sig=230,4 Ref=off

RT [min]	Type	Width [min]	Area	Height	Area%	Name
11.949	MM m	1.2080	2498.0105	127.8912	49.9874	
13.112	MM m	1.3441	2499.2691	117.1147	50.0126	

Enantioenriched

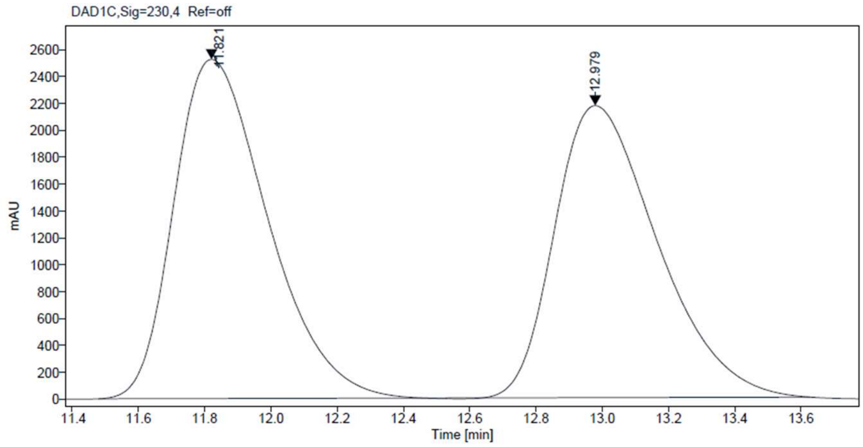

Signal: DAD1C,Sig=230,4 Ref=off

RT [min]	Type	Width [min]	Area	Height	Area%	Name
11.821	MM m	1.0498	49602.5904	2522.9333	51.4764	
12.979	MM m	1.0982	46757.2069	2172.4392	48.5236	

(*S*)-1-(Pyridin-2-yl)ethan-1-ol (20a)

Racemic

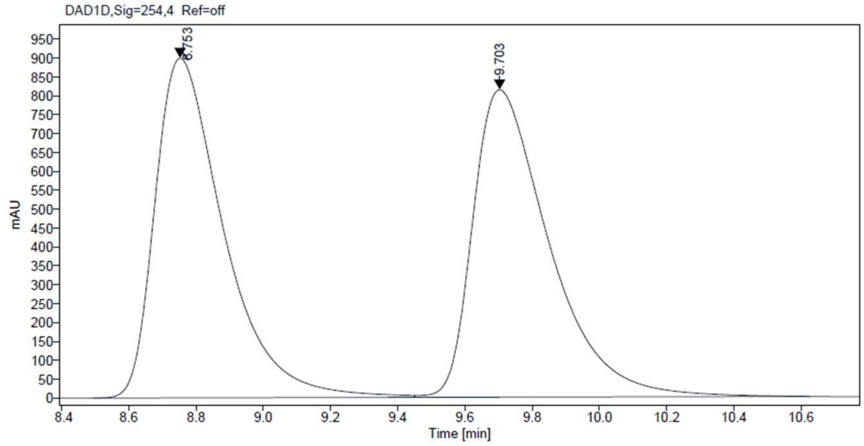

Signal: DAD1D,Sig=254,4 Ref=off

RT [min]	Type	Width [min]	Area	Height	Area%	Name
8.753	MM m	0.9568	12789.5657	899.7410	49.8745	
9.703	MM m	1.1776	12853.9363	814.2907	50.1255	

Enantioenriched

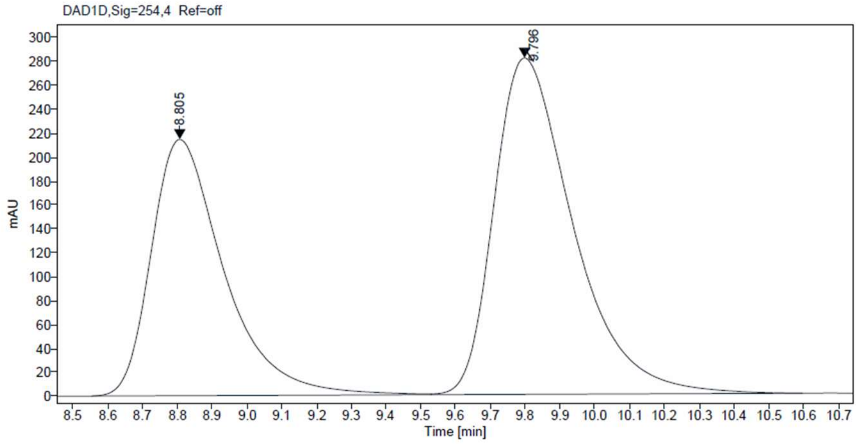

Signal: DAD1D,Sig=254,4 Ref=off

RT [min]	Type	Width [min]	Area	Height	Area%	Name
8.805	MM m	0.9755	3113.2207	214.4744	41.2026	
9.796	MM m	1.0679	4442.6649	281.4014	58.7974	

(*S*)-1-(Pyridin-3-yl)ethan-1-ol (21a)

Racemic

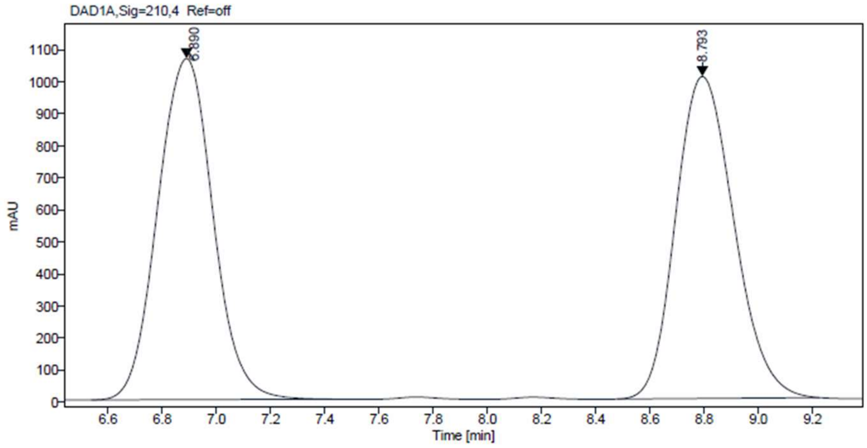

Signal:	DAD1A,Sig=210,4 Ref=off					
RT [min]	Type	Width [min]	Area	Height	Area%	Name
6.890	MM m	0.8939	15104.7521	1065.4199	50.1503	
8.793	MM m	0.7476	15014.2308	1005.8601	49.8497	

Enantioenriched

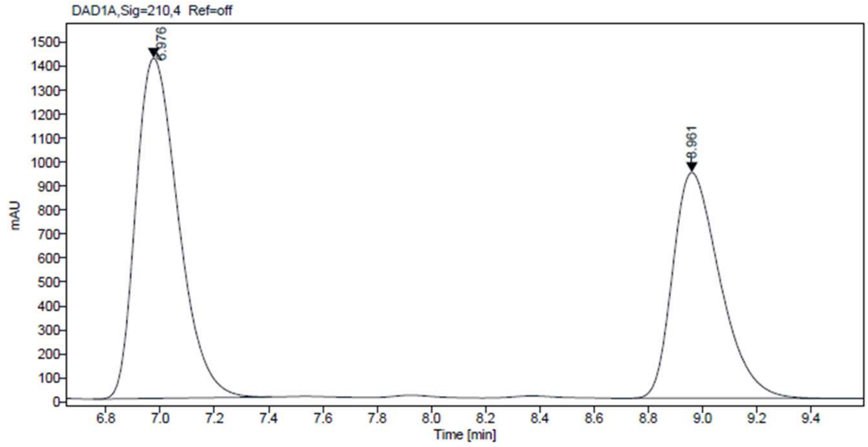

Signal:	DAD1A,Sig=210,4 Ref=off					
RT [min]	Type	Width [min]	Area	Height	Area%	Name
6.976	MM m	0.6575	15417.8280	1418.1336	57.5444	
8.961	MM m	0.6885	11375.1136	941.1760	42.4556	

(*S*)-1-(Pyridin-4-yl)ethan-1-ol (22a)

Racemic

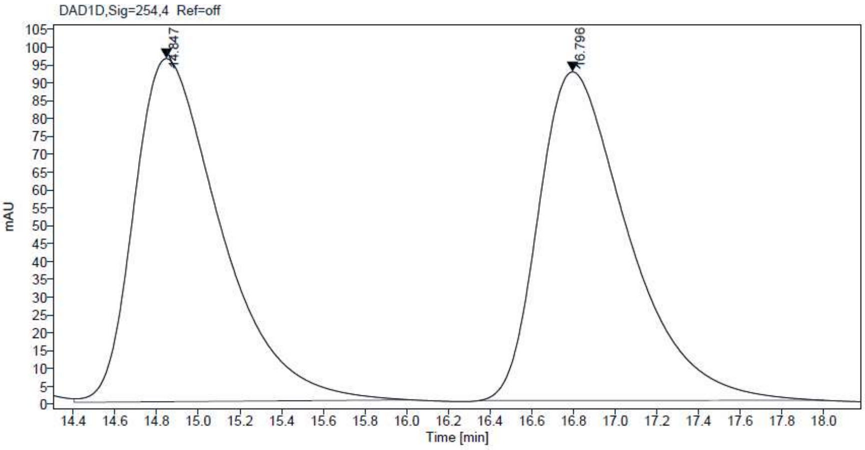

Signal: DAD1D,Sig=254,4 Ref=off

RT [min]	Type	Width [min]	Area	Height	Area%	Name
14.847	MM m	1.6234	2780.3724	96.0642	50.2366	
16.796	MM m	1.6516	2754.1799	92.0001	49.7634	

Enantioenriched

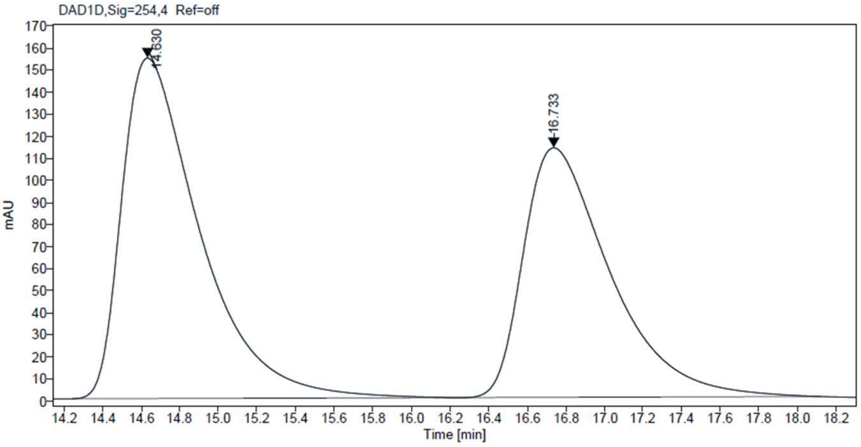

Signal: DAD1D,Sig=254,4 Ref=off

RT [min]	Type	Width [min]	Area	Height	Area%	Name
14.630	MM m	2.0268	4344.4830	154.4270	55.1536	
16.733	MM m	1.8526	3532.5848	113.1566	44.8464	

(*S*)-1-(Pyrimidin-4-yl)ethan-1-ol (23a)

Racemic

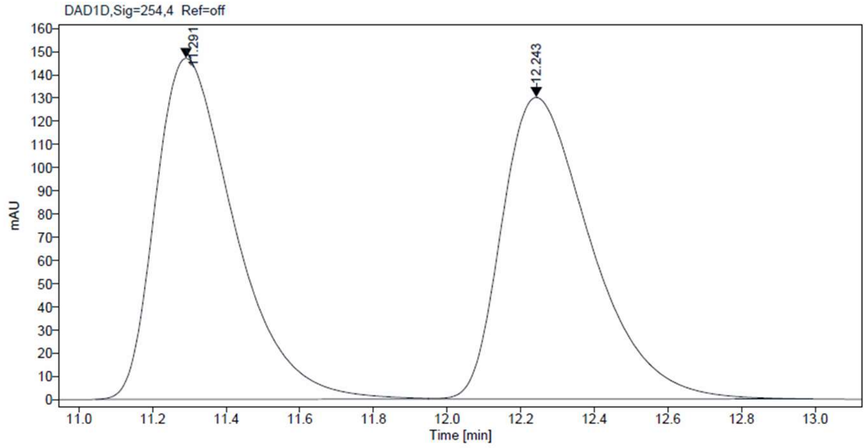

Signal: DAD1D,Sig=254,4 Ref=off

RT [min]	Type	Width [min]	Area	Height	Area%	Name
11.291	MM m	0.9048	2197.7925	147.0467	50.1339	
12.243	MM m	1.0354	2186.0502	130.0988	49.8661	

Enantioenriched

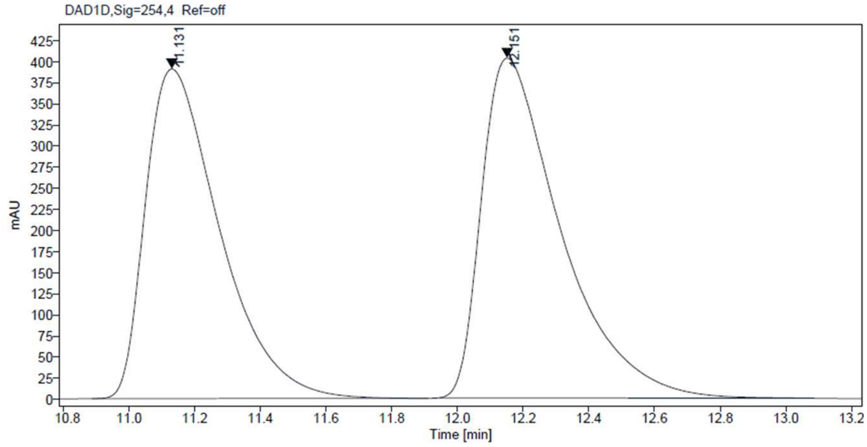

Signal: DAD1D,Sig=254,4 Ref=off

RT [min]	Type	Width [min]	Area	Height	Area%	Name
11.131	MM m	1.0247	6119.6720	390.6036	47.5476	
12.151	MM m	1.1386	6750.9484	402.6260	52.4524	

(*S*)-1-(Thiophen-2-yl)ethan-1-ol (24a)

Racemic

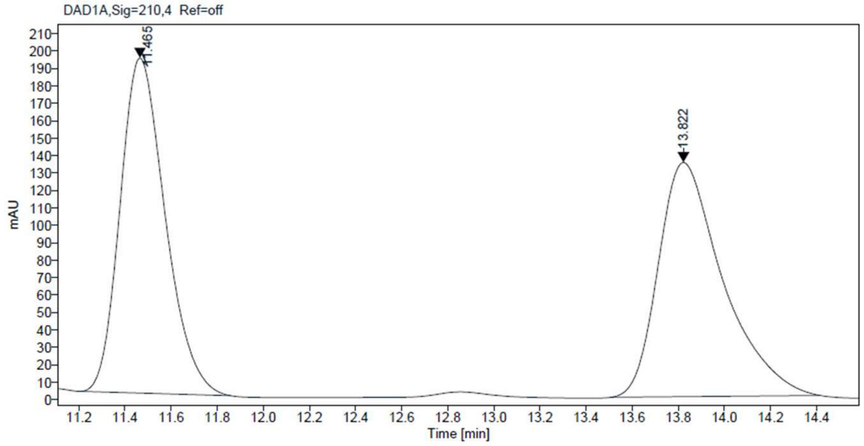

Signal: DAD1A,Sig=210,4 Ref=off

RT [min]	Type	Width [min]	Area	Height	Area%	Name
11.465	MM m	0.6511	2614.1502	192.3081	50.0416	
13.822	MM m	0.9061	2609.8091	134.3669	49.9584	

Enantioenriched

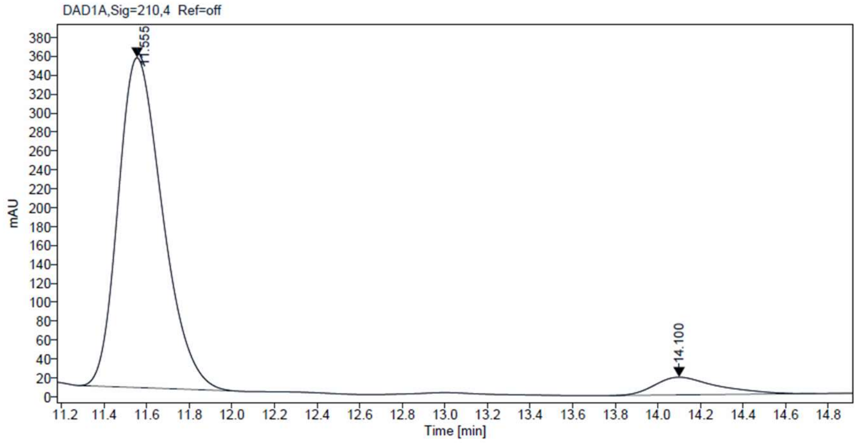

Signal: DAD1A,Sig=210,4 Ref=off

RT [min]	Type	Width [min]	Area	Height	Area%	Name
11.555	MM m	0.7358	5044.5023	348.8564	92.9625	
14.100	MM m	0.9689	381.8793	18.7138	7.0375	

(*S*)-1-Phenylpropan-1-ol (25a)

Racemic

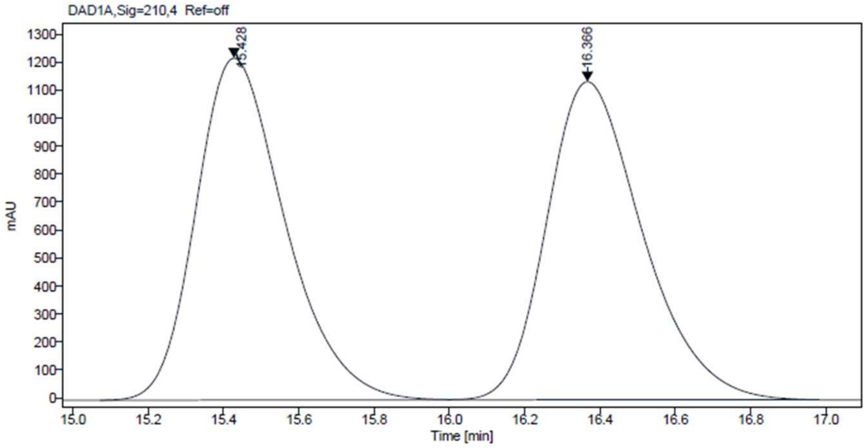

Signal: DAD1A,Sig=210,4 Ref=off

RT [min]	Type	Width [min]	Area	Height	Area%	Name
15.428	MM m	0.9261	20107.6198	1222.0516	49.9962	
16.366	MM m	0.9536	20110.6893	1136.5710	50.0038	

Enantioenriched

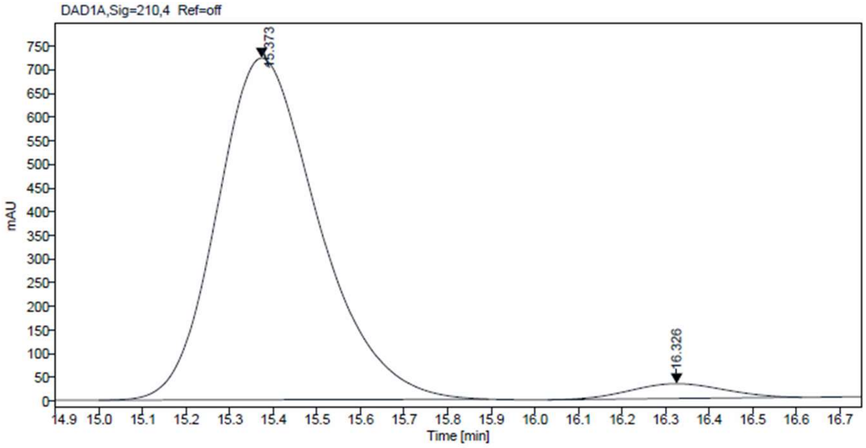

Signal: DAD1A,Sig=210,4 Ref=off

RT [min]	Type	Width [min]	Area	Height	Area%	Name
15.373	MM m	0.8956	11687.2522	722.4260	96.1544	
16.326	MM m	0.5830	467.4256	31.2975	3.8456	

(*S*)-1-phenylbutan-1-ol (26a)

Racemic

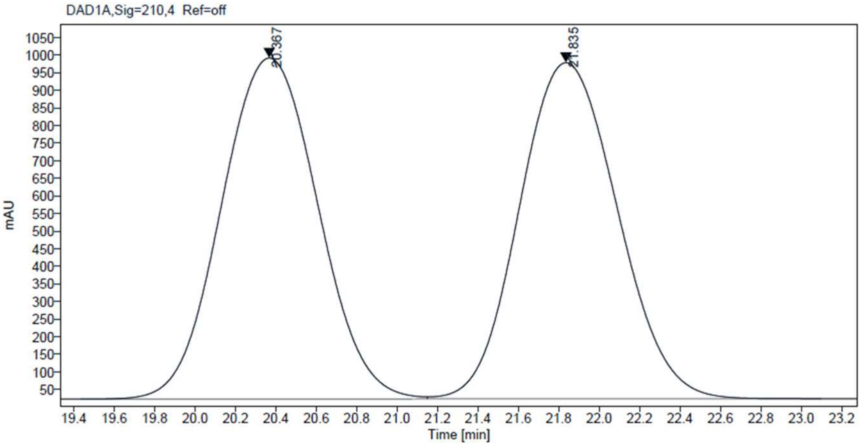

Signal: DAD1A,Sig=210,4 Ref=off

RT [min]	Type	Width [min]	Area	Height	Area%	Name
20.367	MM m	1.7154	32143.4085	968.6905	49.9768	
21.835	MM m	1.9496	32173.2484	954.8932	50.0232	

Enantioenriched

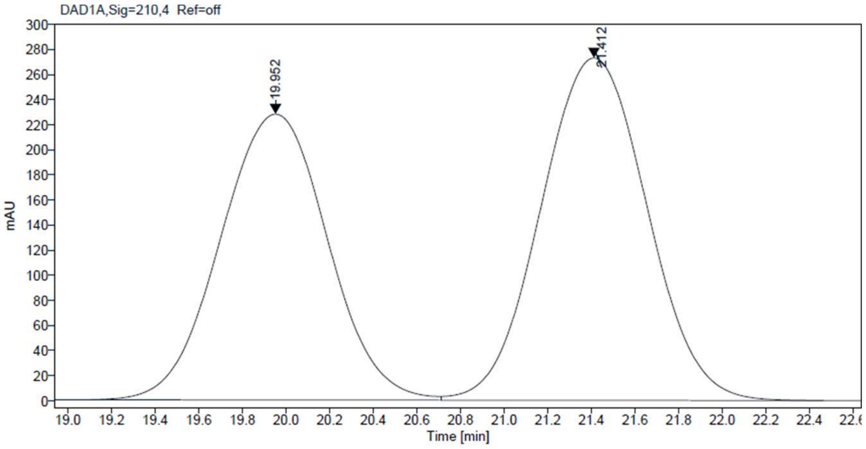

Signal: DAD1A,Sig=210,4 Ref=off

RT [min]	Type	Width [min]	Area	Height	Area%	Name
19.952	MM m	1.6736	7782.4431	227.8637	45.7701	
21.412	MM m	1.7526	9220.8848	272.8651	54.2299	
